# Changes in lipid metabolism driven by steroid signalling modulate proteostasis in *C. elegans*

**DOI:** 10.15252/embr.202255556

**Published:** 2023-04-27

**Authors:** Ana P Gómez‐Escribano, Carlos Mora‐Martínez, Marta Roca, Denise S Walker, Joaquín Panadero, Maria D Sequedo, Ratni Saini, Hans‐Joachim Knölker, Jose Blanca, Juan Burguera, Agustin Lahoz, Joaquin Cañizares, José M Millán, Nick O Burton, William R Schafer, Rafael P Vázquez‐Manrique

**Affiliations:** ^1^ Laboratory of Molecular, Cellular and Genomic Biomedicine Instituto de Investigación Sanitaria La Fe Valencia Spain; ^2^ Centro de Investigación Biomédica en Red de Enfermedades Raras (CIBERER) Valencia Spain; ^3^ Joint Unit for Rare Diseases IIS La Fe‐CIPF Valencia Spain; ^4^ Institute of Biotechnology University of Helsinki Helsinki Finland; ^5^ Unidad Analítica Instituto de Investigación Sanitaria La Fe Valencia Spain; ^6^ Neurobiology Division, MRC Laboratory of Molecular Biology Cambridge Biomedical Campus Cambridge UK; ^7^ Unidad Genómica‐Bioinformática Instituto de Investigación Sanitaria La Fe Valencia Spain; ^8^ Fakultät Chemie Technische Universität Dresden Dresden Germany; ^9^ Instituto Universitario de Conservación y Mejora de la Agrodiversidad Valenciana Valencia Spain; ^10^ Department of Neurology Hospital Universitario y Politécnico La Fe Valencia Spain; ^11^ Unidad de Biomarcadores y Medicina de Precisión, Unidad Analítica Instituto de Investigación Sanitaria, Fundación Hospital La Fe Valencia Spain; ^12^ Van Andel Institute Grand Rapids MI USA

**Keywords:** *Caenorhabditis elegans*, fat metabolism, nuclear receptors, protein aggregation, steroid hormone signalling, Metabolism, Molecular Biology of Disease, Neuroscience

## Abstract

Alzheimer's, Parkinson's and Huntington's diseases can be caused by mutations that enhance protein aggregation, but we still do not know enough about the molecular players of these pathways to develop treatments for these devastating diseases. Here, we screen for mutations that might enhance aggregation in *Caenorhabditis elegans*, to investigate the mechanisms that protect against dysregulated homeostasis. We report that the stomatin homologue UNC‐1 activates neurohormonal signalling from the sulfotransferase SSU‐1 in ASJ sensory/endocrine neurons. A putative hormone, produced in ASJ, targets the nuclear receptor NHR‐1, which acts cell autonomously in the muscles to modulate polyglutamine repeat (polyQ) aggregation. A second nuclear receptor, DAF‐12, functions oppositely to NHR‐1 to maintain protein homeostasis. Transcriptomics analyses of *unc‐1* mutants revealed changes in the expression of genes involved in fat metabolism, suggesting that fat metabolism changes, controlled by neurohormonal signalling, contribute to protein homeostasis. Furthermore, the enzymes involved in the identified signalling pathway are potential targets for treating neurodegenerative diseases caused by disrupted protein homeostasis.

## Graphical abstract

A neurohormonal signal emanating from *Caenorhabditis elegans* ASJ neurons modulates protein homeostasis in the body wall muscles. The signal depends on the stomatin homologue UNC‐1 in neurons and the nuclear receptor NHR‐1 in muscles.

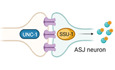

## Introduction

Many neurodegenerative diseases, such as Huntington's (HD), Alzheimer's and Parkinson's diseases, and Amyotrophic lateral sclerosis (ALS) are caused by toxic protein aggregates that stem from incorrect protein folding (Valastyan & Lindquist, 2014). In non‐disease states, this aggregation is prevented via natural protein homeostasis mechanisms that ensure an adequate expression of proteins, proper folding and localization of these molecules, and the removal of misfolded proteins. Many pathways and molecules have been shown to influence this maintenance of appropriately folded proteins and their location in the cellular environment. For example, deleterious mutations in genes encoding components of chemical synapses accelerate the decline of protein homeostasis in post‐synaptic muscle cells and contribute to the progression of age‐related disorders (Garcia *et al*, 2007). However, though much is known about these pathways, we still do not know enough about all of the molecular players and their mechanisms to develop treatments for these devastating diseases.

In a properly functioning homeostasis system, the accumulation of a considerable number of misfolded proteins would induce cytoplasmic stress by poisoning the machinery of the autophagic and proteasomal systems. This in turn activates the unfolded protein response (UPR) pathways in the endoplasmic reticulum, mitochondria and cytosol (Almanza *et al*, 2019). UPR signals activate nuclear transcription factors that induce the expression of genes associated with folding and protein degradation, autophagy and other protective pathways (Almanza *et al*, 2019). Proteostasis can also be modulated by the metabolic status of the organism and has been shown to be connected to lipid metabolism (Steinbaugh *et al*, 2015; Webster *et al*, 2017; Lee *et al*, 2019; Higuchi‐Sanabria *et al*, 2020; Joshi *et al*, 2021). For example, inhibiting the genes that encode amino‐acyl tRNA synthetases increases fat production and activates the proteasomal AMPK network to extend survival under starvation conditions (Webster *et al*, 2017). In addition, the lipid homeostasis regulator MDT‐15 is required for maintaining HSF‐1‐dependent proteostasis at low temperatures in *Caenorhabditis elegans* expressing a polyQ (*35Q::YFP*) (Lee *et al*, 2019). Lipids can also modulate proteostasis via lipid depletion through overexpression of XBP‐1s in serotonergic, but not dopaminergic, neurons (Higuchi‐Sanabria *et al*, 2020). Moreover, XBP‐1s remodels lipid metabolism by non‐cell autonomous signalling, which decreases triglyceride and increases oleic acid levels (Imanikia *et al*, 2019). In addition, the folding of proteins containing expanded polyQs in muscle cells can be regulated by chemical synaptic functions in neurons (Garcia *et al*, 2007; Silva *et al*, 2013). For example, a small increase in physiological cholinergic signalling at motor synapses induces a calcium‐dependent activation of muscular HSF‐1, which in turn activates the expression of chaperones that protect against protein aggregation (Silva *et al*, 2013). Additionally, Taylor & Dillin (2013) described another non–cell autonomous mechanism by XBP‐1 to regulate UPR pathways and longevity in *C. elegans*. However, despite these and similar observations, the mechanisms by which neuronal signalling controls protein aggregation in distant tissues remains unknown.

Nuclear receptors comprise a group of transcription factors that regulate the expression of genes in a ligand–binding‐dependent manner (Chawla, 2001; Antebi, 2015). These ligands are usually lipid molecules, such as steroid hormones, vitamins (D_3_ and A), metabolites, and xenobiotics (Sever & Glass, 2013). Sometimes required for receptor binding and promoting hormonal signalling to distal tissues, these ligands require processing by sulfotransferases, which add a sulphate moiety, and sulfatases, which remove the sulphate. In terms of lipid metabolism, Joshi *et al* (2021) claim that sensory neurons secrete biogenic amines to modulate lipid signalling, which in turn activates UPR pathways to maintain proteostasis. This work is evidence that sensory neurons can release signalling molecules to regulate gene expression.

*Caenorhabditis elegans* has 284 nuclear receptor genes, compared to 48 in humans (Maglich *et al*, 2001). One of the best‐known nuclear receptors in *C. elegans*, DAF‐12, regulates lipid metabolism, lifespan and development (Antebi *et al*, 2000; Ludewig, 2004; Wang *et al*, 2015) through the dafachronic acid hormones. This control depends on the tight modulation of the expression of genes encoding key metabolic enzymes, which includes negative feedback loops operated by their enzymatic products (Bi *et al*, 2018). For example, NHR‐49, a homologue of the Hepatocyte Nuclear Factor 4‐α (HNF4α) (Goh *et al*, 2018), has a crucial role in the regulation of lipid metabolism, longevity and nutrient response (Gilst *et al*, 2005). Following activation, nuclear receptors dimerise, producing homo‐ and heterodimers, and bind DNA domains in the vicinity of promoters to regulate gene expression, sometimes in opposite manners depending on the nuclear receptor partner. For instance, heterodimeric NHR‐49/NHR‐80 promotes lipid desaturation while NHR‐49/NHR‐66 blocks sphingolipid metabolism and lipid remodelling (Pathare *et al*, 2012). Among the lipids regulated by these nuclear receptors, oleic acid has been shown to promote longevity in germline ablated mutants (Goudeau *et al*, 2011). This lipid is also able to enhance proteostasis, in worms expressing polyQs, via XBP‐1 signalling (Imanikia *et al*, 2019). Oleic acid is synthesised by several key enzymes, that are encoded in the *C. elegans* genome: FAT‐6 and FAT‐7, whose expression is regulated by NHR‐80 (Watts & Browse, 2002). Overall, though extracellular signalling has been shown to modulate protein homeostasis the upstream and downstream processes involved in this regulation are not completely understood. Nor is it clear whether other synaptic‐related phenotypes that may influence protein homeostasis.

Here, we studied these pathways in *C. elegans* via a chemical mutagenesis screen of worms expressing polyQs in muscle cells, which allowed us to isolate a loss‐of‐function allele of *unc‐1* that enhances protein aggregation and alters motor coordination. We show that the stomatin homologue UNC‐1 functions in neurons, likely by modulating electrical synapses, to regulate protein homeostasis by non‐cell autonomous signalling. Disrupting *unc‐1* disrupts a key electrical synapse, likely then causing the secretion of excess sulphated signal that activates NHR‐1 to downregulate genes encoding enzymes for fat metabolism, which in turn disrupts proteostasis. We also show that signalling through the well‐known DAF‐12 functions antagonistically to NHR‐1 to control protein aggregation. These results provide the first evidence that nuclear receptors modulate polyQ aggregation by remodelling lipid metabolism. Moreover, some of the enzymes involved in the NHR–1‐activating signalling pathway are potential druggable targets, which may be used to treat neurodegenerative diseases caused by protein homeostasis disruption.

## Results

### A forward genetic screen identifies *unc‐1* as a modulator of polyQ aggregation

**Figure 1:**
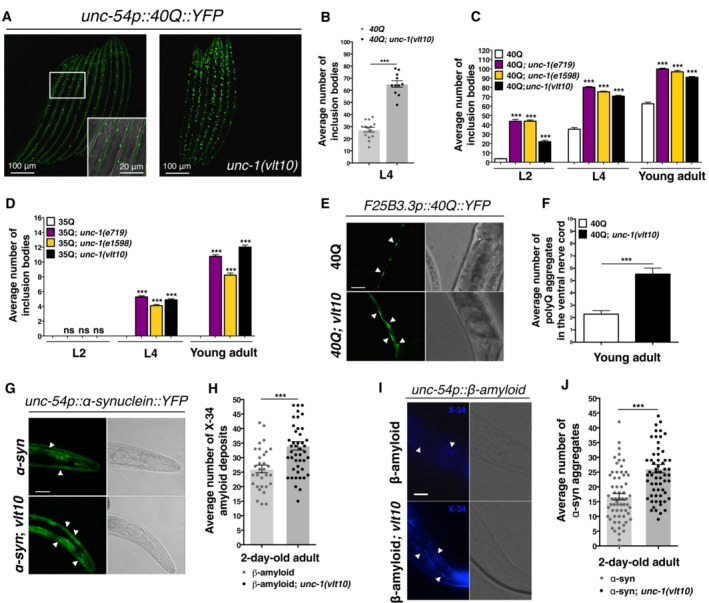
UNC‐1 modulates protein aggregation Representative confocal microscopy images of L4 40Q and 40Q; *unc‐1(vlt10)* mutant worms. The inset (20× further zoom) shows the shape and pattern of the inclusion bodies in muscle cells (white arrows).The mean number of polyQ inclusion bodies in homozygous *unc‐1(vlt10)* animals.The mean number of polyQ inclusion bodies in 40Q animals carrying *e719* and *e1598* alleles of *unc‐1* at different stages (L2, L4 and young adult).The mean number of polyQ inclusion bodies in 35Q animals carrying *e719* and *e1598* alleles of *unc‐1* at different stages (L2, L4 and young adult).Representative images from fluorescence microscopy showing neuronal aggregates of polyQs (white arrows) in the ventral nerve cord of F25B3.3p::40Q::YFP and F25B3.3p::40Q::YFP; *unc‐1 (vlt10)* young adult animals.The mean number of neuronal aggregates of polyQs in (E).Representative images from confocal microscopy showing the area of the muscle tissue between the two pharyngeal bulbs where α‐synuclein aggregates are located (white arrows).The counted average number of α‐synuclein aggregates in (G).Representative fluorescence microscopy images of the area between the two pharyngeal bulbs highlighting amyloid deposits (white arrows).The counted average number of amyloid deposits in (I). Data information: All plotted data show the mean ± standard error of the mean (SEM). At least 30 animals were tested for each condition for all graphs except for (G), which used 60 animals. Each analysis has been reproduced at least three independent times. ***P < 0.001; ns: not significant. The analyses in (B, F, H and J) were done using a Mann–Whitney *U*‐test, and those in (C and D) were done using a one‐way ANOVA with *post‐hoc* Tukey test. Scale bar: 20 μm unless otherwise specified. Source data are available online for this figure.

To expand our knowledge of the signalling pathways controlling protein homeostasis, we performed a chemical mutagenesis screen for genes regulating protein aggregation in a worm model of polyQ diseases. For this screen, we used animals that express a transgene containing a 40‐glutamine repeat fused to a yellow fluorescent protein (40Q::YFP) in muscle cells. In these animals, 40Q aggregates in an age‐dependent manner and forms inclusion bodies that can be observed and counted under a dissecting microscope. Though several mutants were identified from this screen (Appendix Table S1), only one matched our criteria, that carrying the *vlt10* allele in *unc‐1* (Appendix Fig S1A and B, Appendix Supplementary Methods), which substantially enhanced aggregation without affecting 40Q transgene expression (Fig 1A and B, Appendix Fig S1A and B). In addition to enhanced polyQ aggregation, the *unc‐1(vlt10)* animals showed an uncoordinated phenotype, suggesting that this mutation may affect the synaptic function of the nervous system and/or the muscle cells, both tissues in which *unc‐1* is expressed (Rajaram *et al*, 1998, 1999; Sedensky *et al*, 2001; Chen *et al*, 2007).

### Depletion of *unc‐1* enhances polyQ aggregation

To confirm that the loss of function of *unc‐1* is responsible for the enhanced aggregation phenotype of *unc‐1(vlt10)* mutants, we examined the effect of other mutant alleles of *unc‐1* (*e719* and *e1598*) on animals expressing two polyQs with different repeat lengths, 35Q and 40Q (which we will refer to as 35Q or 40Q animals). In both cases, analysis of the inclusion bodies produced by polyQ aggregation showed that mutations in *unc‐1* (*e719* and *e1598*) phenocopy the aggregation pattern of *vlt10* worms (Fig 1C and D) without altering *40Q::YFP* transgene expression (Appendix Fig S1C and D). In addition, we investigated whether *unc‐1(vlt10)* would modify the late aggregation phenotype of a strain expressing 35Q fused to YFP (35Q::YFP) in body wall muscles, which produces both a later and weaker phenotype than animals expressing 40Q and inclusion bodies at late adult stages (Fig 1D). In 35Q; *unc‐1(vlt10)* animals, the *vlt10* allele enhanced and accelerated the phenotype of these animals to a similar extent as other *unc‐1* alleles, *e179* and *e1598* (Fig 1D). These results indicate that *unc‐1* modulates polyQ aggregation in muscle cells.

As *unc‐1* is expressed in neurons, we investigated whether *vlt10* affected polyQ aggregation in this tissue using a worm strain expressing 40Q across the entire nervous system. We introduced the *unc‐1(vlt10)* mutation, and as expected, *unc‐1(vlt10)* increased neuronal aggregate formation in the ventral nerve cord of young adults, further demonstrating the requirement of *unc‐1* in protein homeostasis (Fig 1E and F). Altogether, these data indicate that *unc‐1* is required to prevent the aggregation of polyQ‐containing proteins, both in muscle cells and neurons.

### *unc‐1(vlt10)* enhances α‐synuclein and β‐amyloid aggregation

Having shown that *unc‐1* regulates polyQ aggregation, we tested whether its function is polyQ‐specific or if it affects other aggregation‐prone proteins, such as α‐synuclein and β‐amyloid that aggregate in brains of Parkinson's and Alzheimer's patients, respectively. To do this, we introduced the *unc‐1(vlt10)* allele into transgenic worms expressing α‐synuclein::YFP (referred to as α‐syn) and worms expressing human β‐amyloid in body wall muscles. In contrast with worms expressing polyQs, which show aggregation early in the life cycle of worms, both α‐syn and β‐amyloid aggregates do not appear until later stages of development (2‐day‐old adults). Quantifying the aggregate numbers showed that the loss of *unc‐1* function increased both α‐syn and β‐amyloid aggregation in day 2 adults compared with controls (Fig 1G–J). These data suggest that *unc‐1*, rather than being polyQ‐specific, is a general modulator of protein homeostasis.

### *unc‐1* required in nervous system to maintain non–cell autonomous protein homeostasis

To investigate whether UNC‐1 also modulates muscle aggregation in a cell‐autonomous manner, as it is expressed throughout both the nervous system and muscle cells, we examined whether the aggregation phenotype of *40Q*; *unc‐1(vlt‐10)* animals could be rescued via the expression of *unc‐1* cDNA in the muscles. Indeed, expressing *unc‐1* under the control of the muscle‐specific *myo‐3* promoter (*myo‐3p*) appeared to partially rescue polyQ aggregation, since we observed a decrease in YFP‐labelled aggregates (Appendix Fig S2A and B, Appendix Table S2). However, this was likely an artefact of altered polyQ expression (Appendix Fig S2A and B).

**Figure 2:**
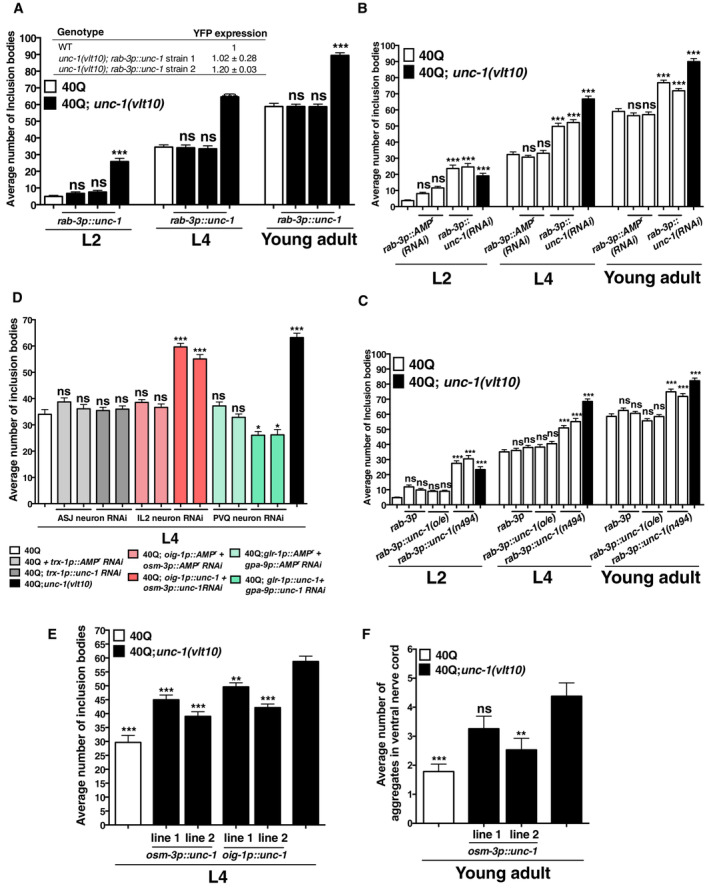
UNC‐1 is required in IL2 neurons to modulate protein homeostasis in muscle cells and neurons The cDNA of *unc‐1* was expressed in the whole nervous system of worms, and the rescue of polyQ aggregation in *unc‐1* mutants was measured at several stages (L2, L4 and young adult) by counting the number inclusion bodies per animal. We produced two independent transgenic stable lines (line 1 and line 2), carrying the *rab‐3p::unc‐1*(cDNA) transgene. The inserted table shows the expression of the *40Q::YFP* transgene as determined by qRT–PCR using a specific probe for YFP and normalised to *pmp‐3* gene.The average number of polyQ inclusion bodies in muscle cells after the tissue‐specific expression of RNAi against *unc‐1* in the nervous system. We expressed dsRNA of *unc‐1* specifically in neurons (using promoter of *rab‐3*) of 40Q::YFP worms, to evaluate polyQ inclusion bodies after silencing.The average number of polyQ inclusion bodies in muscle cells, in the presence of overexpressed (o/e) *unc‐1*, and its dominant negative allele *n494* in the nervous system.The average number of polyQ inclusion bodies in muscle cells, after the expression of RNAi against *unc‐1*, in ASJ, IL2 and PVQ neurons using specific promoters or combination of them.The average number of polyQ inclusion bodies in muscle cells after the cDNA of *unc‐1* was reintroduced in *unc‐1(vlt10)* mutants expressing 40Q::YFP in muscle cells using the promoters from either *osm‐3* or *oig‐1*, separately, which would express the cDNA in some neurons, including IL2. We produced two independent transgenic stable lines (line 1 and line 2), carrying the *osm‐3p::unc‐1(cDNA)* transgene, and two independent transgenic stable lines (line 1 and line 2) carrying *oig‐1p::unc‐1(cDNA)* transgene. Comparisons are with *unc‐1(vlt10)*.The same extrachromosomal arrays as in (E), that drive the expression of *osm‐3p::unc‐1(cDNA)* in IL2 neurons, were introduced in *unc‐1(vlt10)* mutants, that express 40Q::YFP in the whole nervous system. Then, we analysed the average number of ventral nerve cord aggregates. Comparisons are with *unc‐1(vlt10)*. Data information: Bars indicate mean ± SEM. Thirty animals were tested per strain and/or condition. Each analysis has been reproduced at least three independent times. ***P < 0.001; **P < 0.01; *P < 0.05; ns: not significant, as calculated using one‐way ANOVA with *post‐hoc* Tukey test. Source data are available online for this figure.

In contrast, when we expressed *unc‐1* across the nervous system using the pan‐neuronal promoter of the *rab‐3* gene, *unc‐1(vlt10)* animals had substantially reduced polyQ aggregation in all stages (Fig 2A), with no effect on the expression of the transgene (Fig 2A). To confirm these results, we knocked‐down *unc‐1* expression in a tissue‐specific manner using RNAi by expressing double‐stranded RNAs against the gene across the entire nervous system (*rab‐3* promoter) using transgenesis (Appendix Fig S8). As a negative control, we used RNAi against the ampicillin resistance gene (*AMP* ^*r*^). This silencing of *unc‐1* across the nervous system increased the number of inclusion bodies compared with 40Q and 40Q; *AMP* ^*r*^ *(RNAi)* animals (Fig 2B), further suggesting that polyQ aggregation in muscle cells is controlled from neuronal *unc‐1* expression. Finally, to further demonstrate that *unc‐1* is acting neuronally, we disrupted *unc‐1* function across the nervous system by overexpressing a cDNA containing the *n494* dominant allele of *unc‐1*, again using the *rab‐3* promoter to drive expression in all neurons. These animals exhibited a similar level of inclusion bodies to 40Q; *unc‐1(vlt10)* animals (Fig 2C). Moreover, overexpression of the wild type *unc‐1* gene, which does not alter protein aggregation dynamics, showed that it is not an overload of UNC‐1 that increases polyQ aggregation (Fig 2C). Altogether these results show that UNC‐1 acts in neurons to modulate polyQ aggregation in muscle cells.

### *unc‐1* is required in IL2 neurons to modulate polyQ aggregation

Previous reports showed a genetic interaction between *unc‐1* and *ssu‐1* relating to anaesthetic sensitivity and motor phenotypes in worms (Carroll *et al*, 2006). Since *ssu‐1* is expressed exclusively in a single class of neurons, a pair of amphid sensory neurons called ASJ (Carroll *et al*, 2006), we speculated that *unc‐1* may be required in this cell to modulate polyQ aggregation. To test this, we tried to rescue *unc‐1(vlt10)* by expressing the *unc‐1* cDNA under the control of the *trx‐1* promoter, which expresses only in ASJ neurons. However, we did not observe any change in 40Q aggregation (65.2 ± 9.1 (*unc‐1* mutants) vs. 64.1 ± 7.5 (rescued *unc‐1* mutants), Mean ± SD).

Since UNC‐1 is known to regulate electrical synapses (Chen *et al*, 2007), we then hypothesised that *unc‐1* may be required in neurons that connect with ASJ through gap junctions. As the electrical and chemical synaptic connections of the entire *C. elegans* nervous system have been precisely characterised (Cook *et al*, 2019), we know that ASJ makes electrical synapses with one of the six inner labial IL2 sensory neurons, IL2L, and one of the two PVQ posterior interneurons, PVQR (Cook *et al*, 2019). Thus, we disrupted the function of *unc‐1* specifically in ASJ, IL2 and PVQ using RNAi induced from transgenes. In agreement with our failure to rescue *unc‐1(vlt10)* in ASJ, knockdown of *unc‐1* in ASJ had no effect, supporting the idea that ASJ is not the site of function. In contrast, knockdown in the IL2 neurons significantly increased polyQ aggregation to the same level as the *unc‐1* mutants (Fig 2D), suggesting that *unc‐1* acts in the IL2s to prevent polyQ aggregation. Surprisingly, silencing *unc‐1* in PVQ neurons had the opposite effect, inducing a mild reduction of polyQ aggregation. In both cases, this suggests that interactions of these neurons with ASJ could modulate polyQ aggregation (Fig 2D).

To confirm the role of the IL2s, we tested for cell‐specific rescue by reintroducing the cDNA of *unc‐1* in the IL2 neurons of 40Q; *unc‐1(vlt10)* animals using two different IL2‐specific promoters, *oig‐1p* and *osm‐3p*. Though the expression patterns of these promoters cover several neurons, they overlap exclusively in IL2 neurons, meaning that the RNAi will occur only in IL2 neurons. These animals showed reduced polyQ aggregation in muscle cells and neurons (Fig 2E and F). Altogether, these data confirmed that loss of function of *unc‐1(vlt10)* from IL2 neurons, which are electrically coupled to the ASJ neuron, modulates polyQ aggregation. UNC‐1 may also function by modulating gap junctions (Appendix Table S3, Appendix Figs S3 and S9A), where its function is required (Chen *et al*, 2007). We thus also depleted different gap junction components (innexins) and other stomatin‐like proteins to analyse the aggregation patterns of worms expressing muscular 40Q. Besides *unc‐1*, there are another nine genes that encode stomatin‐like protein in *C. elegans* (Hobert, 2013). As *unc‐1* functions in neurons, we tested whether other neuronally expressed stomatin‐like proteins modulate polyQ aggregation in muscle cells. Four of the genes encoding stomatin‐like proteins are expressed in neurons, *mec‐2*, *unc‐24*, *sto‐1* and *sto‐6* (Carroll *et al*, 2006; Taylor *et al*, 2021) Analysis of two of these genes, *mec‐2* (expressed in mechanosensory neurons) and *unc‐24* (wide neuronal expression), showed that only *unc‐24* mutants have a phenotype comparable to *unc‐1* mutants, suggesting that not all neuronal stomatin‐like proteins are involved in the regulation of polyQ aggregation (Appendix Table S3). Since *unc‐1* modulates electrical synapses (Chen *et al*, 2007), we tested whether innexins (gap junction components) are polyQ aggregation modulators. To test this hypothesis, we introduced mutant alleles of some innexins that are widely expressed in the nervous system (*unc‐9*, *unc‐7* and *inx‐7*), and others that are expressed in a restricted set of neurons (*inx‐2* and *inx‐6*), into a polyQ background (Altun *et al*, 2009) (Appendix Table S3). However, we observed a selective effect where only the ablation of *unc‐7*, *unc‐9* and *inx‐2* enhances polyQ aggregation similar to *unc‐1* mutants (Appendix Table S3).

To confirm the role of neuronal innexins, we performed cell‐specific RNAi against some of the innexin genes, using the pan‐neuronal *rab‐3* promoter. Silencing of *inx‐2* or *unc‐7* increased inclusion body formation, in contrast with *inx‐6(RNAi)* that did not modify the aggregation pattern (Appendix Fig S3A). Our data indicate that disruption of some components of neuronal gap junctions alters polyQ aggregation in muscle cells. We then investigated whether *unc‐1* interacts genetically with *inx‐2* or *unc‐7*. The double mutant 40Q; *unc‐1(vlt10)*; *unc‐7(e5)* produced similar levels of aggregation to the *unc‐1* worms (Appendix Fig S3B). This suggests that the two genes function in the same process. In contrast, 40Q; *unc‐1(vlt10)*; *inx‐2(vlt22)* animals showed an additive effect, suggesting that *unc‐1* and *inx‐2* operate in parallel pathways to modulate protein homeostasis (Appendix Fig S3C). Altogether, these results suggest that neuronal electrical synapses modulate protein homeostasis.

These experiments showed that altering some of these components indeed increased polyQ aggregation in muscle. Our data are consistent with a model in which loss of *unc‐1* function perturbs gap junctions between IL2 and ASJ, resulting in altered signalling from ASJ. We wondered, therefore, whether the *unc‐1* mutation would increase the general excitability of the ASJs, since, by connecting cells, gap junction opening can act to shunt current, with the connecting cell acting as a sink. If so, we reasoned that increased ASJ excitability and signalling could result from disrupted gap junctions between the IL2 and ASJ that would prevent this shunting.

To further investigate this, we used the fact that the ASJ neurons are sensitive to temperature, with previous studies showing that calcium concentrations in these neurons increased upon warming and decreased upon cooling (Ohta *et al*, 2014; Ujisawa *et al*, 2016). Since an increase in ASJ general excitability might be expected to increase overall cellular activity and the response to a temperature stimulus, we examined whether these temperature responses were intact in *unc‐1(vlt‐10)* animals by expressing the genetically encoded ratiometric calcium indicator Cameleon in ASJ. We did not see any difference in the fluorescence ratio, either at the starting temperature of 23°C or after shifting to 17°C (Appendix Fig S4A, B and D), compared to wild type animals, so tonic, global calcium concentrations do not appear to be increased. When we shifted the temperature back to 23°C, we observed a defect in the up‐step in calcium concentration (Appendix Fig S4A, C and E), which was, if anything, more suggestive of a decrease in excitability. Thus, the effect of *unc‐1(vlt10)* on signalling from ASJ does not stem from a general increase in ASJ excitability, so cannot simply be explained by a disruption in the gap junctions eliminating current shunting to IL2.

### Ablation of the sulfotransferase gene *ssu‐1* rescues *unc‐1*‐associated protein homeostasis

It has been shown that the uncoordinated locomotion of *unc‐1*‐defective worms could be partially rescued in mutant alleles of *ssu‐1*, a gene that encodes the only known alcohol cytosolic sulfotransferase in the worm (Carroll *et al*, 2006). Alcohol sulfotransferases are promiscuous enzymes that are able to add a sulphate moiety to a variety of molecules such as peptides and steroid hormones, as well as xenobiotics targeted for elimination (reviewed by Gamage *et al*, 2006). *ssu‐1* expression is restricted to the ASJ pair of sensory neurons, which function in food sensing, hormone release, Dauer formation and temperature sensing (Schackwitz *et al*, 1996; Carroll *et al*, 2006; Hattori *et al*, 2006; Ohta *et al*, 2014). As sulphates are known to be involved in hormone processing, with sulfotransferases adding a sulphate moiety and sulfatases removing it, we wanted to determine what, if any, involvement the *ssu‐1* sulfotransferase might have in proteostasis.

**Figure 3:**
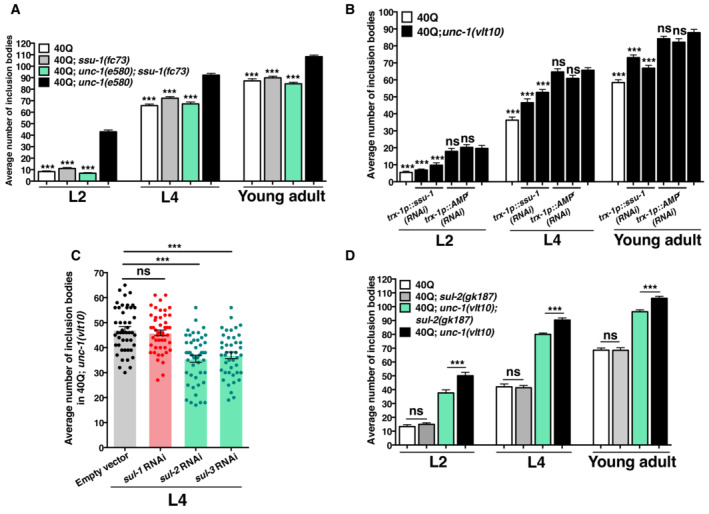
Neurohormonal signalling disruption produces an excess of signal that enhances polyQ aggregation The average number of polyQ inclusion bodies in muscle cells after *ssu‐1* and *unc‐1* suppression in 40Q::YFP animals at different stages (L2, L4 and young adults).The average number of polyQ inclusion bodies in muscle cells after RNAi was used against ssu‐1 in ASJ neurons in *unc‐1(vlt10)* worms in two independent transgenic lines.The average number of polyQ inclusion bodies in muscle cells after ubiquitous silencing of the different sulfatases of *C. elegans* (*sul‐1*, *sul‐2* and *sul‐3*) in the *unc‐1(vlt10)* background.The average number of polyQ inclusion bodies in muscle cells after disruption of *sul‐2* by the loss‐of‐function allele, *gk187*, in the *unc‐1(vlt10)* background at different stages (L2, L4 and young adult). Data information: The plotted data show the mean ± SEM. For graphs (A, B and D), 30 animals were analysed per condition and/or strain and per experiment. For graph (C), 40 animals were analysed per condition and/or strain and per experiment. Each analysis has been reproduced at least three times. ***P < 0.001; ns: not significant, where values are in reference to the 40Q; *unc‐1(vlt10)* strain (graph A and B) using the one‐way ANOVA with *post‐hoc* Tukey test for all cases. Source data are available online for this figure.

To investigate the potential role of *ssu‐1* in protein homeostasis as an interactor of *unc‐1*, we introduced the transgene expressing 40Q into worms carrying the loss‐of‐function alleles *ssu‐1(fc73)* and *unc‐1(e580)*. *ssu‐1* single‐mutant animals did not exhibit a modified aggregation phenotype (Fig 3A). In contrast, mutations in *ssu‐1* suppressed the effects caused by mutations in *unc‐1* on protein aggregation in muscle cells (Fig 3A, Appendix Fig S1C and D). This is consistent with previous evidence that *ssu‐1* mutations modified several other *unc‐1* phenotypes (Carroll *et al*, 2006). To confirm these results, we induced cell‐specific RNAi against *ssu‐1* in the ASJ neurons of *unc‐1(vlt10)* mutants using the *trx‐1* promoter. As expected, reducing the expression of *ssu‐1* in ASJ partially rescued the aggregation phenotype of *unc‐1(vlt10)* (Fig 3B). These data indicate that SSU‐1 activity in the ASJ sensory neurons is required for the disrupted protein homeostasis in muscle cells exhibited by *unc‐1* mutants.

### Arylsulfatase activity modulates the aggregation dynamics of polyQs

Our results suggest that SSU‐1 catalyses the production of a sulfonated signalling molecule in *unc‐1* mutants and that this sulfonated molecule promotes the aggregation of polyQs in muscle cells. Sulfonated molecules are more water‐soluble, and thus may more easily diffuse into organs and tissues than non‐sulfonated signals. However, to bind nuclear receptors, the sulfonated molecules need to lose their sulfonate moiety. Hence, we hypothesised that removing sulfonate from the signalling molecule may alter the aggregation phenotype. To test this, we disrupted the sulfatase enzymatic activity of the only three genes encoding enzymes with predicted sulfatase activity in *C. elegans*: *sul‐1*, *sul‐2* and *sul‐3*. *sul‐1* encodes a predicted protein similar to mammalian 6‐O‐endosulfatases (www.wormbase.org; and Appendix Fig S5), and the predicted enzymes from *sul‐2* and *sul‐3* are closely related to arylsulfatases (www.wormbase.org; and Appendix Fig S5). We fed animals with bacteria expressing dsRNA targeting each of these three genes, and a reduced function of either *sul‐2* and *sul‐3* decreased the aggregation observed in *unc‐1* animals (Fig 3C). In contrast, *sul‐1(RNAi)* animals did not have reduced inclusion bodies (Fig 3C), which suggests that only arylsulfatase function is able to modulate the aggregation of polyQs in *unc‐1* mutants. We then analysed the aggregation phenotype of the only arylsulfatase mutant available, *sul‐2(gk187)*, and observed that ablation of *sul‐2* partially reduces the aggregation phenotype of *unc‐1* animals (Fig 3D, Appendix Fig S1C and D). This agrees with our RNAi findings suggesting that arylsulfatase activity is required to modulate protein homeostasis.

### Activation of the nuclear receptor DAF‐12 reduces polyQ aggregation

**Figure 4:**
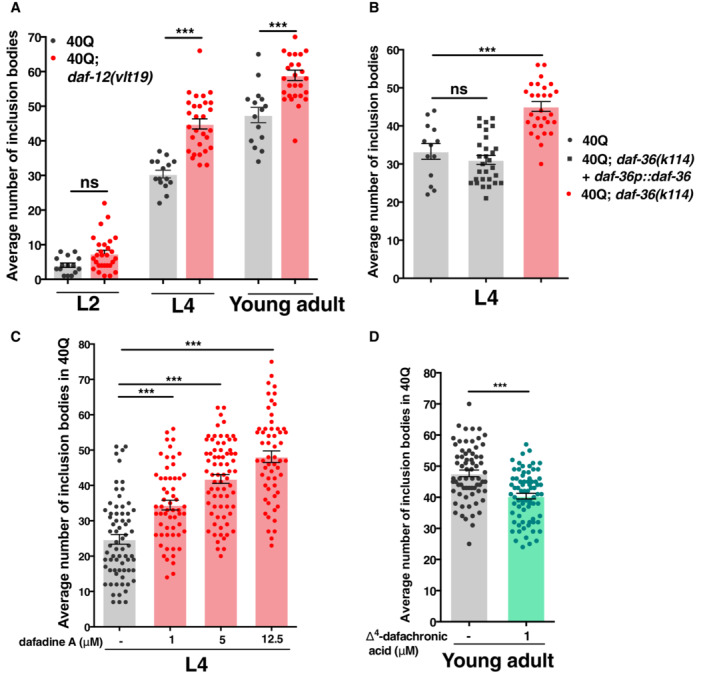
DAF‐12 signalling is required to maintain protein homeostasis The mean number of polyQ inclusion bodies in muscle cells in *daf‐12(vlt19)* mutants at different stages (L2, L4 and young adult).The average number of polyQ inclusion bodies in muscle cells with *daf‐36* suppression in 40Q::YFP compared with *daf‐36* mutants rescued with an extrachromosomal array containing the whole genomic region of the gene.The average number of polyQ inclusion bodies in muscle cells after blocking DAF‐9 via dafadine A in 40Q::YFP animals.The average number of polyQ inclusion bodies in muscle cells after treating 40Q::YFP young adult animals with 1 μM of Δ4‐dafachronic acid. Data information: We analysed the following number of worms per strain and/or condition: 30 animals for graph (A and B); more than 55 animals for graph (C); and more than 65 animals for graph (D). Each analysis has been reproduced at least three times. ***P < 0.001; ns: not significant. Statistical test was done using the one‐way ANOVA with multiple comparative test (Tukey's) (graphs A–C) or the Mann–Whitney *U*‐test (graph D). Source data are available online for this figure.

Motivated by the fact that sulfotransferases such as SSU‐1 add a sulphate moiety to steroid hormones, and there is evidence that SUL‐2 can remove sulphate from steroid hormones (Pérez‐Jiménez *et al*, 2021), we next investigated whether the signalling pathway of the dafachronic acids, targeting the DAF‐12 nuclear receptor, is activated in ASJ upon *unc‐1* disruption to modulate protein homeostasis. To investigate whether DAF‐12 modulates the phenotype of 40Q; *unc‐1* worms, we used CRISPR to generate a mutation in *daf‐12*, *vlt19* that affects the ligand‐binding domain (Appendix Fig S9B). While 40Q; *daf‐12* animals showed a substantial increase in the number of polyQ aggregates (Fig 4A), homozygous 40Q; *unc‐1*; *daf‐12* mutants were synthetically lethal and could not be analysed.

To further confirm these data, we investigated the role of dafachronic acids by disrupting two enzymes involved in their synthesis, DAF‐36 and DAF‐9 (Matyash *et al*, 2004). However, when carrying the loss of function of *daf‐36* allele *k114*, together with the insertion expressing 40Q, the worms were sterile. To avoid this issue, we rescued *daf‐36* by reintroducing a DNA construct that contained the coding and regulatory region of *daf‐36* into 40Q; *daf‐36(k114)* worms as an extrachromosomal array. We then analysed the *daf‐36* homozygous worms that lost the array, and these animals showed a substantially higher number of inclusion bodies than their siblings carrying the array (Fig 4B). To investigate the role of DAF‐9 in the aggregation of polyQs, we used different concentrations of a drug that specifically inhibits this enzyme, dafadine A (1, 5, 12.5 μM). L4 40Q animals treated with this drug showed similar polyQ‐related phenotypes as *daf‐12* mutants (Fig 4C), whereas the 40Q worms cultured on 1 μM Δ^4^‐dafachronic acid had a reduced amount of polyQ aggregates (Fig 4D). Collectively, these data suggest that signalling through DAF‐12 is required to maintain protein homeostasis.

### Activation of the nuclear receptor NHR‐1 enhances protein aggregation

It is unlikely that DAF‐12 is the target of the steroid signal from ASJ because disrupting the pathway enhances aggregation and may cause a synergy with the pathway disrupted by the loss of *unc‐1* function. Hence, we looked at NHR‐1, a nuclear receptor that regulates insulin sensitivity in *C. elegans* and that is controlled by SSU‐1 (Burton *et al*, 2018). To introduce mutant alleles of *nhr‐1* on 40Q; *unc‐1(vlt10)* worms, we used CRISPR to generate the mutation *vlt16*, which emulates *nhr‐1(n6242)*. Additionally, we isolated and characterised a new allele of *nhr‐1*, *vlt15*, which was produced by an abnormal homologous recombination and which is still likely a null allele because it causes a frameshift in the gene to produce a truncated protein (Appendix Fig S9C).

**Figure 5:**
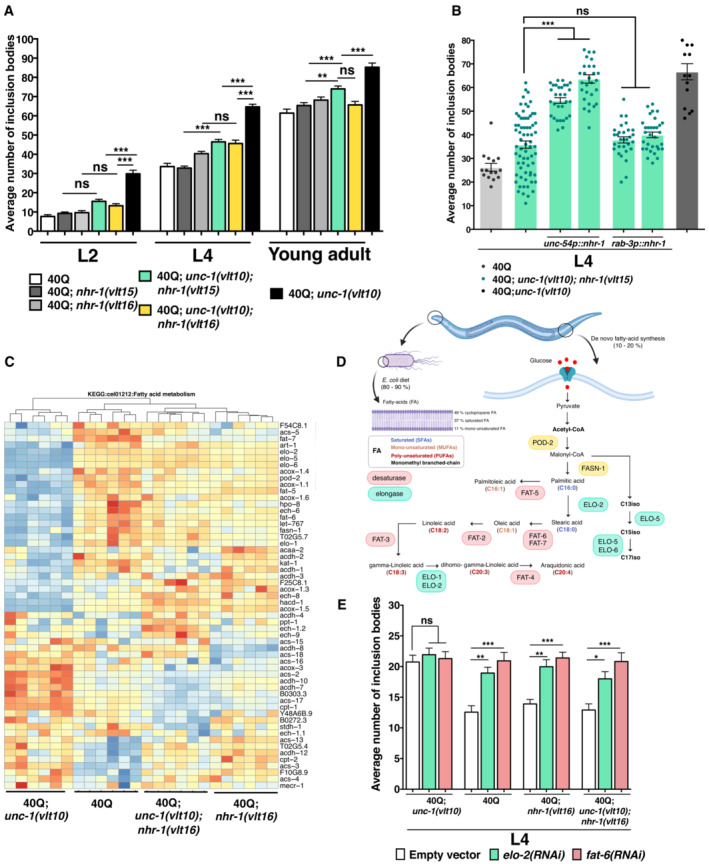
NHR‐1 modulates protein homeostasis by controlling genes of the fat metabolism The average number of polyQ inclusion bodies in *unc‐1* animals bearing *nhr‐1* loss of function alleles *vlt15* and *vlt16* in 40Q::YFP animals.The average number of polyQ inclusion bodies after reintroducing the cDNA of *nhr‐1* in muscle cells (*unc‐54p::nhr‐1*) and neurons (*rab‐3p::nhr‐1*) of the *unc‐1; nhr‐1* double mutant worms as compared to non‐rescued animals.Heatmap showing the gene expression profile related to the metabolism of fatty acids (KEGG: “Fatty Acid Metabolism”) of 40Q, 40Q; *unc‐1(vlt10)* and 40Q; *nhr‐1(vlt16)* single mutants and 40Q; *unc‐1(vlt10)*; *nhr‐1(vlt16)* double mutant worms expressing polyQs. Blue indicates genes with reduced expression levels, while red indicate increased expression in each biological sample (*N* = 6). The highlighted genes have been selected according to a *P* < 0.05 and a fold change ≥ 2.Diagram of the *de novo* fatty acid synthesis pathway highlighting the role of several genes found to be downregulated (*pod‐2*, *elo‐2*, *elo‐5*, *fat‐5*, *fat‐6*, *fat‐7*, among others) in *unc‐1* mutants.The average number of polyQ inclusion bodies in muscle cells in *unc‐1* and *nhr‐1* animals treated with RNAi against *elo‐2* and *fat‐6*. Data information: Each analysis has been reproduced at least three times (more than 30 animals). *P < 0.05; **P < 0.01; ***P < 0.001; ns: not significant, as calculated using the one‐way ANOVA with *post‐hoc* Tukey test. Source data are available online for this figure.

**Figure EV1:**
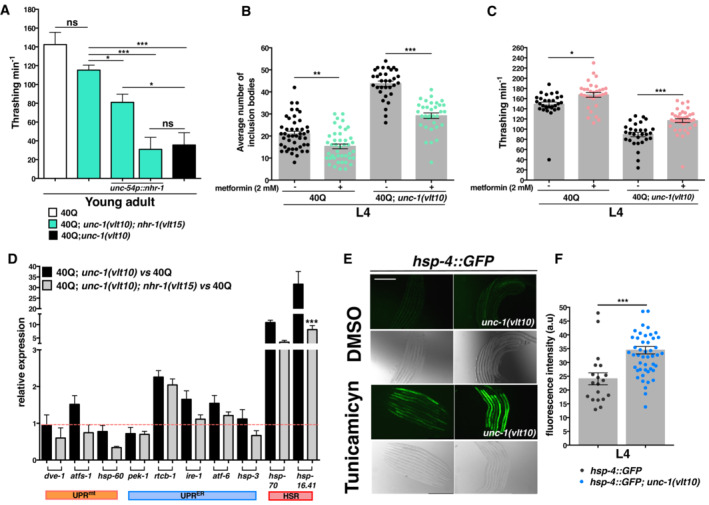
Ablating *nhr‐1* improves movement and health span and reduces *unc‐1(vlt10)*‐associated stress AMotility measurement after the muscle‐specific restoration of NHR‐1 in *unc‐1(vlt10); nhr‐1(vlt16)* young adults compared to non‐rescued double mutants.B, CThe effects of metformin treatment in *unc‐1* mutants as measured by the average number of polyQ inclusion bodies in muscle cells (B) and motility (C).DThe average expression levels of several genes related to the UPR in the endoplasmic reticulum, cytosol and mitochondria in 40Q; *unc‐1(vlt10)* and 40Q; *unc‐1(vlt10); nhr‐1(vlt15)* mutants as compared to 40Q (discontinuous red line).ERepresentative images from wild type and *unc‐1(vlt10)* animals expressing the *hsp‐4::GFP* transgene, which induces stress in the endoplasmic reticulum. The expression of this transgene is activated by a mild treatment with tunicamycin (1 μg/ml). scale bar: 250 μm.FMeasured fluorescence intensity of the animals imaged in (E). Data information: All plotted data show the mean ± standard error of the mean (SEM). At least 15 worms were analysed for thrashing assay and more than 30 animals were analysed for scoring inclusion bodies. UPR‐stress gene expression was evaluated from three biological replicates and ER‐stress reporter intensity was measured in more than 40 worms. *P < 0.05; **P < 0.01; ***P < 0.001; ns: not significant, as calculated using the one‐way ANOVA with *post‐hoc* Tukey test (graphs A–D) and the Mann–Whitney U‐test (graph F). Source data are available online for this figure.

An analysis of both mutant alleles showed that disrupting *nhr‐1* in *unc‐1* worms substantially reduced the inclusion bodies observed in *unc‐1* mutants (Fig 5A), suggesting that NHR‐1 receives the signal generated by SSU‐1 to regulate protein homeostasis. In addition, *nhr‐1* ablation also partially restored the uncoordinated phenotype of *unc‐1* worms (Fig EV1) in the same way as *ssu‐1* ablation (Carroll *et al*, 2006). Carroll *et al* (2006) showed that disruption of *ssu‐1* rescued the uncoordinated phenotype of *unc‐1* mutants. In this regard, disruption of NHR‐1 induced a similar rescue of motility in 40Q; *unc‐1*; *nhr‐1* mutants (Fig EV1A). Reintroduction of wild type *nhr‐1* in muscle cells, in the 40Q; *unc‐1*; *nhr‐1* worms, substantially reduced motility, to levels similar to the *unc‐1* mutants (Fig EV1A). Rescuing the polyQ‐induced motor defect was also associated with a reduction in polyQ aggregation (Figs 5B and EV1A). To further confirm this correlation between motor restoration and reduced polyQ aggregation, we treated 40Q and 40Q; *unc‐1(vlt10)* animals with 2 mM metformin, an anti‐diabetic drug that activates AMPK to reduce polyQ aggregation (Sanchis *et al*, 2019; Gómez‐Escribano *et al*, 2020). As expected, metformin treatment reduced polyQ aggregation and improved motor movement in 40Q and 40Q; *unc‐1(vlt10)* animals, suggesting that the motility rescue is due to decreased polyQ aggregation (Fig EV1B and C).

**Figure EV2:**
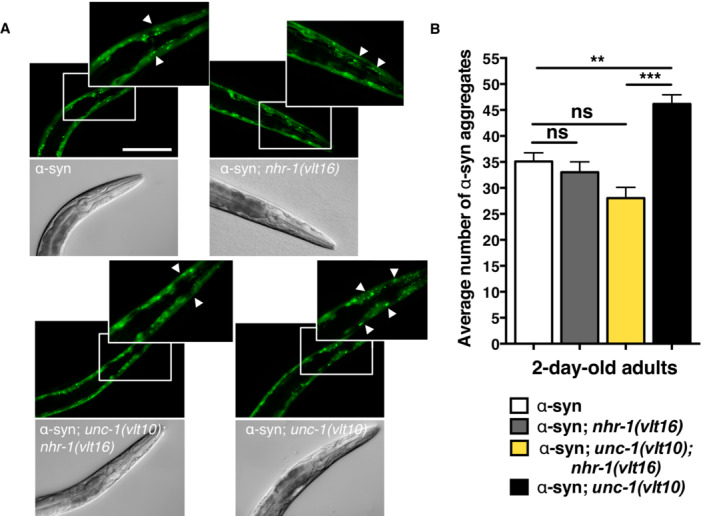
NHR‐1 modulates α‐synuclein aggregation Representative images of muscle pharyngeal cells of mutants containing *vlt10* and *vlt16* alleles in a α‐synuclein background. Magnified insets show the two pharyngeal bulbs from which the α‐synuclein aggregates (white arrows) were measured. Scale bar: 100 μm.The mean number of α‐synuclein aggregates in *unc‐1* animals bearing *nhr‐1* loss of function alleles *vlt15* and *vlt16*. Data information: The plotted data show the mean ± standard error of the mean (SEM). At least 30 animals were analysed in a three independent experiments. **P < 0.01; ***P < 0.001; ns: not significant, as calculated using the one‐way ANOVA with *post‐hoc* Tukey test. Source data are available online for this figure.

To further investigate, whether NHR‐1 is a modulator of proteostasis, or just a modulator of polyQ aggregation, we introduced both *vlt16* and *vlt10* alleles into an α‐synuclein::YFP‐expressing strain of worms. Analysis of protein aggregation, in 2‐day‐old adults, shows that ablation of *nhr‐1* did not modify the number of α‐synuclein aggregates in muscle cells (Fig EV2A and B). However, the *nhr‐1* mutation was able to rescue protein aggregation of *unc‐1* mutants (Fig EV2A and B), which suggests that *nhr‐1* modulates proteostasis imbalance induced by aggregation‐prone proteins.

One of the features of polyQ aggregation is that it induces some pathways of the UPR and the expression of genes encoding chaperones (Leitman *et al*, 2013; Shacham *et al*, 2019). Hence, we wanted to see whether these pathways were switched on in *unc‐1* animals and whether disruption of *nhr‐1* may restore them, which we did by examining the expression of key genes involved in such signalling events. If that was the case, disruption of *nhr‐1* should reduce the associated stress in *unc‐1(vlt10)* worms, and deactivate these pathways. Hence, we performed real‐time PCR for selected genes related to the UPR of the mitochondria (UPR^mt^), endoplasmic reticulum (UPR^ER^) and cytosol (HSR) (Taylor *et al*, 2014), in *unc‐1* and *nhr‐1* mutants. As expected, *unc‐1* mutants showed an increased expression of some of these genes, while double mutants *unc‐1*; *nhr‐1* showed reduced expression, suggesting that they have reduced stress (Fig EV1D). For example, the expression of *atfs‐1*, *ire‐1* and *atf‐6* was upregulated in *unc‐1* mutants, while their expression was normal in the *unc‐1*; *nhr‐1* double mutant (Fig EV1D); the same pattern occurs with the expression of the chaperone proteins *hsp‐70* and *hsp‐16.41* (Fig EV1D). To further demonstrate this, we sought to test the UPR^ER^ reporter *hsp‐4::GFP in vivo* in wild type and *unc‐1* mutants. To increase the ER‐stress signal we treated *unc‐1(vlt10)* animals with mild amounts of tunicamycin (see the Material and Methods section) and compared them with wild‐type worms. After treatment, *unc‐1(vlt10)* animals showed higher *hsp‐4::GFP* expression levels than wild type animals which further shows that *vlt10* animals have ER‐stress (Fig EV1E and F). Altogether, these results show that *nhr‐1* ablation restores a basal stress condition in *unc‐1* mutants.

### *nhr‐1* ablation rescues enhanced polyQ aggregation of *daf‐12* mutants

**Figure EV3:**
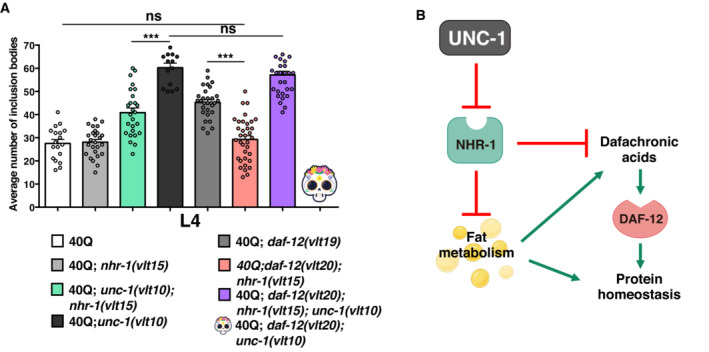
NHR‐1 and DAF‐12 antagonization regulates protein homeostasis The average number of polyQ inclusion bodies in muscle cells after *nhr‐1* ablation in *daf‐12* and *daf‐12; unc‐1* double mutants.A diagram showing the relationship between NHR‐1 and DAF‐12 in modulating protein homeostasis through fat metabolism changes and steroid hormonal signalling. The activation of NHR‐1 represses the expression of genes involved in fat metabolism and dafachronic acid synthesis. Data information: The plotted data show the mean ± standard error of the mean (SEM). At least 20 animals were analysed in a three independent experiments. ***P < 0.001; ns: not significant, as calculated using the one‐way ANOVA with *post‐hoc* Tukey test. Source data are available online for this figure.

We have shown that *nhr‐1* and *daf‐12* modulate polyQ aggregation in an opposing manner, and we wanted to next study whether they interact genetically. To do this, we first used CRISPR to produce a double mutant *nhr‐1*; *daf‐12* on a 40Q background because all three *loci* map very close to each other. The double mutants showed the same amount of polyQ aggregation as 40Q animals, indicating that *nhr‐1* is epistatic over *daf‐12* (Fig EV3A). We then introduced the *unc‐1* mutation in these animals. As above, *daf‐12* and *unc‐1* are synthetically lethal, but the loss‐of‐function allele of *nhr‐1* allowed us to isolate triple mutants *unc‐1*; *daf‐12*; *nhr‐1* expressing polyQs (Fig EV3A). Because NHR‐1 seems to inhibit hormone signalling through DAF‐12, we studied the effect of ablating *nhr‐1* in the expression of some of the genes related to the synthesis of dafachronic acids (*daf‐36* and *daf‐9*). To do this, we used the expression data from our transcriptomic analysis of the 40Q, 40Q; *unc‐1*, 40Q; *nhr‐1* and 40Q; *unc‐1*; *nhr‐1* worms (see below). This analysis showed that *daf‐36* is clearly downregulated in 40Q; *unc‐1* worms compared to 40Q animals, while *daf‐9* tended to also be downregulated but less clearly (Appendix Fig S6A and B). Regardless, the worms carrying a lesion in *nhr‐1* had increased expression of the two genes (Appendix Fig S6A and B). These data suggest that NHR‐1 regulates the synthesis of dafachronic acids, and thus the signalling of DAF‐12 (Appendix Fig S6C), and that both NHR‐1 and DAF‐12 produce opposing signals to modulate protein homeostasis (Fig EV3B).

### NHR‐1 regulates fat metabolism genes to modulate protein homeostasis

To elucidate the mechanism behind the effect of NHR‐1 function, we performed transcriptomic analysis on the following strains: 40Q, 40Q; *unc‐1(vlt10)*, 40Q; *nhr‐1(vlt16)* and 40Q; *unc‐1(vlt10)*; *nhr‐1(vlt16)*. Here, *unc‐1(vlt10)* mutants exhibited a distinct transcriptomic signature compared to 40Q, and introducing the *nhr‐1* mutation in *40Q*; *unc‐1* mutants restored the transcriptomic signature to the 40Q background (Appendix Fig S7). Among the many genes whose expression was altered in *unc‐1(vlt10)* mutants, we have identified 532 genes whose expression is rescued by the *nhr‐1(vlt16)* mutation (Appendix Fig S7). To identify cellular processes that were altered in *unc‐1* mutants, we used the WormBase tool for Gene ontology (GO) analysis (Angeles‐Albores *et al*, 2016, 2018) and KEGG pathway analysis (Kanehisa, 2000). We found that genes that were differentially expressed in *unc‐1* mutants were significantly enriched in terms related to lipid metabolism, including fatty acid metabolism (KEGG:cel01212), biosynthesis (KEGG:cel00061), degradation (KEGG:cel00071) and fat content increase (WBPhenotype:0001184), UPR response and immune response, among others.

Among the altered genes, we identified expression differences in genes encoding key enzymes of lipid metabolism (Fig 5C and D). For example, we noticed that acetyl‐CoA carboxylase (ACC) encoded by *pod‐2* and fatty acid synthase *FAS* encoded by *fasn‐1* show lower expression levels in *unc‐1* mutants compared with double mutants (Fig 5C). Both enzymes are involved in the first steps of *de novo* fatty acid synthesis. Known for catalysing carbon chain extensions of fatty acids, elongases (*elo‐1*, *elo‐2*, *elo‐5* and *elo‐6*) also had altered expression in *unc‐1* mutants compared to the 40Q worms (Fig 5C). Other types of enzymes were also downregulated in *unc‐1* mutants, like desaturases (*fat‐5*, *fat‐6* and *fat‐7*), which are involved in removing hydrogen atoms from carbon to produce double bonds (Fig 5C). In clear contrast, *nhr‐1* ablation reversed the expression patterns of these genes in *unc‐1* mutants, and their expression was closer of that of 40Q worms (Fig 5C).

To validate the transcriptomic results, we analysed the role of the elongase *elo‐2* and the desaturase *fat‐6* in terms of polyQ aggregation, using RNAi by feeding to reduce their function in 40Q and mutant backgrounds. Both genes are essential for producing oleic acid, a lipid that is neuroprotective in rodents (Song *et al*, 2019), induces lifespan extension (Fang *et al*, 2016) and prevents polyQ aggregation in worms (Goudeau *et al*, 2011; Han *et al*, 2017; Imanikia *et al*, 2019). Silencing *fat‐6* and *elo‐2* expression increased the number of inclusion bodies in all strains except 40Q; *unc‐1(vlt10)* animals (Fig 5E), where there are already high levels of inclusion bodies. These functional data further suggest that NHR‐1 controls the expression of some enzymes of the fat metabolism, which in turn are essential for maintaining protein homeostasis.

**Figure EV4:**
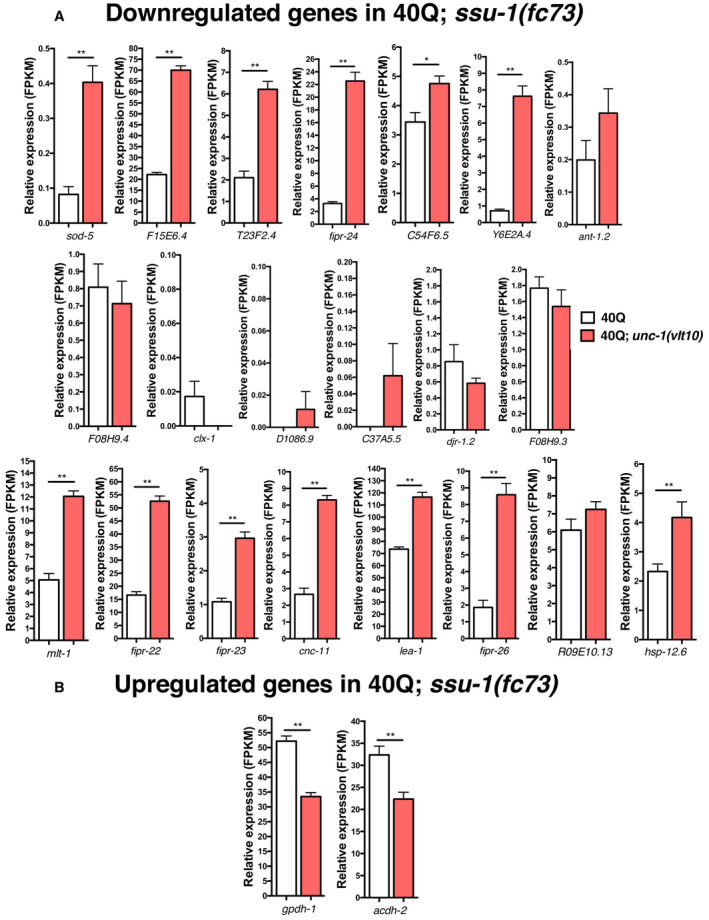
Ablation of *unc‐1* modifies downregulated and upregulated genes in *ssu‐1* Relative expression levels of genes that are not expressed in *ssu‐1(fc73)* mutants or are downregulated but are upregulated in *unc‐1(vlt10)* mutants.Genes that are upregulated in *ssu‐1(fc73)* but are downregulated in *unc‐1(vlt10)* mutants. Data information: The plotted data show the mean ± standard error of the mean (SEM). FPKM values are from the transcriptomic analysis (*N* = 6 for each mutant strain) in Fig 5. *P < 0.05; **P < 0.01; ns: not significant. The statistical analysis was done using a Mann–Whitney *U*‐test. Source data are available online for this figure.

Finally, we wanted to confirm whether *unc‐1* perturbs *ssu‐1* signalling, which impacts polyQ aggregation. For this, we took advantage of our transcriptomic data, comparing it to genes shown by Burton *et al* (2018) to be regulated by *ssu‐1*. The expression analysis showed that almost all genes have an opposite expression profile in *unc‐1* mutants (Fig EV4A and B), suggesting that *unc‐1* affects *ssu‐1* signalling and that both are connected.

### *unc‐1* animals show altered fat metabolism and ablation of *nhr‐1* restores their lipid profile

**Figure 6:**
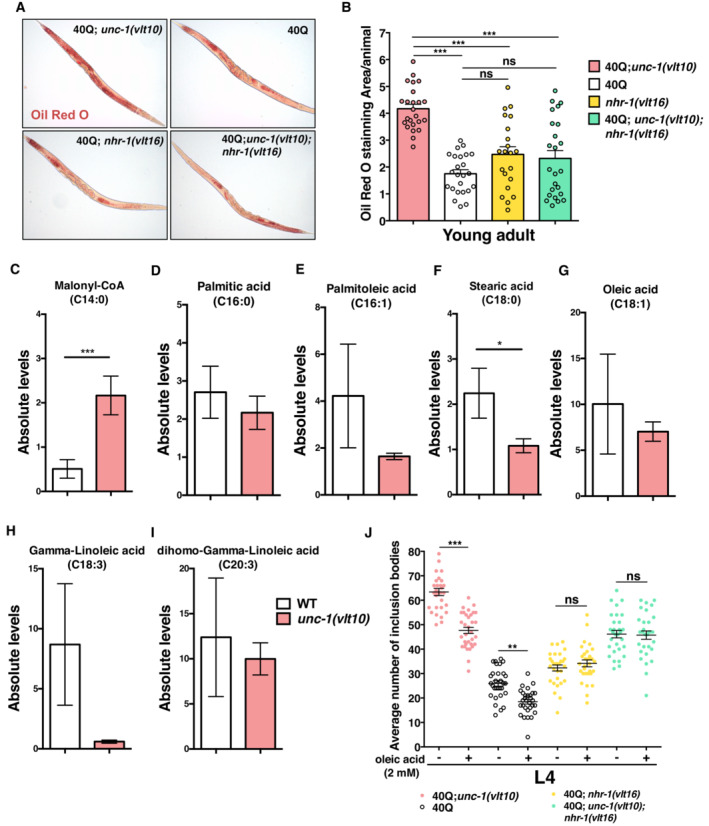
Lipid metabolism modifies polyQ aggregation ARepresentative images of simple and double young adult mutants of unc‐1 and nhr‐1 stained with Oil Red O to examine neutral lipid accumulation.BSemi‐quantitative analysis of the OilRed O staining in (A) using a plugging from ImageJ.C–IAbsolute lipid levels of saturated (manoloyl‐CoA, palmitic acid and stearic acid), monounsaturated (oleic acid) and polyunsaturated (gamma‐linoleic and dihomo‐gamma linoleic acids) fatty acids in wild type and *unc‐1(vlt10)*mutants.JThe average number of polyQ inclusion bodies in muscle cells after treatment with 2 mM of oleic acid in 40Q, 40Q;*unc‐1(vlt10)*, 40Q; *nhr‐1(vlt16)* and double mutant of both genes. Data information: The plotted data show the mean ± standard error of the mean (SEM). Each analysis has been reproduced at least three times (more than thirty animals) in A, B and J. At least twelve worm biological samples containing more than 2000 animals per sample were analysed in C‐I. *P < 0.05; **P < 0.01; ***P < 0.001; ns: not significant. The statistical analysis was done using a Mann–Whitney *U*‐test for C–I and one‐way ANOVA with *post‐hoc* Tukey test for B and J. Source data are available online for this figure.

To further investigate the altered lipid metabolism associated with *unc‐1* mutants, we analysed their lipid content using Oil Red O. This die stains neutral lipids (triglycerides, mono‐ and di‐glycerides, cholesterol esters, etc.) to provide a semi‐quantitative measure of total neutral lipids. We observed a significant enhanced staining in 40Q; *unc‐1* mutants compared to 40Q worms (Fig 6A and B). Conversely, *nhr‐1* suppression completely restored the lipid content (Fig 6A and B).

**Figure EV5:**
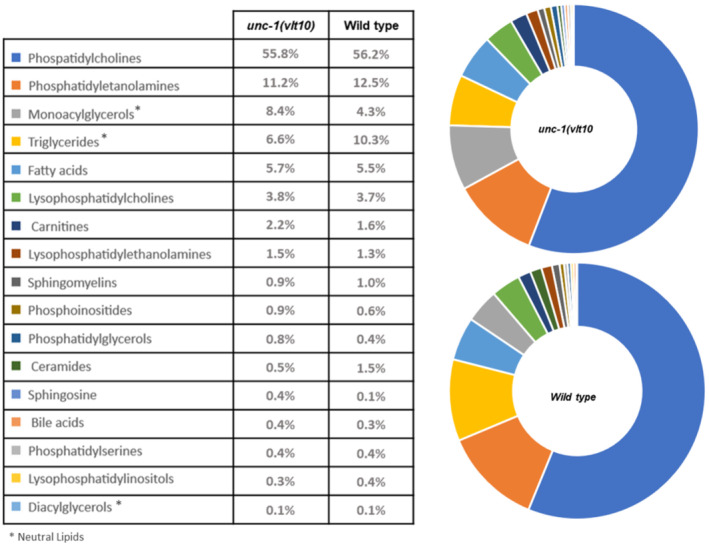
Lipid abundance in *unc‐1* mutants List of lipid classes detected in lipidomic assay in wild type and *unc‐1(vlt10)* mutants. Asterisks show neutral lipids. Data information: At least 12 worm biological samples containing more than 2000 animals per sample were analysed. Source data are available online for this figure.

Delving deeper into this apparent lipid dysregulation of *unc‐1* mutants, we performed an untargeted lipidomic assay to determine the specific fatty acid content associated with the *vlt10* allele. Though we observed more staining of neutral lipids in *unc‐1* mutants in this assay, we still observed a reduction in triglycerides (Fig EV5A). To learn which fatty acids may be remodelled, we investigated the absolute total levels of these compounds in our strains (Fig 6C–I). Stearic acid, the precursor of oleic acid, was significantly lower in mutants than in wild type worms (Fig 6F), while the levels of manolyl‐CoA increased (Fig 6C). Moreover, the levels decreased for palmitic acid, palmitoleic acid, oleic acid, gamma‐linoleic acid and dihomo‐gamma‐linoleic acid (Fig 6D, E and G–I). These results thus suggest that *vlt10* can induce the remodelling of lipid abundancy by decreasing fatty acids and, in turn, triglycerides, but may increase the amount of neutral lipids.

Among other fatty acids, *elo‐2* and *fat‐6* are involved in the *de novo* synthesis of oleic acid (Ntambi *et al*, 2002; Brock *et al*, 2007) and are protective of neurons stressed by polyQ expression (Imanikia *et al*, 2019). Hence, we tested whether this fatty acid can modulate polyQ aggregation. To do so, we treated 40Q worms with 2 mM of oleic acid and analysed the polyQ inclusion bodies. As expected, oleic acid reduced polyQ aggregation in 40Q worms (Fig 6J), but this treatment was more effective in *unc‐1* mutants compared with 40Q, which had lower expression levels of oleic acid synthesis enzymes (Fig 6J). Oleic acid treatment was ineffective in *nhr‐1(vlt16)* worms (Fig 6J), which suggests that *nhr‐1* is required for the beneficial effect of oleic acid.

## Discussion

To uncover hidden mechanisms of protein homeostasis, we isolated a gene from a forward genetic screen, *unc‐1*, that alters protein aggregation and motor coordination in *C. elegans*. We show that UNC‐1 functions non‐cell autonomously, in neurons, and disruption of the gene led to secretion of an excess of a sulphated signal that activates NHR‐1 in different tissues. Over‐activation of NHR‐1 results in downregulation of genes encoding enzymes for fat metabolism, which in turns disrupts proteostasis. In contrast, signalling through DAF‐12 functions antagonistically to NHR‐1 to control protein aggregation.

The identified gene, *unc‐1*, encodes a *C. elegans* stomatin‐like protein that regulates protein homeostasis in muscle cells and neurons, acting in IL2 neurons (Figs 1 and 2). Our data also shows that the effect on aggregation of polyQs is specific to certain stomatin‐like proteins (Appendix Table S2). Aggregation can similarly be enhanced by the loss of function of several innexin genes, which suggests the involvement of gap junctions—the pore components of the electrical synapse—at least in invertebrates (see review Dahl & Muller, 2014). This suggests that electrical synapses are essential for maintaining appropriate proteostasis in *C. elegans*. If the ASJ‐IL2 gap junctions served to simply shunt current, as we had expected, the loss of function of *unc‐1* would result in a general increase in ASJ excitability (Appendix Fig S4), though this was not the case. Rather, the differential effect on different ASJ functions suggests that these innexins play more complex roles, which might be specific to different aspects of ASJ function. However, innexins can also operate as hemichannels (Bouhours *et al*, 2011; Hervé & Derangeon, 2013), which may explain this specificity. We believe that these data open many questions, regarding the influence of the electrical synapses, or their constituent subunits, over the control of metabolism, that are worth pursuing in the future. What is sensed by ASJ and/or IL2, and why these upstream signals regulate, is unknown to us, and it will be very interesting to study them in the future.

Our results also suggest that disrupting the communication between some neurons triggers the secretion of excess sulphated hormone, from ASJ, in a process dependent on SSU‐1 (Fig 3A). An important remaining question is the nature of the sulphated signal released by ASJ. We have used multiple metabolomic approaches to try to identify this putative hormone, but this may require technological advances currently outside our reach. Whatever the nature of the signalling coming out of the neuron, our data shows that altering the function of *unc‐1* induces changes in genes regulated by *ssu‐1*, which shows that the flow of information from *unc‐1* downstream, involves *ssu‐1* (Fig EV4A and B).

For this hormonal signal to induce an enhanced polyQ aggregation on muscle cells and neurons, it requires functioning arylsulfatases SUL‐2 and SUL‐3 (Fig 3C). These proteins are arylsulfatases, evolutionarily closer to human steroid sulfatases, which suggests that the sulphur groups of this unknown hormone need to be removed for receptor binding.

From our epistasis analysis, we saw that the steroid hormone produced by ASJ seems to be controlled by NHR‐1 and that this nuclear receptor regulates genes related to lipid metabolism, which in turn affects proteostasis in *C. elegans*. Our transcriptomic analysis of single and double *unc‐1* and *nhr‐1* mutants indicated that many genes involved in fatty acid synthesis are altered in *unc‐1* worms and that introducing *nhr‐1* loss‐of‐function alleles restores their expression to close to that of 40Q worms (Fig 5C and E). We validated this role of fat metabolism by reducing the function of *elo‐2* and *fat‐6*, whose silencing increases polyQ aggregation (Fig 5E). Because FAT‐6, together with FAT‐7, converts stearic acid into oleic acid, a protective lipid that promotes longevity and improve neuronal proteostasis (Goudeau *et al*, 2011; Imanikia *et al*, 2019), we examined oleic acid synthesis in these worms. We showed that the enzymes involved in oleic acid synthesis are downregulated in *unc‐1* mutants and that oleic acid is effective in reducing polyQ aggregation, suggesting reduced oleic acid synthesis in these animals. However, this lipid had no effect over *nhr‐1* mutants (Fig 6J), suggesting that oleic acid may depend on functional NHR‐1 to produce the beneficial effects on proteostasis. While oleic acid production is controlled by NHR‐1, this result is still not surprising given that some nuclear receptors are controlled by feedback from the products of the enzymes they regulate (Bagamasbad & Denver, 2011). In support of both this and the transcriptomics analysis, an untargeted lipidomic assay showed that the *unc‐1* mutants tended to produce lower amounts of certain free‐fatty acids. Altogether, these results suggest that an altered neurohormonal signalling dysregulates fat metabolism, which in turns affects protein homeostasis.

Our data also strongly suggest that different steroid hormones and different signalling pathways exert opposing effects on protein homeostasis. In contrast with NHR‐1 disruptions, disrupting the nuclear receptor DAF‐12 (i.e., mutating *daf‐12* or culturing worms on dafadine A, a well‐known *daf‐9* inhibitor) enhanced polyQ aggregation; adding dafachronic acids produced a protective effect (Fig 4) (Martin *et al*, 2008, 2009). In agreement with our findings, Farina *et al* (2017) showed that the neurosteroids that activate DAF‐12 are neuroprotective in *C. elegans* models of polyQ‐induced toxicity. Moreover, other authors showed that *daf‐12* signalling promotes protein homeostasis by modulating the function of the ER stress response (Mark *et al*, 2016). In addition, *nhr‐1* is epistatic over *daf‐12*, suggesting that the two receptors compete to modulate protein homeostasis (Fig EV3, Appendix Fig S6).

To our knowledge, this is the first report showing that different steroid hormone signals have opposing modulator effects on protein homeostasis in animals, and it will be of interest to investigate if this is evolutionarily conserved between invertebrates and mammals. There is also evidence that neurosteroids may protect against neurodegenerative diseases that involve the dysregulation of protein homeostasis, such as for Alzheimer's, Parkinson's or HD (see review: Borowicz *et al*, 2011). These points are especially important for the development of drugs against inflammatory and metabolic and neurodegenerative diseases, as the wide range of metabolic processes regulated by nuclear receptors makes them outstanding druggable targets (see reviews: Schulman & Heyman, 2004; Saijo *et al*, 2010; Schulman, 2010; Skerrett *et al*, 2014; Moutinho *et al*, 2019). Inhibitors for many of the proteins in these signalling pathways have also been developed to treat different kinds of cancer and metabolic diseases, such as against sulfotransferases (Chapman *et al*, 2002; Rath *et al*, 2004), the cytochrome *daf‐9*/CYP2J2 (Chen *et al*, 2009), sulfatases (Mostafa & Taylor, 2013; Yue *et al*, 2016; Pérez‐Jiménez *et al*, 2021) and modulators of NHRs (Chen, 2008; Pinne & Raucy, 2014; Davis *et al*, 2018; Hong *et al*, 2018; Kang *et al*, 2018). Additionally, some attempts have been made to use fatty acid‐enriched diets to treat neurodegeneration, although the results were not very promising (see reviews: Shamim *et al*, 2018; Bono‐Yagüe *et al*, 2020). We expect that new approaches will focus on applying modulators of key enzymes of the lipid metabolism to modify the amount of protective lipids within the stressed tissue.

Overall, this work has provided the first evidence that nuclear receptors modulate proteostasis through the remodelling of lipid metabolism. Our findings transfer the attention to lipids to find potential pharmacological targets for treating pathological conditions produced by toxic mutant proteins that induce neurodegeneration.

## Materials and Methods

### Maintenance and culture of *C. elegans*

All strains were maintained under standard conditions (Brenner, 1974). Some strains used in this work were obtained from Caenorhabditis Genetics Center (CGC; Minneapolis, MN, USA). The CGC is funded by the National Institutes of Health's National Center for Research Resources (NGRRs). CW911 strain: *ssu‐1(fc73) V*; *unc‐1(e580) X* was kindly provided by Phillip G. Morgan (University of Washington, Seattle, USA). AX5828: dbEx804[*trx‐1p::YC3.60*, *unc‐122p::GFP*] was kindly provided by Mario de Bono. Appendix Table S4 provides a detailed list of strains used in this study. All strains were outcrossed at least three times and cultured at 20°C.

### Genetic manipulation of *C. elegans*

To generate vectors for gene expression in *C. elegans*, we took advantage of Gateway^®^ MultiSite Pro system (Invitrogen, Waltham, MA, USA). Primer sequences for cloning are listed at Appendix Table S5. Briefly, we amplified 1.7 kb from promoter region upstream of *rab‐3* gene using specific primers wild type gDNA. We cloned the attB1‐B2‐flanked PCR product into pDONR™ 221‐P1P5r to generate pDONR 221‐P1P5r‐*rab‐3p* (pAPG1). We amplified the cDNA of *unc‐1* to clone it into pDONR 221‐P5P2‐*unc‐1* (pAPG2). We used pDONR 221‐P1P5r‐*myo‐3p* kindly provided by Robyn Branicky. Plasmids containing each promoter region, were recombined with the cDNA of *unc‐1*, within a destination vector that contains the transcription terminator from the gene *unc‐54*, pHP2 (Walker *et al*, 2009), to generate the following destination constructs: pDEST HP2‐*rab‐3p::unc‐1::unc‐54t* (pAPG3) and pDEST HP2‐*myo‐3p::unc‐1::unc‐54t* (pAPG4). To induce *nhr‐1* expression in muscle cells and neurons we used pVD105 and pVD106, respectively (Burton *et al*, 2018).

To produce a dominant negative form of *unc‐1*, the *n494* allele, we introduced the mutation into a cDNA of *unc‐1* containing in pAPG2 to obtain pDONR 221 P5P2‐ *unc‐1(n494)* (pAPG5). Next, we used pAPG1 and pAPG5 as donor vectors and pHP2 as destination vector, to generate pAPG6 (*rab‐3p::unc‐1(n494)::unc‐54t*). To rescue *unc‐1* in IL2 neurons we obtained fusion PCR products using *osm‐3p* and *oig‐1p* (see more detailed Appendix Fig S8). DNA mixtures for injection consisted of 25–50 ng/μl of the interest DNA [except for *myo‐3p* or *unc‐54p* expression analysis (1–5 ng/μl)], a DNA marker (pCFJ90; *myo‐2p*::mCherry) at 2.5–5 ng/μl, together with the empty yeast plasmid pYES (Thermofisher, Waltham, MA, USA) as carrier DNA, at a total final concentration of 120 ng/μl.

### Cell‐specific RNA interference

To induce tissue‐specific RNAi we developed PCR fusion products using a promoter region to sense and antisense fragments of the interest gene (Appendix Fig S8). Therefore, simultaneous expression of these DNA constructs in a specific tissue, such as nervous system (which are refractory to RNAi), induce a double complementary strand RNA synthesis to activate RNAi mechanism in neurons. We amplified promoter regions (of *rab‐3*, *trx‐1*, *oig‐1*, *osm‐3*, *glr‐1*, *gpa‐9*) to allow us to express dsRNA in a tissue‐specific manner (nervous system, ASJ, IL2, PVQ neurons, respectively) (Appendix Fig S8, Appendix Table S5). In contrast with ASJ, there are not promoters, to our knowledge, that drive the expression of genes exclusively in IL2L and/or PVQR neurons (Chelur & Chalfie, 2007). We therefore used pairs of promoters, whose expression overlapped only in our neurons of interest, to drive expression of the two RNA strands, such that expression of dsRNA was restricted to IL2 or PVQ. In parallel, we amplified sense/antisense fragment from our genes of interest (*unc‐1*, *unc‐7*, *inx‐2*, *inx‐6* and *ssu‐1*), adding a sequence complementary to the specific promoter that we wanted to fuse with it (Appendix Fig S8, Appendix Table S5). Both PCR products were combined in a single PCR reaction to amplify fused PCR products, using appropriate external primers complementary to the promoter and gene (Appendix Fig S8, Appendix Table S5). We used a DNA fragment from the bacterial β‐Lactamase gene (which we named AMP^r^) as negative control in the tissue‐specific RNAi experiments. We injected 2.5 ng/μl of each construct (sense/antisense) together with pCFJ90 (encodes a pharynx‐expressed mCherry as a transgenic marker) as a reporter, and pYES (Thermofisher) as carrier DNA until 120 ng/μl.

### RNA interference by feeding

To induce ubiquitous and constitutive RNAi, we feed the animals with modified HT115 *Escherichia coli* strain expressing dsRNA of each gene. We generated RNAi vectors for these genes cloning a coding fragment that covers all isoforms of each gene, into an EcoRV site of pL4440 vector. The PCR product for each gene was amplified using Phusion polymerase (Thermofisher) and specific primers (see detailed sequence in Appendix Table S5). HT115 strains containing empty pL4440, as a control, and RNAi vectors (pAPG11‐*sul‐1*, pAPG12‐*sul‐2*, pAPG13‐*sul‐3*, pAPG52‐*elo‐2* and pAPG53‐*fat‐6*) were grown in liquid Luria‐Bertani medium containing 50 μg/ml carbenicillin over night at 37°C. Next, bacteria culture was induced by IPTG for 2 h at 37°C, before seeding RNAi plates. After drying plates, synchronised animals (L1 or L3; depending of the assay) were feed with HT115 bacteria containing empty vector or RNAi vectors until reaching L4 stage to be scored.

### Construction of knock‐in worms using CRISPR

We used CRISPR/Cas9 system to introduce point mutations into *nhr‐1* and deletions into *inx‐2* and *daf‐12* sequences. We based on our design and strategy from Julián Ceron Lab (IDIBELL Institute, Barcelona, Spain) (Vicencio *et al*, 2019). To introduce deletions into *inx‐2* and *daf‐12* genes, we used two crRNAs for each one, which are complementary to distal exon regions. We generated a full deletion of *inx‐2*, *vlt22*, using the following gRNAs: crRNA#1 5′ – ACCGGAGCTCTCCCACCAAA – 3′ and crRNA#2 5′ – GCACAATGAGAAACCAGTAT – 3′. We used the ssODN sequence to isolate mutant strains easier: 5′ – ACGTAGCGTTT GCGTGCGCACACCTCGCGTAGTGGTCCGCGTTCGCATTACTTGCGCTGG**GGAAA**CATACTGTACTGATCGATCAAGAGTTTTCACTGTCTTCTCGTCCATCACCAGCCATATTCATAATTTCTTTCAAT – 3′ (neutral nucleotides are marked in bold and it is useful to genotyping mutants) (Appendix Fig S9A). For *daf‐12*, we generated *vlt19* allele using the following crRNAs: crRNA#1 5′ – ATATTATGGATGTTACCATG – 3′ and gRNA#2 5′ – GGAATCGTTGTTCGGAGAGC – 3′. *vlt19* encodes a deletion of 500 pb inside of ligand binding domain (Appendix Fig S9B). We targeted *nhr‐1* to emulate *n6242* allele by the following crRNA: 5′ – CACCACTCCACACCGTCTTC – 3′. The ssODN sequence to induce knock‐in to generate the allele of interest was: 5′ – CCAACGAAGAAAATCAAGATGAGCAGCGGATCTGACGACGAGCAAGCCACCACTCCACAC**A**G**A**CT**C** *T*A**A**GACCAGGTCAGTGGGCGAAACACATTTACCCCAATTTGGATGCATCTTGAATTTCAACAA – 3′ to generate *vlt16* in *nhr‐1* (in bold are marked neutral changes and in italics are denoted the point‐nonsense mutation (C/T) (Appendix Fig S9C)). We isolated a new allele, *vlt15*, by an abnormal random recombination that encodes a premature stop codon later than *vlt16* (Appendix Fig S9C). To produce the mixture for injection, the crRNAs were suspended in 20 μl of IDTE nuclease free buffer to obtain 100 μM stock. ssODNs were suspended in the same buffer at 1 μg/μl. CRISPR components were added at the following final concentrations: Cas9: 4.5 μM; ALT‐R trackRNA: 32 μM; target gene crRNA: 35 μM; ssODN target gene: 175 ng/μl. For targeting deletions, we used final concentrations 17.5 μM for each crRNAs. We used disruption of *dyp‐10* gene as a selection marker.

### Quantitative PCR

The relative expression of genes was measured by RT–qPCR using the ViiA7 thermal cycler from Applied Biosystems (Waltham, MA, USA) using Taqman™ probes. To verify the expression of genes included within extrachromosomal arrays, we selected by hand worms expressing reporter (*myo‐2p::mCherry*) for RNA extraction (OMEGA Bio‐Tek, Norcross, GA, USA) using the M165FC dissecting microscope (Leica, Wetzlar, Germany). To evaluate expression of genes in mutants, we collected a synchronised population of young adult worms in RNA lysis buffer. Extracts were frozen at −80°C before RNA extraction. RNA samples were treated by DNAase treatment (Qiagen, Hilden, Germany). cDNA synthesis was done using 0.1–1 μg approximately of RNA following the manufacturer protocol (Takara Bio, Kusatsu, Japan). We used the 2xPrimeTime^®^ Gene Expression Master Mix (Takara Bio), and the PCR program was as follows: 1 cycle of 10 min at 50°C; 1 cycle of denaturalization at 95°C 15 s; 40 cycles of polymerisation at 60°C 1 min. To normalise relative expression, we used the housekeeping control, *pmp‐3* (Taqman probe from Applied biosystems, Ce02485188_m1). To measure expression of interest genes or transgenes we designed customised probes from IDTDNA. We considered all isoforms for each gene in the customised design. We included at least three technical replicates for each measuring.

### Western blot

We grew synchronised L1 worms until young adult stage to isolate total protein extract using RIPA buffer. The BCA kit was used to quantify total protein and after this, we added 4× SDS sample loading buffer and preserved the samples at −80°C. Total protein extracts were separated by 12% sodium dodecyl sulphated‐polyacrylamide gel electrophoresis (SDS‐PAGE) and transferred to polyvinylidene di‐fluoride (PVDF) membranes (Bio‐Rad Laboratories, Hercules, CA, USA) by semi‐dry blotting (Trans‐Blot Turbo, Bio‐Rad). Membrane blocking was done with 5% non‐fat milk, according to the specification of the following primary antibodies: mouse anti‐polyQ (1:1,000, Sigma Ref. #P1874) and mouse anti‐actin (1:500, Santa Cruz Biotechnologies ref. #sc‐47778) to normalise the results. PVDF membranes were stripped after detecting actin. Primary antibody incubation was done at 4°C and next we incubated the second antibody anti‐mouse conjugated to HRP at room temperature (1:10,000, Abcam, ref. #ab97023). PVDF membranes were stripped after detecting actin and next, polyQ was detected in the same membrane. Images were obtained using NZY Advanced ECL (Nzytech, ref. #MB40201). Quantification was done using the ImageJ software.

### Analysis of the effect of ∆^4^‐dafachronic acid and dafadine A on worms

Briefly, we grown synchronised L1 animals on Dafachronic acid 1 μM (DA dissolved in ethanol) (Martin *et al*, 2008, 2009) in M9 buffer containing 5 μg/ml cholesterol, 12.5 μg/ml nystatin, 50 μg/ml streptomycin and OP50 *E. coli* at 0.5 Optic density. Dafadine A (Sigma‐Aldrich‐Merck, St. Louis, MO, USA) was added at three dose (1, 5, 12.5 μM) Animals were growth in liquid medium at 60°C and moderate shaking until young adults. We evaluated the effect of both compounds at least three times in independent experiments over polyQ aggregation pattern.

### X‐34 and Oil Red O staining

X‐34 dye (Sigma‐Aldrich‐Merck) stains specifically amyloid deposits. One‐day synchronised adults were incubated in a drop containing 1 mM X‐34 and 10 mM TRIS pH 7.5 solution. Stained animals were washed with PBS‐Tween and transferred to fresh NGM plates. We used the Oil Red O (Sigma‐Aldrich‐Merck) dye to stain triglycerides and lipoproteins for semi‐quantitative purposes. Young adults were stained with diluted 3:2 Oil Red O stock solution for 2 h at room temperature in a rotator. After this, animals were washed two times with PBS‐Tween (0.1%) for 30 min. Stained animals were mounted in 2% agarose pads with sodium azide (0.05 M) to count amyloid deposits using a DM2500 fluorescent microscope (Leica). To quantify the stained area of worms, with the Oild Red O stain, we used a DMD108 microscope (Leica). We analysed the pictures obtained from these animals using the Image J software.

### Oleic acid treatment of worms

We generated NGM plates containing 2 mM oleic acid, which was added to molten NGM medium. Plates were conserved in the dark and seeded with OP50 before use it. Synchronised L1 animals were grown until they reached the L4 stage to score polyQ inclusion bodies.

### Quantification of polyQ inclusion bodies in muscle cells and neurons

The expression of the *40Q::YFP* transgene produces body inclusion formation in muscle cells in an age‐dependent manner. The average number of inclusion bodies were obtained by counting total number of polyQ::YFP inclusion bodies in muscle cells per animal *in vivo* using a dissecting microscope equipped with fluorescence (M165FC, Leica). PolyQ aggregation happens in an age‐dependent manner, so we analysed aggregation in specific developmental stages (L2, L4 and young adult). Animals were analysed from a heterogeneous population and, after scoring, they were removed to avoid undesired duplicates. Scoring was from at least three independent experiments, in which we counted at least, 10 animals per genotype and stage. Thirty animals or more were analysed for each genotype and stage in total.

To score neuronal polyQ aggregates we used a model that expresses 40 glutamines under the control of the promoter of the *F25B3.3* gene. Neuronal aggregation is more complex to follow, in whole animals, so we selected the Ventral Nerve Cord only to count inclusion bodies. Sixty young adults were analysed for each genotype in total, from three independent experiments. The number of neuronal polyQ aggregates was scored using the DM2500 Leica vertical microscope, equipped with fluorescence.

### Scoring of number of α‐synuclein::YFP aggregates in muscle cells

The *unc‐54p::α‐synuclein::YFP* transgene induces late aggregation, in contrast with polyQ aggregation. Moreover, unlike 40Q::YFP inclusion bodies, which are visible using a dissecting microscope, α‐synuclein::YFP aggregates are only detectable using upright microscopes (like, the DM2500, Leica). Since it is not feasible to perform a complete count of whole muscle tissue, we selected the area located between the positions of the two pharyngeal bulbs, to evaluate the aggregation pattern of α‐synuclein. Sixty 2‐day‐old adults were analysed for each genotype in total, from three independent experiments.

### Scoring of number of beta‐amyloid X‐34‐stained deposits in muscle cells

To evaluate β‐amyloid protein aggregation, we used a *C. elegans* model that expresses the human Aβ peptide constitutively in muscle cells. To detect β‐amyloid deposits, we stained 1‐day‐old adults with X‐34 dye (Sigma‐Aldrich‐Merk). Stained 2‐day‐old animals were analysed using an upright microscope equipped with fluorescence (DM2500, Leica). We selected the area between the positions of the two pharyngeal bulbs to evaluate the number of β‐amyloid deposits. Sixty 2‐day‐old adult animals were analysed for each genotype in total, from three independent experiments.

### Motility thrashing assay

We evaluated motility capacity of young adults by thrashing. This assay consists of scoring the number of thrashes that show an animal when swimming in M9 buffer. We have considered a thrash when animal moves simultaneous head and tail. Animals were acclimated for 30 s before scoring number of thrashes for the following 30 s. Each animal was collected independently in a well. The average number of thrashes was extrapolated for 1 min to show the mean of the values. At least 15–20 animals were analysed for each genotype and transgenic line in three independent experiments at 20°C. Animals damaged during handling were discarded in this study.

### Calcium imaging

Calcium imaging in ASJ was carried out as described previously (Kerr *et al*, 2000; Walker & Schafer, 2020). The ratiometric calcium indicator YC3.60 (Cameleon) was expressed in ASJ using the *trx‐1* promoter (Fenk & de Bono, 2015). Briefly, animals were glued onto 2% agarose pads and temperature stimuli were administered using perfusion of CTX buffer (25 mM KPO_4_ pH6, 1 mM CaCl_2_, 1 mM MgSO_4_). Images were recorded at 5 Hz using a Dualview beam splitter (Optical Insights) to simultaneously record two wavelengths and an iXon EM camera with IQ1.9 capture software (Andor Technology). Fluorescence was quantified and analysed in Matlab (Mathworks) using SpikeFinder and NeuronTracker, custom analysis scripts written by Rabinowitch *et al* (2013).

### Transcriptomic analysis

Synchronised L1 (40Q, 40Q; *unc‐1(vlt10)*, 40Q; *nhr‐1(vlt16)* and 40Q; *unc‐1(vlt10); nhr‐1(vlt16)*) were grown in NGM plates at 20°C until they reached young adult stage. They were subsequently collected in M9 buffer, frozen, thawed and mechanically lysed with 200 mg of glass beads (Sigma‐Aldrich‐Merck) for 30 s using a Fastprep shaker apparatus (Thermofisher Scientific, FP120 model). RNA was extracted using NZY total RNA isolation kit, Nzytech. Supernatant was recovered from lysate samples by centrifuging extracts at maximum speed for 1 min. The purified RNA was treated with DNAse, quantified and sent for sequencing by Novogene (Cambridge, UK). Six biological replicates, consisting of pooled bulk nematode RNA were sequenced for each genotype. Briefly, after quality control, mRNA was enriched using oligo(dT) beads and randomly fragmented. cDNA was synthesised using random hexamers and reverse transcriptase. After first‐strand synthesis, a custom second‐strand synthesis buffer (Illumina) was added with dNTPs, RNase H and *E. coli* polymerase I in order to generate the second strand by nick‐translation. To prepare the final cDNA library, a round of purification, terminal repair, A‐tailing, ligation of sequencing adapters, size selection and PCR enrichment were performed. Library concentration was first quantified using a Qubit 2.0 fluorometer (Life Technologies). Insert size was checked on an Agilent 2100 and quantified using quantitative PCR (Q‐PCR).

Reads were aligned to the *C. elegans* genome assembly WBCel235 using HISAT2 (Kim *et al*, 2015) for alignment, HTSeq (Anders *et al*, 2015) for gene expression quantification and DESeq2 (Love *et al*, 2014) for differential expression analysis. Genes with a fold change greater than 2 and a corrected *P*‐value lower than 0.05 were retained. EnhancedVolcano (Blighe *et al*, 2022) was used to plot fold change versus *P*‐values. Pathway enrichment analysis was performed using KEGG tool (www.genome.jp/kegg/) (Kanehisa, 2000). Heatmaps were performed in R (R Core Team, 2017) using the pheatmap package (Kolde, 2019). Read counts were centred and scaled for each gene to have mean zero and standard deviation one across the row.

### Untargeted lipidomics assay

Young adults synchronised wild type and *unc‐1(vlt10)* animals were collected in RIPA 1× buffer (Sigma‐Aldrich‐Merck) to obtain protein extract. We collected six biological samples per genotype in each independent experiment (*n* = 3 experiments and *n* = 10 cultures of worms per genotype). Total protein extract was quantified using the commercial kit (Pierce™ BCA Protein Assay kit #23225, Thermofisher) to normalise the lipid abundance of the samples. Samples were analysed using a liquid chromatography equipment coupled to a high‐resolution mass spectrometer with an orbitrap detector (UPLC‐Q‐Exactive Plus) and an electrospray source (ESI) following the procedures optimised in the Analytical Unit (internal method not published). Samples and quality controls were randomly injected into the chromatographic system to avoid variability within the analytical sequence, as well as to improve the quality and reproducibility of the study. Data were acquired in in Full MS, DIA (data independent analysis) and DDA (data dependent analysis) scan modes and processed using an in‐house script in the R software (v.3.6.1) with the XCMS and CAMERA packages for detection, filtering and alignment of spikes. The LipidMS library was used to identify candidate lipids (Alcoriza‐Balaguer *et al*, 2019).

### Imaging worms by microscopy

Fluorescence images were acquired using an SP5 confocal microscope and DM2500 vertical fluorescent microscope (Leica). Oil Red O images to analyse stained area per animal were taken with a digital dissecting microscope DMD108 (Leica). In all cases, animals were mounted on agarose pads (2% on water) containing one drop of sodium acid (0.05 M) to anaesthetise them. Nematode selection and manipulation were performed using dissecting microscopes without or with fluorescence (MS5 and M165FC, Leica).

### Statistics

Statistical analysis was performed using an analysis of variance test (one‐way ANOVA) combined with a Tukey test to perform multiple comparisons of different strains and/or conditions. To perform comparisons between two conditions we used a Mann Whitney *t*‐test to obtain the statistical significance of the data. In the graphs, we show the mean ± standard error (SEM). SEM is displayed as a bar, while asterisks show significant of the data (*P*‐value) (*P < 0.05; **P < 0.01; ***P < 0.001). The abbreviation “ns” means not significant.

## Author contributions

**Rafael P Vázquez‐Manrique:** Conceptualization; data curation; supervision; funding acquisition; investigation; visualization; methodology; writing – original draft; project administration; writing – review and editing. **Ana P Gómez‐Escribano:** Conceptualization; data curation; formal analysis; validation; investigation; visualization; methodology; writing – original draft; writing – review and editing. **Carlos Mora‐Martínez:** Conceptualization; resources; software; formal analysis; methodology; writing – review and editing. **Marta Roca:** Software; formal analysis; investigation; methodology; writing – review and editing. **Denise S Walker:** Formal analysis; investigation; methodology; writing – review and editing. **Joaquín Panadero:** Data curation; software; formal analysis; investigation. **Maria D Sequedo:** Investigation; methodology. **Ratni Saini:** Investigation. **Hans‐Joachim Knölker:** Investigation. **Jose Blanca:** Investigation. **Juan Burguera:** Conceptualization; investigation. **Agustin Lahoz:** Conceptualization; validation; investigation; writing – review and editing. **Joaquin Cañizares:** Investigation. **José M Millán:** Conceptualization; investigation; writing – review and editing. **Nick O Burton:** Conceptualization; investigation; writing – original draft; writing – review and editing. **William R Schafer:** Investigation; writing – review and editing.

## Disclosure and competing interests statement

The authors declare that they have no conflict of interest.

## Supporting information



Appendix

Expanded View Figures PDF

Source Data for Expanded View and Appendix

PDF+

Source Data for Figure 1

Source Data for Figure 2

Source Data for Figure 3

Source Data for Figure 4

Source Data for Figure 5

Source Data for Figure 6

## Data Availability

The transcriptomic data from this publication have been deposited to the Gene Expression Omnibus (https://www.ncbi.nlm.nih.gov/geo/) with the accession number GSE220662 (https://www.ncbi.nlm.nih.gov/geo/query/acc.cgi?acc=GSE220662).
