# Supplementary material for: Changes in lipid metabolism driven by steroid signalling modulate proteostasis in C. elegans
Source: EMBO Rep. 2023 Apr 27;24(6):e55556. doi: 10.15252/embr.202255556 (PMC10240203; doi:10.15252/embr.202255556)
Supplement: Supplementary file 1 — Appendix [file EMBR-24-e55556-s006.pdf]

Appendix

Changes in lipid metabolism driven by steroid signalling modulate proteostasis in *C. elegans*

Gómez-Escribano AP, Mora-Martínez C, Roca M, Walker DS, Panadero J, Sequedo MD, Saini R, Knölker HJ, Blanca J, Burguera J, Lahoz A, Cañizares J, Millán JM, Burton NO, Schafer WR, Vázquez-Manrique RP*

*Corresponding author. Email: rafael_vazquez@iislafe.es

Appendix – Table of content

- Isolation of the *unc-1(vlt10)* mutation ……………………………………………………2
- Appendix Figure S1……………………………………………………………………………4
- Appendix Figure S2……………………………………………………………………………5
- Appendix Figure S3……………………………………………………………………………6
- Appendix Figure S4……………………………………………………………………………7
- Appendix Figure S5……………………………………………………………………………8
- Appendix Figure S6……………………………………………………………………………9
- Appendix Figure S7…………………………………………………………………………...10
- Appendix Figure S8…………………………………………………………………………...11
- Appendix Figure S9…………………………………………………………………………...12
- Appendix Table S1……………………………………………………………………………13
- Appendix Table S2……………………………………………………………………………13
- Appendix Table S3……………………………………………………………………………14
- Appendix Table S4……………………………………………………………………………15
- Appendix Table S5……………………………………………………………………………19
- Appendix References…………………………………………………………………………22

Isolation of the *unc-1(vlt10)* mutation

We induced random mutagenesis using 47 mM EMS (methanesulfonic acid ethyl ester, Sigma, St. Louis, Missouri, USA) in L4 animals of the AM141 strain. We incubated worms for 4 h at 20°C on this solution. After washing them they were pipetted onto NGM plates, seeded with OP50, and allowed them to lay the F1. F1 animals were bleached when they reached adulthood, and we searched among the F2 for animals uncoordinated and with abnormal aggregation patterns. Once isolated animals with the right phenotype (i.e. with abnormal motility and altered polyQ aggregation), they were outcrossed 5 times against the wild type background (N2, Bristol), before high-throughput sequencing of their genomic DNA. The reads provided by the sequencing service (Centre Nacional d'Anàlisi Genòmica – Centre de Regulacio Genomica, Barcelona, Spain) were mapped against the *C. elegans* WS245 reference using the mem algorithm implemented by the BWA software (Li, 2011, 2013). BAQ qualities were calculated and applied to the BAM alignments by using samtools calmd (Li, 2011). The SNP calling process was carried out by Freebayes (Garrison & Marth, 2012) with a minimum mapping quality of 57, a base quality threshold of 20, a minimum coverage of 6 and a minimum SNP quality of 20. A filtering process was stablished to look for the SNPs whose allelic frequencies were likely to have been affected by the selection process. SNPs were filtered out if they had more than one allele in all families, or if the reference allele was not present in any family, or if there were more than three mutant alleles when all families were considered. For the SNPs that passed all the filters a selection index was calculated. It consisted in the difference between the frequency of the most frequent allele in the back-crossed population and the frequency of that same allele in parental population, thus the SNPs with the highest differences between both populations would had the highest selection index. The predicted effect of each SNP was calculated by SnpEff (Cingolani *et al*, 2012).

To determine the molecular identity of this allele we sequenced the whole genome of this strain after six outcrossing steps and also the genome of the original strain (RVM10). EMS causes random lesions through the chromosomes and after outcrossing RVM10, most of these mutations would be lost during outcrossing, except the DNA changes that lie around the allele responsible for the aggregation phenotype. We took advantage of this, and when we compared both genomes

we observed a region with dense amount of mutations that "peaked" around the left arm of chromosome X (Appendix Figure S1A). Detailed analysis of this region showed that the *unc-1* gene, of RVM10, had a nonsense mutation in homozygosis that gives rise to premature stop codon, which would produce a putative null (Appendix Figure S1B). *unc-1* encodes a homologue of the Stomatin-like protein family from mammals (Lapatsina *et al*, 2012; Rajaram *et al*, 1998). The product of this gene has been shown to be involved in modulation of the electrical synapse (Chen *et al*, 2007) and sensitivity to anaesthetics (Rajaram *et al*, 1998). Null alleles of this gene are well-known to cause strong uncoordination (Chen *et al*, 2007), similar to *vlt10* mutant from our screen.

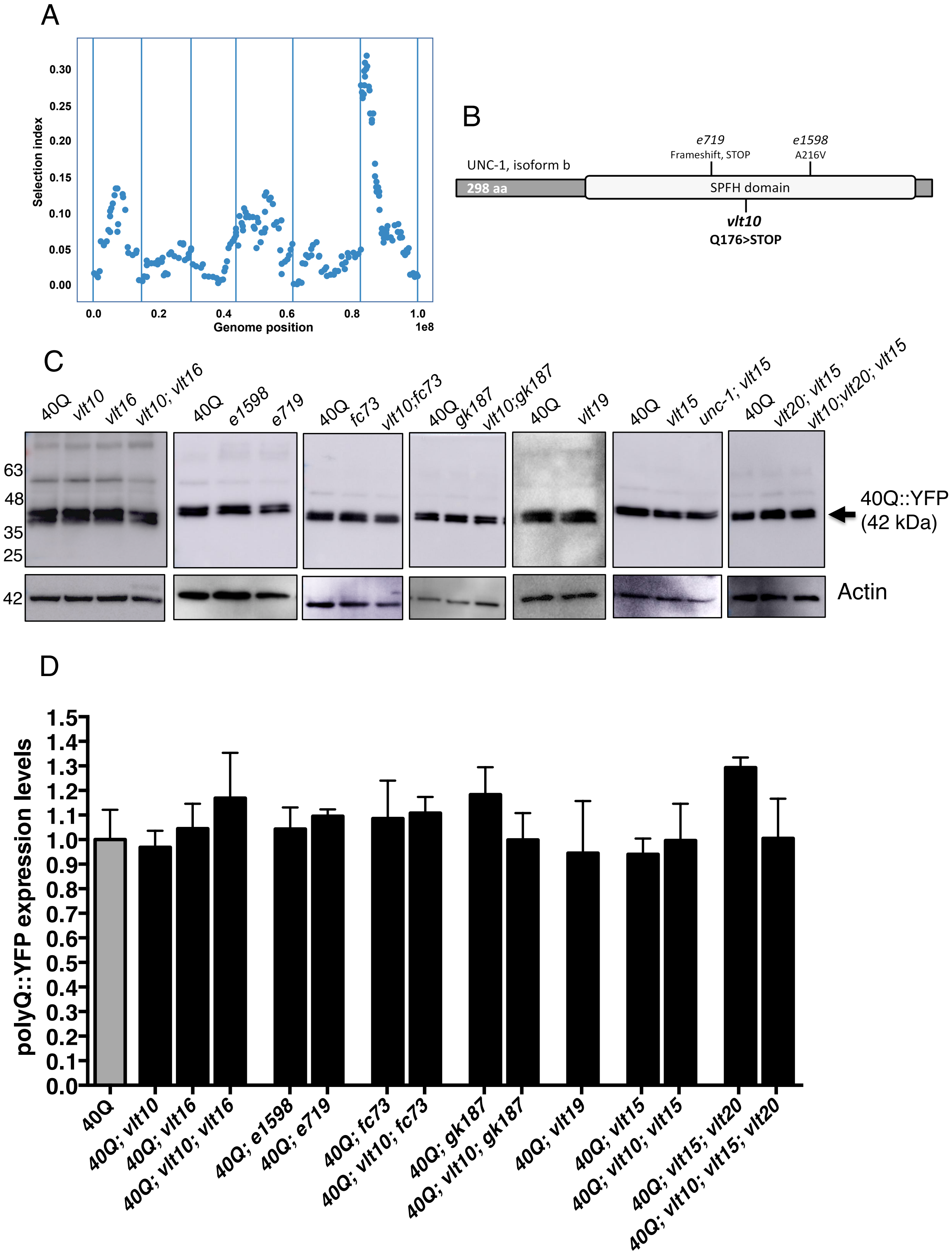
A
Selection index
0.30
0.25
0.20
0.15
0.10
0.05
0.00
0.0
0.2
0.4
0.6
0.8
1.0
1e8
Genome position
B
UNC-1, isoform b
298 aa
e719
Frameshift, STOP
e1598
A216V
SPFH domain
vlt10
Q176>STOP
C
40Q
vlt10
vlt16
vlt10; vlt16
40Q
e1598
e719
40Q
fc73
vlt10;fc73
40Q
gk187
vlt10;gk187
40Q
vlt19
40Q
vlt15
unc-1; vlt15
40Q
vlt20; vlt15
vlt10;vlt20; vlt15
63
48
35
25
42
40Q::YFP
(42 kDa)
Actin
D
polyQ::YFP expression levels
1.5
1.4
1.3
1.2
1.1
1.0
0.9
0.8
0.7
0.6
0.5
0.4
0.3
0.2
0.1
0.0
40Q
40Q; vlt10
40Q; vlt16
40Q; vlt10; vlt16
40Q; e1598
40Q; e719
40Q; fc73
40Q; vlt10; fc73
40Q; gk187
40Q; vlt10; gk187
40Q; vlt19
40Q; vlt15
40Q; vlt10; vlt15
40Q; vlt15; vlt20
40Q; vlt10; vlt15; vlt20

Appendix Figure S1. *vlt10* allele produces a premature stop codon in *unc-1/Stoml3,* which enhances polyQ aggregation without altering *40Q::YFP* transgene expression. (**A**) A map of SNPs used to determine the location of *vlt10* in the *C. elegans* genome. (**B**) Gene diagram showing the location in the SPFH domain of the premature stop codon (Q176 > STOP) produced by *vlt10* in *unc-1,* which likely produces a truncated UNC-1 and a putative loss of function protein. (**C**) Immunoblots showing the 40Q::YFP protein (42 kDa) in all genetic backgrounds. The double band identified by the anti-polyQ antibody most likely reflects proteolytic cleavage of 40Q::YFP. We used actin to normalize loading levels. (**D**) ImageJ quantification of 40Q::YFP protein levels in all genetic backgrounds, normalized to 40Q::YFP wild type (grey) to verify any changes according to transgene expression. All plotted data show the mean ± standard error of the mean (SEM). We performed ANOVA test, with post-hoc Tukey test, which shows that there were not significant differences between samples. Three biological samples were evaluated for each strain.

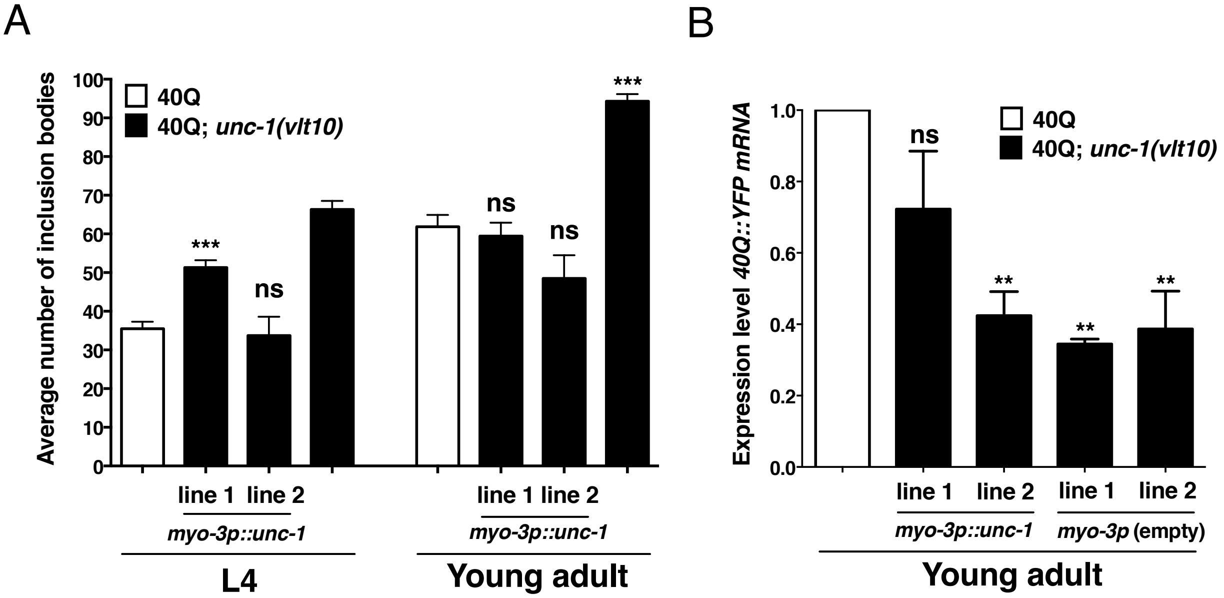

Appendix Figure S2. **Reintroduction of *unc-1* in muscle cells of *vlt10* mutants reduces *40Q::YFP* expression via a change in polyQ expression**. (**A**) The average number of polyQ inclusion bodies in muscle cells after introducing a construct containing the cDNA of *unc-1* under the control of the promoter of the *myo-3* gene in both transgenic stable lines (line 1 and line 2) expressing the *myo-3p::unc-1* (cDNA) transgene. (**B**) Relative expression levels of the *unc-54p::40Q::YFP* transgene in both transgenic stable lines (line 1 and line 2) expressing the *myo-3p::unc-1* (cDNA) or the *myo-3p* promoter-only control transgene.. Data information: The plotted data show mean ± standard error of the mean (SEM). At least thirty animals were used and at least three independent experiments were performed per experiment and condition. ****P*

< 0.001; $**P < 0.01$; ns: not significant, as calculated using the one-way ANOVA with post-hoc Tukey test.

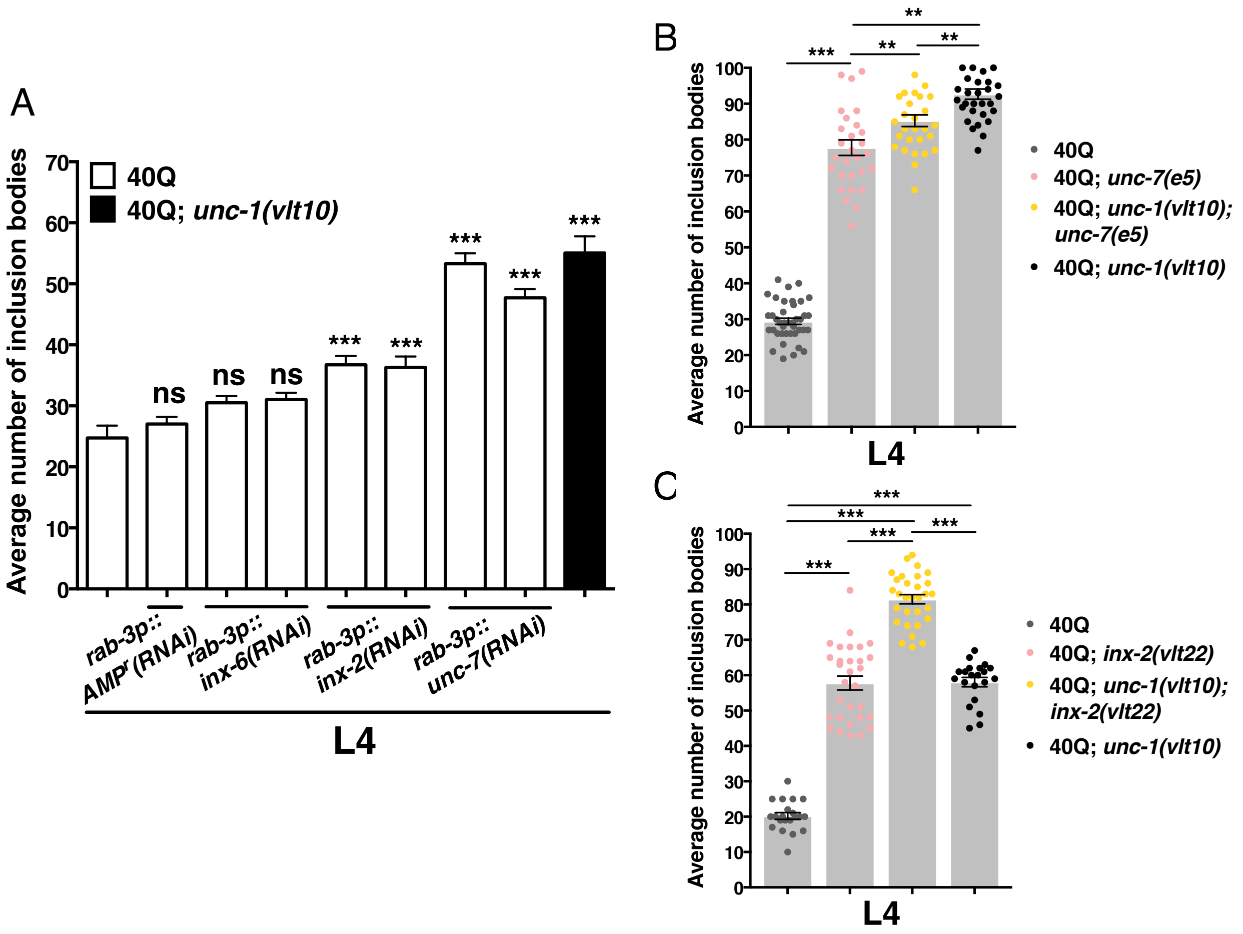

Appendix Figure S3. Neuronal disruption of innexins INX-2 and UNC-7 enhances aggregation of polyQs in muscle cells. (**A**) The average number of polyQ inclusion bodies after tissue-specific silencing of *inx-2*, *inx-6* and *unc-7* in neurons. (**B**) The average number of polyQ inclusion bodies in muscle cells for double mutant *unc-7(e5)*; *unc-1(vlt10)* compared with the single mutants and wild type animals . (**C**) The average number of polyQ inclusion bodies for double mutant *unc-1(vlt10); inx-2(vlt22)* compared with the single mutants and wild type animals. Data information: The plotted data show mean ± standard error of the mean (SEM). Thirty animals per condition and/or strain and per experiment were analysed. Each analysis has been reproduced at least three times. $***P < 0.001$; $**P < 0.01$; ns: not significant in reference to the wild type *40Q::YFP* strain (graph A), as calculated using the one-way ANOVA with post-hoc Tukey test.

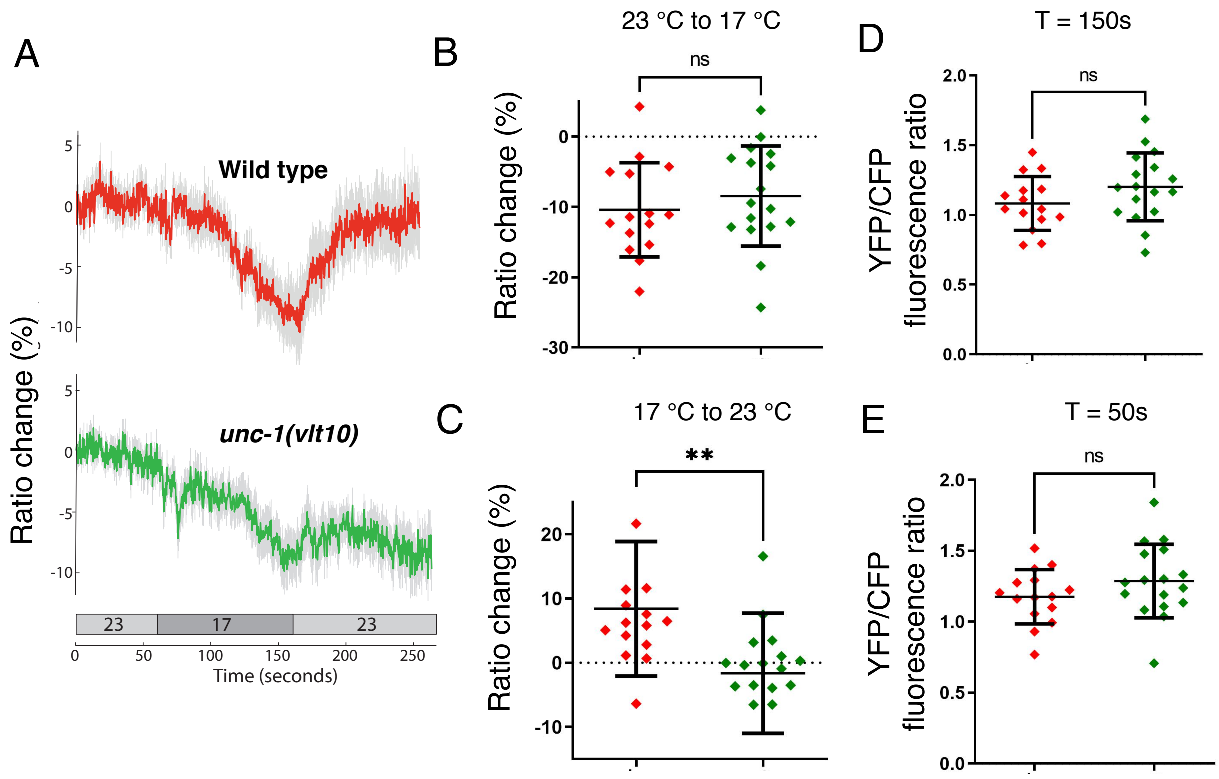

Appendix Figure S4. Calcium imaging in ASJ of *unc-1* mutants. (**A**–**E**) Calcium imaging in ASJ neurons with and without *unc-1(vlt10)* transgene in response to temperature shifts. (**A**) Mean traces of % YFP/CFP fluorescence ratio change. Grey trace indicates SEM, bar at the bottom indicates the perfusion temperature. (**B**) Scatter plot showing individual ratio changes in response to the temperature shift from 23°C to 17°C, made by comparing the mean YFP/CFP fluorescence ratio at T = 50s with T = 150s using a 20-frame window. (**C**) Scatter plot showing individual ratio changes in response to the temperature shift from 17°C to 23°C, made by comparing YFP/CFP fluorescence ratio at T = 150s with T = 50s. (**D**–**E**) Scatter plots showing the YFP/CFP fluorescence ratio at T = 150s (D) and T = 50s (E). Error bars show mean ± SEM. Data information: n = 15 (wild type), 17 (*unc-1(vlt10)*) animals, recorded over at least three days. ** $P < 0.01$, ns: not significant, as calculated using unpaired t-test.

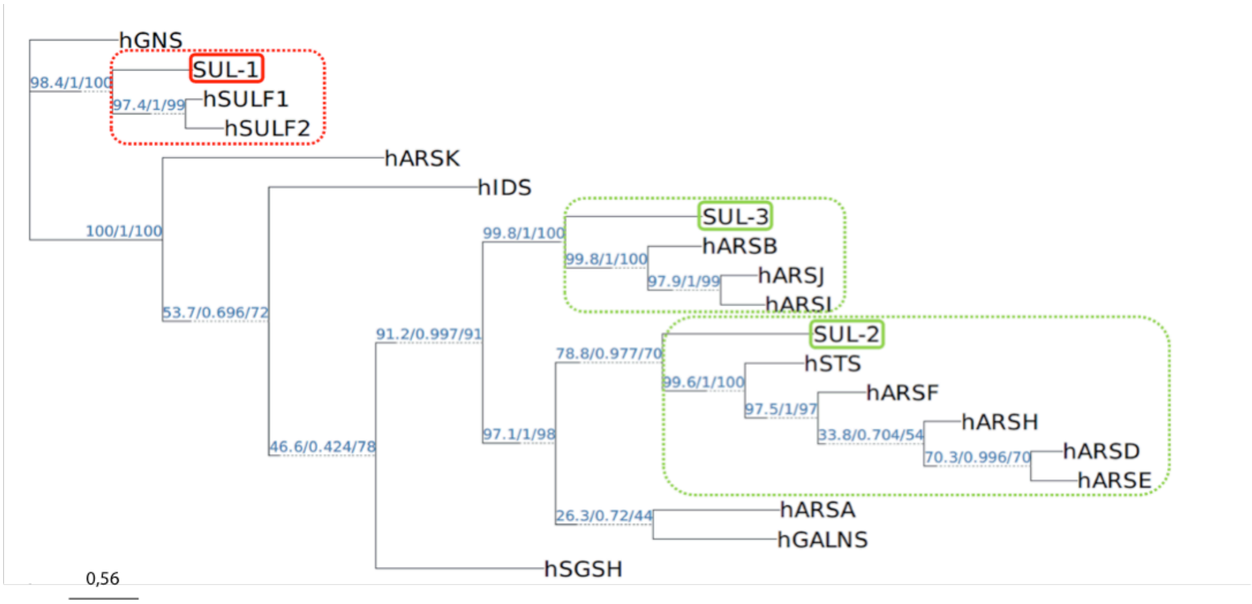

Appendix Figure S5. Phylogenetic analysis of *C. elegans* and human sulfatases. The diagram shows the phylogenetic tree of all *C. elegans* sulfatases (SUL-1, SUL-2 and SUL-3) obtained using the MUSCLE (Edgar, 2004) and IQ-TREE version 1.6.8 software (Chernomor *et al*, 2016; Li, 2013; Nguyen *et al*, 2015). The phylogenetic tree shows bootstrap values which provides confidence values for each node. This analysis suggests that SUL-2 and SUL-3 are closer to arylsulfatases, while SUL-1 is closer to hSULF1 and hSULF2.

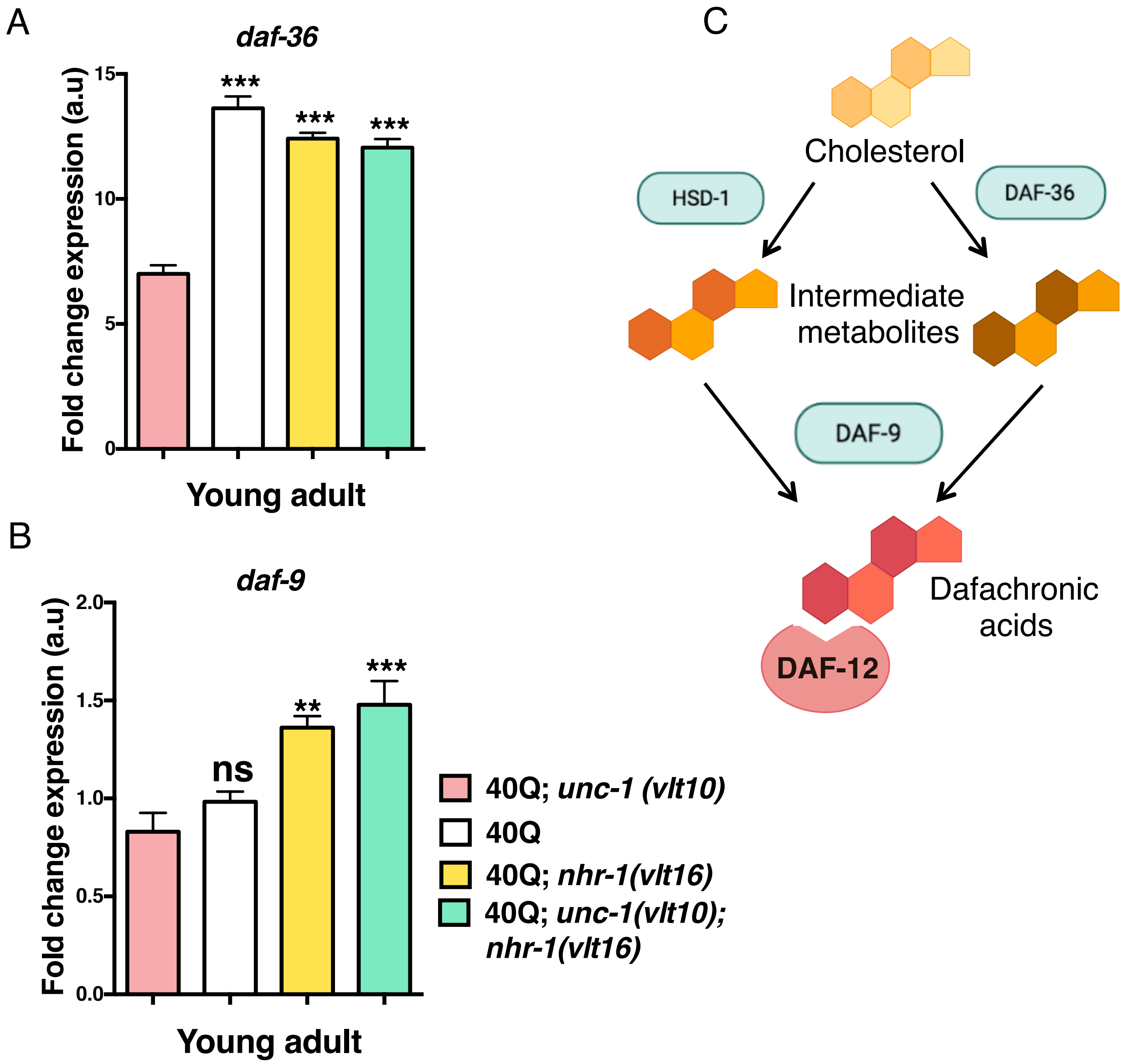

Appendix Figure S6. NHR-1 regulates the expression of some genes related to DAF-12 signalling. (**A**) Graph of the relative expression of *daf-36*, a gene that encodes an oxidoreductase enzyme involved in cholesterol processing, in *unc-1* and *nhr-1* single and double mutants compared to wild type worms. (**B**) Graph of the relative expression of *daf-9*, a cytochrome involved in dafachronic acids synthesis, in *unc-1* and *nhr-1* single and double mutants compared to wild type worms. (**C**) Diagram of parts of the synthesis pathway of dafachronic acids, placing the activity of the HSD-1, DAF-36 and DAF-9 enzymes. Data information: The plotted data show mean ± standard error of the mean (SEM). Six biological replicates were analysed for each strain. $^{**}P < 0.01$; $^{***}P < 0.005$, as calculated using the one-way ANOVA with post-hoc Tukey test and compared to *unc-1* mutants

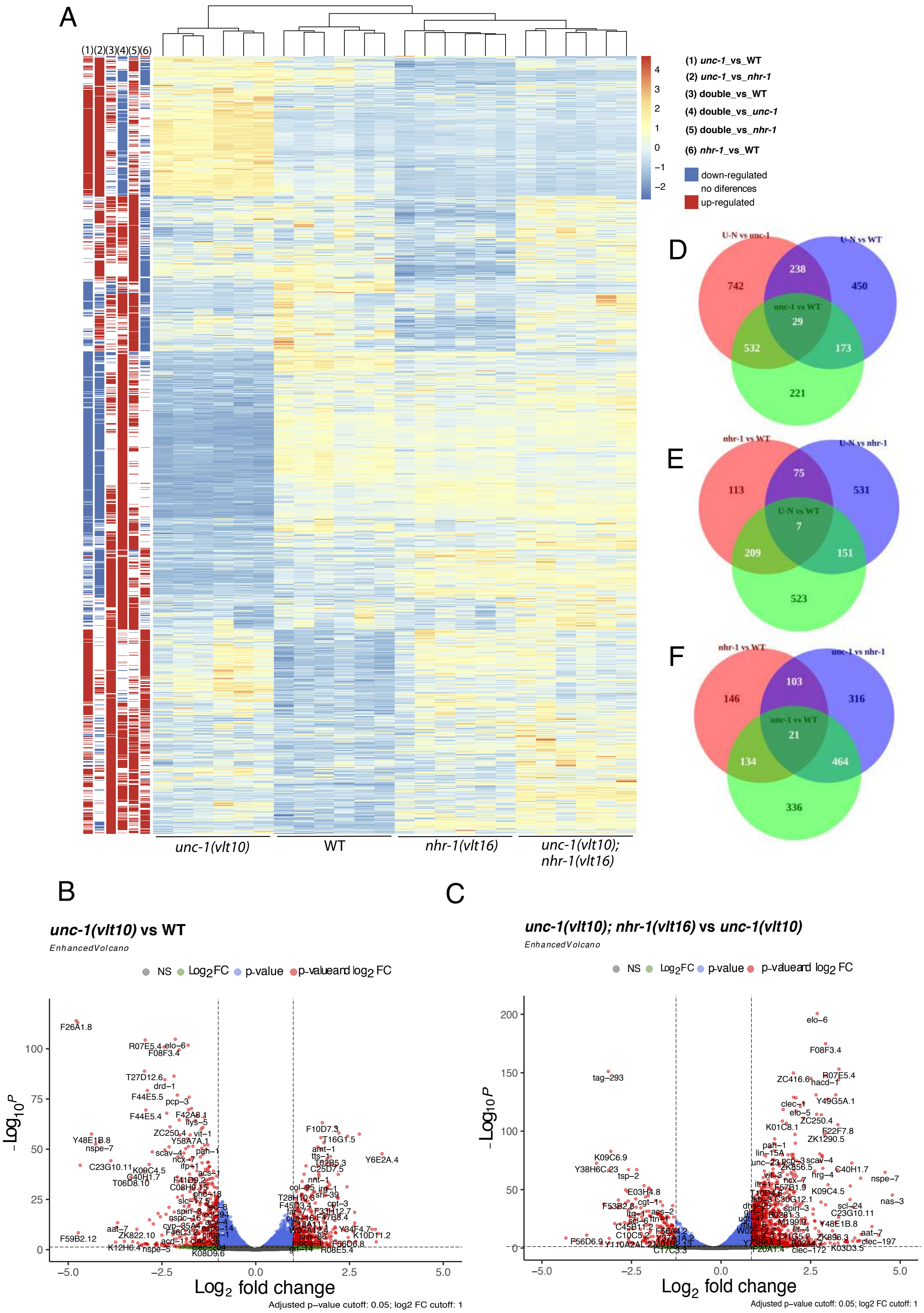
A
(1)(2)(3)(4)(5)(6)
(1) unc-1_vs_WT
(2) unc-1_vs_nhr-1
(3) double_vs_WT
(4) double_vs_unc-1
(5) double_vs_nhr-1
(6) nhr-1_vs_WT
down-regulated
no diferences
up-regulated
4
3
2
1
0
−1
−2
unc-1(vlt10)
WT
nhr-1(vlt16)
unc-1(vlt10); nhr-1(vlt16)
D
U-N vs unc-1
U-N vs WT
unc-1 vs WT
742
238
450
29
532
173
221
E
nhr-1 vs WT
U-N vs nhr-1
U-N vs WT
113
75
531
7
209
151
523
F
nhr-1 vs WT
unc-1 vs nhr-1
unc-1 vs WT
146
103
316
21
134
464
336
B
unc-1(vlt10) vs WT
EnhancedVolcano
NS Log2FC p-value p-value and log2 FC
−Log10 P
Log2 fold change
Adjusted p−value cutoff: 0.05; log2 FC cutoff: 1
C
unc-1(vlt10); nhr-1(vlt16) vs unc-1(vlt10)
EnhancedVolcano
NS Log2FC p-value p-value and log2 FC
−Log10 P
Log2 fold change
Adjusted p−value cutoff: 0.05; log2 FC cutoff: 1

Appendix Figure S7. Transcriptomic signature of *unc-1* and *nhr-1* mutants into a polyQ background. (A) Heatmap showing DESeq2 normalized read counts of differentially expressed genes (corrected $P < 0.05$ and fold change > 2, see Methods). Data were scaled and centred by gene. Heat map rows are based on Z-scores. The left side of the heatmap includes the statistically significant changes between genotypes [1: 40Q; *unc-1(vlt10)* vs 40Q; 2: 40Q; *unc-1(vlt10)* vs 40Q; *nhr-1(vlt16)*; 3: double mutant vs 40Q; 4: double mutant vs 40Q; *unc-1(vlt10)*; 5: double mutant vs 40Q; *nhr-1(vlt16);* and 6; *nhr-1(vlt16)* vs 40Q]. **(B–C)** Volcano plots showing the log2 fold change versus the -log10 of the DESeq2 corrected p-value for each pair of genotypes (40Q; *unc-1* vs 40Q and 40Q; *unc-1;nhr-1* vs 40Q; *unc-1*). **(D–F)** Venn diagrams showing the number of genes that are differentially expressed between genotypes. For example, graph D shows that there are 523 genes that are differentially expressed between double and 40Q; *unc-1* mutants and also between *unc-1* and 40Q, but not between double mutants and 40Q. This shows that these genes are rescued by the *vlt16* allele. In contrast, there are 173 differentially expressed genes that are specific to animals carrying the *vlt10* allele and that are not altered by *vlt16*.

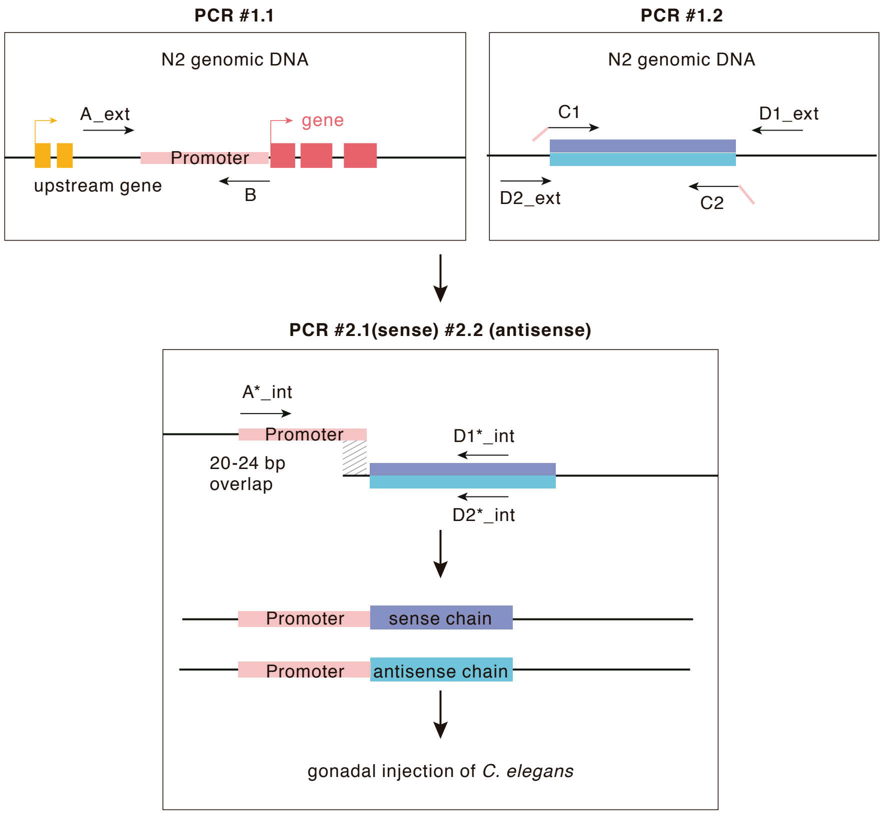

Appendix Figure S8. Diagram showing the production of PCR-generated constructs to induce RNAi. A promoter sequence for a gene fragment was assembled to promote tissue-specific RNAi via fusion PCR. External (ext) and internal (int) primers were used to amplify the promoter region (red), sense (purple) and antisense (blue) sequence from genomic DNA. The primers had 20 complementary nucleotides (red tail on primers) that allow fusion using nested primers.

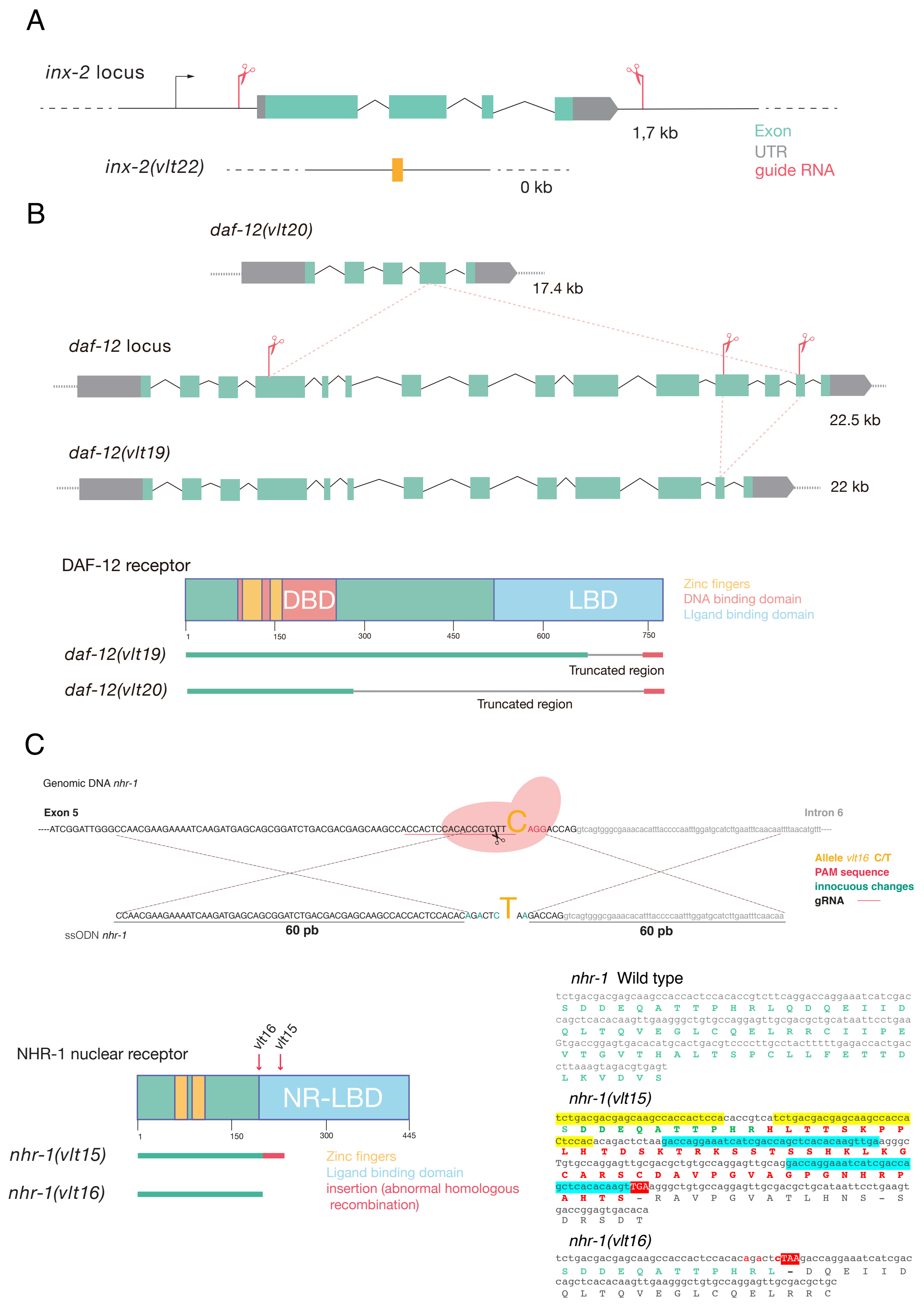
A
inx-2 locus
1,7 kb
Exon
UTR
guide RNA
inx-2(vlt22)
0 kb
B
daf-12(vlt20)
17.4 kb
daf-12 locus
22.5 kb
daf-12(vlt19)
22 kb
DAF-12 receptor
DBD
LBD
Zinc fingers
DNA binding domain
LIgand binding domain
1
150
300
450
600
750
daf-12(vlt19)
Truncated region
daf-12(vlt20)
Truncated region
C
Genomic DNA nhr-1
Exon 5
Intron 6
----ATCGGATTGGGCCAACGAAGAAAATCAAGATGAGCAGCGGATCTGACGACGAGCAAGCCACCACTCCACACCGTCTT C AGGACCAGgtcagtgggcgaaacacatttaccccaatttggatgcatcttgaatttcaacaattttaacatgttt----
Allele vlt16 C/T
PAM sequence
innocuous changes
gRNA
CCAACGAAGAAAATCAAGATGAGCAGCGGATCTGACGACGAGCAAGCCACCACTCCACACAGACTC T AAGACCAGgtcagtgggcgaaacacatttaccccaatttggatgcatcttgaatttcaacaa
60 pb
60 pb
ssODN nhr-1
nhr-1 Wild type
tctgacgacgagcaagccaccactccacaccgtcttcaggaccaggaaatcatcgac
S D D E Q A T T P H R L Q D Q E I I D
cagctcacacaagttgaagggctgtgccaggagttgcgacgctgcataattcctgaa
Q L T Q V E G L C Q E L R R C I I P E
Gtgaccggagtgacacatgcactgacgtccccttgcctacttttgagaccactgac
V T G V T H A L T S P C L L F E T T D
cttaaagtagacgtgagt
L K V D V S
NHR-1 nuclear receptor
vlt16
vlt15
NR-LBD
1
150
300
445
nhr-1(vlt15)
nhr-1(vlt16)
Zinc fingers
Ligand binding domain
insertion (abnormal homologous recombination)
nhr-1(vlt15)
tctgacgacgagcaagccaccactccacaccgtcatctgacgacgagcaagccacca
S D D E Q A T T P H R H L T T S K P P
Ctccacacagactctaagaccaggaaatcatcgaccagctcacacaagttgaagggc
L H T D S K T R K S S T S S H K L K G
Tgtgccaggagttgcgacgctgtgccaggagttgcaggaccaggaaatcatcgacca
C A R S C D A V P G V A G P G N H R P
gctcacacaagtTGAagggctgtgccaggagttgcgacgctgcataattcctgaagt
A H T S - R A V P G V A T L H N S - S
gaccggagtgacaca
D R S D T
nhr-1(vlt16)
tctgacgacgagcaagccaccactccacacagactcTAAgaccaggaaatcatcgac
S D D E Q A T T P H R L - D Q E I I D
cagctcacacaagttgaagggctgtgccaggagttgcgacgctgc
Q L T Q V E G L C Q E L R R C

Appendix Figure S9. CRISPR strategies to modify gene expression. (**A**) The *inx-2* gene was disrupted using two sgRNAs that cut all coding sequences of the gene. The *vlt22* allele obtained from this experiment represents a complete loss of the *inx-2* gene. (**B**) The *daf-12* gene was disrupted using a combination of two gRNAs that target exons 12 and 14. *vlt19* is a 500-bp deletion that affects the ligand-binding domain (LBD) in the C-terminal region of the DAF-12 protein. (**C**) Diagram to introduce the *vlt16* mutation in *nhr-1,* which emulates the allele *n6242* and the novel *vlt15* allele as a result of an abnormal homologous recombination containing duplicate insertions (yellow and blue shading). *vlt15* induces a frameshift (red pattern) that produces a change (C/T) and a premature TGA stop codon upstream. Both alleles, *vlt16* and *vlt15*, are putative nulls.

Appendix Table S1. Modifier genes isolated in this work.

**Gene**	Orthologue in mammals	Mutation	Type of modifier of polyQ aggregation
unc-1	Stomatin like protein 3	Putative null (nonsense mutation)	Enhancer
tmem-24	C2CD2	Putative null (nonsense mutation)	Enhancer
pqn-62	Prion-like protein (no orthologue known in mammals)	Putative null (nonsense mutation)	Enhancer
sir-2.3	SIRT4	Putative null (nonsense mutation)	Suppressor

Appendix Table S2. Relative expression level of 40Q::YFP transgene.

	40Q	***40Q; unc-1(vlt10) + myo-3p::unc-1(cDNA)* line 1**	***40Q; unc-1(vlt10) + myo-3p::unc-1(cDNA)* line 2**	***40Q; unc-1(vlt10) + myo-3p* line 1**	***40Q; unc-1(vlt10) + myo-3p* line 2**
**EXP_1**	**1**	**0.4**	**0.521**	**0.348**	**0.553**

**EXP_2**	**1**	**0.849**	**0.294**	**0.319**	**0.418**
**EXP_3**	**1**	**0.919**	**0.457**	**0.367**	**0.189**

Table S3. Stomatin-like proteins and innexins are required to maintain protein homeostasis

**Genotype**	**Focis (%)[1]**	**P value[2]**	**Protein**	**Function[3]**	**Expression[3]**
unc-1(vlt10)	171.7 ± 3.1	***	Stomatin-like protein	Locomotion<br><br>Sensitivity to volatile anaesthetics	NS-WE[4]
unc-24(e138)	149.1 ± 3.8	***	Stomatin-like protein	Locomotion<br><br>Sensitivity to volatile anaesthetics	NS-WE
mec-2(e75)	114.5 ± 5.2	ns	Stomatin-like protein	Mechanosensation	Mechanosensory neurons
unc-7(e5)	178.7 ± 4.7	***	Innexin	Locomotion<br>Sensitivity to volatile anaesthetics<br>Sensitivity to ivermectin	NS[4]<br><br>Glial cells<br><br>Mesoderm
unc-9(ec27)	185.4 ± 2.7	***	Innexin	Locomotion<br>Sensitivity to volatile anaesthetics<br>Sensitivity to ivermectin<br>Electrical coupling of muscle cells	NS-WE<br><br>Enteric muscles<br><br>RS[4]<br><br>Hypodermis

					Glial cells<br>Embryos
inx-2(vlt22)	175.4 ± 4.3	***	Innexin	Role initial cellular proliferation stage	NS-AVK neuron<br>Pharynx<br>Intestine<br>Embryos
inx-7(ok2319)	98.7 ± 3.8	ns	Innexin	Unknown	NS-WE<br>Pharynx<br>RS<br>Mesoderm<br>Embryos
inx-6(rr5)	98.8 ± 4.1	ns	Innexin	Synchronized pharyngeal muscle contractions	Pharynx<br>Mesoderm<br>Embryos

[1]Average of inclusion bodies normalised with the background of wildtype siblings ±Standard Error of the Mean.
[2]ANOVA with post-hoc analysis of Tukey: ***p<0.001 referred to wild type; ns: no statistically significant.
[3]Function and expression according by the review of Altun et al., (Altun et al., 2009).
[4]NS-WE: nervous system-wide expression; NS: some neurons; RS: reproductive system.

Appendix Table S4. *C. elegans* strains used in this work.

Strain[1]	Genotype	Reference
Bristol N2	*Caenorhabditis elegans* wild type background	Brenner, 1974
AM141	*rmIs133[unc-54p::40Q::YFP] X*	Morley et al., 2002
RVM10	*rmIs133[unc-54p::40Q::YFP] X*; *unc-1(vlt10) X*	This work
CB719	*unc-1(e719)* X	Park and Horvitz, 1986
CB1598	*unc-1(e1598) X*	Park and Horvitz, 1986
RVM241	*unc-1(vlt10) X*	This work
RVM23	*rmIs133[unc-54p::40Q::YFP] X*; *unc-1(e719) X*	This work
RVM20	*rmIs133[unc-54p::40Q::YFP] X*; *unc-1(e1598) X*	This work
AM140	*rmIs132[unc-54p::35Q::YFP] X*	Morley et al., 2002
RVM21	*rmIs132[unc-54p::35Q::YFP]; unc-1(e1598) X*	This work
RVM24	*rmIs132[unc-54p::35Q::YFP] X; unc-1(e719) X*	This work
RVM26	*rmIs132[unc-54p::35Q::YFP] X; unc-1(vlt10) X*	This work
NL5901	*pkIs2386[unc-54p::α-synucleina::YFP + unc-119(+)] IV*	van Ham et al., 2008
CL2006	*dvIs2[pCL12(unc-54/Abeta human peptide 1-42 minigene)+pRF4]*	Link, 1995
RVM317	*pkIs2386[unc-54p::α-synuclein::YFP + unc-119(+)] IV; unc-1(vlt10) X*	This work
RVM328	*dvIs2[pCL12(unc-54/Abeta human peptide 1-42 minigene)+pRF4], unc-1(vlt10) X*	This work
RVM47	*rmIs133[unc-54p::40Q::YFP] X; unc-1(vlt10) X; vltEx47[myo-3p::unc-1(o/e); myo-2p::mCherry strain 1]*	This work
RVM155	*rmIs133[unc-54p::40Q::YFP] X; unc-1(vlt10) X; vltEx155[myo-3p::unc-1(o/e); myo-2p::mCherry strain 2]*	This work
RVM158	*rmIs133[unc-54p::40Q::YFP] X; unc-1(vlt10) X; vltEx158[myo-3p(o/e)); myo-2p::mCherry strain 1]*	This work
RVM159	*rmIs133[unc-54p::40Q::YFP] X; unc-1(vlt10) X; vltEx159[myo-3p(o/e)); myo-2p::mCherry strain 2]*	This work
RVM44	*rmIs133[unc-54p::40Q::YFP] X*; *unc-1(vlt10) X; vltEx44[rab-3p::unc-1(o/e)); myo-2p::mCherry strain 1]*	This work
RVM48	*rmIs133[unc-54p::40Q::YFP] X*; *unc-1(vlt10) X; vltEx48[rab-3p::unc-1(o/e)); myo-2p::mCherry strain 2]*	This work
RVM42	*rmIs133[unc-54p::40Q::YFP] X*; *vltEx42[rab-3p::AMP^r(RNAi)); myo-2p::mCherry strain 1]*	This work
RVM43	*rmIs133[unc-54p::40Q::YFP] X; vltEx43[rab-3p:: AMP^r(RNAi)); myo-2p::mCherry strain 2]*	This work
RVM71	*rmIs133[unc-54p::40Q::YFP] X*; *vltEx71[rab-3p::unc-1(RNAi)); myo-2p::mCherry strain 1]*	This work
RVM72	*rmIs133[unc-54p::40Q::YFP] X; vltEx72[rab-3p::unc-1(RNAi)); myo-2p::mCherry strain 2]*	This work
RVM205	*rmIs133[unc-54p::40Q::YFP] X; vltEx205[rab-3p(o/e)); myo-*	This work

	2p::mCherry strain 1]	
RVM206	rmIs133[unc-54p::40Q::YFP] X; vltEx206[rab-3p(o/e)); myo-2p::mCherry strain 2]	This work
RVM201	rmIs133[unc-54p::40Q::YFP] X; vltEx201[rab-3p::unc-1(o/e)); myo-2p::mCherry strain 1]	This work
RVM221	rmIs133[unc-54p::40Q::YFP] X; vltEx221[rab-3p::unc-1(o/e)); myo-2p::mCherry strain 2]	This work
RVM227	rmIs133[unc-54p::40Q::YFP] X; vltEx227[rab-3p::unc-1(n494)(o/e)); myo-2p::mCherry strain 1]	This work
RVM230	rmIs133[unc-54p::40Q::YFP] X; vltEx230[rab-3p::unc-1(n494)(o/e)); myo-2p::mCherry strain 2]	This work
RVM360	rmIs133[unc-54p::40Q::YFP] X; vltEx360[trx-1p::AMP^r(RNAi)); myo-2p::mCherry strain 1]	This work
RVM361	rmIs133[unc-54p::40Q::YFP] X; vltEx361[trx-1p::AMP^r(RNAi)); myo-2p::mCherry strain 2]	This work
RVM364	rmIs133[unc-54p::40Q::YFP] X; vltEx364[trx-1p::unc-1(RNAi)); myo-2p::mCherry strain 1]	This work
RVM365	rmIs133[unc-54p::40Q::YFP] X; vltEx365[trx-1p::unc-1(RNAi)); myo-2p::mCherry strain 2]	This work
RVM368	rmIs133[unc-54p::40Q::YFP] X; vltEx368[oig-1p::AMP^r(RNAi); osm3p::AMP^r(RNAi); myo-2p::mCherry strain 1]	This work
RVM369	rmIs133[unc-54p::40Q::YFP] X; vltEx369[oig-1p::AMP^r(RNAi); osm-3p::AMP^r(RNAi); myo-2p::mCherry strain 2]	This work
RVM355	rmIs133[unc-54p::40Q::YFP] X; vltEx355[oig-1p::unc-1(RNAi); osm-3p::unc-1(RNAi); myo-2p::mCherry strain 1]	This work
RVM356	rmIs133[unc-54p::40Q::YFP] X; vltEx356[oig-1p::unc-1(RNAi); osm-3p::unc-1(RNAi); myo-2p::mCherry strain 2]	This work
RVM362	rmIs133[unc-54p::40Q::YFP] X; vltEx362[glr-1p::AMP^r(RNAi); gpa-9p::AMP^r(RNAi); myo-2p::mCherry strain 1]	This work
RVM363	rmIs133[unc-54p::40Q::YFP] X; vltEx363[glr-1p::AMP^r(RNAi); gpa-9p::AMP^r(RNAi); myo-2p::mCherry strain 2]	This work
RVM366	rmIs133[unc-54p::40Q::YFP] X; vltEx366[glr-1p::unc-1(RNAi); gpa-9p::unc-1(RNAi); myo-2p::mCherry strain 1]	This work
RVM367	rmIs133[unc-54p::40Q::YFP] X; vltEx367[glr-1p::unc-1(RNAi); gpa-9p::unc-1(RNAi); myo-2p::mCherry strain 2]	This work
RVM386	rmIs133[unc-54p::40Q::YFP] X; unc-1 (vlt10) X; vltEx386[oig-1p::unc-1(o/e); myo-2p::mCherry strain 1]	This work
RVM387	rmIs133[unc-54p::40Q::YFP] X; unc-1 (vlt10) X; vltEx387[oig-1p::unc-1(o/e); myo-2p::mCherry strain 2]	This work
RVM384	rmIs133[unc-54p::40Q::YFP] X; unc-1 (vlt10) X; vltEx384[osm-3p::unc-1(o/e); myo-2p::mCherry strain 1]	This work
RVM385	rmIs133[unc-54p::40Q::YFP] X; unc-1 (vlt10) X; vltEx385[osm-3p::unc-1(o/e); myo-2p::mCherry strain 2]	This work
AM101	rmIs110[F25B3.3-p::40Q::YFP]	Brignull et al., 2006
RVM27	rmIs110[F25B3.3-p::40Q::YFP]; unc-1(vlt10) X	This work
RVM445	rmIs110[F25B3.3-p::40Q::YFP]; unc-1(vlt10) X; vltEx445[osm-3p::unc-1(o/e); myo-2p::mCherry strain 1]	This work
RVM446	rmIs110[F25B3.3-p::40Q::YFP]; unc-1(vlt10) X; vltEx445[osm-3p::unc-1(o/e); myo-2p::mCherry strain 2]	This work
CB75	mec-2(e75) X	Chalfie and Sulston, 1981

CB138	*unc-24(e138) IV*	Brenner, 1974
RVM208	*rmIs133[unc-54p::40Q::YFP] X*; *mec-2(e75) X*	This work
RVM192	*rmIs133[unc-54p::40Q::YFP] X*; *unc-24(e138) IV*	This work
CB5	*unc-7(e5) X*	Brenner, 1974
FH85	*unc-9(ec27) X*	Sedensky and Meneely, 1987
MR127	*inx-6(rr5) IV*	Li et al., 2003
RB1792	*inx-7(ok2319) IV*	The C. elegans deletion mutant consortium 2012
RVM209	*rmIs133[unc-54p::40Q::YFP] X*; *unc-7(e5) X*	This work
RVM175	*rmIs133[unc-54p::40Q::YFP] X*; *unc-9(ec27)* X	This work
RVM196	*rmIs133[unc-54p::40Q::YFP] X*; *inx-2(vlt22) X*	This work
RVM197	*rmIs133[unc-54p::40Q::YFP] X*; *inx-6(rr5) IV*	This work
RVM218	*rmIs133[unc-54p::40Q::YFP] X*; *inx-7(ok2319) IV*	This work
RVM340	*rmIs133[unc-54p::40Q::YFP] X; vltEx340[rab-3p::inx-6(RNAi)); myo-2p::mCherry strain 1]*	This work
RVM341	*rmIs133[unc-54p::40Q::YFP] X; vltEx341[rab-3p::inx-6(RNAi)); myo-2p::mCherry strain 2]*	This work
RVM333	*rmIs133[unc-54p::40Q::YFP] X; vltEx333[rab-3p::inx-2(RNAi)); myo-2p::mCherry strain 1]*	This work
RVM342	*rmIs133[unc-54p::40Q::YFP] X; vltEx342[rab-3p::inx-2(RNAi)); myo-2p::mCherry strain 2]*	This work
RVM335	*rmIs133[unc-54p::40Q::YFP] X; vltEx335[rab-3p::unc-7(RNAi)); myo-2p::mCherry strain 1]*	This work
RVM337	*rmIs133[unc-54p::40Q::YFP] X; vltEx337[rab-3p::unc-7(RNAi)); myo-2p::mCherry strain 2]*	This work
RVM346	*rmIs133[unc-54p::40Q::YFP] X; unc-7(e5) X*	This work
RVM337	*rmIs133[unc-54p::40Q::YFP] X; unc-1(vlt10) X; unc-7(e5) X*	This work
RVM349	*rmIs133[unc-54p::40Q::YFP] X; inx-2(vlt22) X*	This work
RVM348	*rmIs133[unc-54p::40Q::YFP] X; unc-1(vlt10) X; inx-2(vlt22) X*	This work
CW911	*ssu-1(fc73) V; unc-1(e580) X; rol-6(su1006)*	Carroll et al., 2006
RVM142	*rmIs133[unc-54p::40Q::YFP] X*; *ssu-1(fc73) V*	This work
RVM143	*rmIs133[unc-54p::40Q::YFP] X*; *unc-1(e580)* X	This work
RVM144	*rmIs133[unc-54p::40Q::YFP] X*; *ssu-1(fc73)* V; *unc-1(e580)* X	This work
RVM188	*rmIs133[unc-54p::40Q::YFP] X; unc-1 (vlt10) X; vltEx188[trx-1p::AMP^r(RNAi)); myo-2p::mCherry strain 1]*	This work
RVM189	*rmIs133[unc-54p::40Q::YFP] X; unc-1 (vlt10) X; vltEx189[trx-1p::AMP^r(RNAi)); myo-2p::mCherry strain 2]*	This work

RVM176	*rmIs133[unc-54p::40Q::YFP] X; unc-1(vlt10) X; vltEx176[trx-1p::ssu-1(RNAi)); myo-2p::mCherry strain 1]*	This work
RVM232	*rmIs133[unc-54p::40Q::YFP] X; unc-1(vlt10) X; vltEx232[trx-1p::ssu-1(RNAi)); myo-2p::mCherry strain 2]*	This work
VC382	*sul-2(gk187) V*	The C. elegans deletion mutant consortium 2012
RVM224	*rmIs133[unc-54p::40Q::YFP] X; sul-2(gk187) V*	This work
RVM225	*rmIs133[unc-54p::40Q::YFP] X; sul-2(gk187) V; unc-1(vlt10) X*	This work
RVM320	*rmIs133[unc-54p::40Q::YFP] X; daf-12(vlt19) X*	This work
AA292	*daf-36(k114) V*	Rottiers et al., 2006
RVM390	*rmIs133[unc-54p::40Q::YFP] X; daf-36(k114) V; vltEx390[daf-36p::daf-36; myo-2p::mCherry]*	This work
RVM288	*rmIs133[unc-54p::40Q::YFP] X; nhr-1(vlt15) X*	This work
RVM289	*rmIs133[unc-54p::40Q::YFP] X; unc-1(vlt10) X; nhr-1(vlt15) X*	This work
RVM293	*rmIs133[unc-54p::40Q::YFP] X; nhr-1(vlt16) X*	This work
RVM294	*rmIs133[unc-54p::40Q::YFP] X; unc-1(vlt10) X; nhr-1(vlt16) X*	This work
RVM290	*rmIs133[unc-54p::40Q::YFP] X; unc-1(vlt10) X; nhr-1(vlt15) X; vltEx290[unc-54p::nhr-1(o/e); myo-2p::mCherry strain 1]*	This work
RVM291	*rmIs133[unc-54p::40Q::YFP] X; unc-1(vlt10) X; nhr-1(vlt15) X; vltEx291[unc-54p::nhr-1(o/e); myo-2p::mCherry strain 2]*	This work
RVM449	*rmIs133[unc-54p::40Q::YFP] X; unc-1(vlt10) X; nhr-1(vlt15) X; vltEx290[rab-3p::nhr-1(o/e); myo-2p::mCherry strain 1]*	This work
RVM450	*rmIs133[unc-54p::40Q::YFP] X; unc-1(vlt10) X; nhr-1(vlt15) X; vltEx290[rab-3p::nhr-1(o/e); myo-2p::mCherry strain 2]*	This work
SJ4005	*zcIs4[hsp-4::GFP] V*	Calfon et al., 2002
RVM373	*zcIs4[hsp-4::GFP] V; unc-1(vlt10) X*	This work
RVM544	*pkIs2386[unc-54p::α-synuclein::YFP + unc-119(+)] IV; nhr-1(vlt16) X*	This work
RVM545	*pkIs2386[unc-54p::α-synuclein::YFP + unc-119(+)] IV; unc-1(vlt10) X; nhr-1(vlt16) X*	This work

[1]Strains are listed in order of appearance in the results.

Appendix Table S5. Primers used in this work.

PCR product	Primer sequence (5' – 3')	Strategy
attB1::rab-3p<br>*::attB5r*	Frw: **ggggacaagtttgtacaaaaaagcaggct**atcttcagatgggagcagtgg<br>Rev: **ggggacaactttgtatacaaagttgt**catctgaaaatagggctactgtagat	Gateway<br>MultiSite Pro
attB5::unc-1<br>*::attB2*	Frw: gggacaactttgtatacaaaagttgaaatgtcaaacaaggaaagaac<br>Rev: ggggaccactttgtacaagaaagctgggtaaaggaaaatatgattattggtc	Gateway<br>MultiSite Pro
unc-1(n494)	Frw: cttgatgaaagaactgaacat<br>Rev: atgttcagttctttcatcaag	Gateway<br>MultiSite Pro
rab-3p	A: M13F<br>B: catctgaaaatagggctactgtagat	Tissue-specific<br>RNAi PCR #1.1
unc-1<br>*_sense/antise*<br>*nse*<br>overlapping:<br>*rab-3p*	C1: **atctacagtagccctattttcagatg**atgtcaaacaaggaaagaacagag<br>D1: M13R<br>C2: **atctacagtagccctattttcagatg**ttattggtctttttcataaatgctcc<br>D2: M13F<br>A*: atcttcagatgggagcagtgg<br>D1*: aaggaaaatatgattattggtc<br>D2*: aaatgtcaaacaaggaaagaac	Tissue-specific<br>RNAi<br>PCR #1.2<br>*PCR #2.1
AMP^r<br>*_sense/antise*<br>*nse*<br>overlapping:<br>*rab-3p*	C1: **atctacagtagccctattttcagatg**gcatcttacggatggcatgacag<br>D1: acagagttcttgaagtggtggc<br>C2: **atctacagtagccctattttcagatg**cgtttggtatggcttcattcagc<br>D2: gcttacagacaagctgtgaccg<br>A*: atcttcagatgggagcagtgg<br>D1*: cgtttggtatggcttcattcagc<br>D2*: gcatcttacggatggcatgacag	Tissue-specific<br>RNAi<br>PCR #1.2<br>*PCR #2.1
inx-2<br>*_sense/antise*<br>*nse*<br>overlapping:<br>*rab-3p*	C1: **atctacagtagccctattttcagatg**gtaccacttgtccttcttttccaag<br>D1: gtcaaggaaatgtttcagaagaacc<br>C2: **atctacagtagccctattttcagatg**gtcaaggaaatgtttcagaagaacc<br>D2: gtaccacttgtccttcttttccaag<br>A*: atcttcagatgggagcagtgg<br>D1*: atttgcagaatgcaattgacaa<br>D2*: ctgccatgtttgtgctccctta	Tissue-specific<br>RNAi<br>PCR #1.2<br>*PCR #2.1
inx-6<br>*_sense/antise*<br>*nse*<br>overlapping:<br>*rab-3p*	C1: **atctacagtagccctattttcagatg**gtcaactttgtgaaccagtact<br>D1: aatgtatacagatggacgtcct<br>C2: **atctacagtagccctattttcagatg**aatgtatacagatggacgtcct<br>D2: gtcaactttgtgaaccagtact<br>A*: atcttcagatgggagcagtgg<br>D1*: tcgaaagctgctagccgagttttg<br>D2*: tgttccactcgaccagcaactagc	Tissue-specific<br>RNAi<br>PCR #1.2<br>*PCR #2.1
unc-7<br>*_sense/antise*<br>*nse*<br>overlapping:<br>*rab-3p*	C1: **atctacagtagccctattttcagatg**ctttgagcactcaaagaaaaactct<br>D1: tcagtctatcgtcccttgaccgtgt<br>C2: **atctacagtagccctattttcagatg**tcagtctatcgtcccttgaccgtgt<br>D2: ctttgagcactcaaagaaaaactct<br>A*: atcttcagatgggagcagtgg<br>D1*: tccgcatcccaaaattgcgtcgg<br>D2*: actataattcaaaagcaacctaatg	Tissue-specific<br>RNAi<br>PCR #1.2<br>*PCR #2.1
trx-1p	A: aaccaattgagttggcacttcg<br>B: aaccttggtgagagacatgatg	Tissue-specific<br>RNAi PCR #1.1
ssu-1<br>*_sense/antise*<br>*nse*<br>overlapping:	C1: **catcatgtctctcaccaaggtt**ccagagctctgtgtgcaatcgc<br>D1: catgtttcgcgtatttttctgc<br>C2: **catcatgtctctcaccaaggtt**ccttatacacagcattttcc<br>D2: cgtggcgggacccaaaatctc	Tissue-specific<br>RNAi<br>PCR #1.2<br>*PCR #2.1

trx-1p	A*: agaatggatacctgatcatt<br>D1*: ccttatacacagcattttcc<br>D2*: ccagagctctgtgtgcaatcgc	
AMP^r _sense/antisense overlapping: *trx-1p*	C1: **catcatgtctctcaccaaggtt**gcatcttacggatggcatgacag<br>D1: acagagttcttgaagtggtggc<br>C2: **catcatgtctctcaccaaggtt**cgtttggtatggcttcattcagc<br>D2: gcttacagacaagctgtgaccg<br>A*: agaatggatacctgatcatt<br>D1*: cgtttggtatggcttcattcagc<br>D2*: gcatcttacggatggcatgacag	Tissue-specific RNAi<br>PCR #1.2<br>*PCR #2.1
unc-1 _sense/antisense overlapping: *trx-1p*	C1: **catcatgtctctcaccaaggtt**atgtcaaacaaggaaagaacagag<br>D1: M13R<br>C2: **catcatgtctctcaccaaggtt**ttattggtctttttcataaatgctcc<br>D2: M13F<br>A*: agaatggatacctgatcatt<br>D1*: aaggaaaatatgattattggtc<br>D2*: aaatgtcaaacaaggaaagaac	Tissue-specific RNAi<br>PCR #1.2<br>*PCR #2.1
oig-1p	A: agagcaagcagtcagtgaaaatgt<br>B: agtcgaacgttttgcagaattatg	Tissue-specific RNAi PCR #1.1
osm-3p	A: gcttaaaatccggctaaaattca<br>B: tccgacgcatagctggaaattttg	Tissue-specific RNAi PCR #1.1
glr-1p	A: aacaagaaagtcgtagttgttac<br>B: tgtgaatgtgtcagattgggtgcc	Tissue-specific RNAi PCR #1.1
gpa-9p	A: gatggtcccggaaaacatcatcg<br>B: cccattgcatatttcattaaac	Tissue-specific RNAi PCR #1.1
unc-1 _sense overlapping: *oig-1p*	C1: **cataattctgcaaaacgttcgact**atgtcaaacaaggaaagaacagag<br>D1: M13R<br>A*: aacatgttttggagcatatttcgcg<br>D1*: aaggaaaatatgattattggtc	Tissue-specific RNAi<br>PCR #1.2<br>*PCR #2.1
unc-1 _antisense overlapping: *osm-3p*	C2: **caaaatttccagctatgcgtcgga**ttattggtctttttcataaatgctcc<br>D2: M13F<br>A*: aattaaatttgcctgaaaatccg<br>D2*: aaatgtcaaacaaggaaagaac	Tissue-specific RNAi<br>PCR #1.2<br>*PCR #2.1
unc-1 _sense overlapping: *glr-1p*	C1:**ggcacccaatctgacacattcaca**atgtcaaacaaggaaagaacagag<br>D1: M13R<br>A*: aataattataagagacgtgtag<br>D1*: aaggaaaatatgattattggtc	Tissue-specific RNAi<br>PCR #1.2<br>*PCR #2.1
unc-1 _antisense overlapping: *gpa-9p*	C2: **gtttaatgaaatatgcaatgggtt**attggtctttttcataaatgctcc<br>D2: M13F<br>A*: accgaatcaaaatatctgaat<br>D2*: aaatgtcaaacaaggaaagaac	Tissue-specific RNAi<br>PCR #1.2<br>*PCR #2.1
AMP^r _sense overlapping: *oig-1p*	C1: **cataattctgcaaaacgttcgact**gcatcttacggatggcatgacag<br>D1: acagagttcttgaagtggtggc<br>A*: aacatgttttggagcatatttcgcg<br>D1*: cgtttggtatggcttcattcagc	Tissue-specific RNAi<br>PCR #1.2<br>*PCR #2.1
AMP^r _antisense overlapping: *osm-3p*	C2: **caaaatttccagctatgcgtcgga**cgtttggtatggcttcattcagc<br>D2: gcttacagacaagctgtgaccg<br>A*: aattaaatttgcctgaaaatccg<br>D2*: gcatcttacggatggcatgacag	Tissue-specific RNAi<br>PCR #1.2<br>*PCR #2.1
AMP^r _sense overlapping:	C1: **ggcacccaatctgacacattcaca**gcatcttacggatggcatgacag<br>D1: acagagttcttgaagtggtggc<br>A*: aataattataagagacgtgtag	Tissue-specific RNAi<br>PCR #1.2

glr-1p	D1*: cgtttggtatggcttcattcagc	*PCR #2.1
AMP[r]<br>*_antisense*<br>overlapping:<br>*gpa-9p*	C2: **gtttaatgaaatatgcaatggg**cgtttggtatggcttcattcagc<br>D2: gcttacagacaagctgtgaccg<br>A*: accgaatcaaaatatctgaat<br>D2*: gcatcttacggatggcatgacag	Tissue-specific RNAi<br>PCR #1.2<br>*PCR #2.1
unc-1(cDNA)<br>*::unc-54t*<br>overlapping:<br>*oig-1p*	C1:**cataattctgcaaaacgttcgact**atgtcaaacaaggaaagaacagag<br>D1: aaacagttatgtttggtatattg<br>A*: aacatgttttggagcatatttcgcg<br>D1*: aatgtattctgtcatttaaggc	Tissue-specific Rescue<br>PCR #1.2<br>*PCR #2.1
unc-1(cDNA)<br>*::unc-54t*<br>overlapping:<br>*osm-3p*	C1:**caaaatttccagctatgcgtcgga**atgtcaaacaaggaaagaacagag<br>D1: aaacagttatgtttggtatattg<br>A*: aattaaatttgcctgaaaatccg<br>D1*: aatgtattctgtcatttaaggc	Tissue-specific Rescue<br>PCR #1.2<br>*PCR #2.1
sul-1	Frw: ttcagctctggaaatgctactac<br>Rev: tctttttgtacttcaagtagtgc	Feeding RNAi
sul-2	Frw: gctcacctagcagagctggattc<br>Rev: gtctctcgtgttgatcttgtgcc	Feeding RNAi
sul-3	Frw: ttacatacaccaaacctccggc<br>Rev: ttgaagtaacccgtttgtggtcc	Feeding RNAi
elo-2	Frw: tcatgcactggtatcatcatgcc<br>Rev: gcgcaaccaggaacagaatcag	Feeding RNAi
fat-6	Frw: cgtaagcatccacaagttaagg<br>Rev: ggtaattgaggaatcgtatggc	Feeding RNAi
skn-1	Frw: gatcgcgagagttgttccactgg<br>Rev: ggctttaataaggtttcgaccgag	Feeding RNAi

Appendix References

Altun ZF, Chen B, Wang Z-W & Hall DH (2009) High resolution map of *Caenorhabditis elegans* gap junction proteins. *Dev Dyn* 238: 1936–1950

Brenner S (1974) The genetics of Caenorhabditis elegans. *Genetics* 77: 71–94

Brignull HR, Moore FE, Tang SJ & Morimoto RI (2006) Polyglutamine Proteins at the Pathogenic Threshold Display Neuron-Specific Aggregation in a Pan-Neuronal Caenorhabditis elegans Model. *Journal of Neuroscience* 26: 7597–7606

Calfon M, Zeng H, Urano F, Till JH, Hubbard SR, Harding HP, Clark SG & Ron D (2002) IRE1 couples endoplasmic reticulum load to secretory capacity by processing the XBP-1 mRNA. *Nature* 415: 92–96

Carroll BT, Dubyak GR, Sedensky MM & Morgan PG (2006) Sulfated Signal from ASJ Sensory Neurons Modulates Stomatin-dependent Coordination in *Caenorhabditis elegans*. *J Biol Chem* 281: 35989–35996

Chalfie M & Sulston J (1981) Developmental genetics of the mechanosensory neurons of Caenorhabditis elegans. *Developmental Biology* 82: 358–370

Chernomor O, von Haeseler A & Minh BQ (2016) Terrace Aware Data Structure for Phylogenomic Inference from Supermatrices. *Syst Biol* 65: 997–1008

Cingolani P, Platts A, Wang LL, Coon M, Nguyen T, Wang L, Land SJ, Lu X & Ruden DM (2012) A program for annotating and predicting the effects of single nucleotide polymorphisms, SnpEff: SNPs in the genome of Drosophila melanogaster strain w1118; iso-2; iso-3. *Fly (Austin)* 6: 80–92

Garrison E & Marth G (2012) Haplotype-based variant detection from short-read sequencing. *arXiv:12073907 [q-bio]*

van Ham TJ, Thijssen KL, Breitling R, Hofstra RMW, Plasterk RHA & Nollen EAA (2008) C. elegans Model Identifies Genetic Modifiers of α-Synuclein Inclusion Formation During Aging. *PLoS Genet* 4: e1000027

Lapatsina L, Brand J, Poole K, Daumke O & Lewin GR (2012) Stomatin-domain proteins. *European Journal of Cell Biology* 91: 240–245

Li H (2011) Improving SNP discovery by base alignment quality. *Bioinformatics* 27: 1157–1158

Li H (2013) Aligning sequence reads, clone sequences and assembly contigs with BWA-MEM. *arXiv:13033997 [q-bio]*

Li S, Dent JA & Roy R (2003) Regulation of Intermuscular Electrical Coupling by the *Caenorhabditis elegans* Innexin *inx-6*. *MBoC* 14: 2630–2644

Link CD (1995) Expression of human beta-amyloid peptide in transgenic Caenorhabditis elegans. *Proceedings of the National Academy of Sciences* 92: 9368–9372

Morley JF, Brignull HR, Weyers JJ & Morimoto RI (2002) The threshold for polyglutamine-expansion protein aggregation and cellular toxicity is dynamic and influenced by aging in Caenorhabditis elegans. *Proceedings of the National Academy of Sciences* 99: 10417–10422

Nguyen L-T, Schmidt HA, von Haeseler A & Minh BQ (2015) IQ-TREE: A Fast and Effective Stochastic Algorithm for Estimating Maximum-Likelihood Phylogenies. *Molecular Biology and Evolution* 32: 268–274

Park EC & Horvitz HR (1986) Mutations with dominant effects on the behavior and morphology of the nematode Caenorhabditis elegans. *Genetics* 113: 821–852

Rajaram S, Sedensky MM & Morgan PG (1998) unc-1: A stomatin homologue controls sensitivity to volatile anesthetics in Caenorhabditis elegans. *Proceedings of the National Academy of Sciences* 95:

8761–8766
Rottiers V, Motola DL, Gerisch B, Cummins CL, Nishiwaki K, Mangelsdorf DJ & Antebi A (2006) Hormonal Control of C. elegans Dauer Formation and Life Span by a Rieske-like Oxygenase. *Developmental Cell* 10: 473–482
Sedensky M & Meneely P (1987) Genetic analysis of halothane sensitivity in Caenorhabditis elegans. *Science* 236: 952–954
The C. elegans Deletion Mutant Consortium (2012) Large-Scale Screening for Targeted Knockouts in the *Caenorhabditis elegans* Genome. *G3* 2: 1415–1425
