# Supplementary material for: Changes in lipid metabolism driven by steroid signalling modulate proteostasis in C. elegans
Source: EMBO Rep. 2023 Apr 27;24(6):e55556. doi: 10.15252/embr.202255556 (PMC10240203; doi:10.15252/embr.202255556)
Supplement: Supplementary file 2 — Expanded View Figures PDF [file EMBR-24-e55556-s009.pdf]

Expanded View Figures

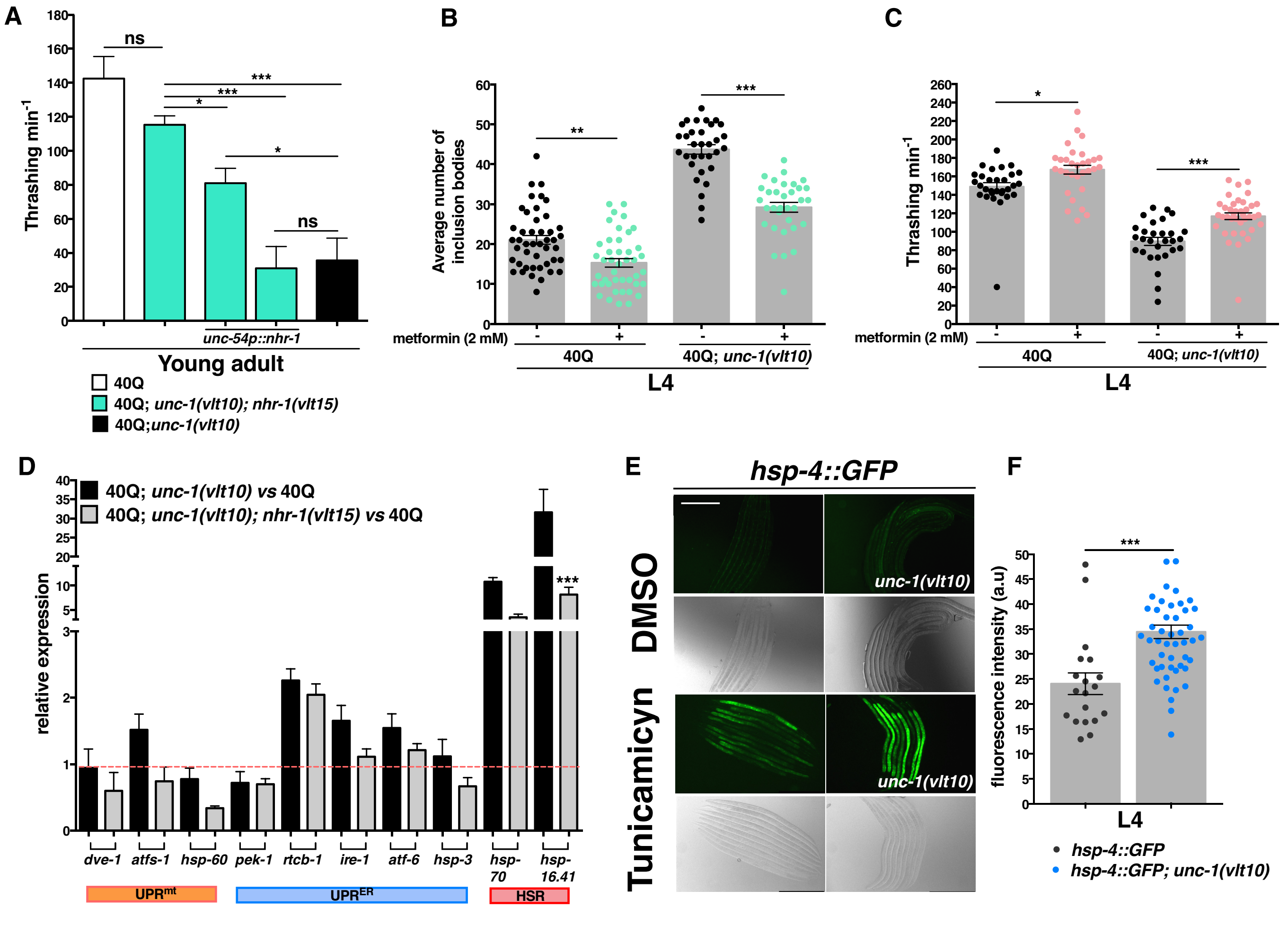

Figure EV1. Ablating *nhr-1* improves movement and health span and reduces *unc-1(vlt10)*-associated stress.

A Motility measurement after the muscle-specific restoration of NHR-1 in *unc-1(vlt10); nhr-1(vlt16)* young adults compared to non-rescued double mutants.

B, C The effects of metformin treatment in *unc-1* mutants as measured by the average number of polyQ inclusion bodies in muscle cells (B) and motility (C).

D The average expression levels of several genes related to the UPR in the endoplasmic reticulum, cytosol and mitochondria in 40Q; *unc-1(vlt10)* and 40Q; *unc-1 (vlt10); nhr-1(vlt15)* mutants as compared to 40Q (discontinuous red line).

E Representative images from wild type and *unc-1(vlt10)* animals expressing the *hsp-4::GFP* transgene, which induces stress in the endoplasmic reticulum. The expression of this transgene is activated by a mild treatment with tunicamycin (1 μg/ml). scale bar: 250 μm.

F Measured fluorescence intensity of the animals imaged in (E).

Data information: All plotted data show the mean ± standard error of the mean (SEM). At least 15 worms were analysed for thrashing assay and more than 30 animals were analysed for scoring inclusion bodies. UPR-stress gene expression was evaluated from three biological replicates and ER-stress reporter intensity was measured in more than 40 worms. *$P < 0.05$; **$P < 0.01$; ***$P < 0.001$; ns: not significant, as calculated using the one-way ANOVA with *post-hoc* Tukey test (graphs A–D) and the Mann–Whitney U-test (graph F).
Source data are available online for this figure.

© 2023 The Authors

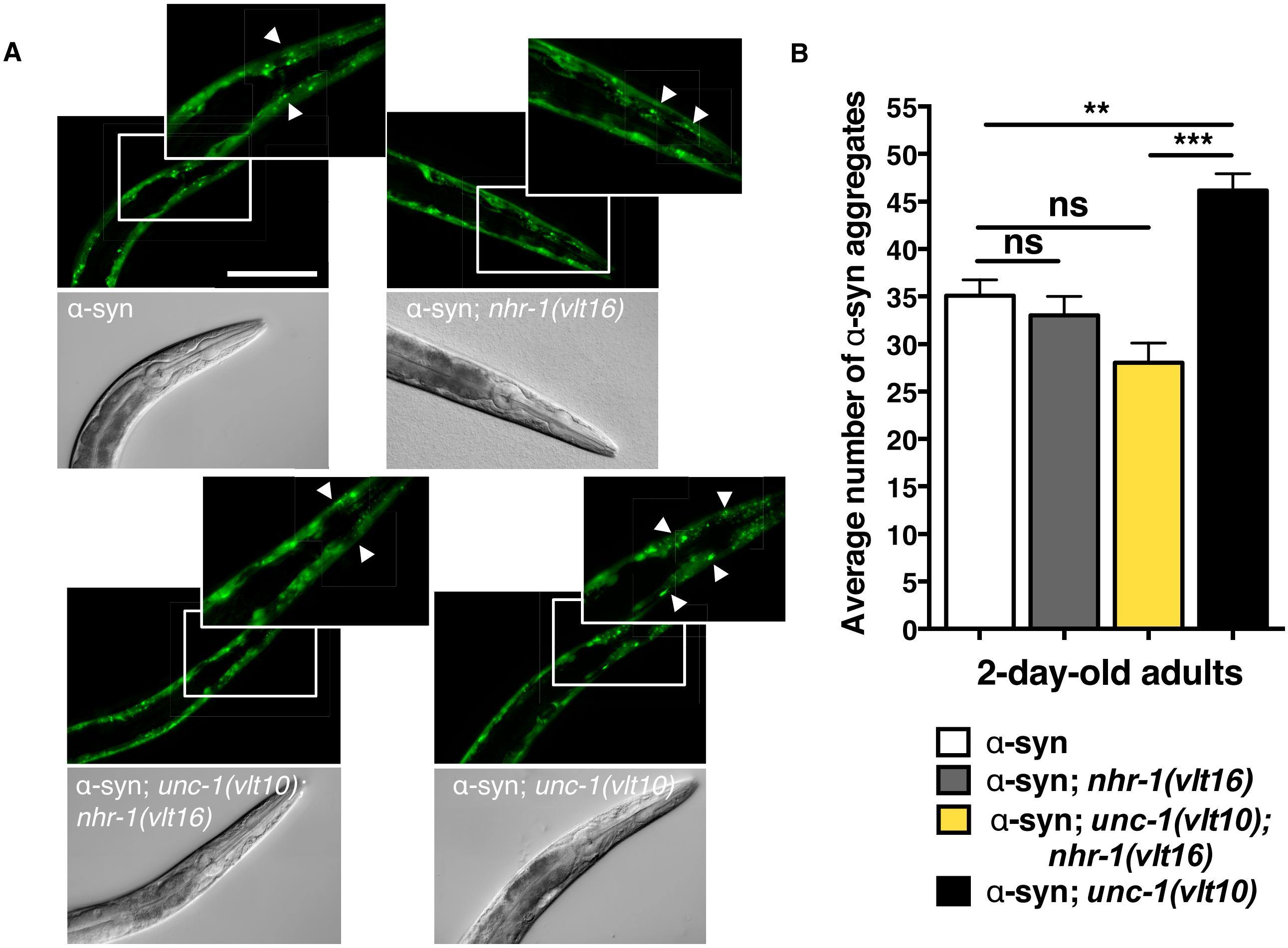

Figure EV2. NHR-1 modulates α-synuclein aggregation.

A Representative images of muscle pharyngeal cells of mutants containing *vlt10* and *vlt16* alleles in a α-synuclein background. Magnified insets show the two pharyngeal bulbs from which the α-synuclein aggregates (white arrows) were measured. Scale bar: 100 μm.
B The mean number of α-synuclein aggregates in *unc-1* animals bearing *nhr-1* loss of function alleles *vlt15* and *vlt16*.

Data information: The plotted data show the mean ± standard error of the mean (SEM). At least 30 animals were analysed in a three independent experiments. $^{**}P < 0.01$; $^{***}P < 0.001$; ns: not significant, as calculated using the one-way ANOVA with *post-hoc* Tukey test.
Source data are available online for this figure.

© 2023 The Authors

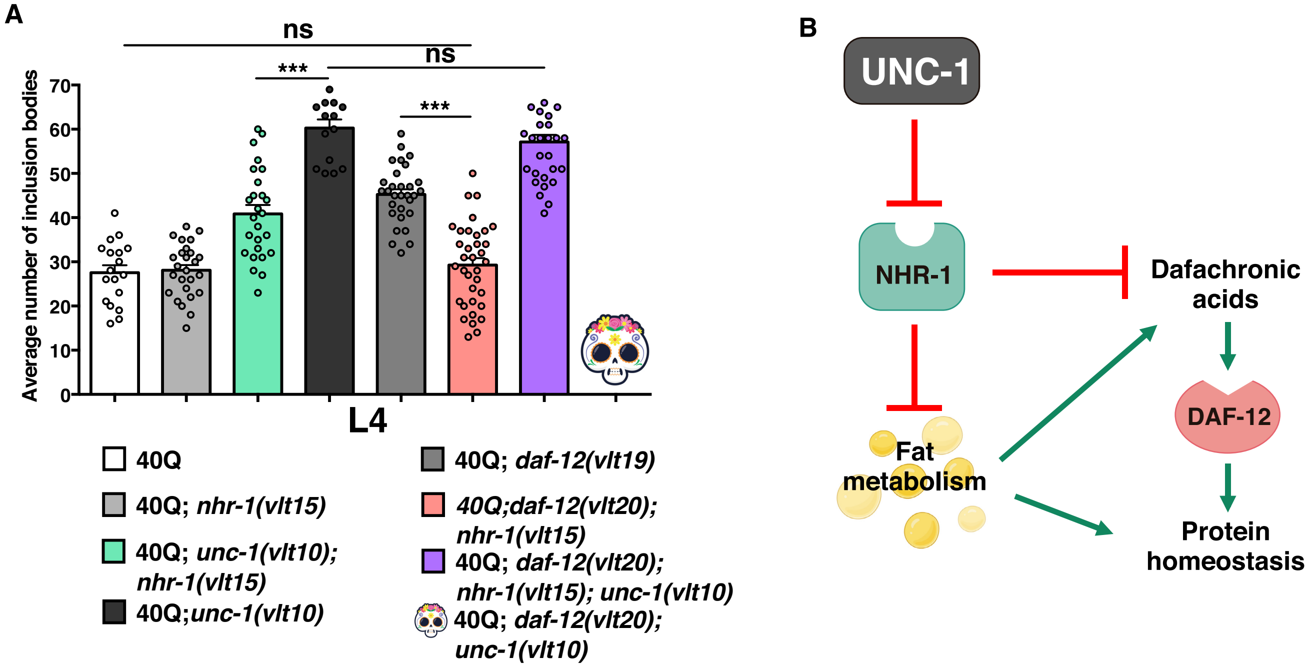

Figure EV3. NHR-1 and DAF-12 antagonization regulates protein homeostasis.

A The average number of polyQ inclusion bodies in muscle cells after *nhr-1* ablation in *daf-12* and *daf-12; unc-1* double mutants.
B A diagram showing the relationship between NHR-1 and DAF-12 in modulating protein homeostasis through fat metabolism changes and steroid hormonal signalling. The activation of NHR-1 represses the expression of genes involved in fat metabolism and dafachronic acid synthesis.

Data information: The plotted data show the mean ± standard error of the mean (SEM). At least 20 animals were analysed in a three independent experiments. ***$P < 0.001$; ns: not significant, as calculated using the one-way ANOVA with *post-hoc* Tukey test.
Source data are available online for this figure.

Figure EV4. Ablation of *unc-1* modifies downregulated and upregulated genes in *ssu-1*.

A Relative expression levels of genes that are not expressed in *ssu-1(fc73)* mutants or are downregulated but are upregulated in *unc-1(vlt10)* mutants.
B Genes that are upregulated in *ssu-1(fc73)* but are downregulated in *unc-1(vlt10)* mutants.

Data information: The plotted data show the mean ± standard error of the mean (SEM). FPKM values are from the transcriptomic analysis ($N = 6$ for each mutant strain) in Fig 5. *$P < 0.05$; **$P < 0.01$; ns: not significant. The statistical analysis was done using a Mann–Whitney *U*-test.
Source data are available online for this figure.

▶

© 2023 The Authors

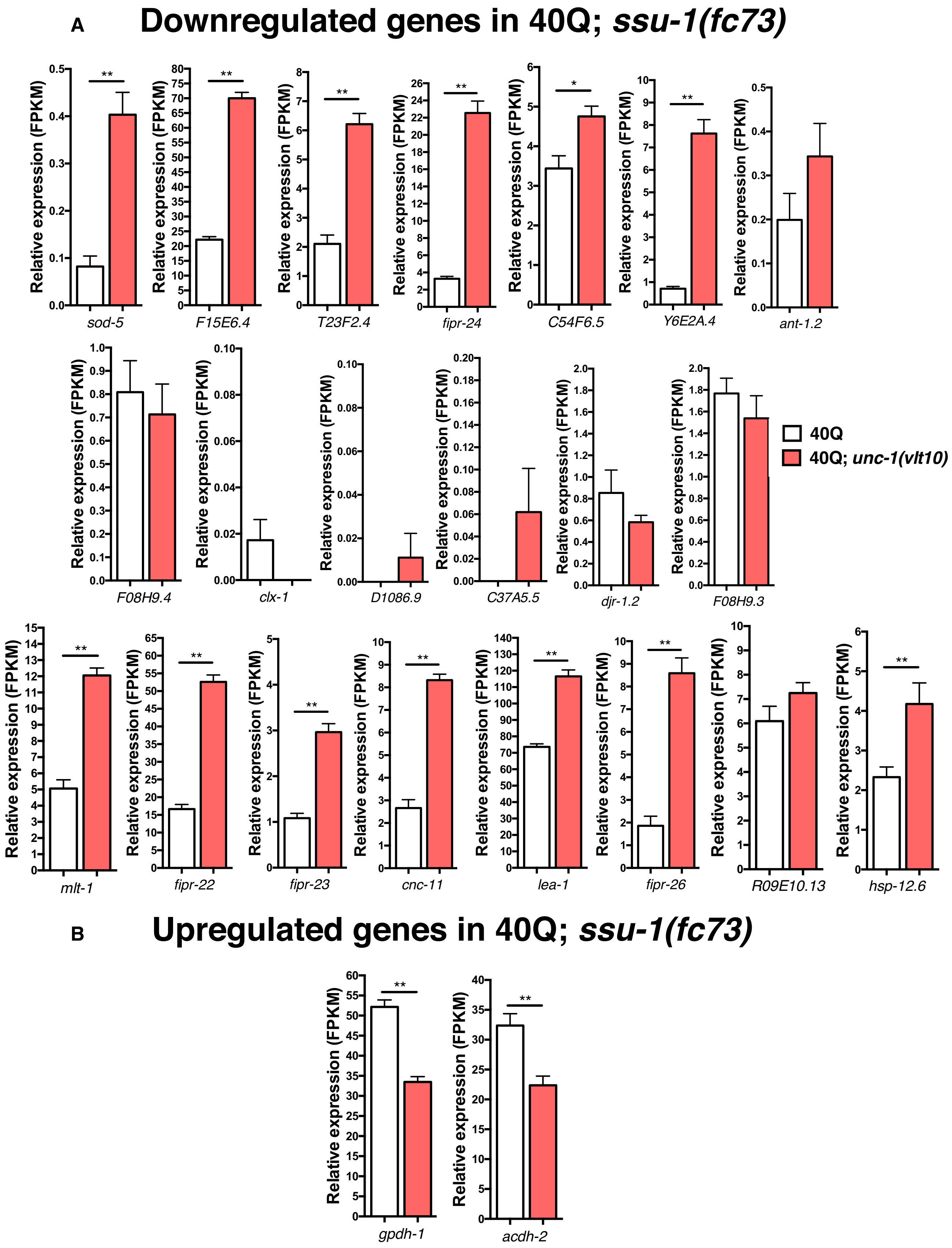

Figure EV4.

 © 2023 The Authors

	unc-1(vlt10)	Wild type
Phospatidylcholines	55.8%	56.2%
Phosphatidyletanolamines	11.2%	12.5%
Monoacylglycerols*	8.4%	4.3%
Triglycerides*	6.6%	10.3%
Fatty acids	5.7%	5.5%
Lysophosphatidylcholines	3.8%	3.7%
Carnitines	2.2%	1.6%
Lysophosphatidylethanolamines	1.5%	1.3%
Sphingomyelins	0.9%	1.0%
Phosphoinositides	0.9%	0.6%
Phosphatidylglycerols	0.8%	0.4%
Ceramides	0.5%	1.5%
Sphingosine	0.4%	0.1%
Bile acids	0.4%	0.3%
Phosphatidylserines	0.4%	0.4%
Lysophosphatidylinositols	0.3%	0.4%
Diacylglycerols *	0.1%	0.1%

* Neutral Lipids

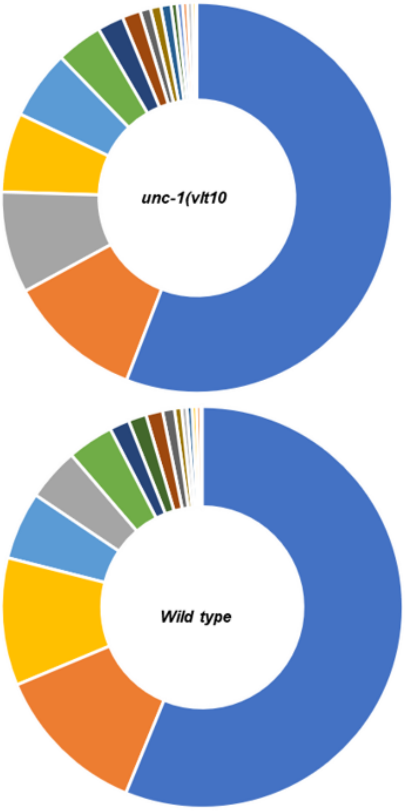

Figure EV5. Lipid abundance in *unc-1* mutants.

List of lipid classes detected in lipidomic assay in wild type and *unc-1(vlt10)* mutants. Asterisks show neutral lipids.
Data information: At least 12 worm biological samples containing more than 2000 animals per sample were analysed.

Source data are available online for this figure.
