# Supplementary material for: Changes in lipid metabolism driven by steroid signalling modulate proteostasis in C. elegans
Source: EMBO Rep. 2023 Apr 27;24(6):e55556. doi: 10.15252/embr.202255556 (PMC10240203; doi:10.15252/embr.202255556)
Supplement: Supplementary file 3 — Source Data for Expanded View and Appendix [file EMBR-24-e55556-s011.zip › EV_and_Appendix_Source_Data/Appendix_Figure_Source_Data/Appendix_FigureS1/Manuscript-EMBOR-2022-55556V3_SourceDataForFigureApp1C.docx]

WB_1: *vlt10* and *vlt16* alleles: Representative images to EV1C.

anti-actin (1:500) anti-polyQ (1:1000)


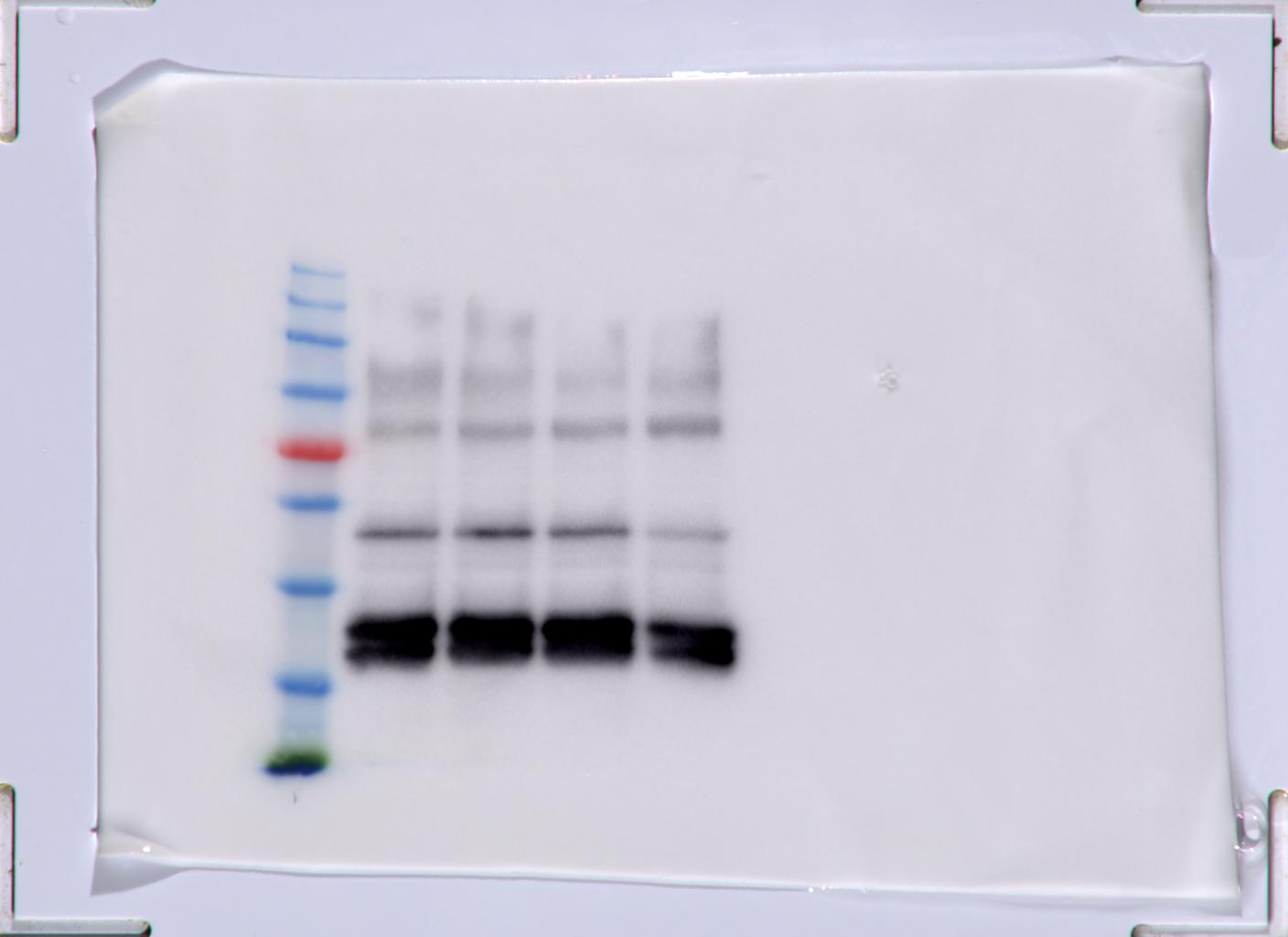


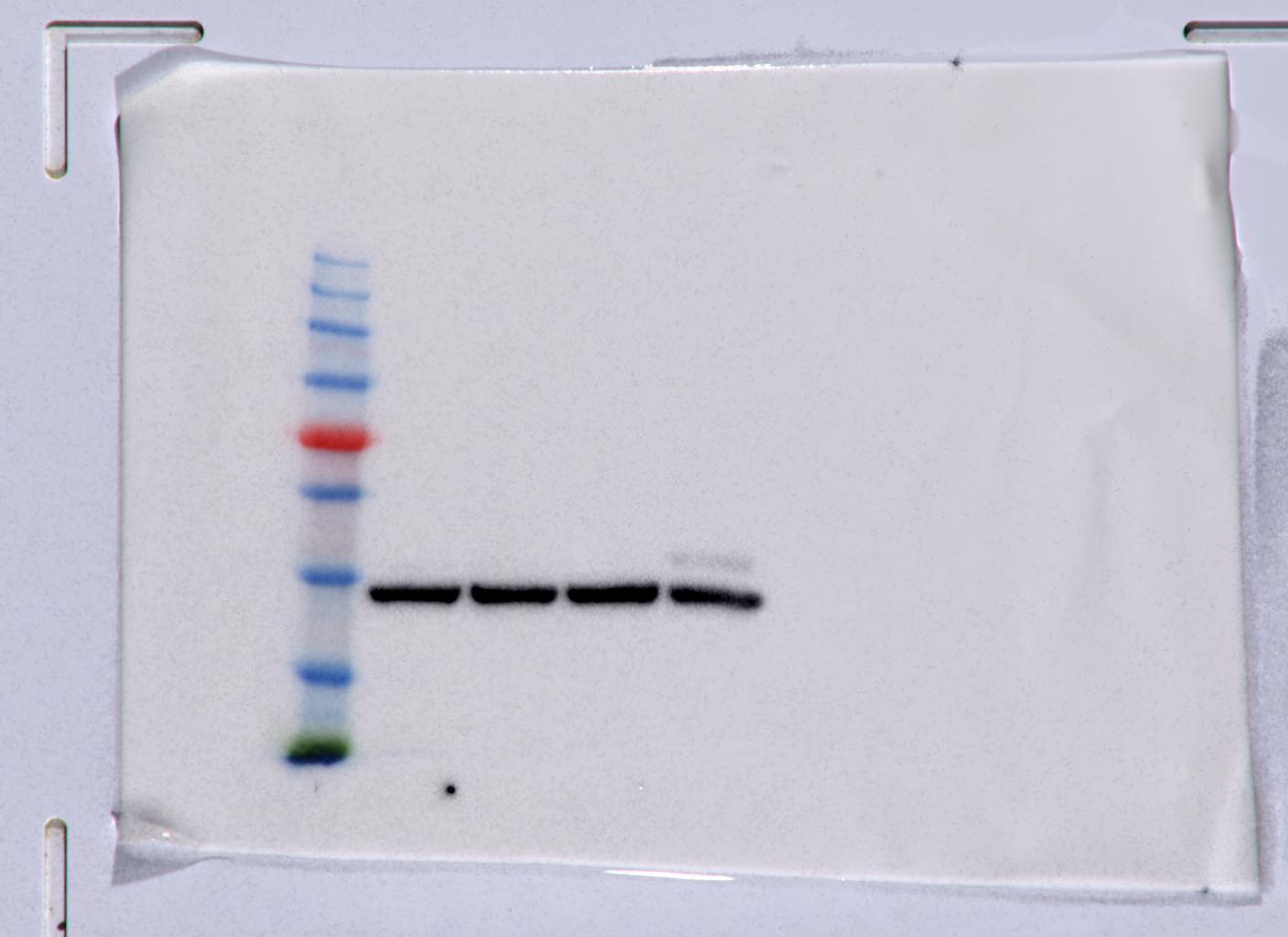
Lane 1: 40Q

Lane 2: 40Q; *unc-1(vlt10)*

Lane 3: 40Q; *nhr-1(vlt16)*

Lane 4: 40Q; *unc-1(vlt10); nhr-1(vlt16)*

WB_2: *vlt10* and *vlt16* alleles

anti-actin (1:500) anti-polyQ (1:1000)

**
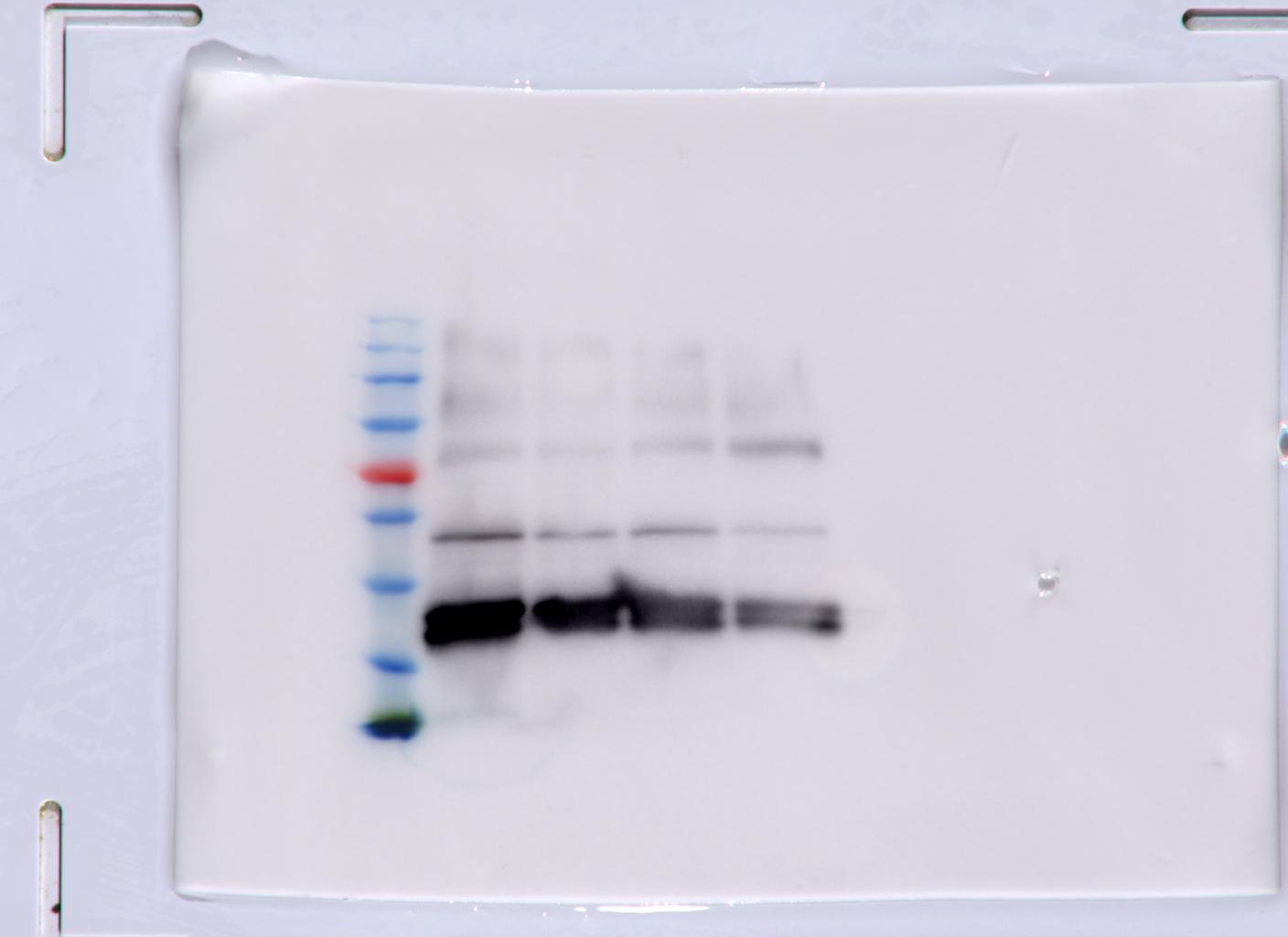
**

**
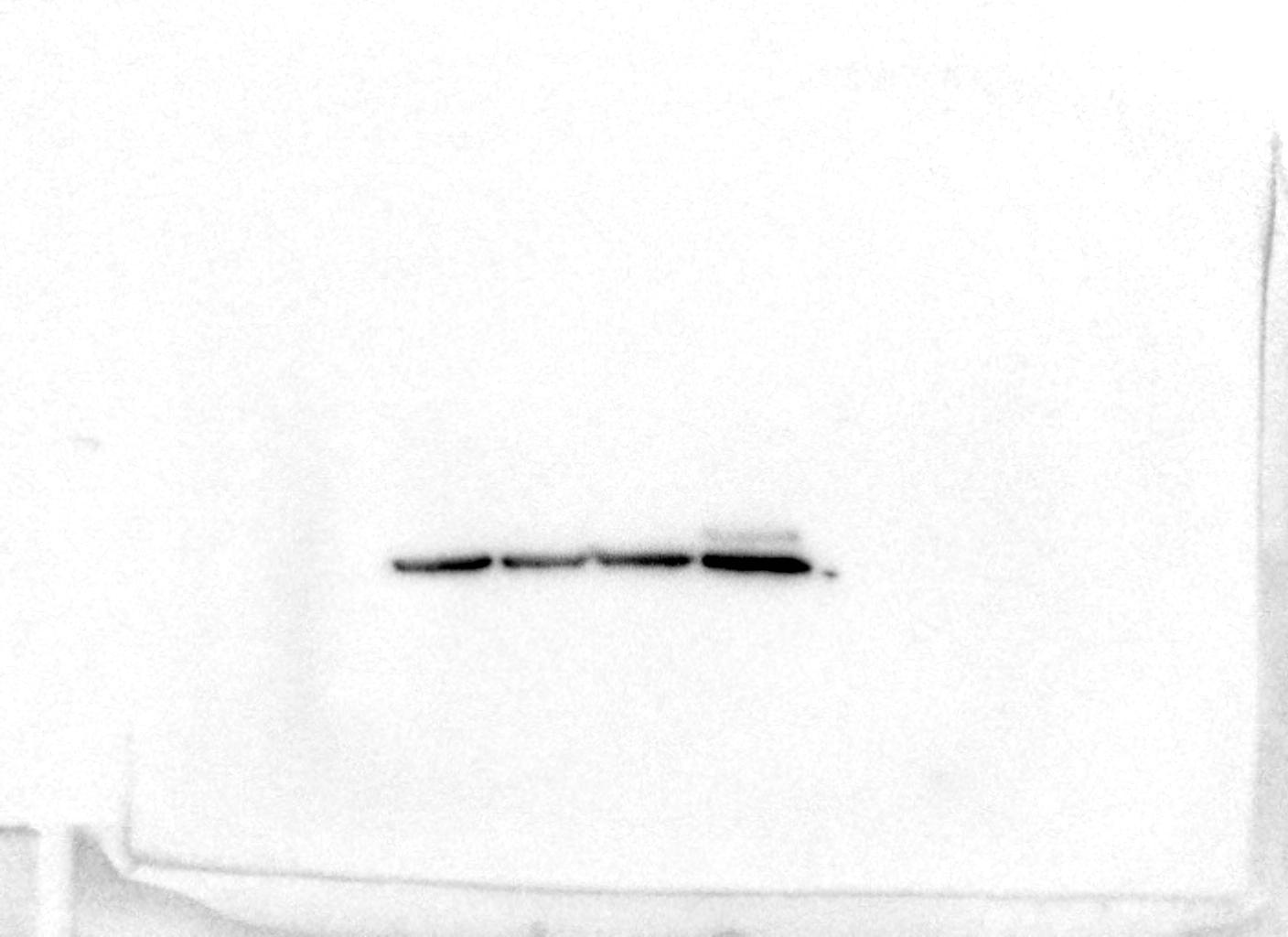
**Lane 1: 40Q

Lane 2: 40Q; *unc-1(vlt10)*

Lane 3: 40Q; *nhr-1(vlt16)*

Lane 4: 40Q; *unc-1(vlt10); nhr-1(vlt16)*

WB_3: *vlt10* and *vlt16* alleles

anti-actin (1:500) anti-polyQ (1:1000)


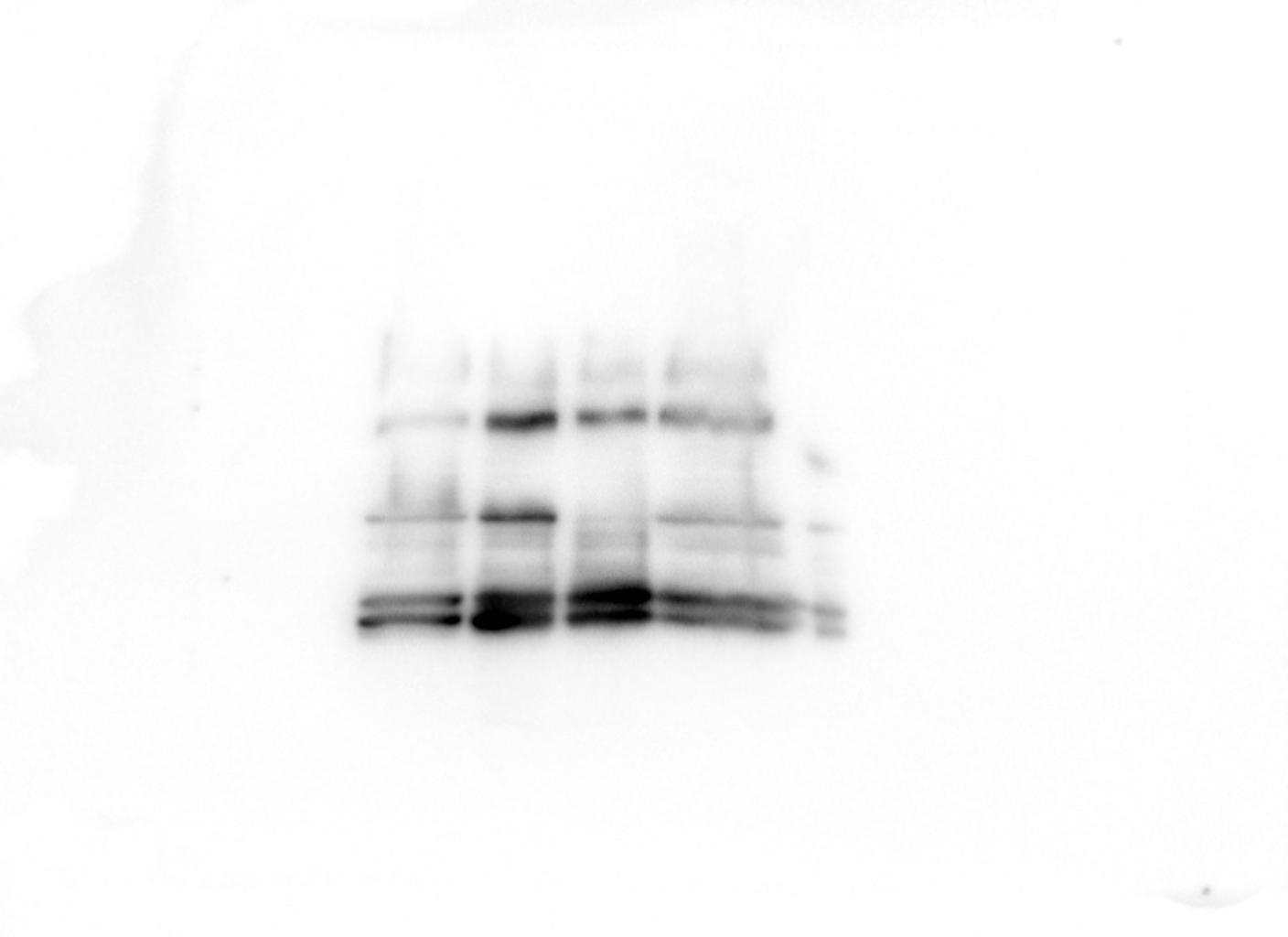

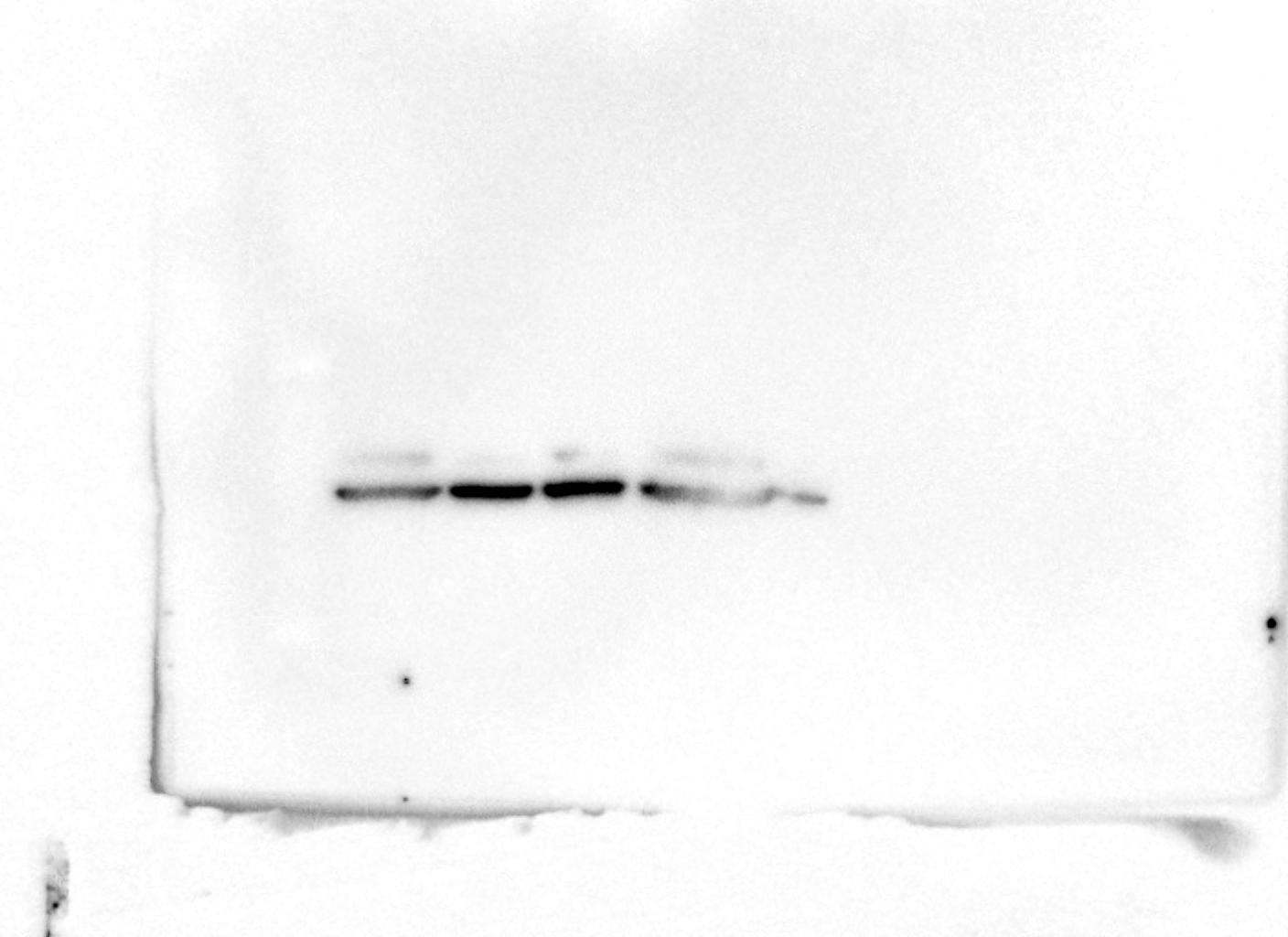


Lane 1: 40Q

Lane 2: 40Q; *unc-1(vlt10)*

Lane 3: 40Q; *nhr-1(vlt16)*

Lane 4: 40Q; *unc-1(vlt10); nhr-1(vlt16)*

WB_1: *e1598* and *e719* alleles. Representative images to EV1C.

anti-actin (1:500) anti-polyQ (1:1000)


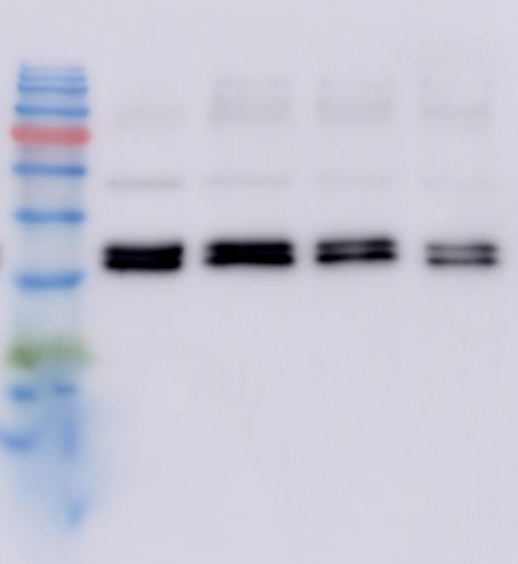

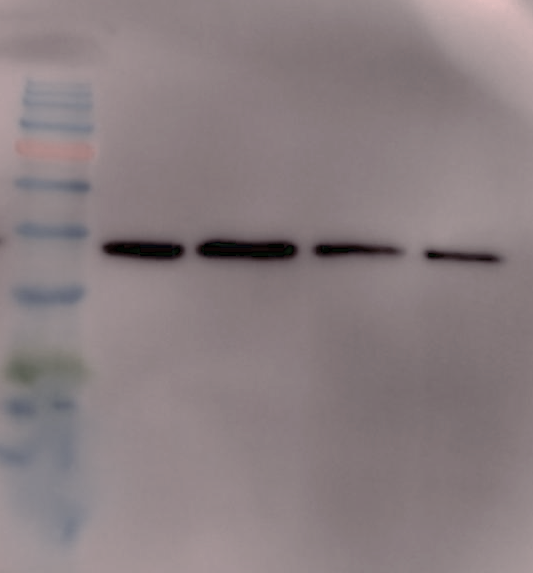


Lane 1: 40Q

Lane 2: 40Q; *unc-1(e1598)*

Lane 3: 40Q; *unc-1(e719)*

WB_2: *e1598* and *e719* alleles.

anti-actin (1:500) anti-polyQ (1:1000)


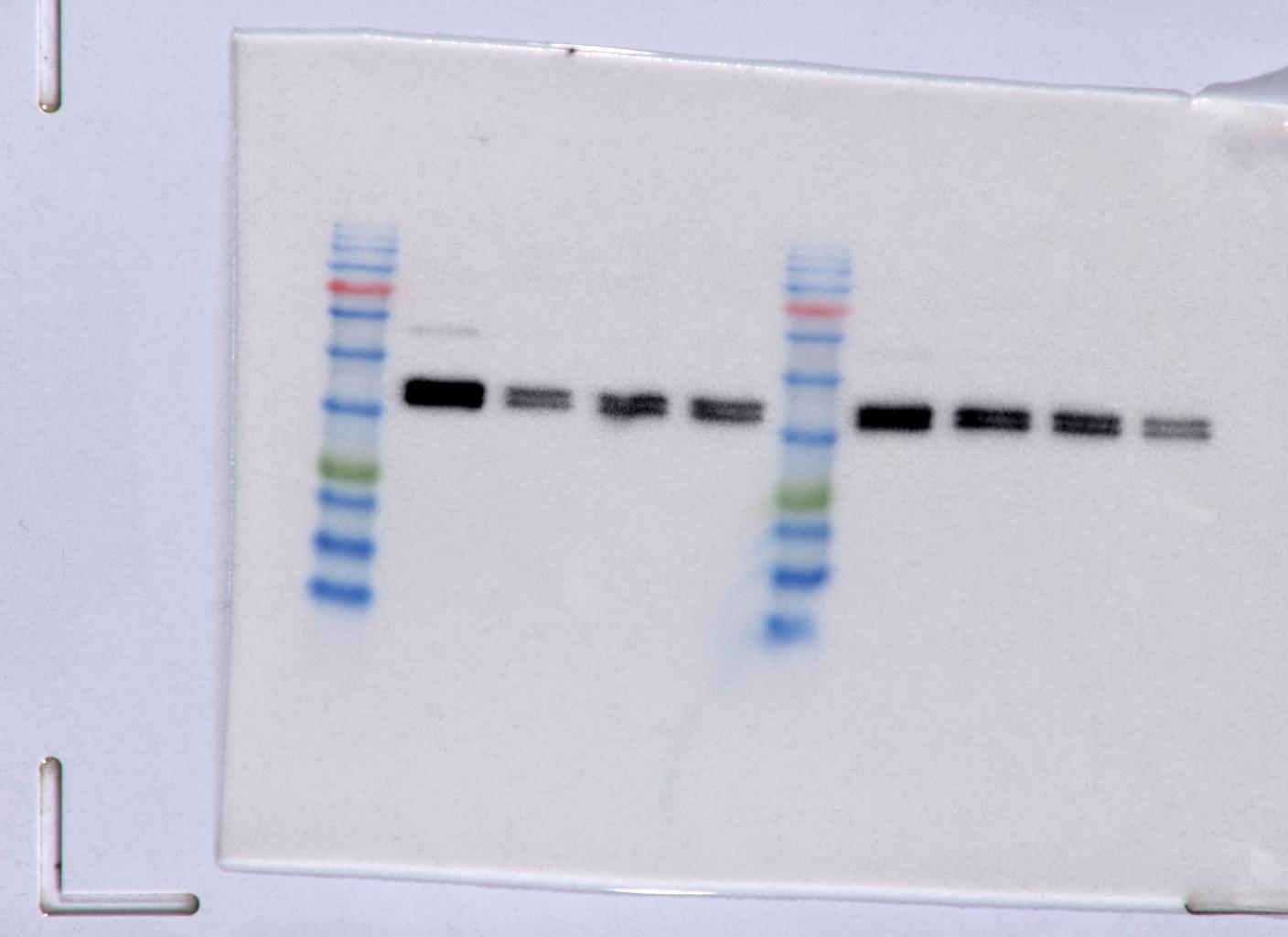


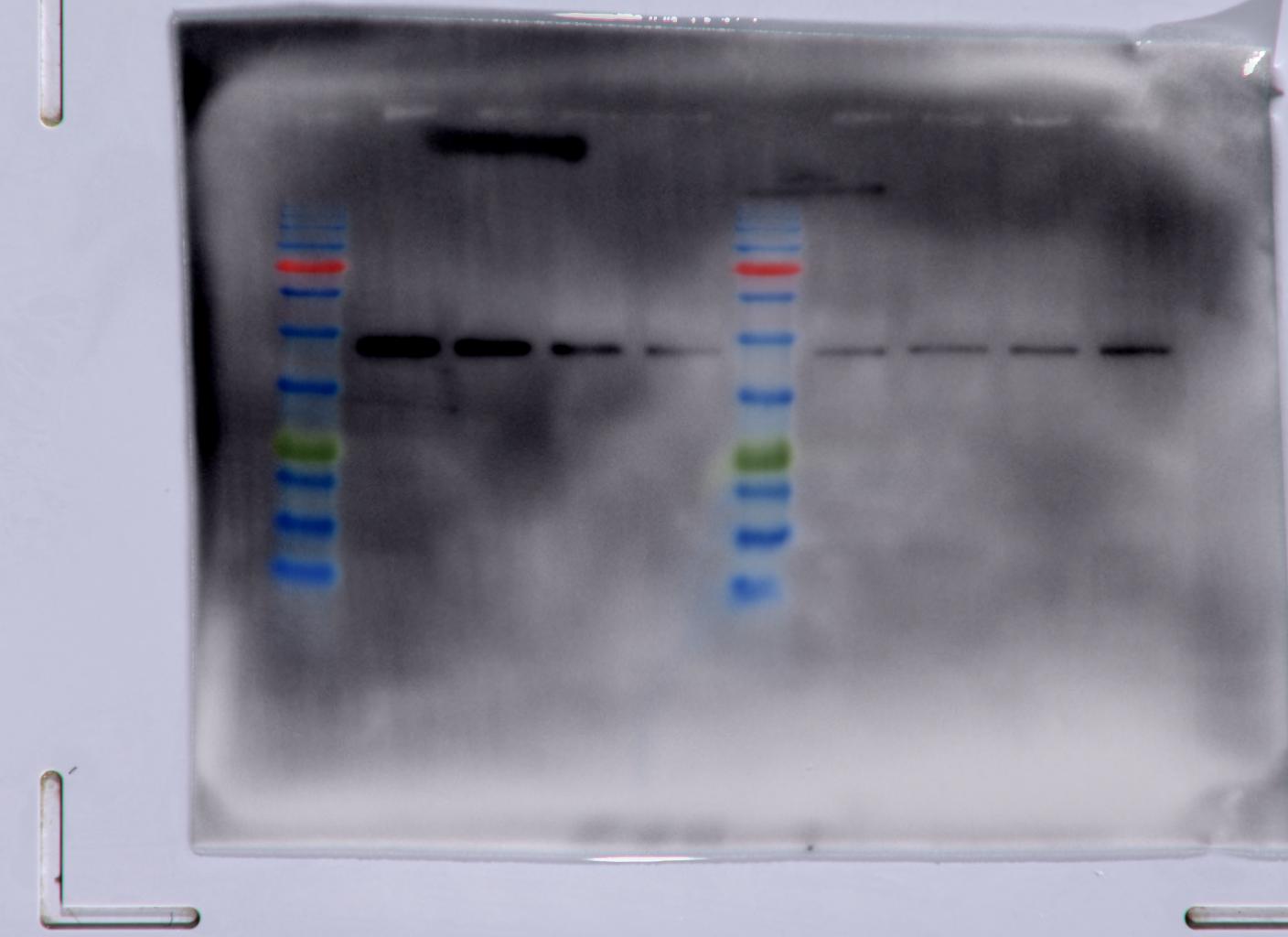


Lane 1: 40Q

Lane 2: 40Q; *unc-1(e1598)*

Lane 3: 40Q; *unc-1(e719)*

WB_3: *e1598* and *e719* alleles.


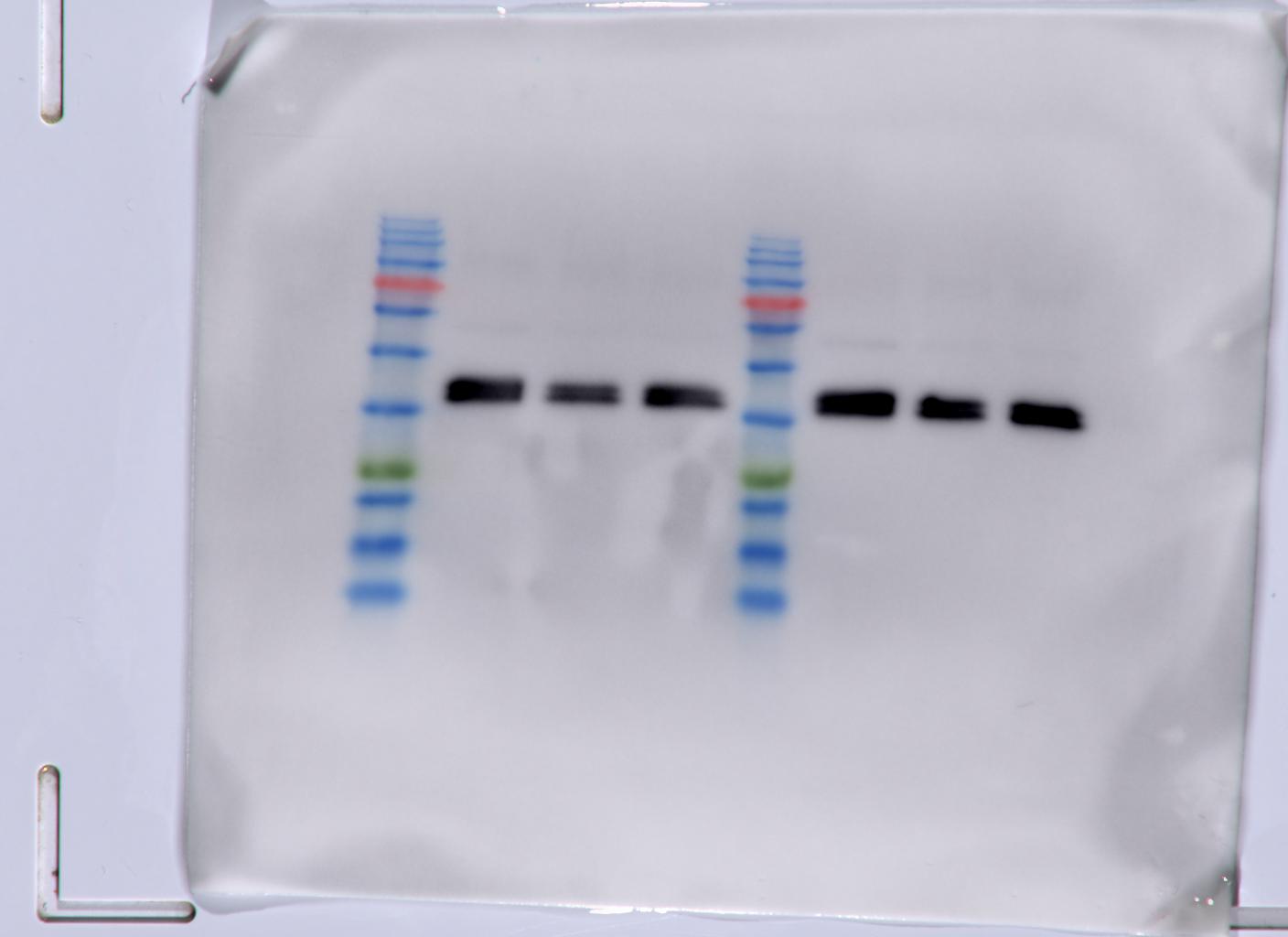
 anti-actin (1:500) anti-polyQ (1:1000)


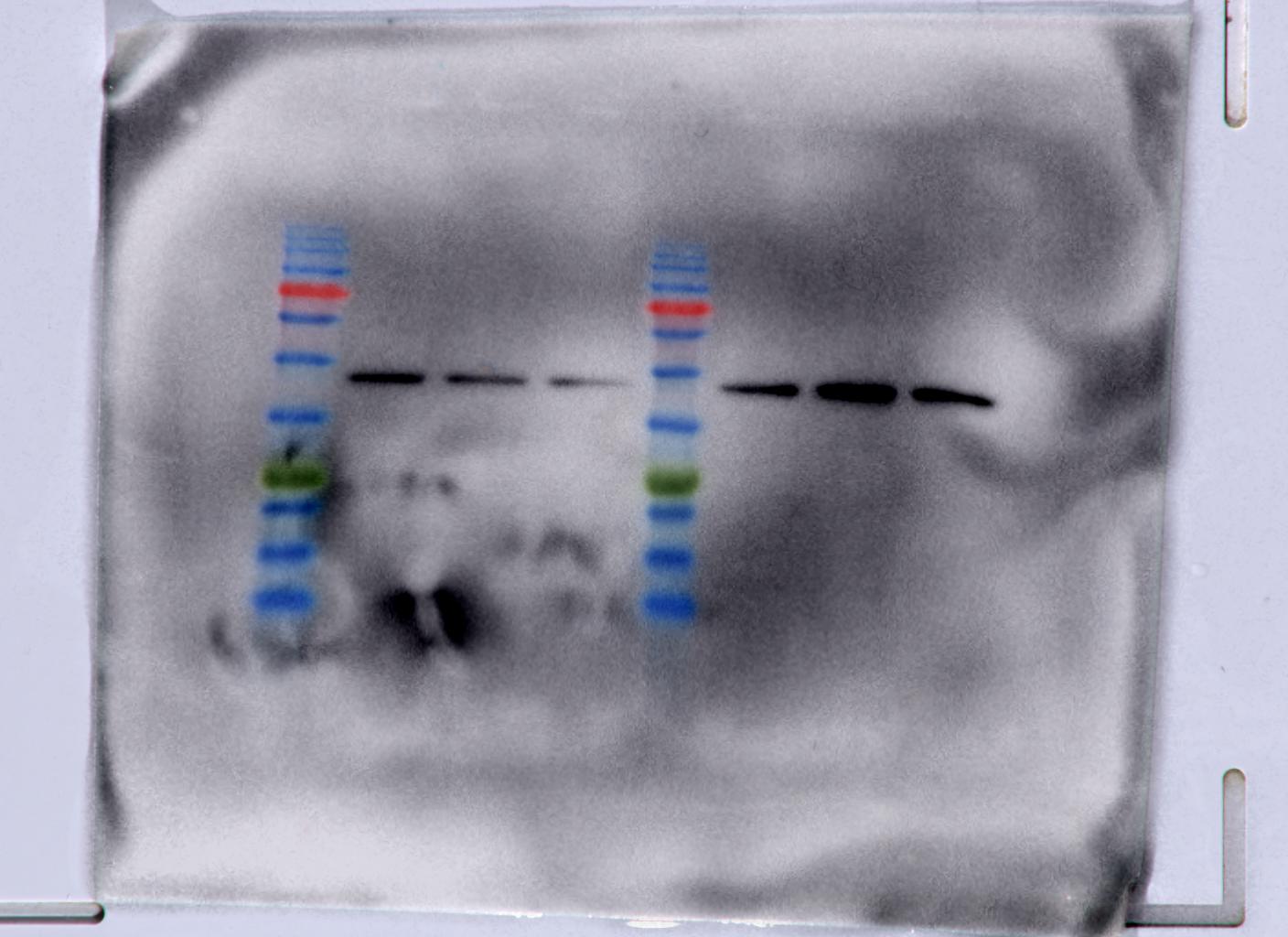


Lane 1: 40Q

Lane 2: 40Q; *unc-1(e1598)*

Lane 3: 40Q; *unc-1(e719)*

WB_1-2: *vlt10* and *fc73* alleles. Representative images (sample 1) to EV1C.

anti-actin (1:500) anti-polyQ (1:1000)


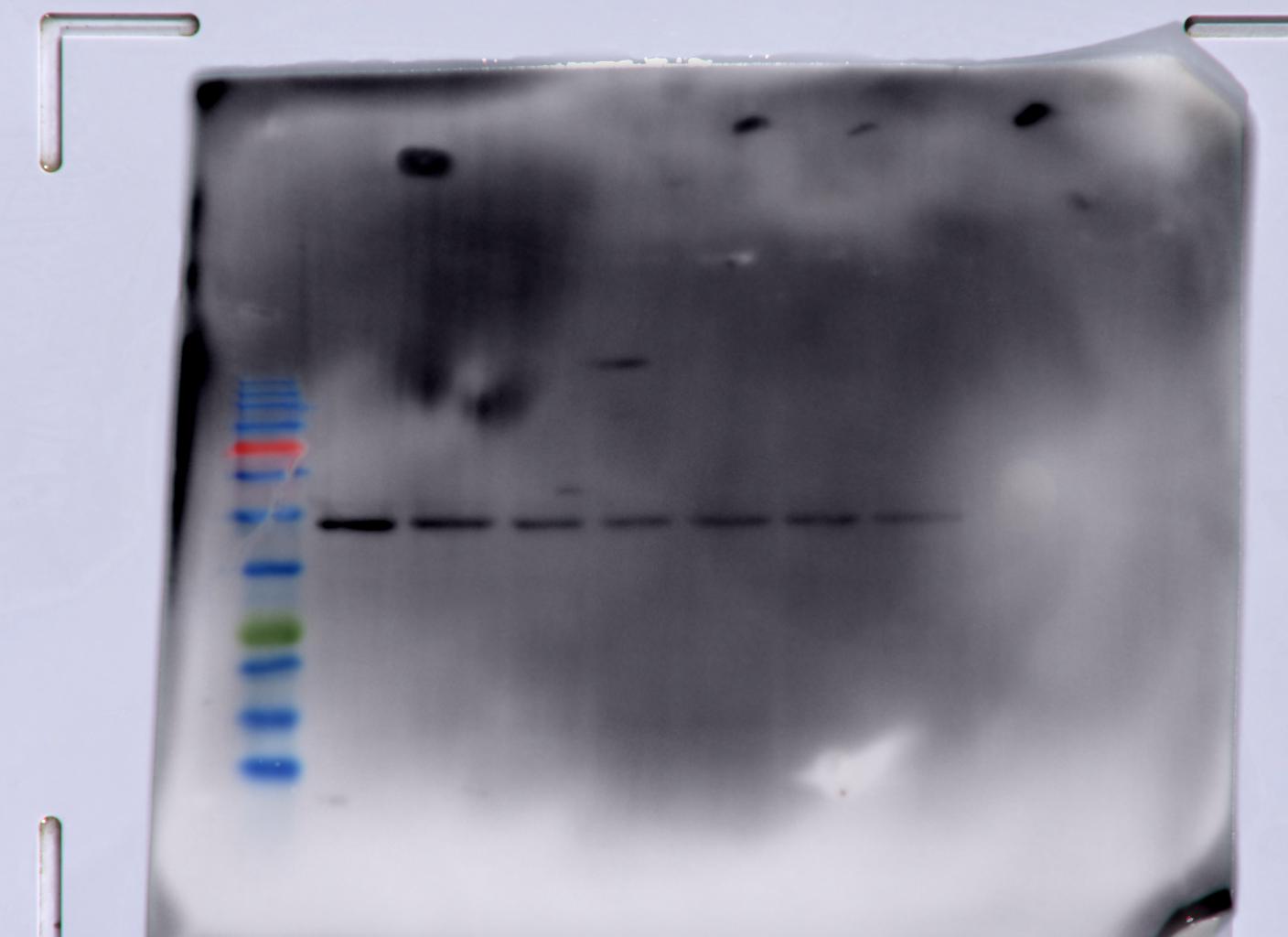


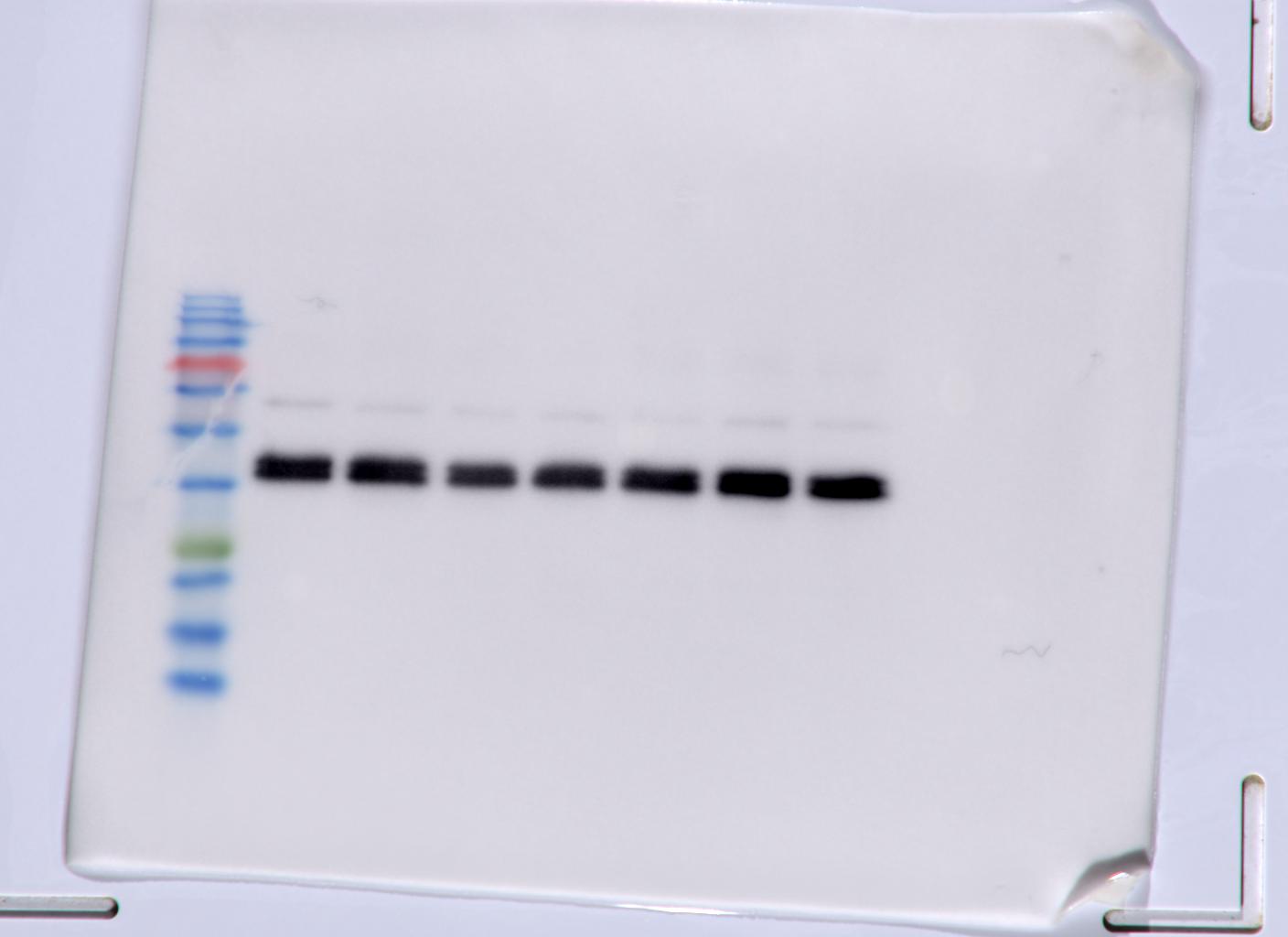


Lane 1: 40Q

Lane 2: 40Q; ssu-1(fc73)_sample 1

Lane 3: 40Q; unc-1(vlt10); ssu-1(fc73)_ sample 1

Lane 4: 40Q; ssu-1(fc73)_sample 2

Lane 5: 40Q; unc-1(vlt10); ssu-1(fc73)_ sample 2

WB_3: *vlt10* and *fc73* alleles

anti-actin (1:500) anti-polyQ (1:1000)


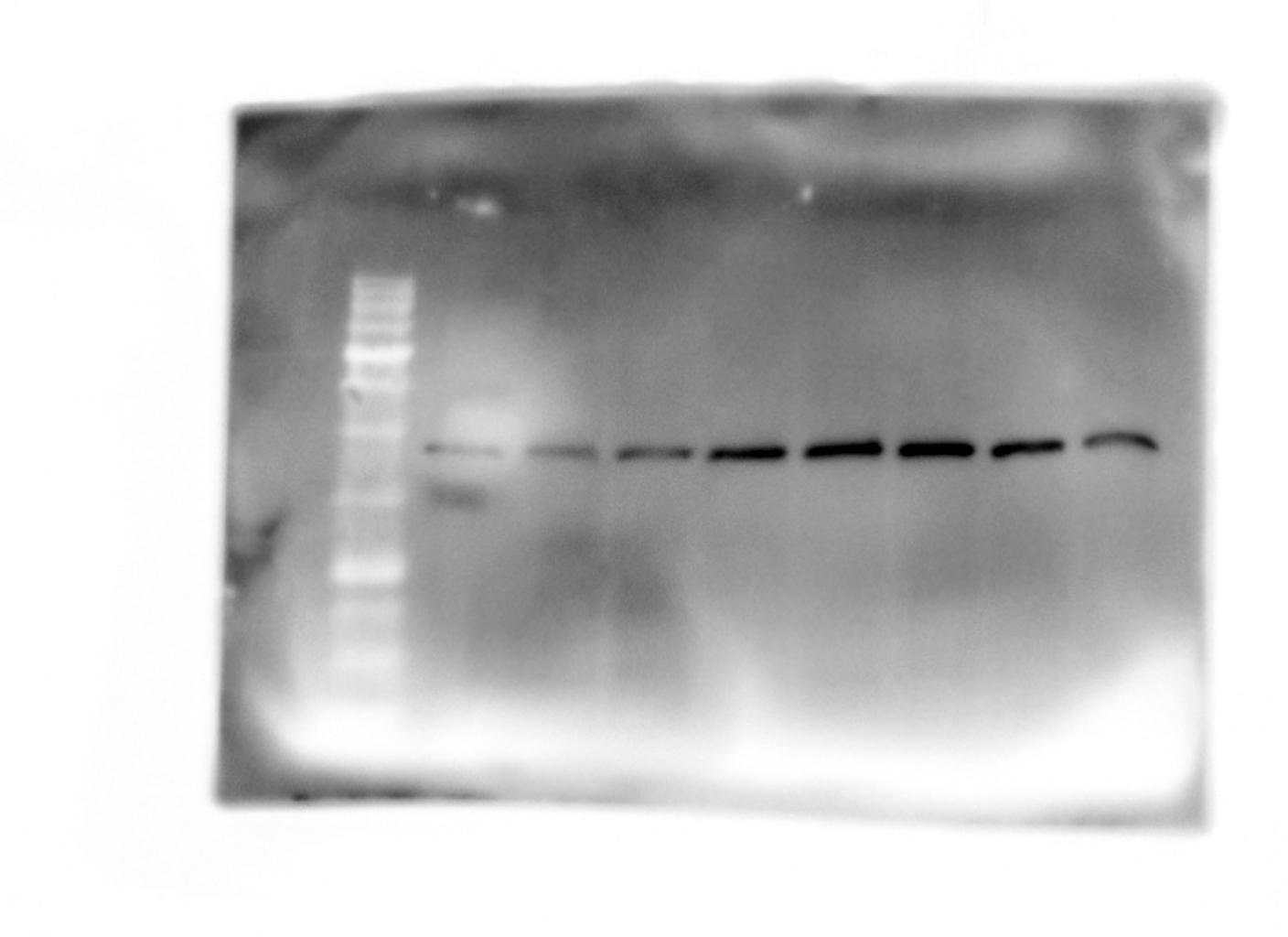


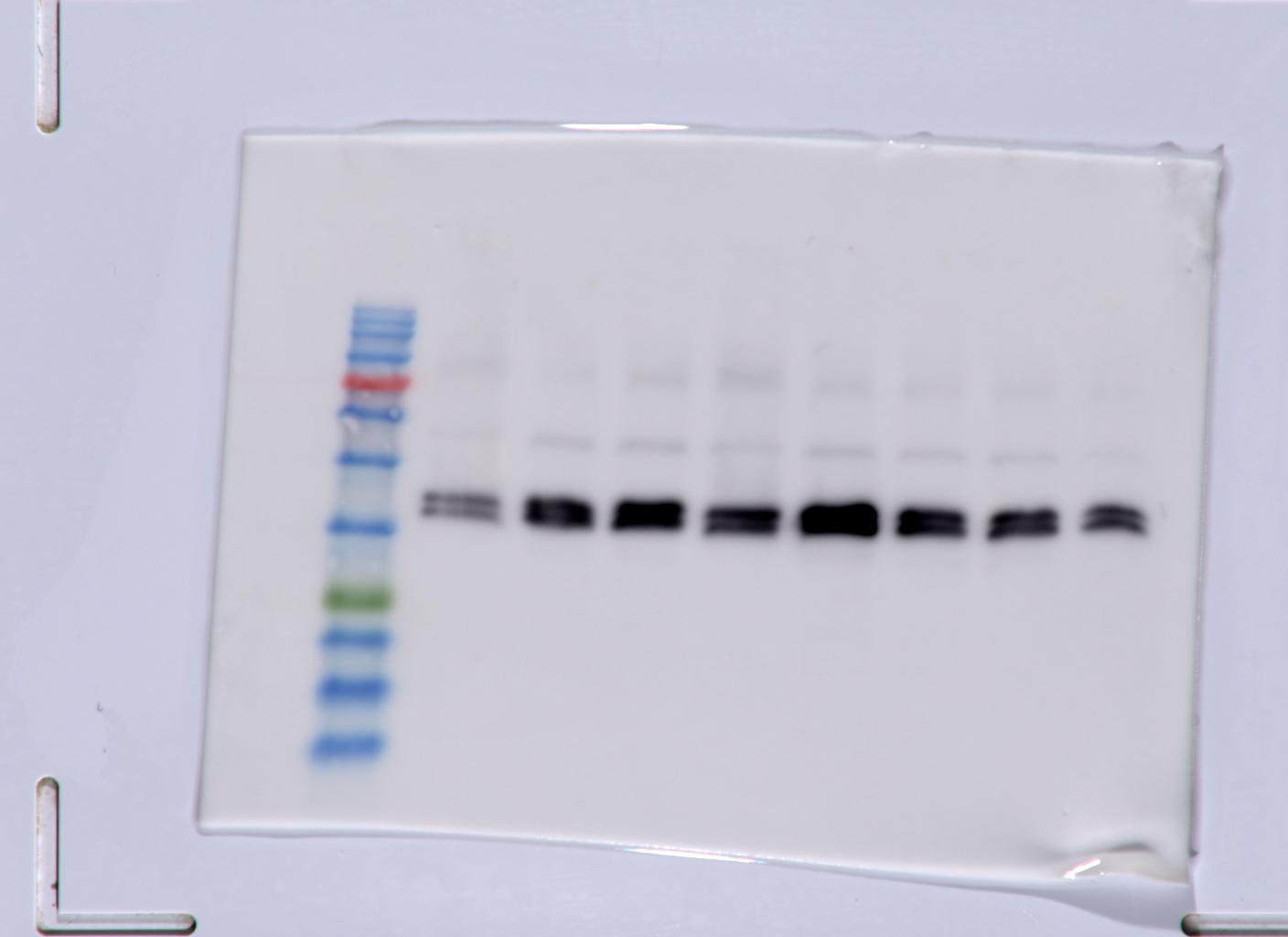


Lane 4: 40Q

Lane 5: 40Q; *ssu-1(fc73)*_

sample 3

Lane 6: 40Q; *unc-1(vlt10); ssu-1(fc73)*_ sample 3

WB_1-3: *vlt10* and *gk187* alleles. Representative images (sample 2) to EV1C.


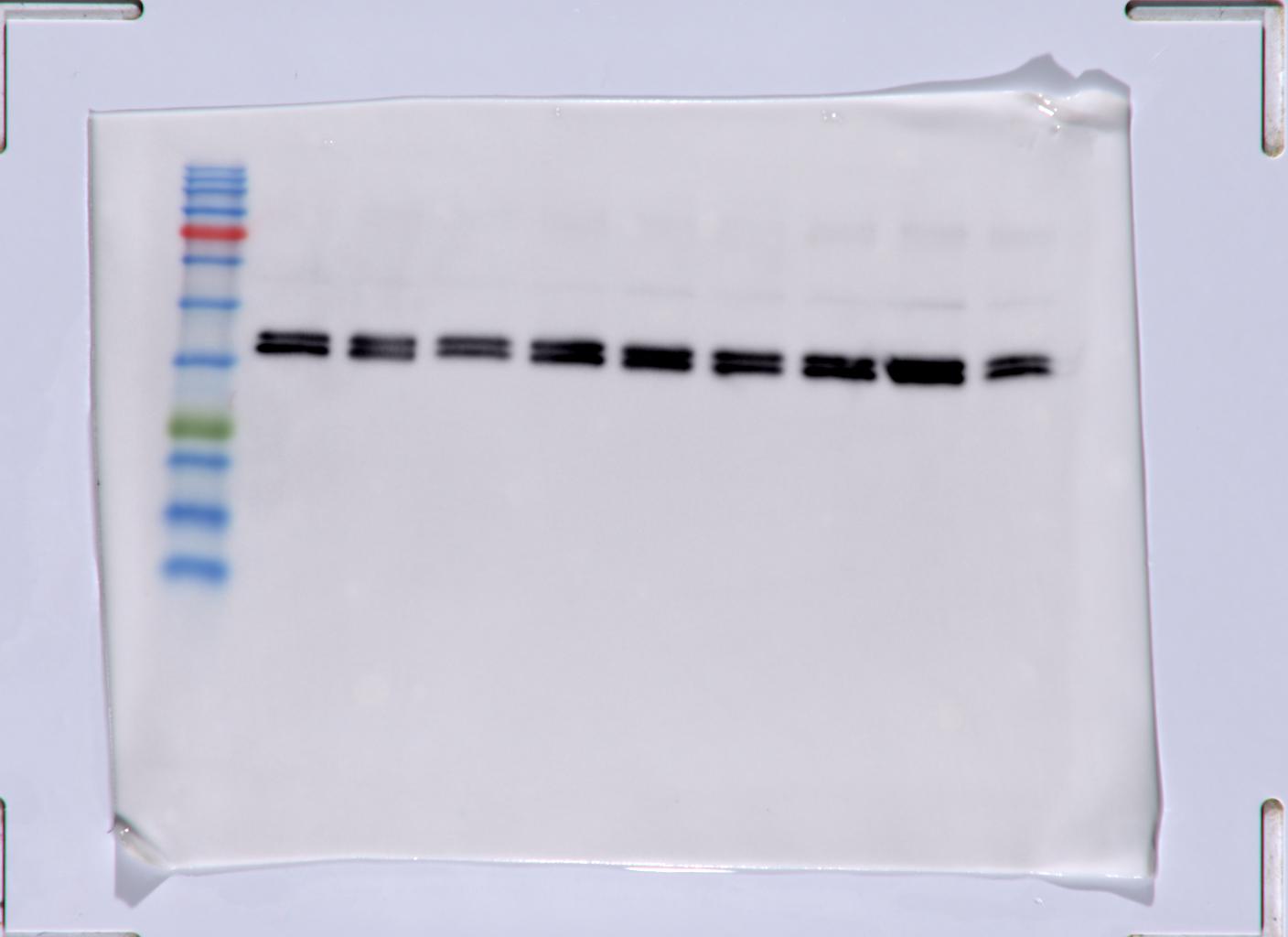

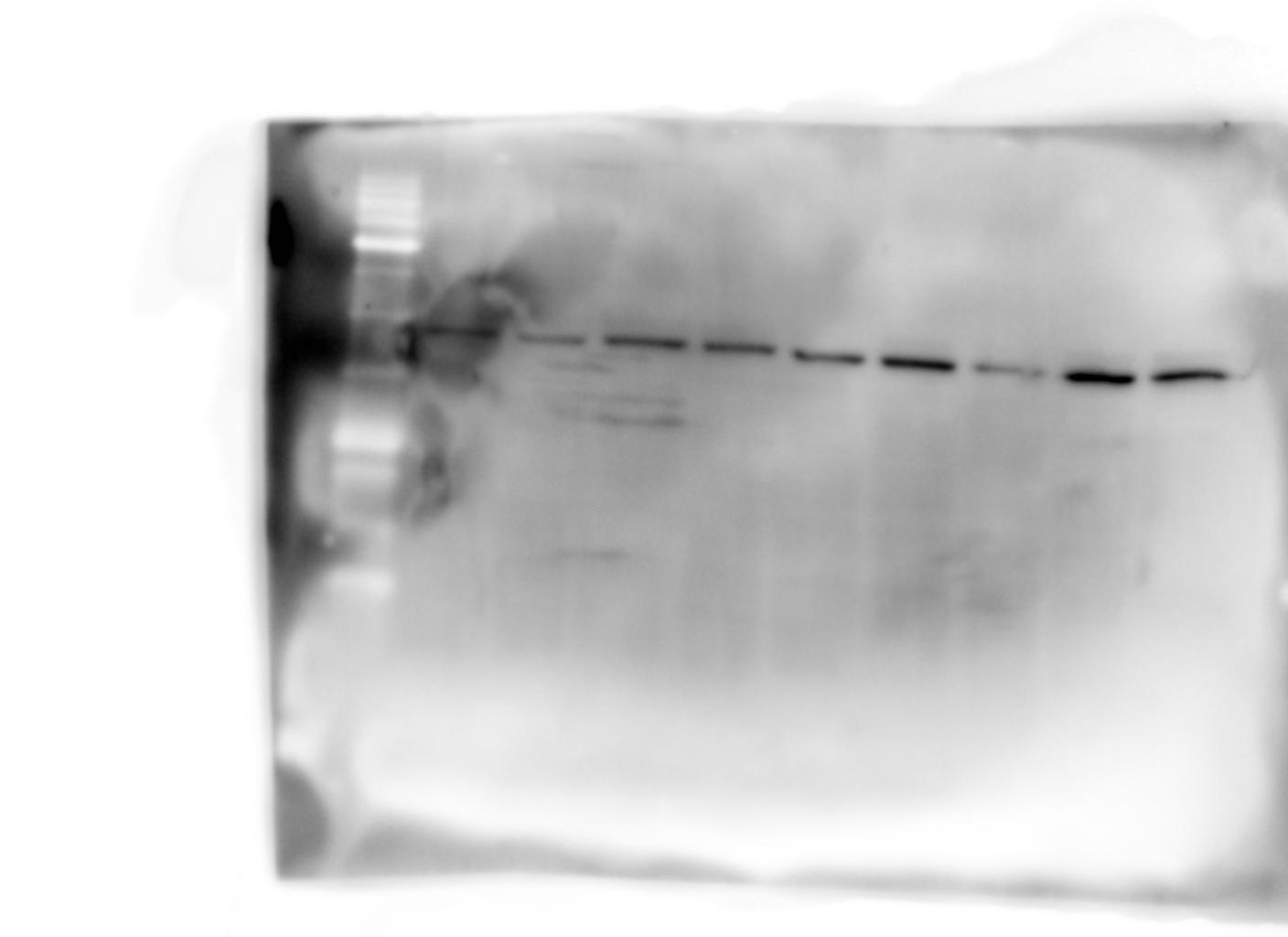
 anti-actin (1:500) anti-polyQ (1:1000)

Lane 2: 40Q;*sul-2(gk187)*_sample 1

Lane 3: 40Q;*unc-1(vlt10); sul-2(gk187)*_sample 1

Lane 4: 40Q

Lane 5: 40Q;*sul-2(gk187)*_sample 2

Lane 6: 40Q;*unc-1(vlt10); sul-2(gk187)*_sample 2

Lane 8: 40Q;*sul-2(gk187)*_sample 3

Lane 9: 40Q;*unc-1(vlt10); sul-2(gk187)*_sample 3

WB_1-3: *vlt19* allele. Representative images (sample 3) to EV1C.

anti-actin (1:500) anti-polyQ (1:1000)


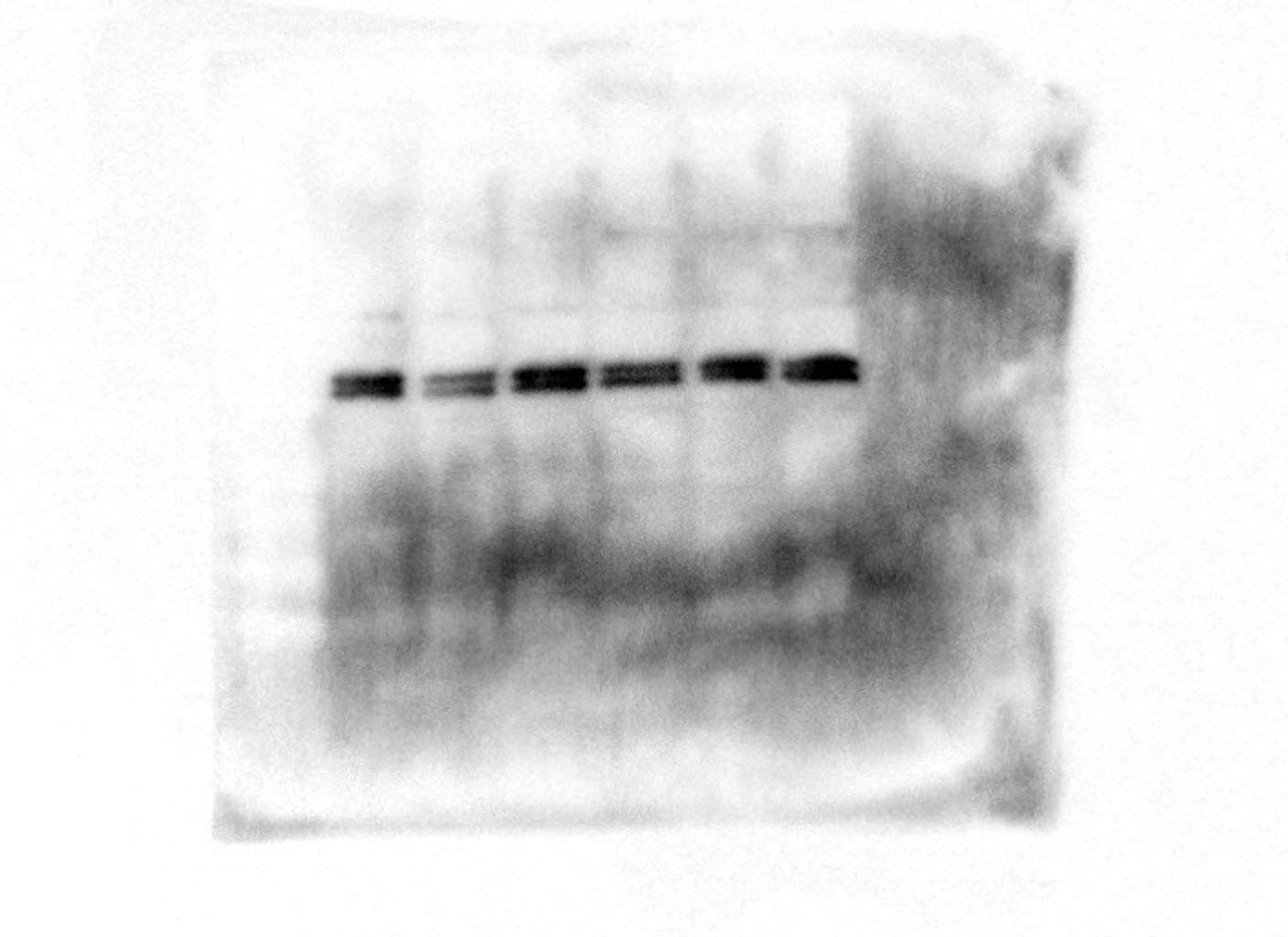

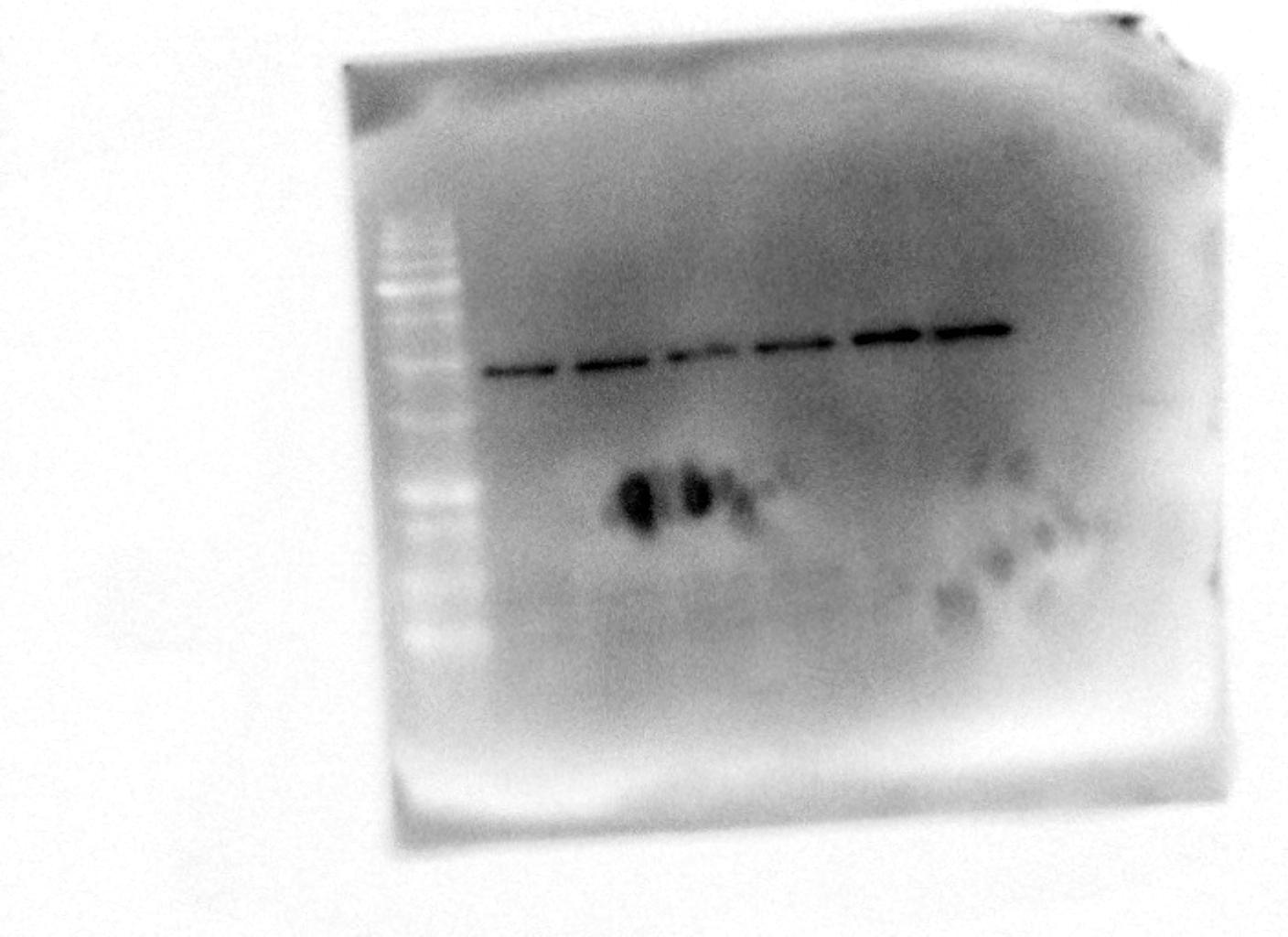


Lane 1: 40Q_sample 1

Lane 2: 40Q; *daf-12(vlt19)*_ sample 1

Lane 3: 40Q_sample 2

Lane 4: 40Q; *daf-12(vlt19)*_ sample 2

Lane 5: 40Q_sample 3

Lane 6: 40Q; *daf-12(vlt19)*_ sample 3

WB_1-3: *vlt15* and *vlt10* alleles. Representative images (sample 3) to EV1C.

anti-actin (1:500) anti-polyQ (1:1000)


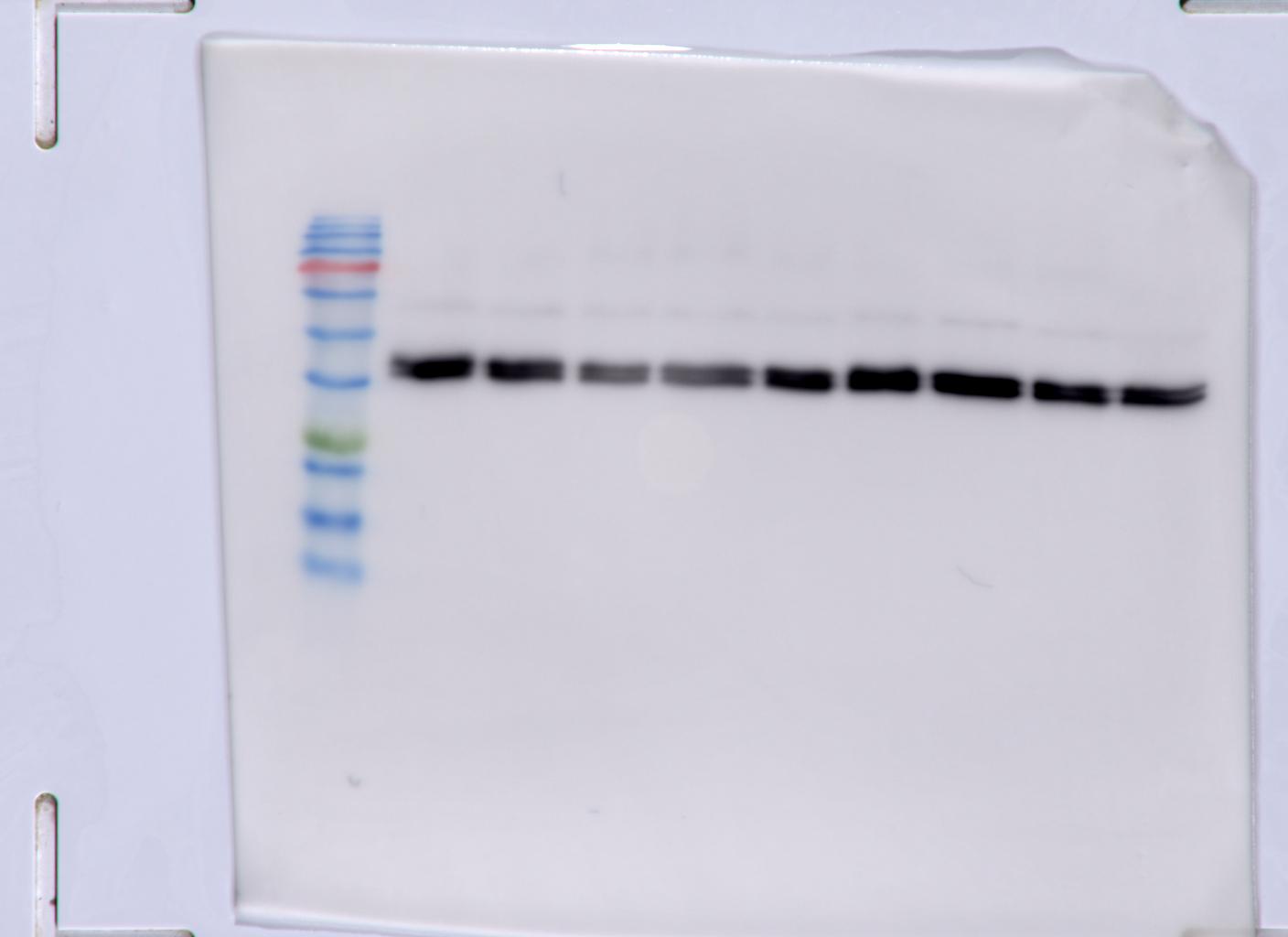

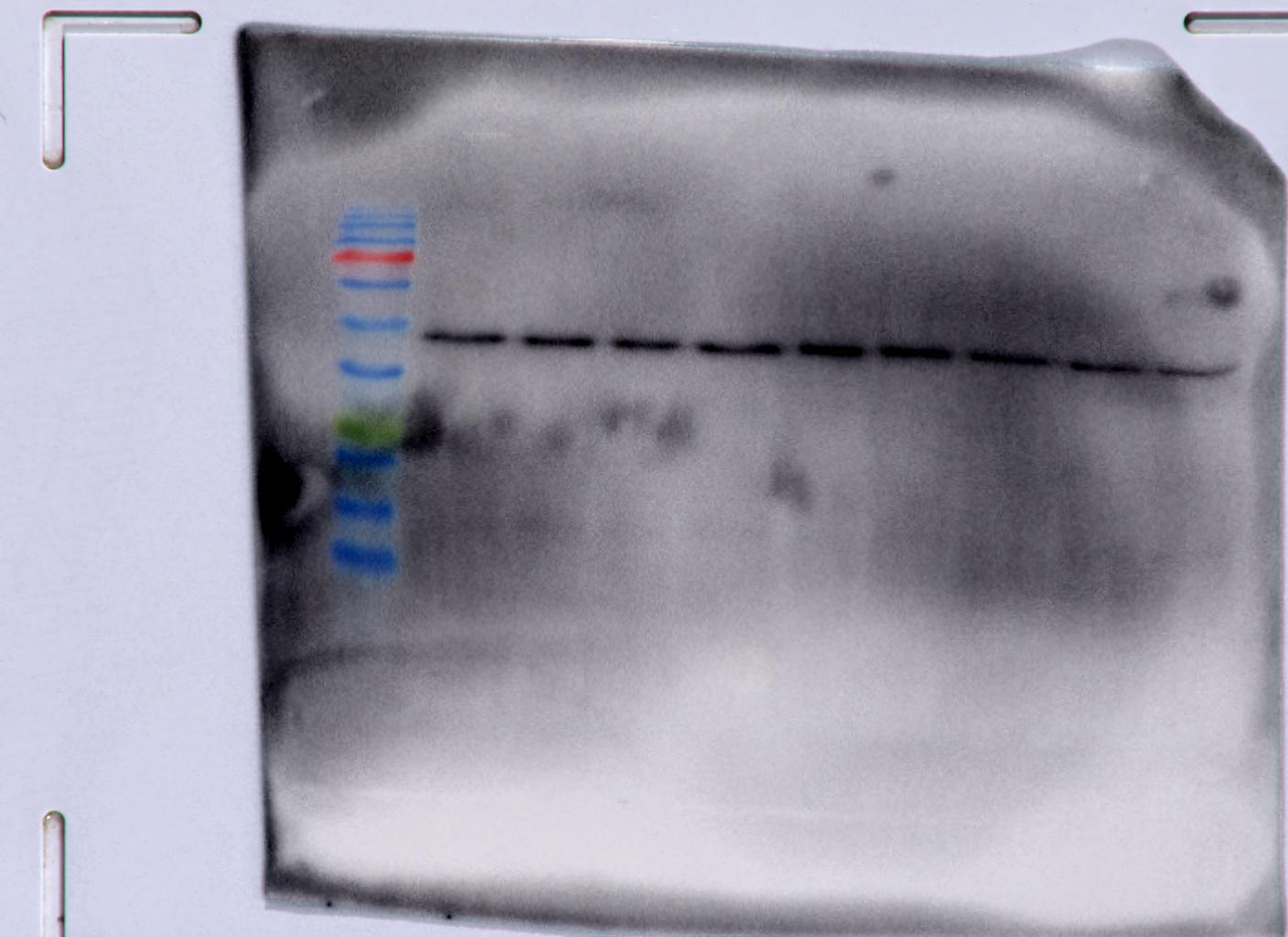


Lane 1: 40Q_sample 1

Lane 2: 40Q; *nhr-1(vlt15)*_ sample 1

Lane 3: 40Q; *unc-1(vlt10)*; *nhr-1(vlt15)*_ sample 1

Lane 4: 40Q_sample 2

Lane 5: 40Q; *nhr-1(vlt15)*_ sample 2

Lane 6: 40Q; *unc-1(vlt10)*; *nhr-1(vlt15)*_ sample 2

Lane 7: 40Q_sample 3

Lane 8: 40Q; *nhr-1(vlt15)*_ sample 3

Lane 9: 40Q; *unc-1(vlt10)*; *nhr-1(vlt15)*_ sample 3

WB_1-2: *vlt20, vlt15* and *vlt10* alleles

anti-actin (1:500) anti-polyQ (1:1000)


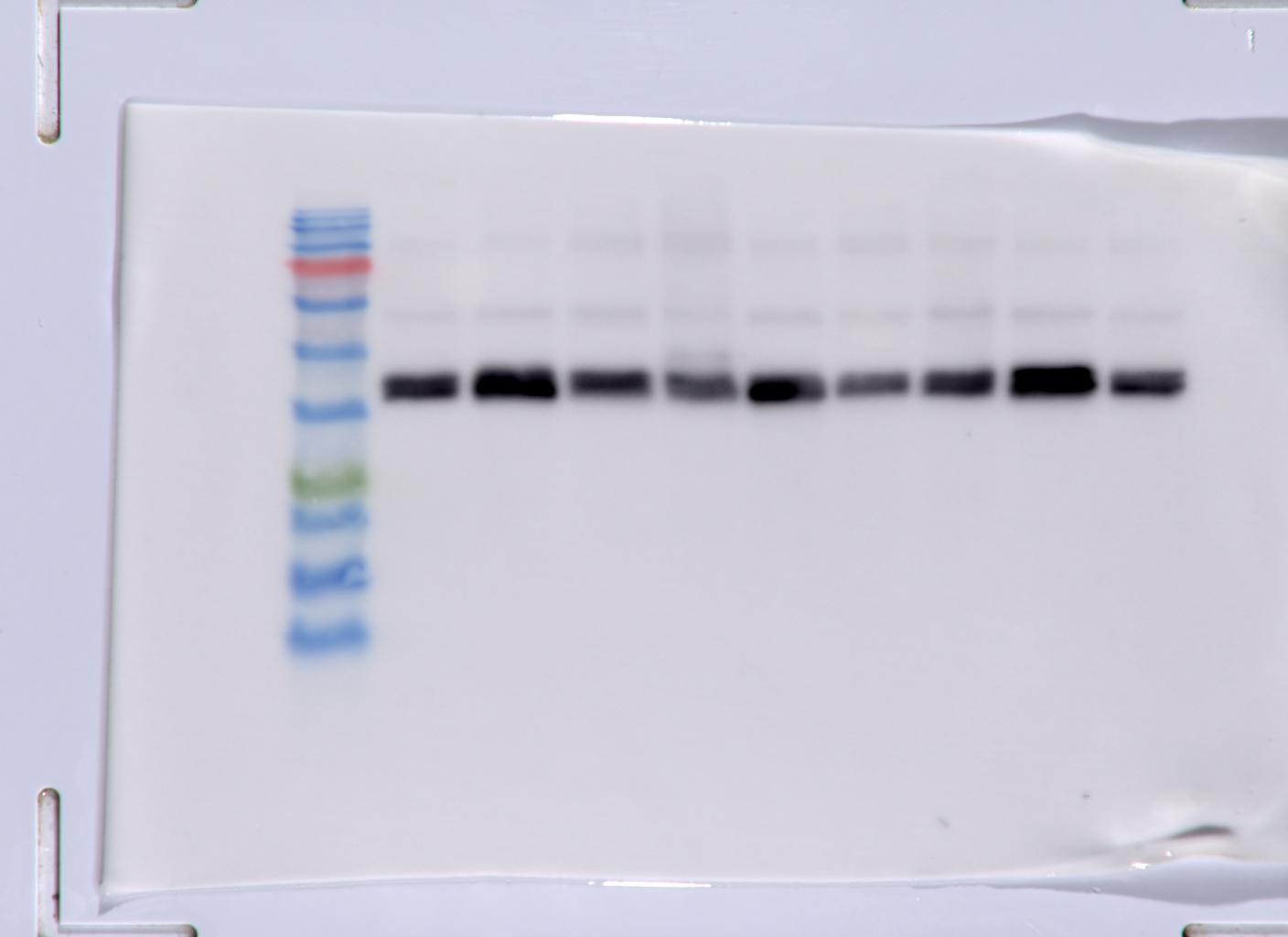

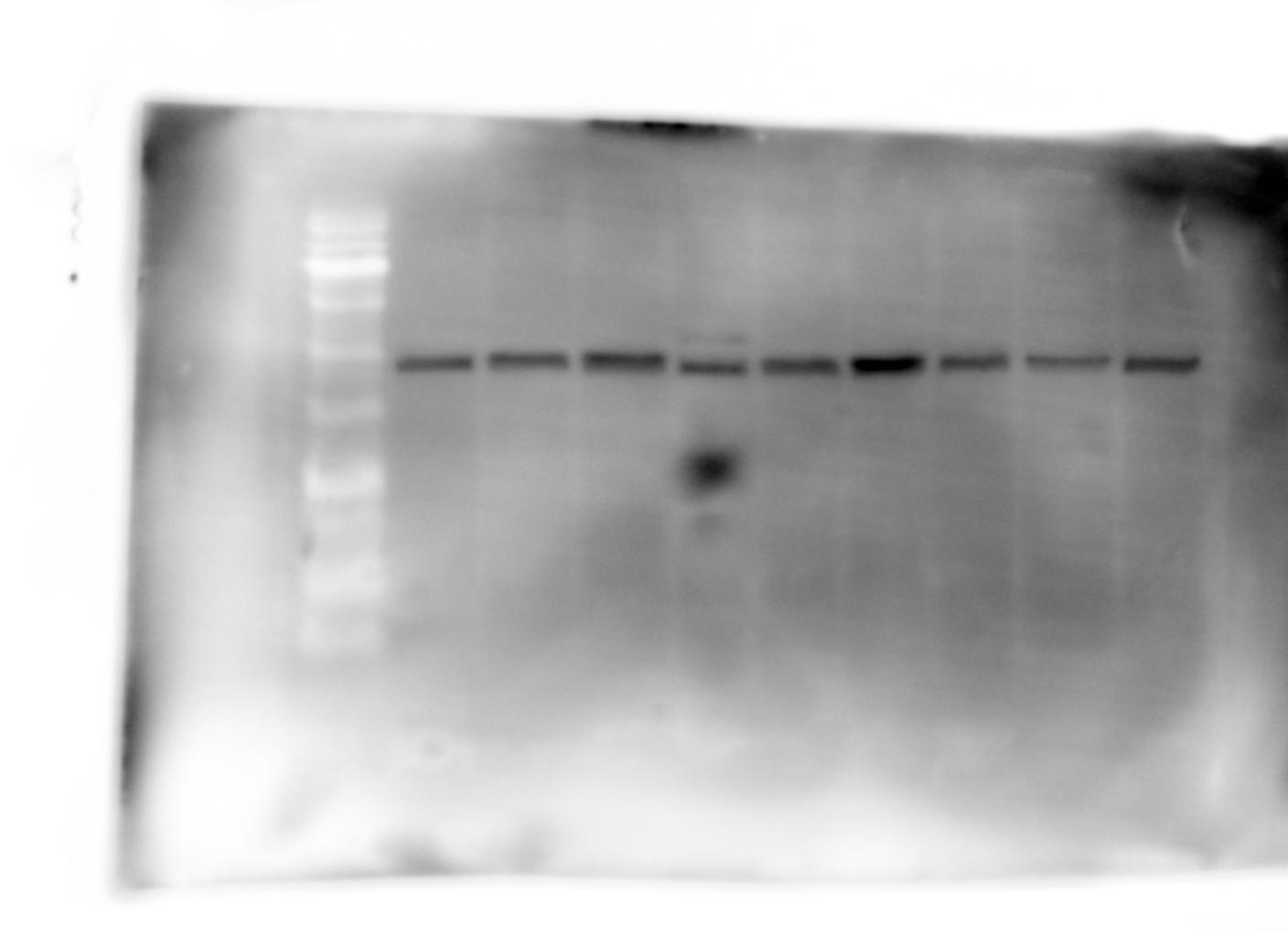


Lane 1: 40Q_ Sample 1

Lane 2: 40Q; *daf-12(vlt20); nhr-1(vlt15)* _ Sample 1

Lane 3: 40Q; *daf-12(vlt20); nhr-1(vlt15); unc-1(vlt10)* _ Sample 1

Lane 4: 40Q_ Sample 2

Lane 5: 40Q; *daf-12(vlt20); nhr-1(vlt15)* _ Sample 2

Lane 9: 40Q; *daf-12(vlt20); nhr-1(vlt15); unc-1(vlt10)* _ Sample 2

WB_3: *vlt20, vlt15* and *vlt10* alleles. Representative images to EV1C.

anti-actin (1:500) anti-polyQ (1:1000)


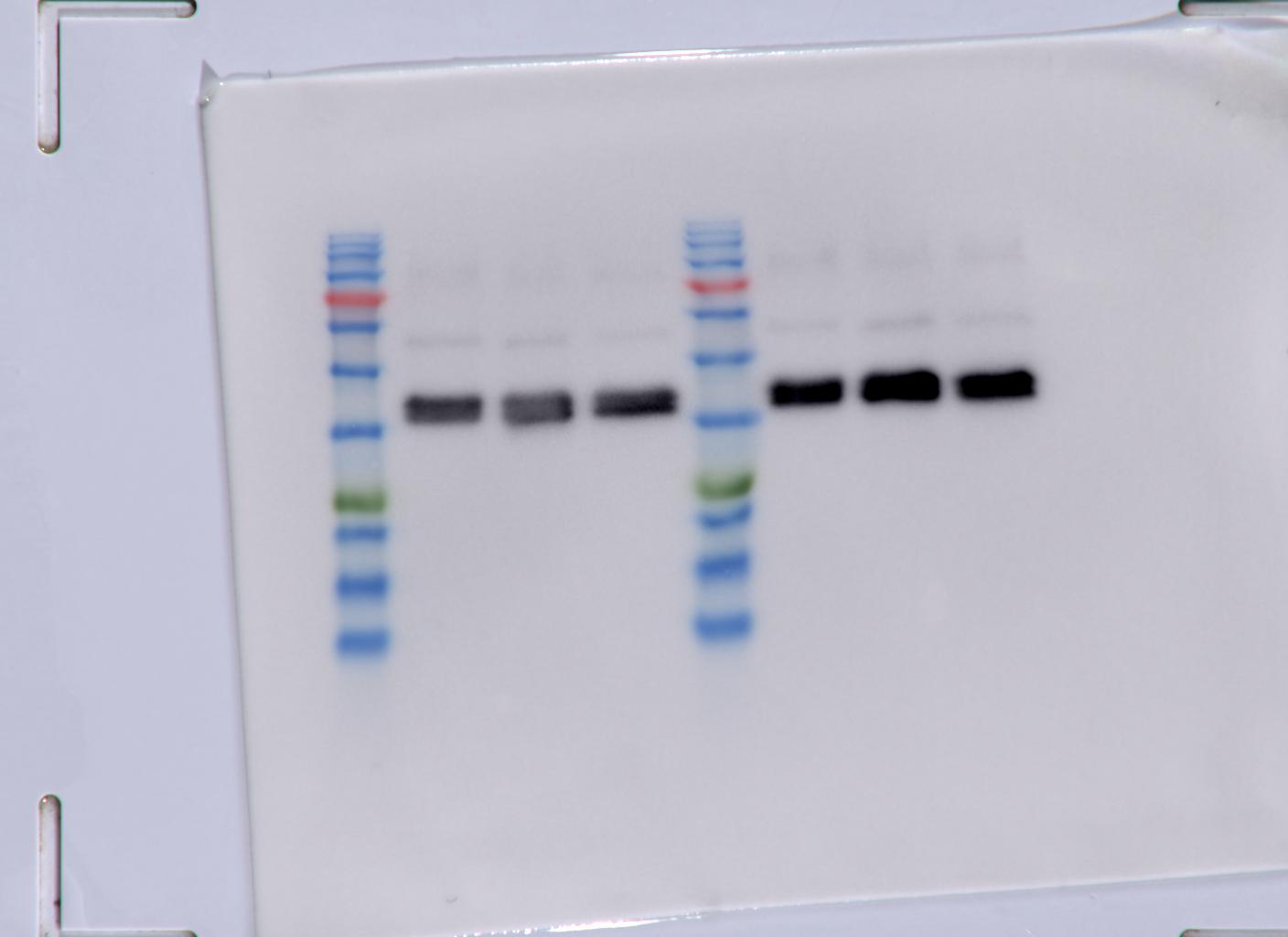

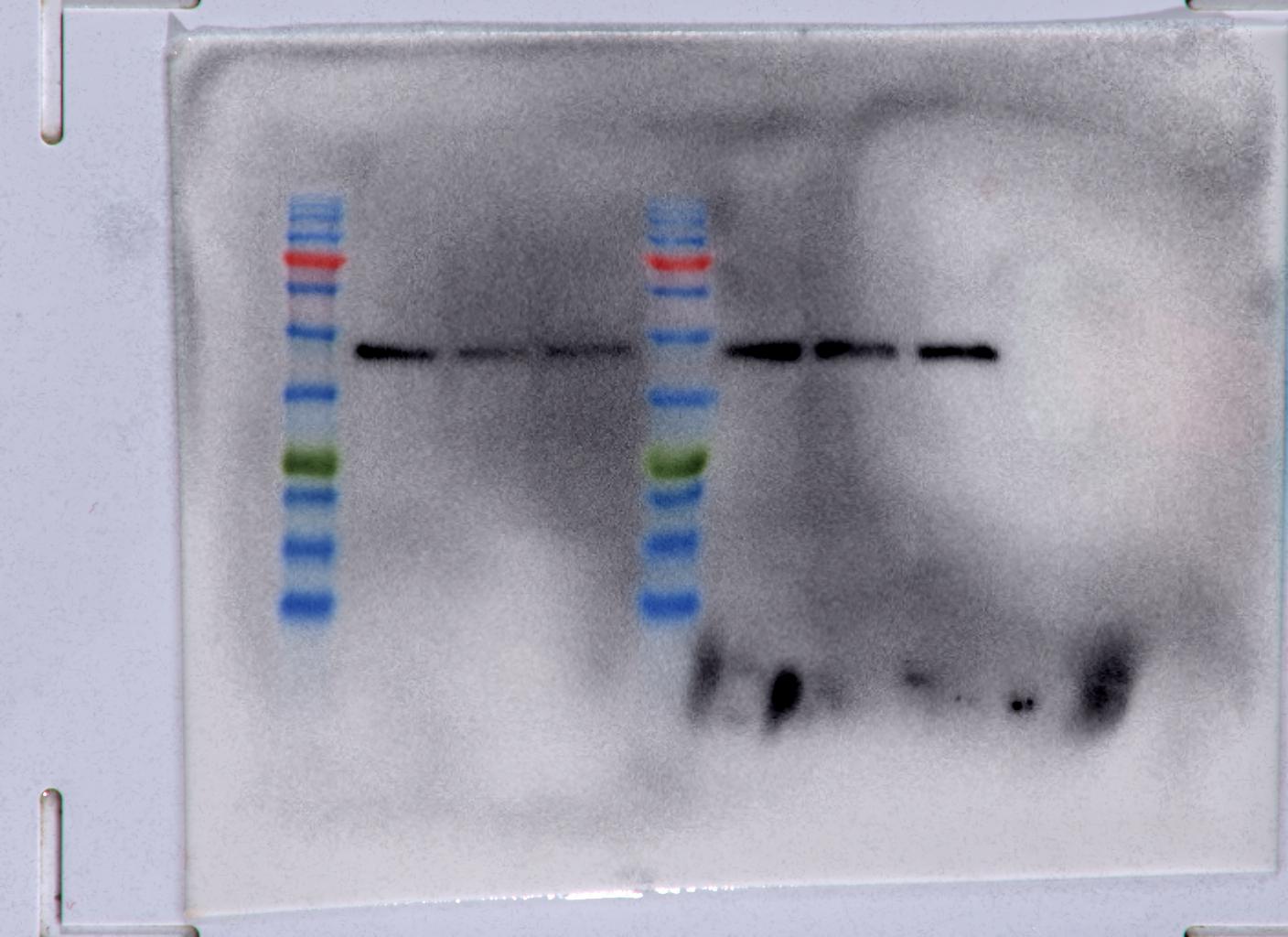


Lane 1: 40Q_ Sample 3

Lane 2: 40Q; *daf-12(vlt20); nhr-1(vlt15)* _ Sample 3

Lane 3: 40Q; *daf-12(vlt20); nhr-1(vlt15); unc-1(vlt10)* _ Sample 3
