# Supplementary figures and images for: Changes in lipid metabolism driven by steroid signalling modulate proteostasis in C. elegans
Source: EMBO Rep. 2023 Apr 27;24(6):e55556. doi: 10.15252/embr.202255556 (PMC10240203; doi:10.15252/embr.202255556)

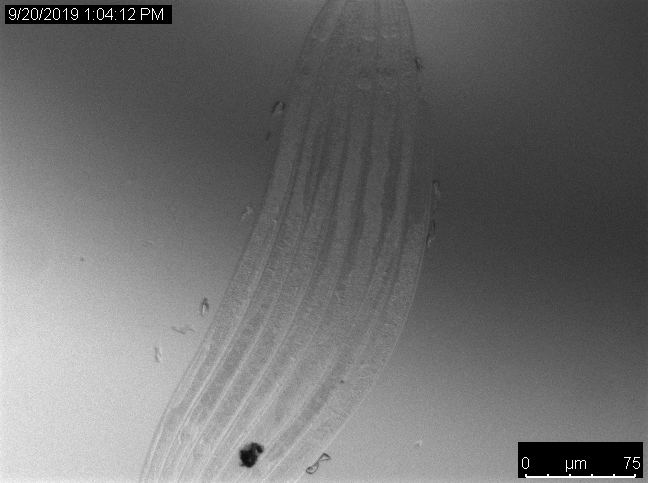

Supplement: Supplementary file 3 — Source Data for Expanded View and Appendix [file EMBR-24-e55556-s011.zip › EV_and_Appendix_Source_Data/EV_Figure_Source/Figure_EV1/Manuscript-EMBOR-2022-55556V3_SourceDataForFigureEV1E/hsp-4 dmso.lif_Image005_ch00.tif]

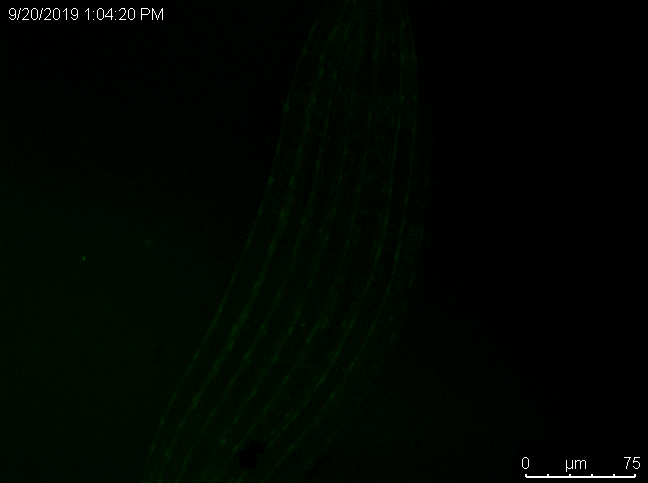

Supplement: Supplementary file 3 — Source Data for Expanded View and Appendix [file EMBR-24-e55556-s011.zip › EV_and_Appendix_Source_Data/EV_Figure_Source/Figure_EV1/Manuscript-EMBOR-2022-55556V3_SourceDataForFigureEV1E/hsp-4 dmso.lif_Image005_ch01.tif]

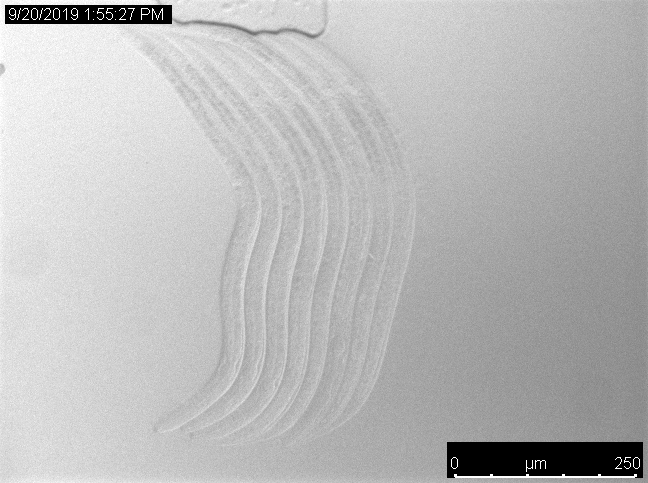

Supplement: Supplementary file 3 — Source Data for Expanded View and Appendix [file EMBR-24-e55556-s011.zip › EV_and_Appendix_Source_Data/EV_Figure_Source/Figure_EV1/Manuscript-EMBOR-2022-55556V3_SourceDataForFigureEV1E/hsp-4 unc_1 tuni.lif_Image002_ch00.tif]

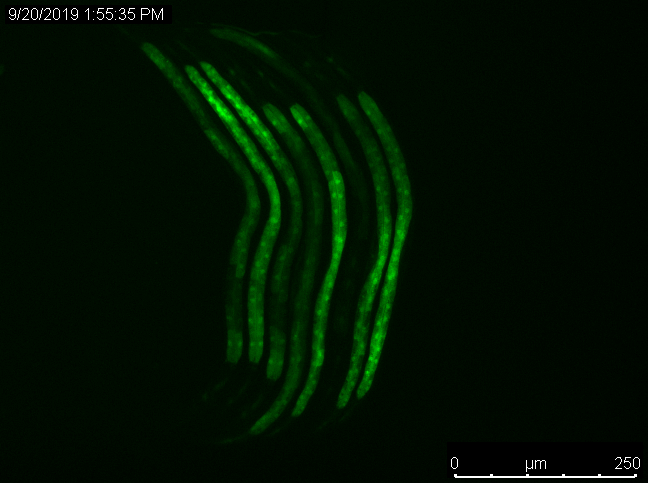

Supplement: Supplementary file 3 — Source Data for Expanded View and Appendix [file EMBR-24-e55556-s011.zip › EV_and_Appendix_Source_Data/EV_Figure_Source/Figure_EV1/Manuscript-EMBOR-2022-55556V3_SourceDataForFigureEV1E/hsp-4 unc_1 tuni.lif_Image002_ch01.tif]

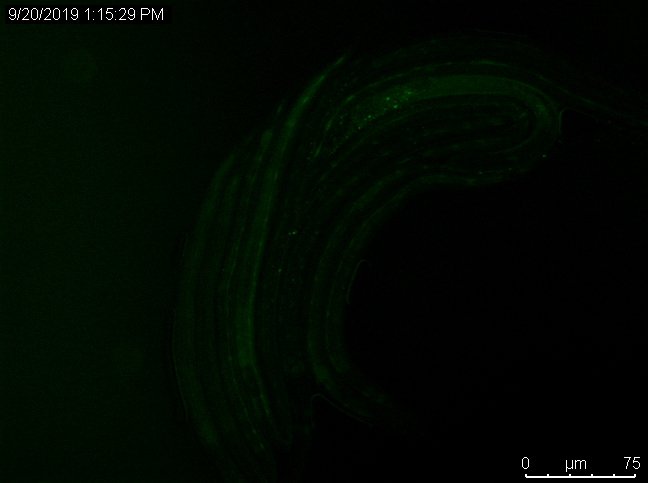

Supplement: Supplementary file 3 — Source Data for Expanded View and Appendix [file EMBR-24-e55556-s011.zip › EV_and_Appendix_Source_Data/EV_Figure_Source/Figure_EV1/Manuscript-EMBOR-2022-55556V3_SourceDataForFigureEV1E/hsp-4 unc_1 dmso.lif_Image007_ch01.tif]

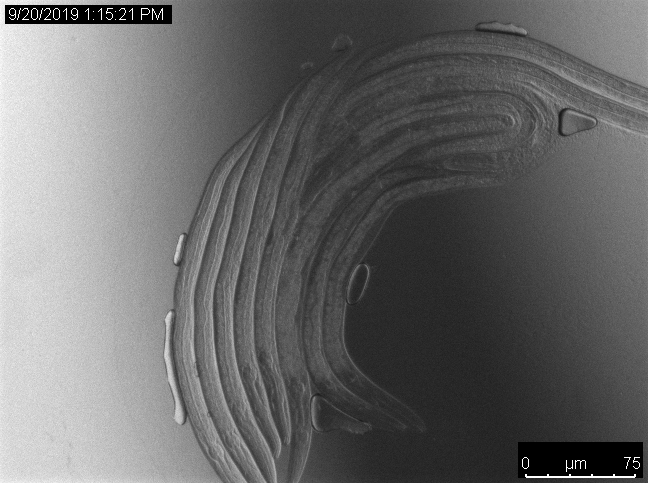

Supplement: Supplementary file 3 — Source Data for Expanded View and Appendix [file EMBR-24-e55556-s011.zip › EV_and_Appendix_Source_Data/EV_Figure_Source/Figure_EV1/Manuscript-EMBOR-2022-55556V3_SourceDataForFigureEV1E/hsp-4 unc_1 dmso.lif_Image007_ch00.tif]

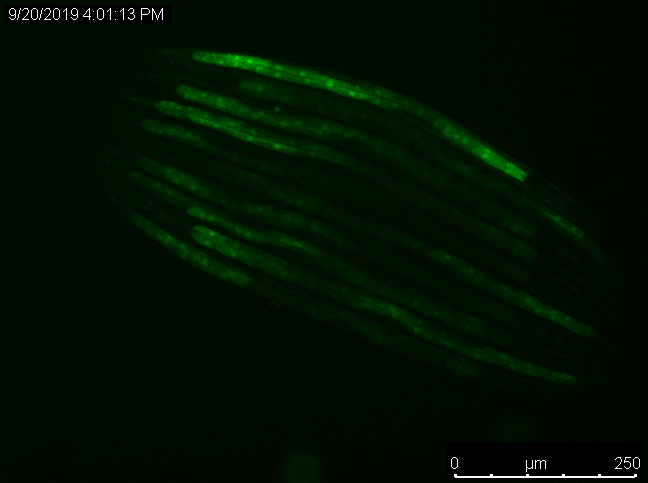

Supplement: Supplementary file 3 — Source Data for Expanded View and Appendix [file EMBR-24-e55556-s011.zip › EV_and_Appendix_Source_Data/EV_Figure_Source/Figure_EV1/Manuscript-EMBOR-2022-55556V3_SourceDataForFigureEV1E/hsp-4 tunicamycin.lif_Image009_ch01.tif]

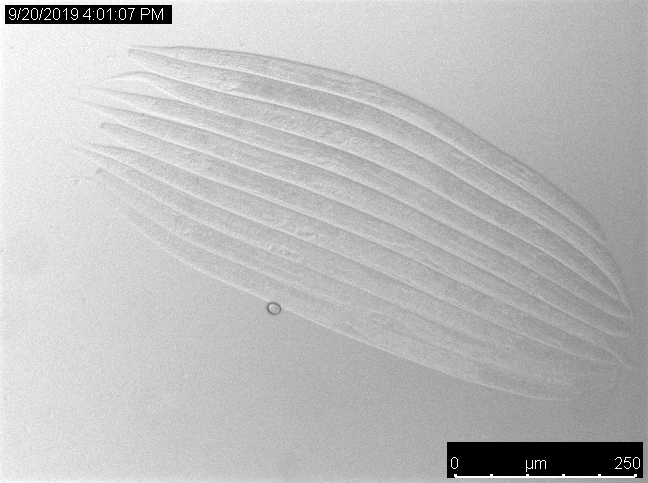

Supplement: Supplementary file 3 — Source Data for Expanded View and Appendix [file EMBR-24-e55556-s011.zip › EV_and_Appendix_Source_Data/EV_Figure_Source/Figure_EV1/Manuscript-EMBOR-2022-55556V3_SourceDataForFigureEV1E/hsp-4 tunicamycin.lif_Image009_ch00.tif]

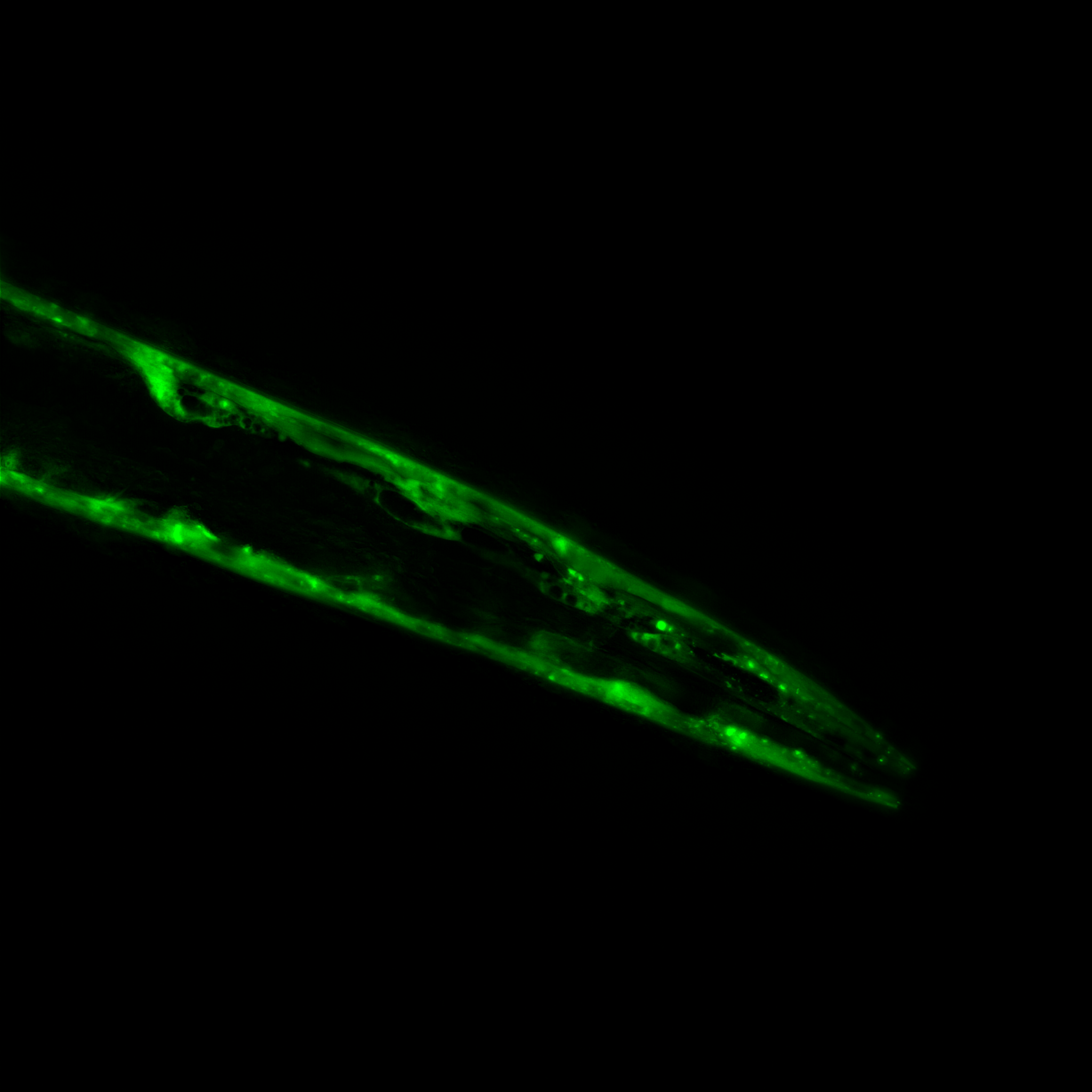

Supplement: Supplementary file 3 — Source Data for Expanded View and Appendix [file EMBR-24-e55556-s011.zip › EV_and_Appendix_Source_Data/EV_Figure_Source/Figure_EV2/Manuscript-EMBOR-2022-55556V3_SourceDataForFigureEV2A/nhr1_2.tif]

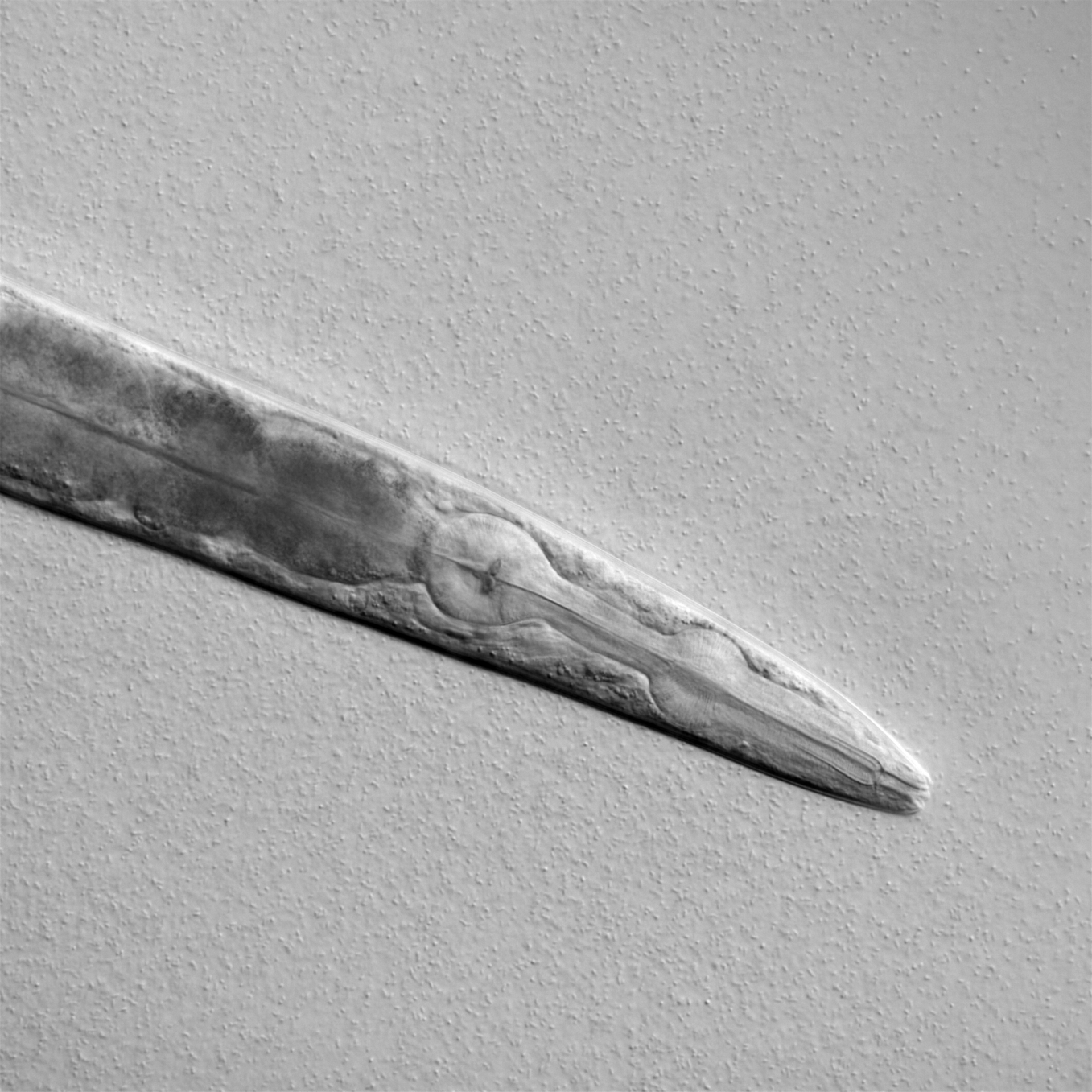

Supplement: Supplementary file 3 — Source Data for Expanded View and Appendix [file EMBR-24-e55556-s011.zip › EV_and_Appendix_Source_Data/EV_Figure_Source/Figure_EV2/Manuscript-EMBOR-2022-55556V3_SourceDataForFigureEV2A/nhr1_2dic.tif]

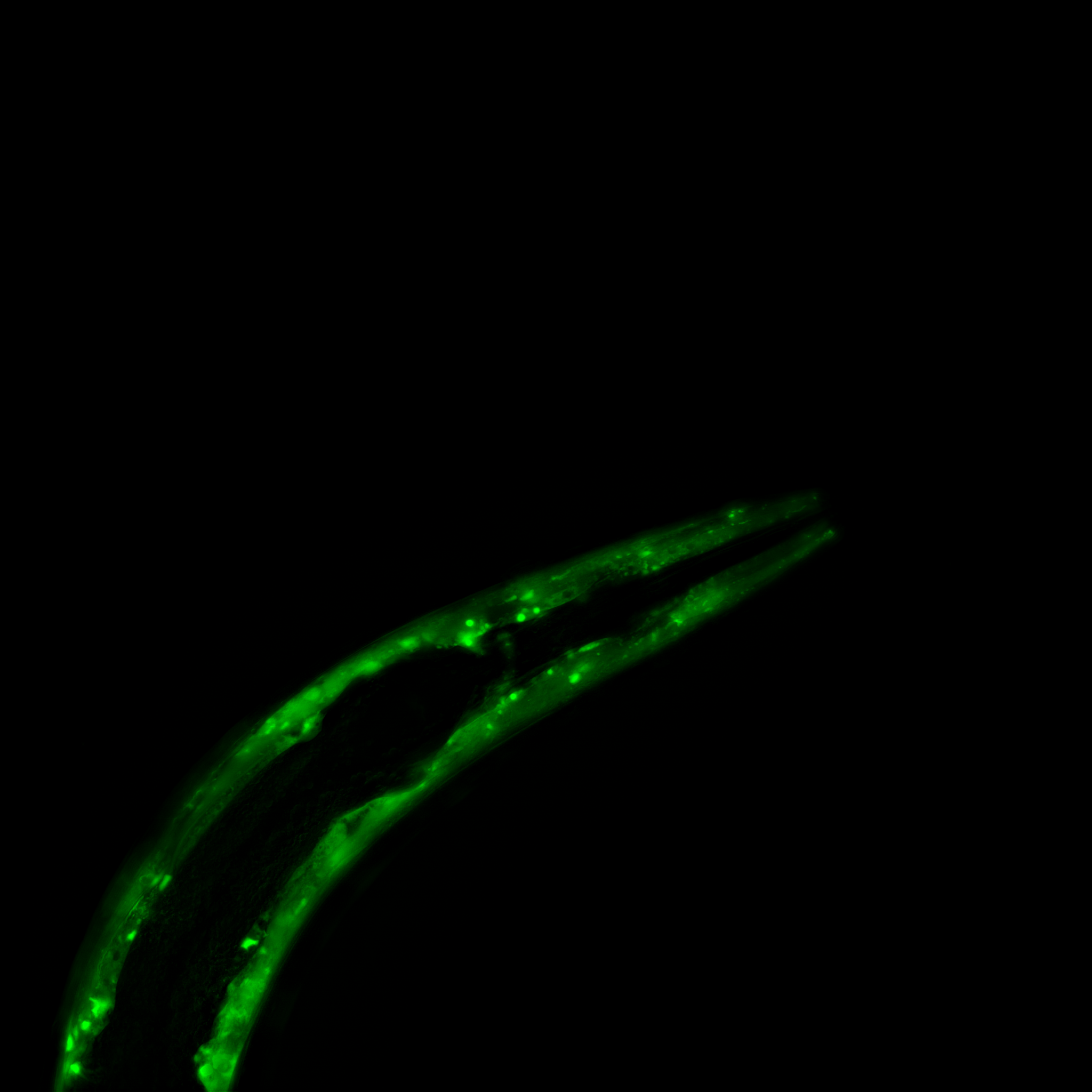

Supplement: Supplementary file 3 — Source Data for Expanded View and Appendix [file EMBR-24-e55556-s011.zip › EV_and_Appendix_Source_Data/EV_Figure_Source/Figure_EV2/Manuscript-EMBOR-2022-55556V3_SourceDataForFigureEV2A/alphasyn_3.tif]

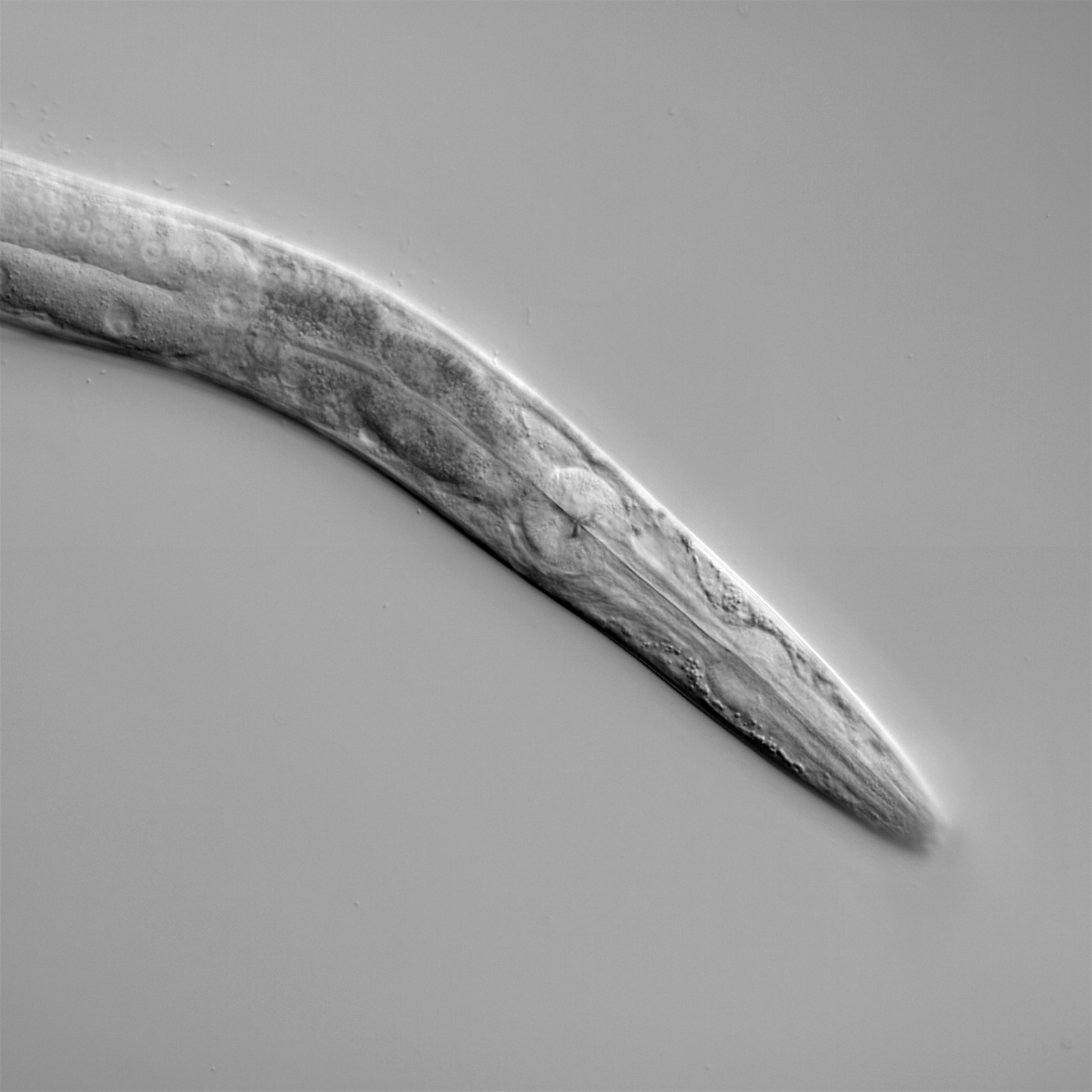

Supplement: Supplementary file 3 — Source Data for Expanded View and Appendix [file EMBR-24-e55556-s011.zip › EV_and_Appendix_Source_Data/EV_Figure_Source/Figure_EV2/Manuscript-EMBOR-2022-55556V3_SourceDataForFigureEV2A/unc1_nhr1_3_dic.tif]

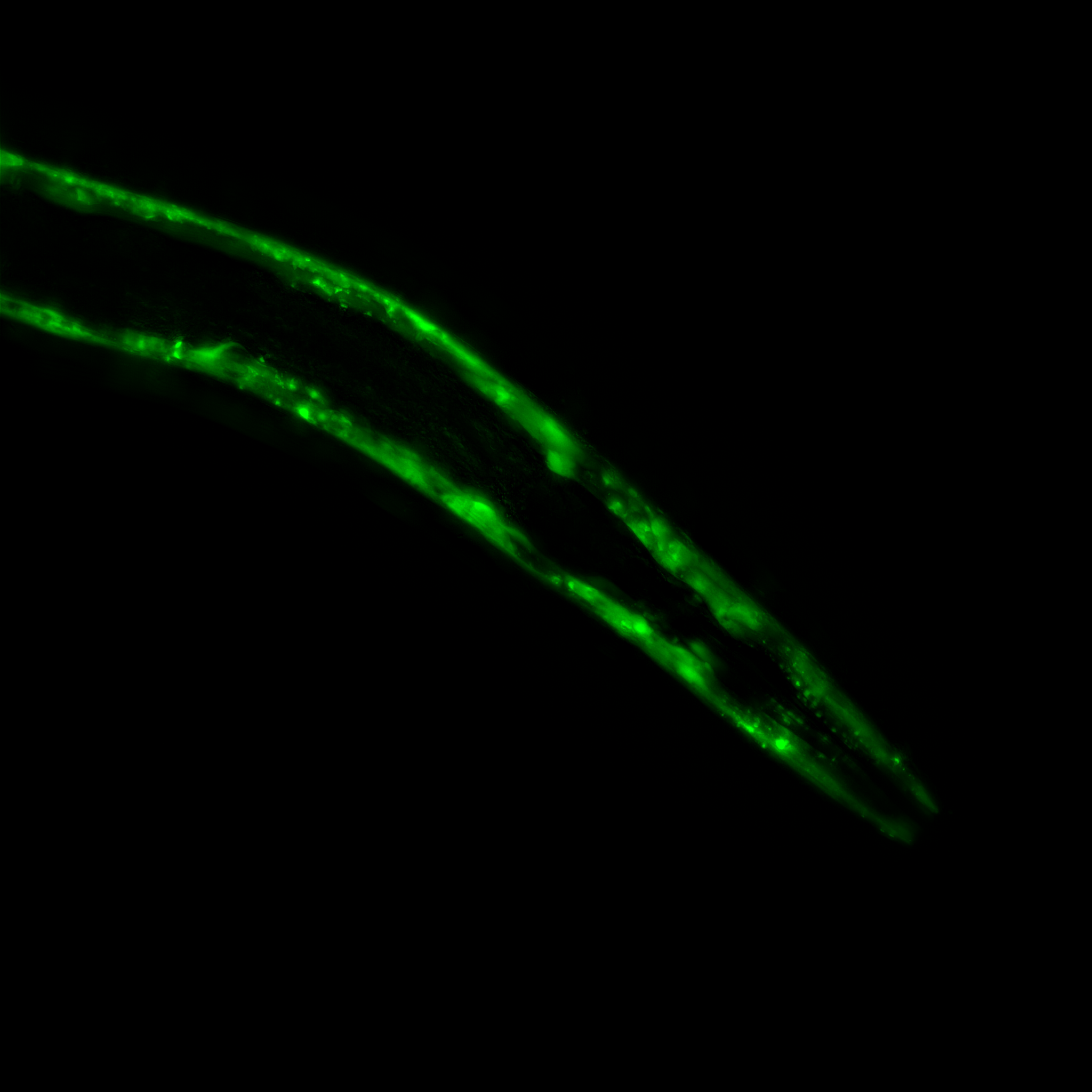

Supplement: Supplementary file 3 — Source Data for Expanded View and Appendix [file EMBR-24-e55556-s011.zip › EV_and_Appendix_Source_Data/EV_Figure_Source/Figure_EV2/Manuscript-EMBOR-2022-55556V3_SourceDataForFigureEV2A/unc1_nhr1_3.tif]

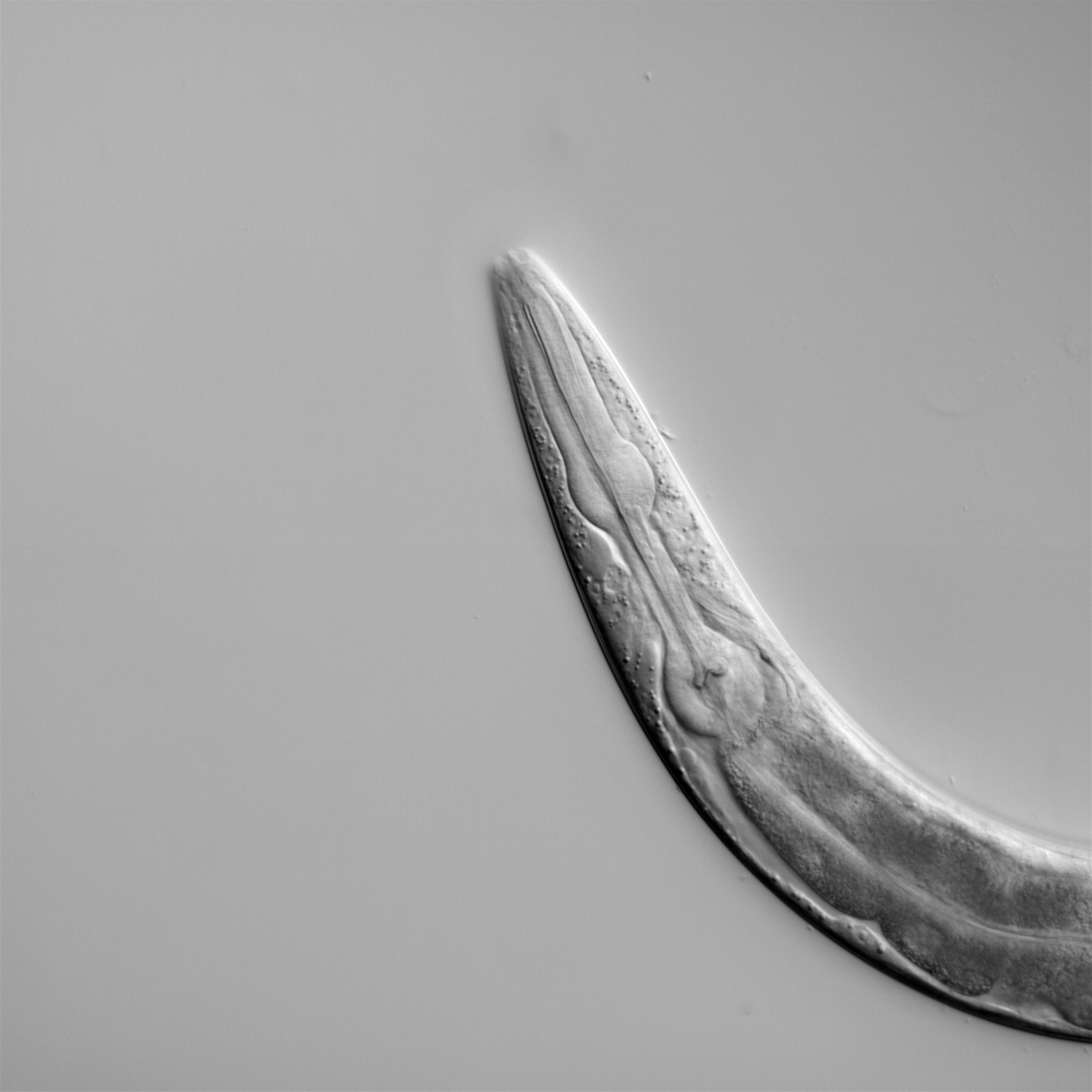

Supplement: Supplementary file 3 — Source Data for Expanded View and Appendix [file EMBR-24-e55556-s011.zip › EV_and_Appendix_Source_Data/EV_Figure_Source/Figure_EV2/Manuscript-EMBOR-2022-55556V3_SourceDataForFigureEV2A/alphasyn_3_dic.tif]

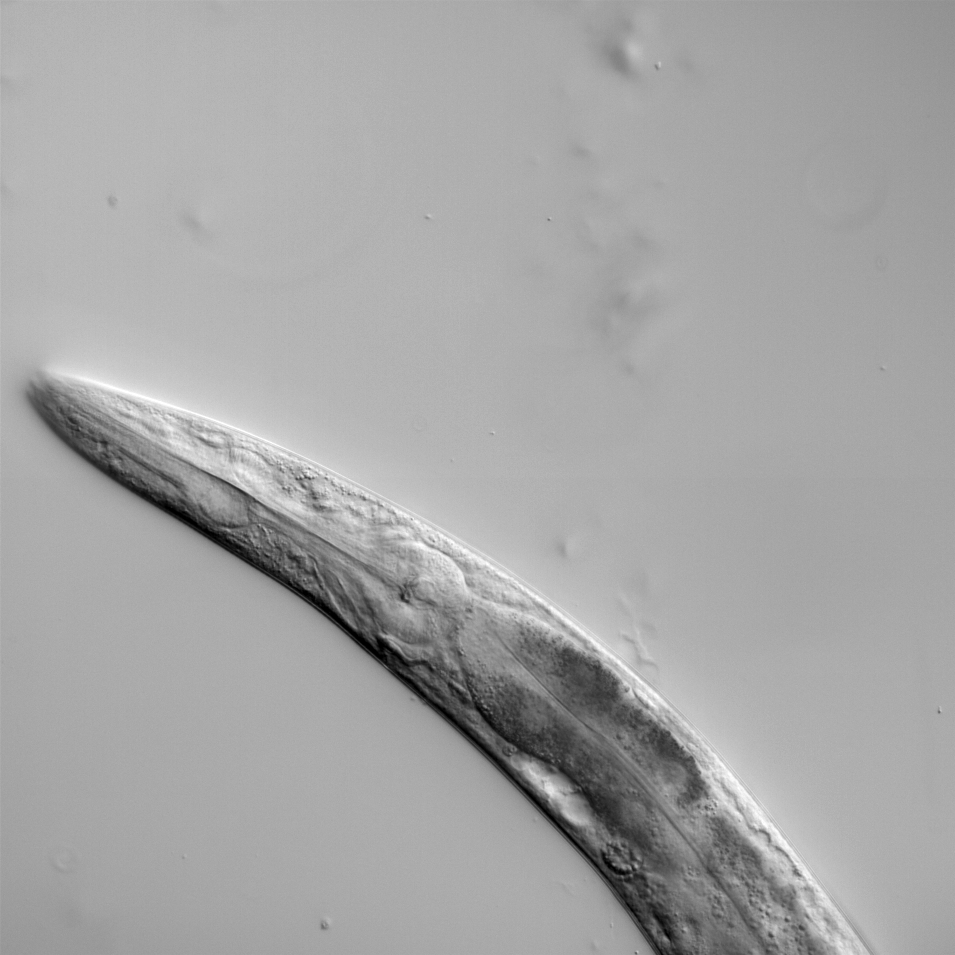

Supplement: Supplementary file 3 — Source Data for Expanded View and Appendix [file EMBR-24-e55556-s011.zip › EV_and_Appendix_Source_Data/EV_Figure_Source/Figure_EV2/Manuscript-EMBOR-2022-55556V3_SourceDataForFigureEV2A/unc1_1_dic.tif]

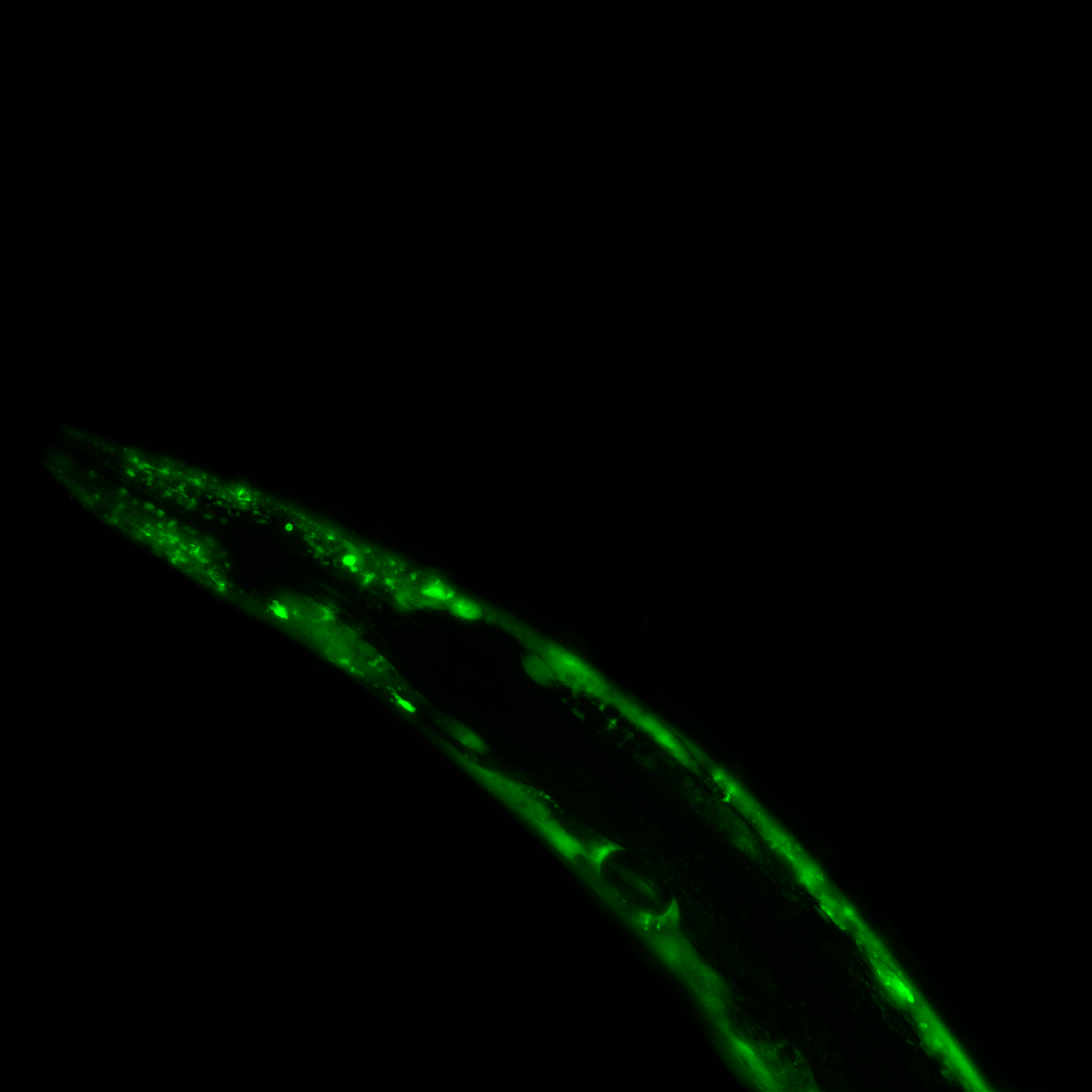

Supplement: Supplementary file 3 — Source Data for Expanded View and Appendix [file EMBR-24-e55556-s011.zip › EV_and_Appendix_Source_Data/EV_Figure_Source/Figure_EV2/Manuscript-EMBOR-2022-55556V3_SourceDataForFigureEV2A/unc1_1.tif]

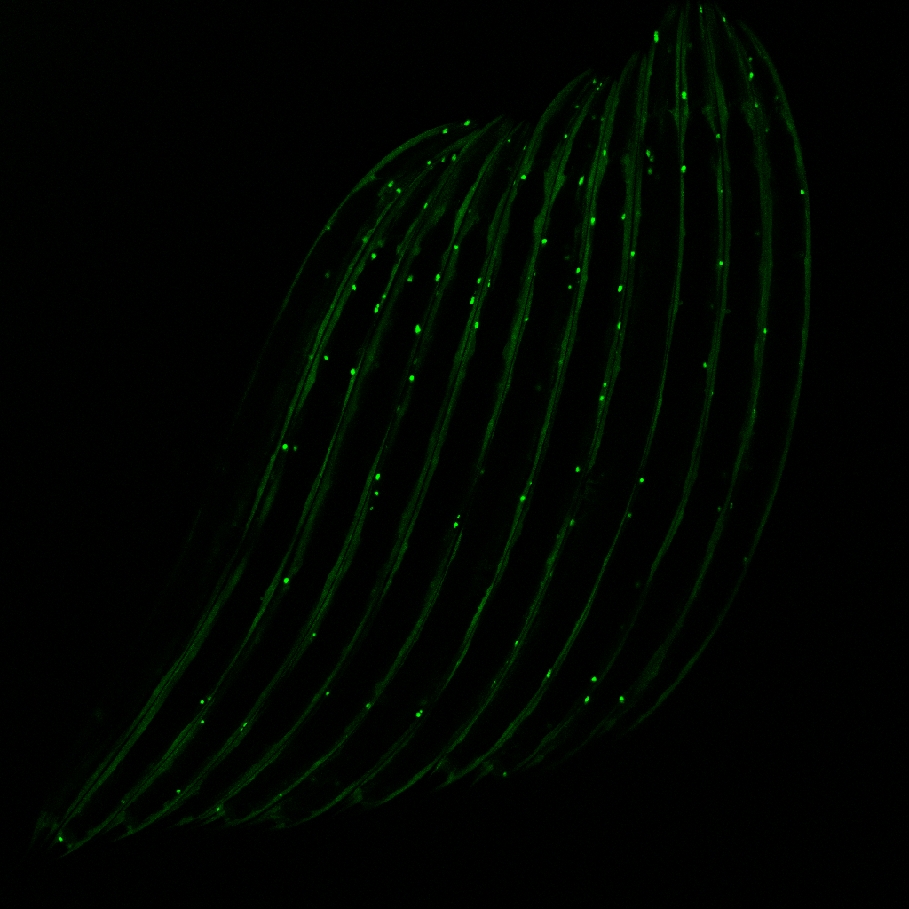

Supplement: Supplementary file 5 — Source Data for Figure 1 [file EMBR-24-e55556-s010.zip › Manuscript-EMBOR-2022-55556V1_SourceDataForFigure1A/Manuscript-EMBOR-2022-55556V3_SourceDataForFigure1A/40Q_3 2048Snapshot1.jpg]

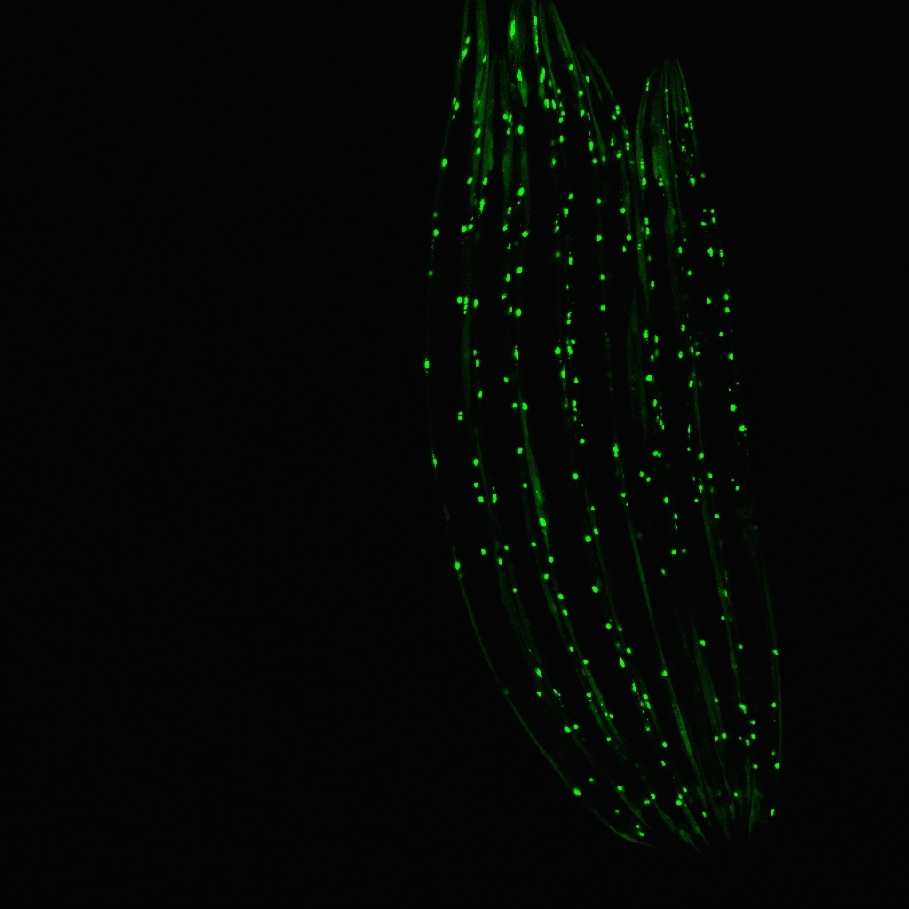

Supplement: Supplementary file 5 — Source Data for Figure 1 [file EMBR-24-e55556-s010.zip › Manuscript-EMBOR-2022-55556V1_SourceDataForFigure1A/Manuscript-EMBOR-2022-55556V3_SourceDataForFigure1A/40Q_unc_1_2 2048Snapshot1.jpg]

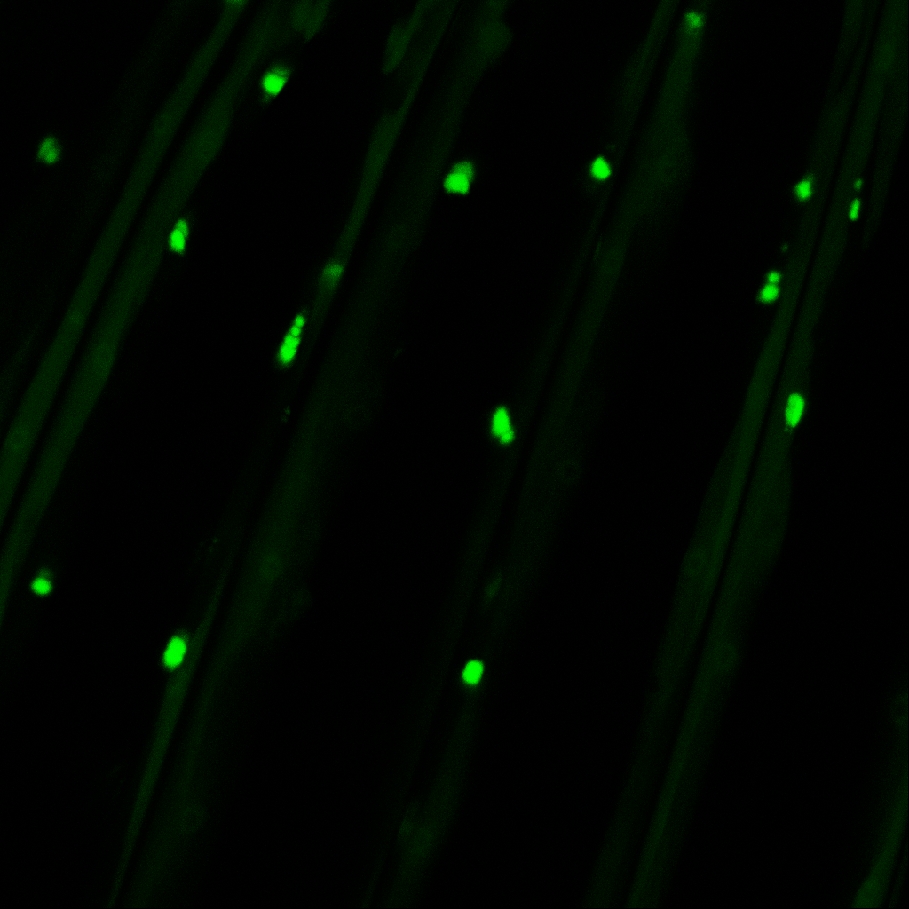

Supplement: Supplementary file 5 — Source Data for Figure 1 [file EMBR-24-e55556-s010.zip › Manuscript-EMBOR-2022-55556V1_SourceDataForFigure1A/Manuscript-EMBOR-2022-55556V3_SourceDataForFigure1A/zoom 40QSnapshot1.jpg]

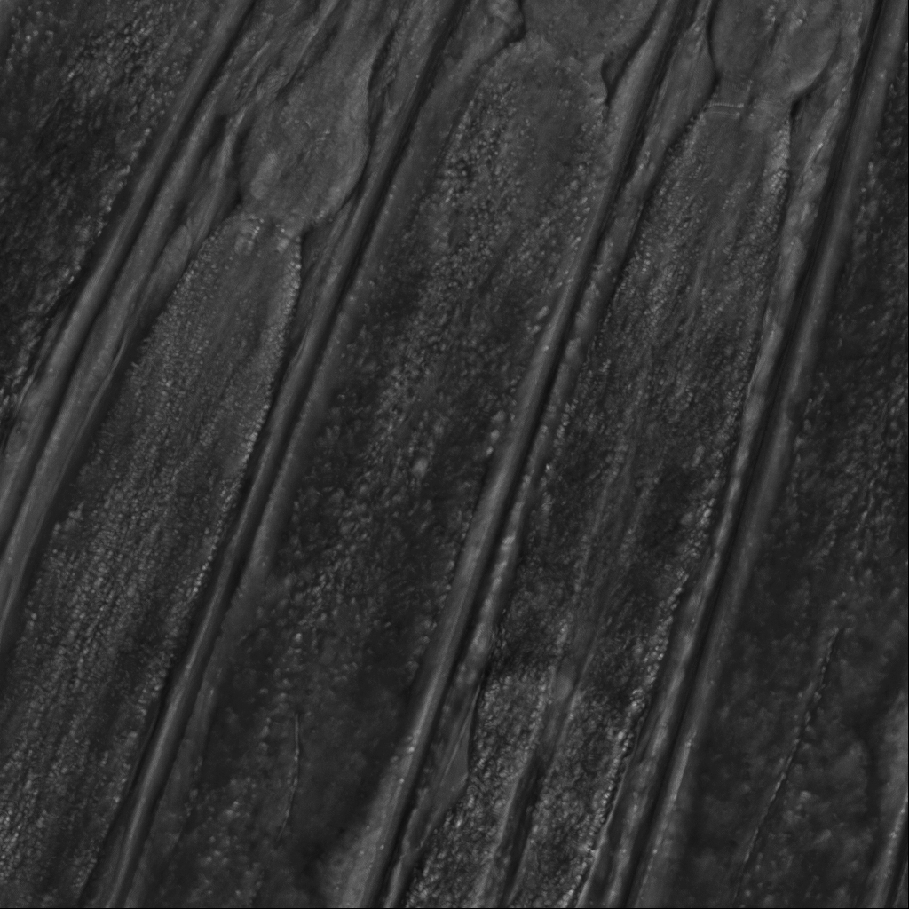

Supplement: Supplementary file 5 — Source Data for Figure 1 [file EMBR-24-e55556-s010.zip › Manuscript-EMBOR-2022-55556V1_SourceDataForFigure1A/Manuscript-EMBOR-2022-55556V3_SourceDataForFigure1A/zoom 40QSnapshot2.jpg]

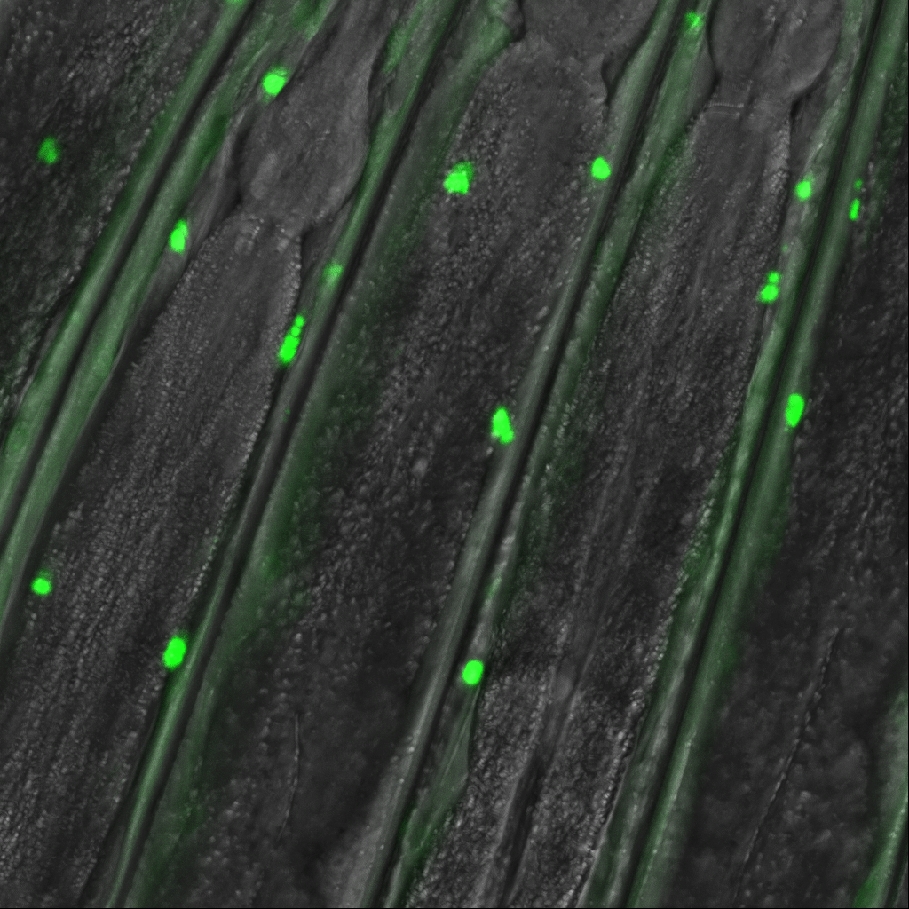

Supplement: Supplementary file 5 — Source Data for Figure 1 [file EMBR-24-e55556-s010.zip › Manuscript-EMBOR-2022-55556V1_SourceDataForFigure1A/Manuscript-EMBOR-2022-55556V3_SourceDataForFigure1A/zoom 40QSnapshot3.jpg]

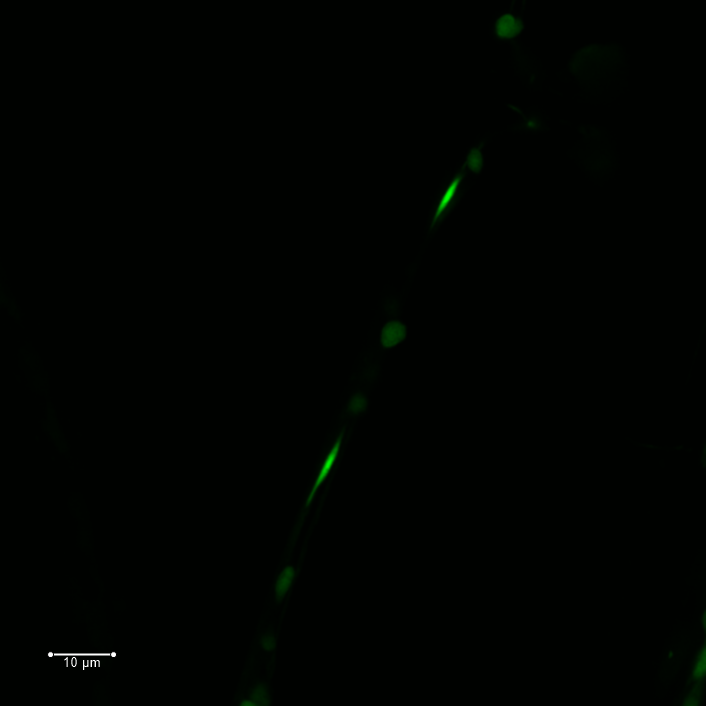

Supplement: Supplementary file 5 — Source Data for Figure 1 [file EMBR-24-e55556-s010.zip › Manuscript-EMBOR-2022-55556V3_SourceDataForFigure1E/Manuscript-EMBOR-2022-55556V3_SourceDataForFigure1E/40Q panneuronal.lif_Series004Snapshot3_ch00.tif]

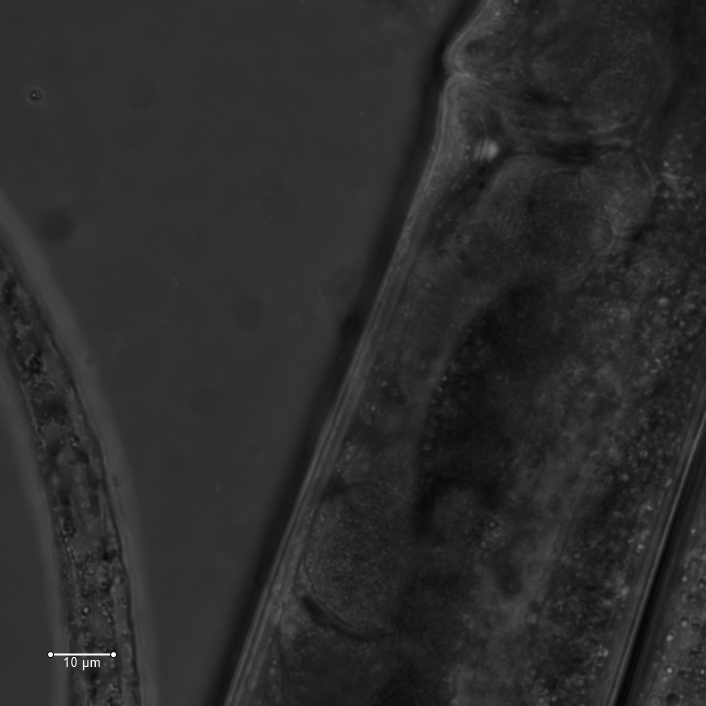

Supplement: Supplementary file 5 — Source Data for Figure 1 [file EMBR-24-e55556-s010.zip › Manuscript-EMBOR-2022-55556V3_SourceDataForFigure1E/Manuscript-EMBOR-2022-55556V3_SourceDataForFigure1E/40Q panneuronal.lif_Series004Snapshot4_ch00.tif]

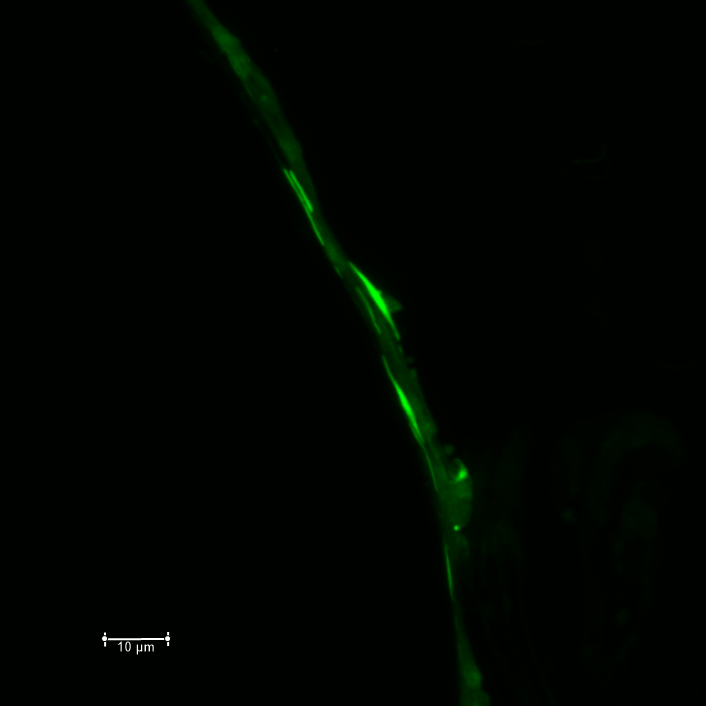

Supplement: Supplementary file 5 — Source Data for Figure 1 [file EMBR-24-e55556-s010.zip › Manuscript-EMBOR-2022-55556V3_SourceDataForFigure1E/Manuscript-EMBOR-2022-55556V3_SourceDataForFigure1E/40Q panneuronal_unc-1.lif_Series013Snapshot2_ch00.tif]

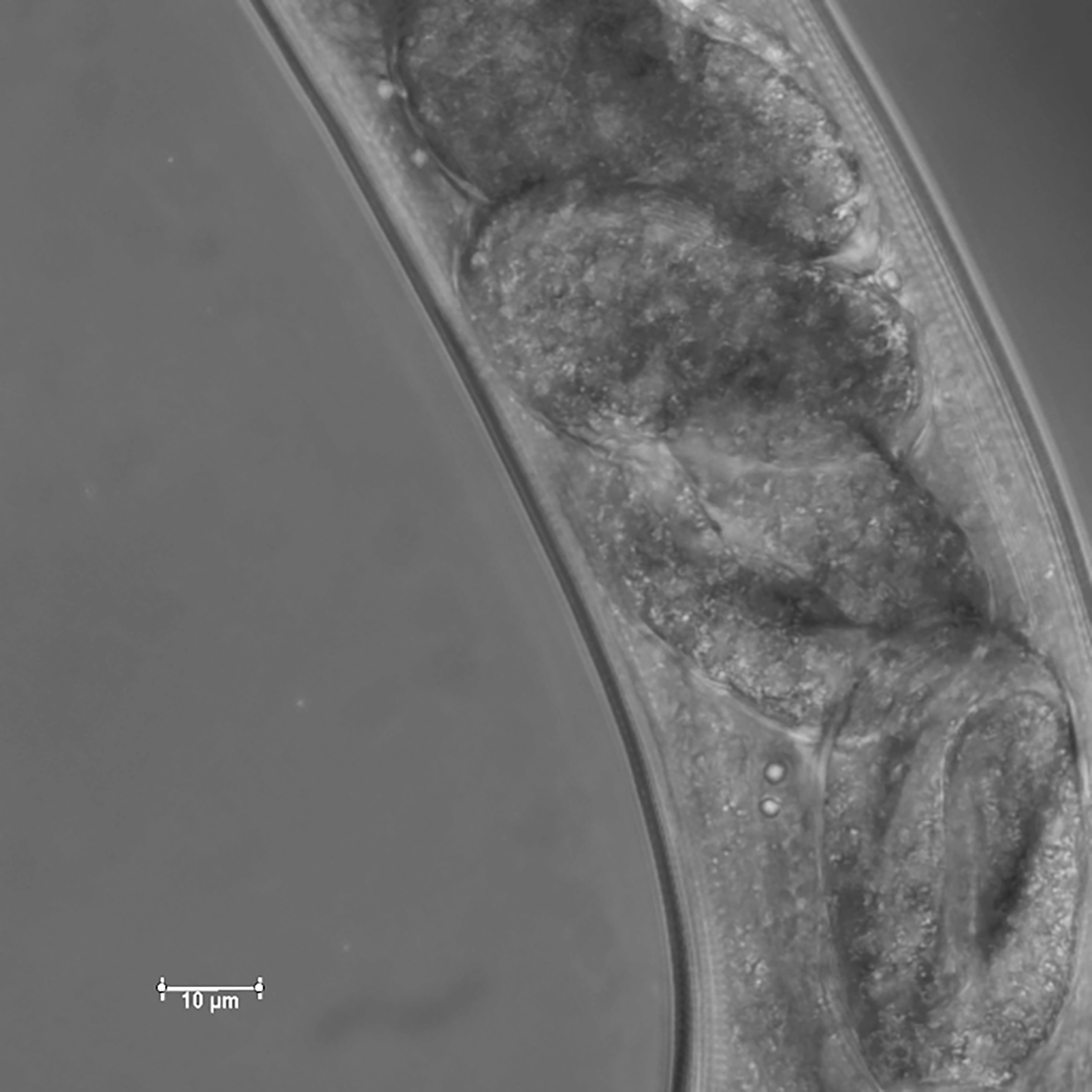

Supplement: Supplementary file 5 — Source Data for Figure 1 [file EMBR-24-e55556-s010.zip › Manuscript-EMBOR-2022-55556V3_SourceDataForFigure1E/Manuscript-EMBOR-2022-55556V3_SourceDataForFigure1E/40Q panneuronal_unc-1.lif_Series013Snapshot3_ch00.tif]

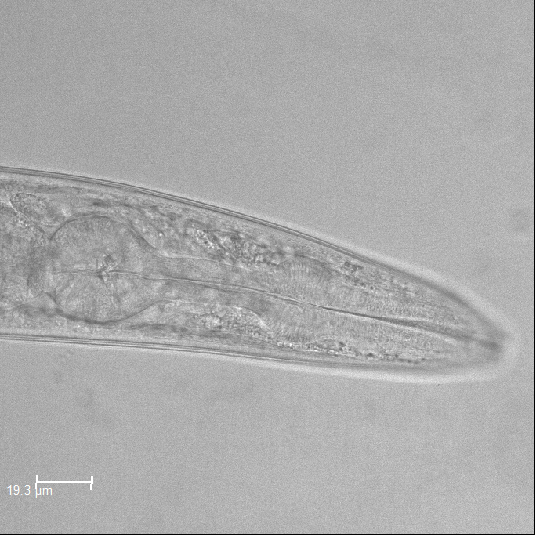

Supplement: Supplementary file 5 — Source Data for Figure 1 [file EMBR-24-e55556-s010.zip › Manuscript-EMBOR-2022-55556V3_SourceDataForFigure1G/Manuscript-EMBOR-2022-55556V3_SourceDataForFigure1G/alpha-synuclein_Series007Snapshot3_ch00.tif]

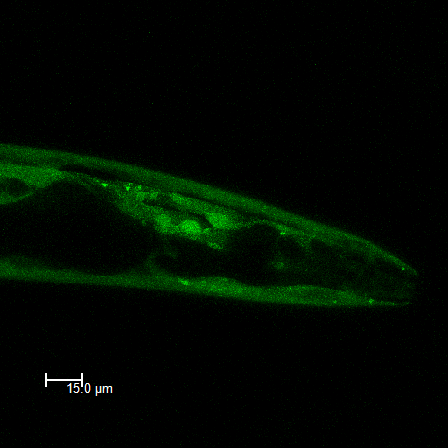

Supplement: Supplementary file 5 — Source Data for Figure 1 [file EMBR-24-e55556-s010.zip › Manuscript-EMBOR-2022-55556V3_SourceDataForFigure1G/Manuscript-EMBOR-2022-55556V3_SourceDataForFigure1G/alpha_synuclein_Series010Snapshot2_ch00.tif]

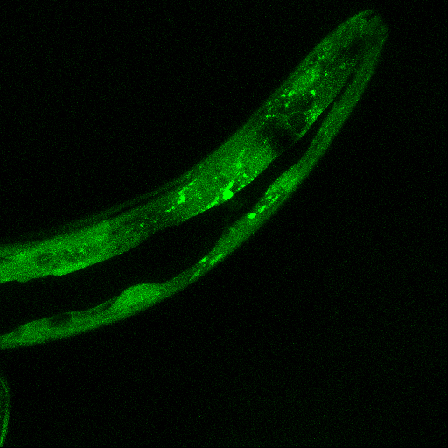

Supplement: Supplementary file 5 — Source Data for Figure 1 [file EMBR-24-e55556-s010.zip › Manuscript-EMBOR-2022-55556V3_SourceDataForFigure1G/Manuscript-EMBOR-2022-55556V3_SourceDataForFigure1G/alpha_synuclein_unc_1_Series013Snapshot1_ch00.tif]

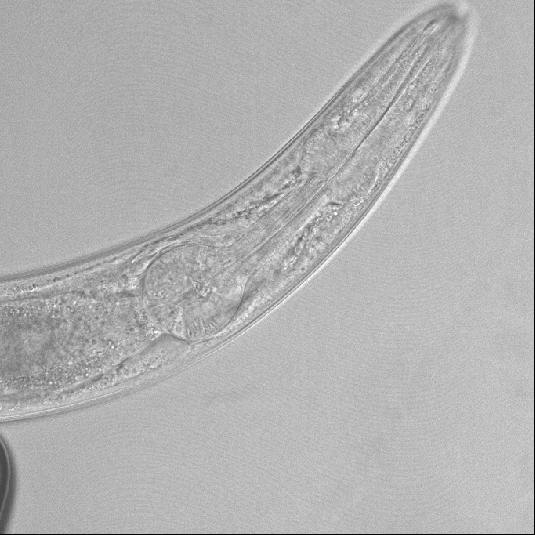

Supplement: Supplementary file 5 — Source Data for Figure 1 [file EMBR-24-e55556-s010.zip › Manuscript-EMBOR-2022-55556V3_SourceDataForFigure1G/Manuscript-EMBOR-2022-55556V3_SourceDataForFigure1G/alpha_synuclein_unc_1_Series014Snapshot2_ch00.tif]

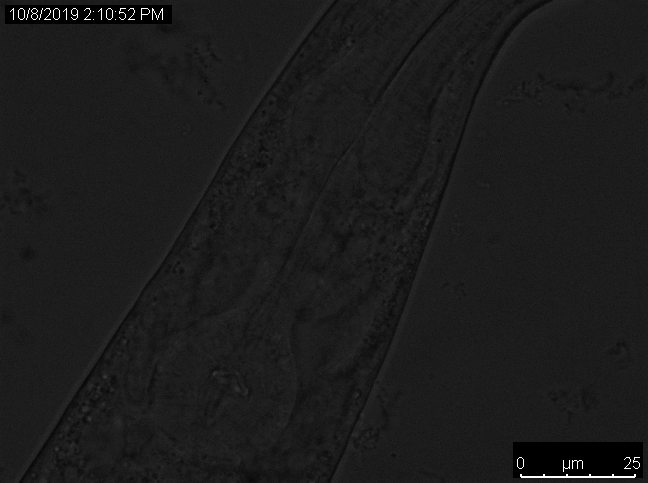

Supplement: Supplementary file 5 — Source Data for Figure 1 [file EMBR-24-e55556-s010.zip › Manuscript-EMBOR-2022-55556V3_SourceDataForFigure1I/Manuscript-EMBOR-2022-55556V3_SourceDataForFigure1I/Beta amyloid.lif_Image061_ch00.tif]

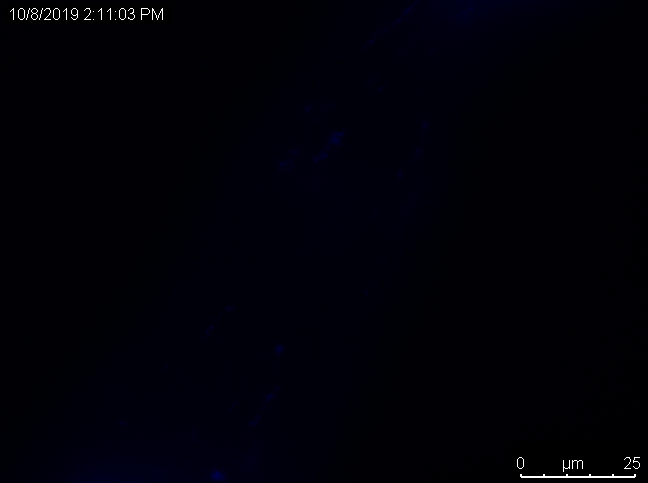

Supplement: Supplementary file 5 — Source Data for Figure 1 [file EMBR-24-e55556-s010.zip › Manuscript-EMBOR-2022-55556V3_SourceDataForFigure1I/Manuscript-EMBOR-2022-55556V3_SourceDataForFigure1I/Beta amyloid.lif_Image062_ch01.tif]

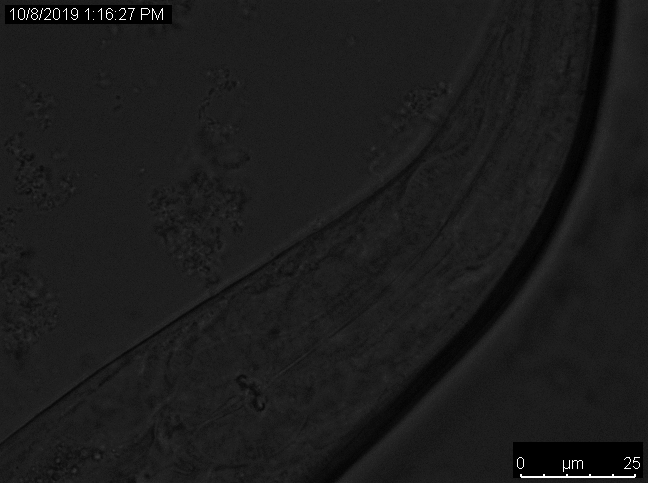

Supplement: Supplementary file 5 — Source Data for Figure 1 [file EMBR-24-e55556-s010.zip › Manuscript-EMBOR-2022-55556V3_SourceDataForFigure1I/Manuscript-EMBOR-2022-55556V3_SourceDataForFigure1I/beta amyloid_unc_1.lif_Image045_ch00.tif]

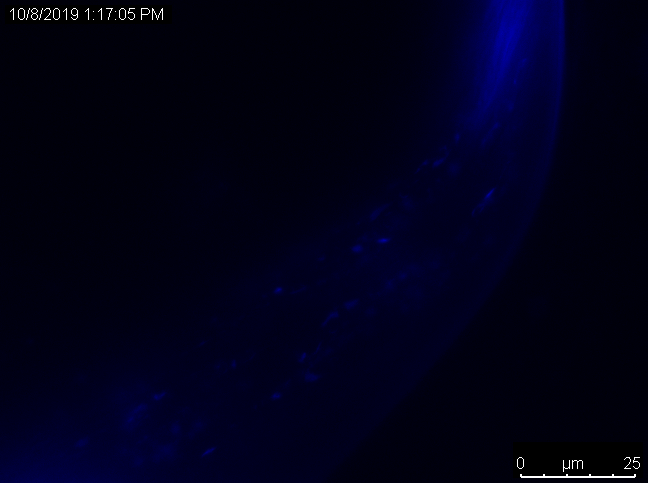

Supplement: Supplementary file 5 — Source Data for Figure 1 [file EMBR-24-e55556-s010.zip › Manuscript-EMBOR-2022-55556V3_SourceDataForFigure1I/Manuscript-EMBOR-2022-55556V3_SourceDataForFigure1I/beta amyloid_unc_1.lif_Image046_ch01.tif]
