# Supplementary material for: Changes in lipid metabolism driven by steroid signalling modulate proteostasis in C. elegans
Source: EMBO Rep. 2023 Apr 27;24(6):e55556. doi: 10.15252/embr.202255556 (PMC10240203; doi:10.15252/embr.202255556)
Supplement: Supplementary file 4 — PDF+ [file EMBR-24-e55556-s008.pdf]

Article

SOURCE DATA TRANSPARENT PROCESS OPEN ACCESS

EMBO reports

Changes in lipid metabolism driven by steroid signalling modulate proteostasis in *C. elegans*

Ana P Gómez-Escribano[1,2,3], Carlos Mora-Martínez[4], Marta Roca[5], Denise S Walker[6], Joaquín Panadero[7], Maria D Sequedo[1,2,3], Ratni Saini[8], Hans-Joachim Knölker[8], Jose Blanca[9], Juan Burguera[10], Agustin Lahoz[5,11], Joaquin Cañizares[9], José M Millán[1,2,3], Nick O Burton[12], William R Schafer[6] & Rafael P Vázquez-Manrique[1,2,3,*]

Abstract

Alzheimer's, Parkinson's and Huntington's diseases can be caused by mutations that enhance protein aggregation, but we still do not know enough about the molecular players of these pathways to develop treatments for these devastating diseases. Here, we screen for mutations that might enhance aggregation in *Caenorhabditis elegans*, to investigate the mechanisms that protect against dysregulated homeostasis. We report that the stomatin homologue UNC-1 activates neurohormonal signalling from the sulfotransferase SSU-1 in ASJ sensory/endocrine neurons. A putative hormone, produced in ASJ, targets the nuclear receptor NHR-1, which acts cell autonomously in the muscles to modulate polyglutamine repeat (polyQ) aggregation. A second nuclear receptor, DAF-12, functions oppositely to NHR-1 to maintain protein homeostasis. Transcriptomics analyses of *unc-1* mutants revealed changes in the expression of genes involved in fat metabolism, suggesting that fat metabolism changes, controlled by neurohormonal signalling, contribute to protein homeostasis. Furthermore, the enzymes involved in the identified signalling pathway are potential targets for treating neurodegenerative diseases caused by disrupted protein homeostasis.

Keywords *Caenorhabditis elegans*; fat metabolism; nuclear receptors; protein aggregation; steroid hormone signalling
Subject Categories Metabolism; Molecular Biology of Disease; Neuroscience
DOI 10.15252/embr.202255556 | Received 8 June 2022 | Revised 2 March 2023 | Accepted 22 March 2023 | Published online 27 April 2023
EMBO Reports (2023) 24: e55556

Introduction

Many neurodegenerative diseases, such as Huntington's (HD), Alzheimer's and Parkinson's diseases, and Amyotrophic lateral sclerosis (ALS) are caused by toxic protein aggregates that stem from incorrect protein folding (Valastyan & Lindquist, 2014). In non-disease states, this aggregation is prevented via natural protein homeostasis mechanisms that ensure an adequate expression of proteins, proper folding and localization of these molecules, and the removal of misfolded proteins. Many pathways and molecules have been shown to influence this maintenance of appropriately folded proteins and their location in the cellular environment. For example, deleterious mutations in genes encoding components of chemical synapses accelerate the decline of protein homeostasis in postsynaptic muscle cells and contribute to the progression of age-related disorders (Garcia *et al*, 2007). However, though much is known about these pathways, we still do not know enough about all of the molecular players and their mechanisms to develop treatments for these devastating diseases.

In a properly functioning homeostasis system, the accumulation of a considerable number of misfolded proteins would induce cytoplasmic stress by poisoning the machinery of the autophagic and proteasomal systems. This in turn activates the unfolded protein response (UPR) pathways in the endoplasmic reticulum, mitochondria and cytosol (Almanza *et al*, 2019). UPR signals activate nuclear transcription factors that induce the expression of genes associated with folding and protein degradation, autophagy and other protective pathways (Almanza *et al*, 2019). Proteostasis can also be modulated by the metabolic status of the organism and has been shown to be connected to lipid metabolism (Steinbaugh *et al*, 2015;

1 Laboratory of Molecular, Cellular and Genomic Biomedicine, Instituto de Investigación Sanitaria La Fe, Valencia, Spain
2 Centro de Investigación Biomédica en Red de Enfermedades Raras (CIBERER), Valencia, Spain
3 Joint Unit for Rare Diseases IIS La Fe-CIPF, Valencia, Spain
4 Institute of Biotechnology, University of Helsinki, Helsinki, Finland
5 Unidad Analítica, Instituto de Investigación Sanitaria La Fe, Valencia, Spain
6 Neurobiology Division, MRC Laboratory of Molecular Biology, Cambridge Biomedical Campus, Cambridge, UK
7 Unidad Genómica-Bioinformática, Instituto de Investigación Sanitaria La Fe, Valencia, Spain
8 Fakultät Chemie, Technische Universität Dresden, Dresden, Germany
9 Instituto Universitario de Conservación y Mejora de la Agrodiversidad Valenciana, Valencia, Spain
10 Department of Neurology, Hospital Universitario y Politécnico La Fe, Valencia, Spain
11 Unidad de Biomarcadores y Medicina de Precisión, Unidad Analítica, Instituto de Investigación Sanitaria, Fundación Hospital La Fe, Valencia, Spain
12 Van Andel Institute, Grand Rapids, MI, USA
*Corresponding author. Tel: +34 96 1246678/1246685; E-mail: rafael_vazquez@iislafe.es

© 2023 The Authors. Published under the terms of the CC BY 4.0 license

Webster *et al*, 2017; Lee *et al*, 2019; Higuchi-Sanabria *et al*, 2020; Joshi *et al*, 2021). For example, inhibiting the genes that encode amino-acyl tRNA synthetases increases fat production and activates the proteasomal AMPK network to extend survival under starvation conditions (Webster *et al*, 2017). In addition, the lipid homeostasis regulator MDT-15 is required for maintaining HSF-1-dependent proteostasis at low temperatures in *Caenorhabditis elegans* expressing a polyQ (*35Q::YFP*) (Lee *et al*, 2019). Lipids can also modulate proteostasis via lipid depletion through overexpression of XBP-1s in serotonergic, but not dopaminergic, neurons (Higuchi-Sanabria *et al*, 2020). Moreover, XBP-1s remodels lipid metabolism by non-cell autonomous signalling, which decreases triglyceride and increases oleic acid levels (Imanikia *et al*, 2019). In addition, the folding of proteins containing expanded polyQs in muscle cells can be regulated by chemical synaptic functions in neurons (Garcia *et al*, 2007; Silva *et al*, 2013). For example, a small increase in physiological cholinergic signalling at motor synapses induces a calcium-dependent activation of muscular HSF-1, which in turn activates the expression of chaperones that protect against protein aggregation (Silva *et al*, 2013). Additionally, Taylor & Dillin (2013) described another non–cell autonomous mechanism by XBP-1 to regulate UPR pathways and longevity in *C. elegans*. However, despite these and similar observations, the mechanisms by which neuronal signalling controls protein aggregation in distant tissues remains unknown.

Nuclear receptors comprise a group of transcription factors that regulate the expression of genes in a ligand–binding-dependent manner (Chawla, 2001; Antebi, 2015). These ligands are usually lipid molecules, such as steroid hormones, vitamins (D_3 and A), metabolites, and xenobiotics (Sever & Glass, 2013). Sometimes required for receptor binding and promoting hormonal signalling to distal tissues, these ligands require processing by sulfotransferases, which add a sulphate moiety, and sulfatases, which remove the sulphate. In terms of lipid metabolism, Joshi *et al* (2021) claim that sensory neurons secrete biogenic amines to modulate lipid signalling, which in turn activates UPR pathways to maintain proteostasis. This work is evidence that sensory neurons can release signalling molecules to regulate gene expression.

Caenorhabditis elegans has 284 nuclear receptor genes, compared to 48 in humans (Maglich *et al*, 2001). One of the best-known nuclear receptors in *C. elegans*, DAF-12, regulates lipid metabolism, lifespan and development (Antebi *et al*, 2000; Ludewig, 2004; Wang *et al*, 2015) through the dafachronic acid hormones. This control depends on the tight modulation of the expression of genes encoding key metabolic enzymes, which includes negative feedback loops operated by their enzymatic products (Bi *et al*, 2018). For example, NHR-49, a homologue of the Hepatocyte Nuclear Factor 4-α (HNF4α) (Goh *et al*, 2018), has a crucial role in the regulation of lipid metabolism, longevity and nutrient response (Gilst *et al*, 2005). Following activation, nuclear receptors dimerise, producing homo- and heterodimers, and bind DNA domains in the vicinity of promoters to regulate gene expression, sometimes in opposite manners depending on the nuclear receptor partner. For instance, heterodimeric NHR-49/NHR-80 promotes lipid desaturation while NHR-49/NHR-66 blocks sphingolipid metabolism and lipid remodelling (Pathare *et al*, 2012). Among the lipids regulated by these nuclear receptors, oleic acid has been shown to promote longevity in germline ablated mutants (Goudeau *et al*, 2011). This lipid is also able to enhance proteostasis, in worms expressing polyQs, via XBP-1 signalling (Imanikia *et al*, 2019). Oleic acid is synthesised by several key enzymes, that are encoded in the *C. elegans* genome: FAT-6 and FAT-7, whose expression is regulated by NHR-80 (Watts & Browse, 2002). Overall, though extracellular signalling has been shown to modulate protein homeostasis the upstream and downstream processes involved in this regulation are not completely understood. Nor is it clear whether other synaptic-related phenotypes that may influence protein homeostasis.

Here, we studied these pathways in *C. elegans* via a chemical mutagenesis screen of worms expressing polyQs in muscle cells, which allowed us to isolate a loss-of-function allele of *unc-1* that enhances protein aggregation and alters motor coordination. We show that the stomatin homologue UNC-1 functions in neurons, likely by modulating electrical synapses, to regulate protein homeostasis by non-cell autonomous signalling. Disrupting *unc-1* disrupts a key electrical synapse, likely then causing the secretion of excess sulphated signal that activates NHR-1 to downregulate genes encoding enzymes for fat metabolism, which in turn disrupts proteostasis. We also show that signalling through the well-known DAF-12 functions antagonistically to NHR-1 to control protein aggregation. These results provide the first evidence that nuclear receptors modulate polyQ aggregation by remodelling lipid metabolism. Moreover, some of the enzymes involved in the NHR–1-activating signalling pathway are potential druggable targets, which may be used to treat neurodegenerative diseases caused by protein homeostasis disruption.

Results

A forward genetic screen identifies *unc-1* as a modulator of polyQ aggregation

To expand our knowledge of the signalling pathways controlling protein homeostasis, we performed a chemical mutagenesis screen for genes regulating protein aggregation in a worm model of polyQ diseases. For this screen, we used animals that express a transgene containing a 40-glutamine repeat fused to a yellow fluorescent protein (40Q::YFP) in muscle cells. In these animals, 40Q aggregates in an age-dependent manner and forms inclusion bodies that can be observed and counted under a dissecting microscope. Though several mutants were identified from this screen (Appendix Table S1), only one matched our criteria, that carrying the *vlt10* allele in *unc-1* (Appendix Fig S1A and B, Appendix Supplementary Methods), which substantially enhanced aggregation without affecting 40Q transgene expression (Fig 1A and B, Appendix Fig S1A and B). In addition to enhanced polyQ aggregation, the *unc-1(vlt10)* animals showed an uncoordinated phenotype, suggesting that this mutation may affect the synaptic function of the nervous system and/or the muscle cells, both tissues in which *unc-1* is expressed (Rajaram *et al*, 1998, 1999; Sedensky *et al*, 2001; Chen *et al*, 2007).

Depletion of *unc-1* enhances polyQ aggregation

To confirm that the loss of function of *unc-1* is responsible for the enhanced aggregation phenotype of *unc-1(vlt10)* mutants, we examined the effect of other mutant alleles of *unc-1* (*e719* and *e1598*) on animals expressing two polyQs with different repeat lengths, 35Q

 © 2023 The Authors

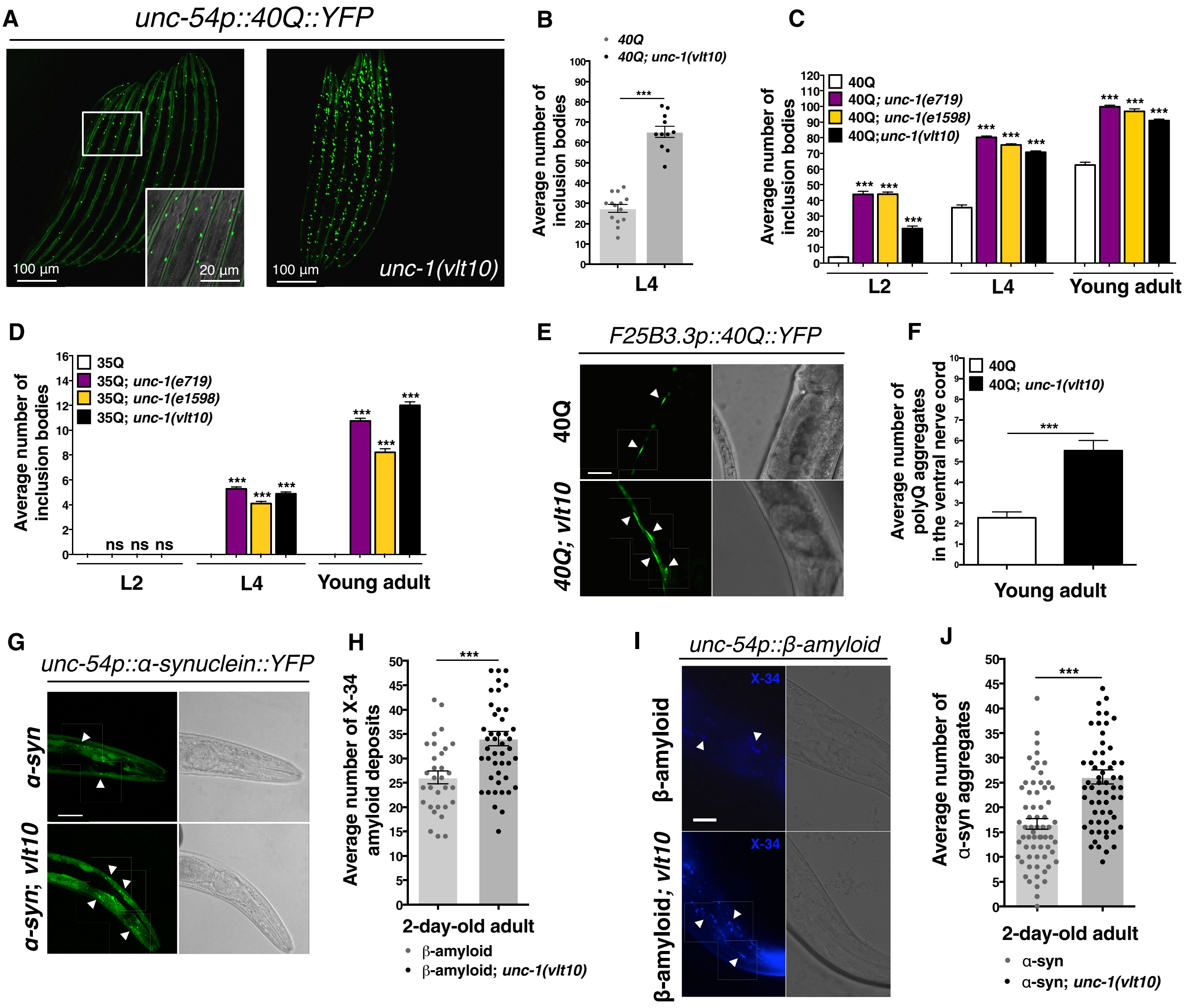

Figure 1. UNC-1 modulates protein aggregation.

- A Representative confocal microscopy images of L4 40Q and 40Q; *unc-1(vlt10)* mutant worms. The inset (20× further zoom) shows the shape and pattern of the inclusion bodies in muscle cells (white arrows).
- B The mean number of polyQ inclusion bodies in homozygous *unc-1(vlt10)* animals.
- C The mean number of polyQ inclusion bodies in 40Q animals carrying *e719* and *e1598* alleles of *unc-1* at different stages (L2, L4 and young adult).
- D The mean number of polyQ inclusion bodies in 35Q animals carrying *e719* and *e1598* alleles of *unc-1* at different stages (L2, L4 and young adult).
- E Representative images from fluorescence microscopy showing neuronal aggregates of polyQs (white arrows) in the ventral nerve cord of F25B3.3p::40Q::YFP and F25B3.3p::40Q::YFP; *unc-1 (vlt10)* young adult animals.
- F The mean number of neuronal aggregates of polyQs in (E).
- G Representative images from confocal microscopy showing the area of the muscle tissue between the two pharyngeal bulbs where α-synuclein aggregates are located (white arrows).
- H The counted average number of α-synuclein aggregates in (G).
- I Representative fluorescence microscopy images of the area between the two pharyngeal bulbs highlighting amyloid deposits (white arrows).
- J The counted average number of amyloid deposits in (I).

Data information: All plotted data show the mean ± standard error of the mean (SEM). At least 30 animals were tested for each condition for all graphs except for (G), which used 60 animals. Each analysis has been reproduced at least three independent times. ***$P < 0.001$; ns: not significant. The analyses in (B, F, H and J) were done using a Mann–Whitney *U*-test, and those in (C and D) were done using a one-way ANOVA with *post-hoc* Tukey test. Scale bar: 20 µm unless otherwise specified.
Source data are available online for this figure.

and 40Q (which we will refer to as 35Q or 40Q animals). In both cases, analysis of the inclusion bodies produced by polyQ aggregation showed that mutations in *unc-1* (*e719* and *e1598*) phenocopy the aggregation pattern of *vlt10* worms (Fig 1C and D) without altering *40Q::YFP* transgene expression (Appendix Fig S1C and D). In addition, we investigated whether *unc-1(vlt10)* would modify the late aggregation phenotype of a strain expressing 35Q fused to YFP (35Q::YFP) in body wall muscles, which produces both a later and weaker phenotype than animals expressing 40Q and inclusion bodies at late adult stages (Fig 1D). In 35Q; *unc-1(vlt10)* animals, the *vlt10* allele enhanced and accelerated the phenotype of these animals to a similar extent as other *unc-1* alleles, *e179* and *e1598* (Fig 1D). These results indicate that *unc-1* modulates polyQ aggregation in muscle cells.

As *unc-1* is expressed in neurons, we investigated whether *vlt10* affected polyQ aggregation in this tissue using a worm strain expressing 40Q across the entire nervous system. We introduced the *unc-1 (vlt10)* mutation, and as expected, *unc-1(vlt10)* increased neuronal aggregate formation in the ventral nerve cord of young adults, further demonstrating the requirement of *unc-1* in protein homeostasis (Fig 1E and F). Altogether, these data indicate that *unc-1* is required to prevent the aggregation of polyQ-containing proteins, both in muscle cells and neurons.

unc-1(vlt10) enhances α-synuclein and β-amyloid aggregation

Having shown that *unc-1* regulates polyQ aggregation, we tested whether its function is polyQ-specific or if it affects other aggregation-prone proteins, such as α-synuclein and β-amyloid that aggregate in brains of Parkinson's and Alzheimer's patients, respectively. To do this, we introduced the *unc-1(vlt10)* allele into transgenic worms expressing α-synuclein::YFP (referred to as α-syn) and worms expressing human β-amyloid in body wall muscles. In contrast with worms expressing polyQs, which show aggregation early in the life cycle of worms, both α-syn and β-amyloid aggregates do not appear until later stages of development (2-day-old adults). Quantifying the aggregate numbers showed that the loss of *unc-1* function increased both α-syn and β-amyloid aggregation in day 2 adults compared with controls (Fig 1G–J). These data suggest that *unc-1*, rather than being polyQ-specific, is a general modulator of protein homeostasis.

unc-1 required in nervous system to maintain non–cell autonomous protein homeostasis

To investigate whether UNC-1 also modulates muscle aggregation in a cell-autonomous manner, as it is expressed throughout both the nervous system and muscle cells, we examined whether the aggregation phenotype of *40Q*; *unc-1(vlt-10)* animals could be rescued via the expression of *unc-1* cDNA in the muscles. Indeed, expressing *unc-1* under the control of the muscle-specific *myo-3* promoter (*myo-3p*) appeared to partially rescue polyQ aggregation, since we observed a decrease in YFP-labelled aggregates (Appendix Fig S2A and B, Appendix Table S2). However, this was likely an artefact of altered polyQ expression (Appendix Fig S2A and B).

In contrast, when we expressed *unc-1* across the nervous system using the pan-neuronal promoter of the *rab-3* gene, *unc-1(vlt10)* animals had substantially reduced polyQ aggregation in all stages (Fig 2A), with no effect on the expression of the transgene (Fig 2A). To confirm these results, we knocked-down *unc-1* expression in a tissue-specific manner using RNAi by expressing double-stranded RNAs against the gene across the entire nervous system (*rab-3* promoter) using transgenesis (Appendix Fig S8). As a negative control, we used RNAi against the ampicillin resistance gene (*AMP*^{r}). This silencing of *unc-1* across the nervous system increased the number of inclusion bodies compared with 40Q and 40Q; *AMP*^{r}*(RNAi)* animals (Fig 2B), further suggesting that polyQ aggregation in muscle cells is controlled from neuronal *unc-1* expression. Finally, to further demonstrate that *unc-1* is acting neuronally, we disrupted *unc-1* function across the nervous system by overexpressing a cDNA containing the *n494* dominant allele of *unc-1*, again using the *rab-3* promoter to drive expression in all neurons. These animals exhibited a similar level of inclusion bodies to 40Q; *unc-1 (vlt10)* animals (Fig 2C). Moreover, overexpression of the wild type *unc-1* gene, which does not alter protein aggregation dynamics, showed that it is not an overload of UNC-1 that increases polyQ aggregation (Fig 2C). Altogether these results show that UNC-1 acts in neurons to modulate polyQ aggregation in muscle cells.

unc-1 is required in IL2 neurons to modulate polyQ aggregation

Previous reports showed a genetic interaction between *unc-1* and *ssu-1* relating to anaesthetic sensitivity and motor phenotypes in worms (Carroll *et al*, 2006). Since *ssu-1* is expressed exclusively in a single class of neurons, a pair of amphid sensory neurons called ASJ (Carroll *et al*, 2006), we speculated that *unc-1* may be required in this cell to modulate polyQ aggregation. To test this, we tried to rescue *unc-1(vlt10)* by expressing the *unc-1* cDNA under the control of the *trx-1* promoter, which expresses only in ASJ neurons. However, we did not observe any change in 40Q aggregation (65.2 $\pm$ 9.1 (*unc-1* mutants) vs. 64.1 $\pm$ 7.5 (rescued *unc-1* mutants), Mean $\pm$ SD).

Since UNC-1 is known to regulate electrical synapses (Chen *et al*, 2007), we then hypothesised that *unc-1* may be required in neurons that connect with ASJ through gap junctions. As the electrical and chemical synaptic connections of the entire *C. elegans* nervous system have been precisely characterised (Cook *et al*, 2019), we know that ASJ makes electrical synapses with one of the six inner labial IL2 sensory neurons, IL2L, and one of the two PVQ posterior interneurons, PVQR (Cook *et al*, 2019). Thus, we disrupted the function of *unc-1* specifically in ASJ, IL2 and PVQ using RNAi induced from transgenes. In agreement with our failure to rescue *unc-1(vlt10)* in ASJ, knockdown of *unc-1* in ASJ had no effect, supporting the idea that ASJ is not the site of function. In contrast, knockdown in the IL2 neurons significantly increased polyQ aggregation to the same level as the *unc-1* mutants (Fig 2D), suggesting that *unc-1* acts in the IL2s to prevent polyQ aggregation. Surprisingly, silencing *unc-1* in PVQ neurons had the opposite effect, inducing a mild reduction of polyQ aggregation. In both cases, this suggests that interactions of these neurons with ASJ could modulate polyQ aggregation (Fig 2D).

To confirm the role of the IL2s, we tested for cell-specific rescue by reintroducing the cDNA of *unc-1* in the IL2 neurons of 40Q; *unc-1(vlt10)* animals using two different IL2-specific promoters, *oig-1p* and *osm-3p*. Though the expression patterns of these promoters cover several neurons, they overlap exclusively in IL2 neurons, meaning that the RNAi will occur only in IL2 neurons. These

 © 2023 The Authors

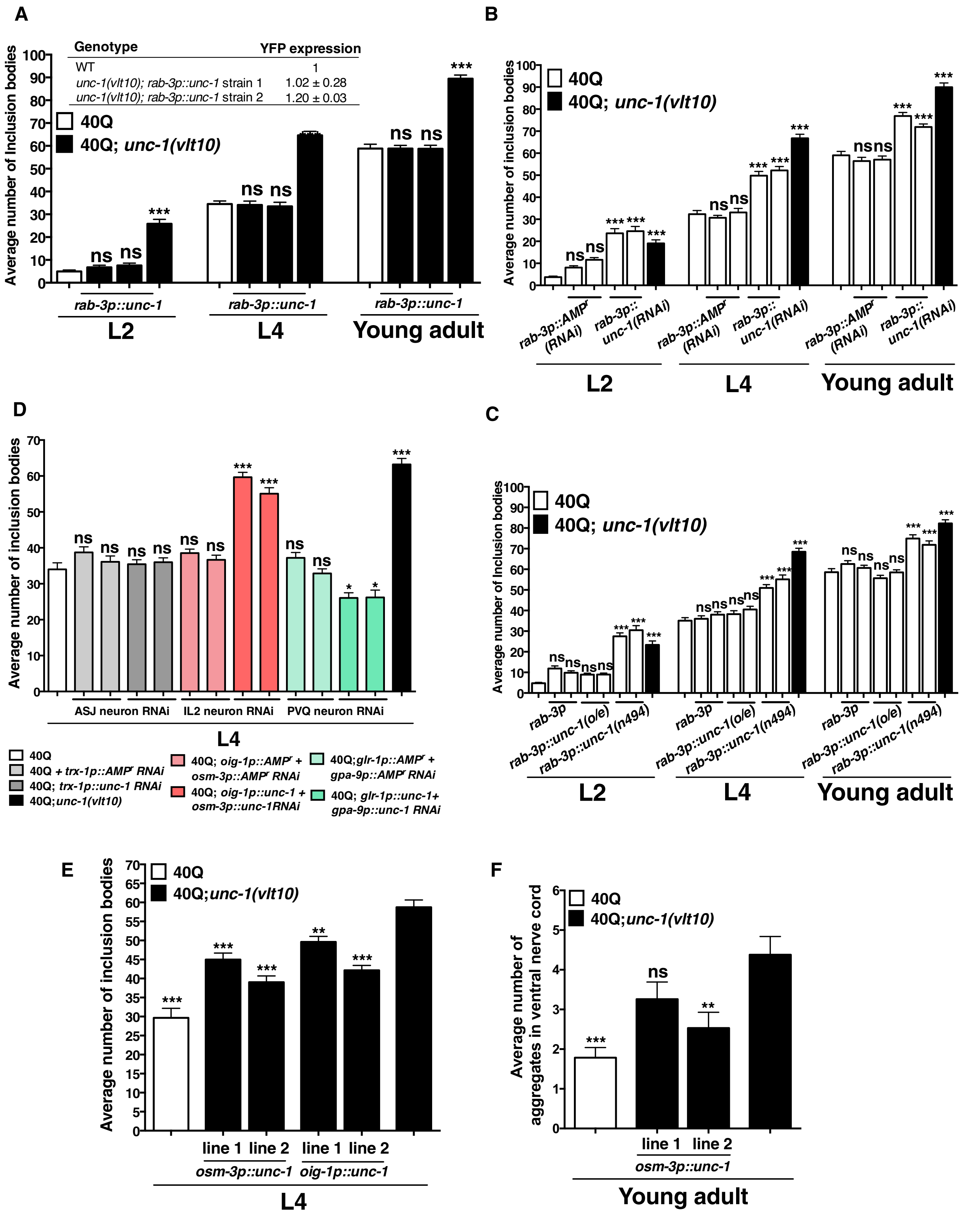
A
Genotype YFP expression
WT 1
unc-1(vlt10); rab-3p::unc-1 strain 1 1.02 ± 0.28
unc-1(vlt10); rab-3p::unc-1 strain 2 1.20 ± 0.03
40Q
40Q; unc-1(vlt10)
Average number of Inclusion bodies
rab-3p::unc-1
L2
L4
Young adult
B
40Q
40Q; unc-1(vlt10)
Average number of inclusion bodies
rab-3p::AMP^r (RNAi)
rab-3p:: unc-1(RNAi)
L2
L4
Young adult
D
Average number of inclusion bodies
ASJ neuron RNAi
IL2 neuron RNAi
PVQ neuron RNAi
L4
40Q
40Q + trx-1p::AMP^r RNAi
40Q; trx-1p::unc-1 RNAi
40Q;unc-1(vlt10)
40Q; oig-1p::AMP^r + osm-3p::AMP^r RNAi
40Q; oig-1p::unc-1 + osm-3p::unc-1RNAi
40Q;glr-1p::AMP^r + gpa-9p::AMP^r RNAi
40Q; glr-1p::unc-1+ gpa-9p::unc-1 RNAi
C
40Q
40Q; unc-1(vlt10)
Average number of Inclusion bodies
rab-3p
rab-3p::unc-1(o/e)
rab-3p::unc-1(n494)
L2
L4
Young adult
E
40Q
40Q;unc-1(vlt10)
Average number of inclusion bodies
line 1 line 2 line 1 line 2
osm-3p::unc-1 oig-1p::unc-1
L4
F
40Q
40Q;unc-1(vlt10)
Average number of aggregates in ventral nerve cord
line 1 line 2
osm-3p::unc-1
Young adult

Figure 2.

Figure 2. UNC-1 is required in IL2 neurons to modulate protein homeostasis in muscle cells and neurons.

A The cDNA of *unc-1* was expressed in the whole nervous system of worms, and the rescue of polyQ aggregation in *unc-1* mutants was measured at several stages (L2, L4 and young adult) by counting the number inclusion bodies per animal. We produced two independent transgenic stable lines (line 1 and line 2), carrying the *rab-3p::unc-1*(cDNA) transgene. The inserted table shows the expression of the *40Q::YFP* transgene as determined by qRT–PCR using a specific probe for YFP and normalised to *pmp-3* gene.

B The average number of polyQ inclusion bodies in muscle cells after the tissue-specific expression of RNAi against *unc-1* in the nervous system. We expressed dsRNA of *unc-1* specifically in neurons (using promoter of *rab-3*) of 40Q::YFP worms, to evaluate polyQ inclusion bodies after silencing.

C The average number of polyQ inclusion bodies in muscle cells, in the presence of overexpressed (o/e) *unc-1*, and its dominant negative allele *n494* in the nervous system.

D The average number of polyQ inclusion bodies in muscle cells, after the expression of RNAi against *unc-1*, in ASJ, IL2 and PVQ neurons using specific promoters or combination of them.

E The average number of polyQ inclusion bodies in muscle cells after the cDNA of *unc-1* was reintroduced in *unc-1(vlt10)* mutants expressing 40Q::YFP in muscle cells using the promoters from either *osm-3* or *oig-1*, separately, which would express the cDNA in some neurons, including IL2. We produced two independent transgenic stable lines (line 1 and line 2), carrying the *osm-3p::unc-1(cDNA)* transgene, and two independent transgenic stable lines (line 1 and line 2) carrying *oig-1p::unc-1(cDNA)* transgene. Comparisons are with *unc-1(vlt10)*.

F The same extrachromosomal arrays as in (E), that drive the expression of *osm-3p::unc-1(cDNA)* in IL2 neurons, were introduced in *unc-1(vlt10)* mutants, that express 40Q::YFP in the whole nervous system. Then, we analysed the average number of ventral nerve cord aggregates. Comparisons are with *unc-1(vlt10)*.

Data information: Bars indicate mean ± SEM. Thirty animals were tested per strain and/or condition. Each analysis has been reproduced at least three independent times. ***$P < 0.001$; **$P < 0.01$; *$P < 0.05$; ns: not significant, as calculated using one-way ANOVA with *post-hoc* Tukey test.
Source data are available online for this figure.

animals showed reduced polyQ aggregation in muscle cells and neurons (Fig 2E and F). Altogether, these data confirmed that loss of function of *unc-1(vlt10)* from IL2 neurons, which are electrically coupled to the ASJ neuron, modulates polyQ aggregation. UNC-1 may also function by modulating gap junctions (Appendix Table S3, Appendix Figs S3 and S9A), where its function is required (Chen *et al*, 2007). We thus also depleted different gap junction components (innexins) and other stomatin-like proteins to analyse the aggregation patterns of worms expressing muscular 40Q. Besides *unc-1*, there are another nine genes that encode stomatin-like protein in *C. elegans* (Hobert, 2013). As *unc-1* functions in neurons, we tested whether other neuronally expressed stomatin-like proteins modulate polyQ aggregation in muscle cells. Four of the genes encoding stomatin-like proteins are expressed in neurons, *mec-2*, *unc-24*, *sto-1* and *sto-6* (Carroll *et al*, 2006; Taylor *et al*, 2021) Analysis of two of these genes, *mec-2* (expressed in mechanosensory neurons) and *unc-24* (wide neuronal expression), showed that only *unc-24* mutants have a phenotype comparable to *unc-1* mutants, suggesting that not all neuronal stomatin-like proteins are involved in the regulation of polyQ aggregation (Appendix Table S3). Since *unc-1* modulates electrical synapses (Chen *et al*, 2007), we tested whether innexins (gap junction components) are polyQ aggregation modulators. To test this hypothesis, we introduced mutant alleles of some innexins that are widely expressed in the nervous system (*unc-9*, *unc-7* and *inx-7*), and others that are expressed in a restricted set of neurons (*inx-2* and *inx-6*), into a polyQ background (Altun *et al*, 2009) (Appendix Table S3). However, we observed a selective effect where only the ablation of *unc-7*, *unc-9* and *inx-2* enhances polyQ aggregation similar to *unc-1* mutants (Appendix Table S3).

To confirm the role of neuronal innexins, we performed cell-specific RNAi against some of the innexin genes, using the pan-neuronal *rab-3* promoter. Silencing of *inx-2* or *unc-7* increased inclusion body formation, in contrast with *inx-6(RNAi)* that did not modify the aggregation pattern (Appendix Fig S3A). Our data indicate that disruption of some components of neuronal gap junctions alters polyQ aggregation in muscle cells. We then investigated whether *unc-1* interacts genetically with *inx-2* or *unc-7*. The double mutant 40Q; *unc-1(vlt10)*; *unc-7(e5)* produced similar levels of aggregation to the *unc-1* worms (Appendix Fig S3B). This suggests that the two genes function in the same process. In contrast, 40Q; *unc-1(vlt10)*; *inx-2(vlt22)* animals showed an additive effect, suggesting that *unc-1* and *inx-2* operate in parallel pathways to modulate protein homeostasis (Appendix Fig S3C). Altogether, these results suggest that neuronal electrical synapses modulate protein homeostasis.

These experiments showed that altering some of these components indeed increased polyQ aggregation in muscle. Our data are consistent with a model in which loss of *unc-1* function perturbs gap junctions between IL2 and ASJ, resulting in altered signalling from ASJ. We wondered, therefore, whether the *unc-1* mutation would increase the general excitability of the ASJs, since, by connecting cells, gap junction opening can act to shunt current, with the connecting cell acting as a sink. If so, we reasoned that increased ASJ excitability and signalling could result from disrupted gap junctions between the IL2 and ASJ that would prevent this shunting.

To further investigate this, we used the fact that the ASJ neurons are sensitive to temperature, with previous studies showing that calcium concentrations in these neurons increased upon warming and decreased upon cooling (Ohta *et al*, 2014; Ujisawa *et al*, 2016). Since an increase in ASJ general excitability might be expected to increase overall cellular activity and the response to a temperature stimulus, we examined whether these temperature responses were intact in *unc-1(vlt-10)* animals by expressing the genetically encoded ratiometric calcium indicator Cameleon in ASJ. We did not see any difference in the fluorescence ratio, either at the starting temperature of 23°C or after shifting to 17°C (Appendix Fig S4A, B and D), compared to wild type animals, so tonic, global calcium concentrations do not appear to be increased. When we shifted the temperature back to 23°C, we observed a defect in the up-step in calcium concentration (Appendix Fig S4A, C and E), which was, if anything, more suggestive of a decrease in excitability. Thus, the effect of *unc-1(vlt10)* on signalling from ASJ does not stem from a general increase in ASJ excitability, so cannot simply be explained by a disruption in the gap junctions eliminating current shunting to IL2.

 © 2023 The Authors

Ablation of the sulfotransferase gene *ssu-1* rescues *unc-1*-associated protein homeostasis

It has been shown that the uncoordinated locomotion of *unc-1*-defective worms could be partially rescued in mutant alleles of *ssu-1*, a gene that encodes the only known alcohol cytosolic sulfotransferase in the worm (Carroll *et al*, 2006). Alcohol sulfotransferases are promiscuous enzymes that are able to add a sulphate moiety to a variety of molecules such as peptides and steroid hormones, as well as xenobiotics targeted for elimination (reviewed by Gamage *et al*, 2006). *ssu-1* expression is restricted to the ASJ pair of sensory neurons, which function in food sensing, hormone release, Dauer formation and temperature sensing (Schackwitz *et al*, 1996; Carroll *et al*, 2006; Hattori *et al*, 2006; Ohta *et al*, 2014). As sulphates are known to be involved in hormone processing, with sulfotransferases adding a sulphate moiety and sulfatases removing it, we wanted to determine what, if any, involvement the *ssu-1* sulfotransferase might have in proteostasis.

To investigate the potential role of *ssu-1* in protein homeostasis as an interactor of *unc-1*, we introduced the transgene expressing 40Q into worms carrying the loss-of-function alleles *ssu-1(fc73)* and *unc-1 (e580)*. *ssu-1* single-mutant animals did not exhibit a modified aggregation phenotype (Fig 3A). In contrast, mutations in *ssu-1* suppressed the effects caused by mutations in *unc-1* on protein aggregation in muscle cells (Fig 3A, Appendix Fig S1C and D). This is consistent with previous evidence that *ssu-1* mutations modified several other *unc-1* phenotypes (Carroll *et al*, 2006). To confirm these results, we induced cell-specific RNAi against *ssu-1* in the ASJ neurons of *unc-1(vlt10)* mutants using the *trx-1* promoter. As expected, reducing the expression of *ssu-1* in ASJ partially rescued the aggregation phenotype of *unc-1(vlt10)* (Fig 3B). These data indicate that SSU-1 activity in the ASJ sensory neurons is required for the disrupted protein homeostasis in muscle cells exhibited by *unc-1* mutants.

Arylsulfatase activity modulates the aggregation dynamics of polyQs

Our results suggest that SSU-1 catalyses the production of a sulfonated signalling molecule in *unc-1* mutants and that this sulfonated molecule promotes the aggregation of polyQs in muscle cells. Sulfonated molecules are more water-soluble, and thus may more easily diffuse into organs and tissues than non-sulfonated signals. However, to bind nuclear receptors, the sulfonated molecules need to lose their sulfonate moiety. Hence, we hypothesised that removing sulfonate from the signalling molecule may alter the aggregation phenotype. To test this, we disrupted the sulfatase enzymatic activity of the only three genes encoding enzymes with predicted sulfatase activity in *C. elegans*: *sul-1*, *sul-2* and *sul-3*. *sul-1* encodes a predicted protein similar to mammalian 6-O-endosulfatases (www.wormbase.org; and Appendix Fig S5), and the predicted enzymes from *sul-2* and *sul-3* are closely related to arylsulfatases (www.wormbase.org; and Appendix Fig S5). We fed animals with bacteria expressing dsRNA targeting each of these three genes, and a reduced function of either *sul-2* and *sul-3* decreased the aggregation observed in *unc-1* animals (Fig 3C). In contrast, *sul-1(RNAi)* animals did not have reduced inclusion bodies (Fig 3C), which suggests that only arylsulfatase function is able to modulate the aggregation of polyQs in *unc-1* mutants. We then analysed the aggregation phenotype of the only arylsulfatase mutant available, *sul-2(gk187)*, and observed that ablation of *sul-2* partially reduces the aggregation phenotype of *unc-1* animals (Fig 3D, Appendix Fig S1C and D). This agrees with our RNAi findings suggesting that arylsulfatase activity is required to modulate protein homeostasis.

Activation of the nuclear receptor DAF-12 reduces polyQ aggregation

Motivated by the fact that sulfotransferases such as SSU-1 add a sulphate moiety to steroid hormones, and there is evidence that SUL-2 can remove sulphate from steroid hormones (Pérez-Jiménez *et al*, 2021), we next investigated whether the signalling pathway of the dafachronic acids, targeting the DAF-12 nuclear receptor, is activated in ASJ upon *unc-1* disruption to modulate protein homeostasis. To investigate whether DAF-12 modulates the phenotype of 40Q; *unc-1* worms, we used CRISPR to generate a mutation in *daf-12*, *vlt19* that affects the ligand-binding domain (Appendix Fig S9B). While 40Q; *daf-12* animals showed a substantial increase in the number of polyQ aggregates (Fig 4A), homozygous 40Q; *unc-1*; *daf-12* mutants were synthetically lethal and could not be analysed.

To further confirm these data, we investigated the role of dafachronic acids by disrupting two enzymes involved in their synthesis, DAF-36 and DAF-9 (Matyash *et al*, 2004). However, when carrying the loss of function of *daf-36* allele *k114*, together with the insertion expressing 40Q, the worms were sterile. To avoid this issue, we rescued *daf-36* by reintroducing a DNA construct that contained the coding and regulatory region of *daf-36* into 40Q; *daf-36(k114)* worms as an extrachromosomal array. We then analysed the *daf-36* homozygous worms that lost the array, and these animals showed a substantially higher number of inclusion bodies than their siblings carrying the array (Fig 4B). To investigate the role of DAF-9 in the aggregation of polyQs, we used different concentrations of a drug that specifically inhibits this enzyme, dafadine A (1, 5, 12.5 μM). L4 40Q animals treated with this drug showed similar polyQ-related phenotypes as *daf-12* mutants (Fig 4C), whereas the 40Q worms cultured on 1 μM Δ^4-dafachronic acid had a reduced amount of polyQ aggregates (Fig 4D). Collectively, these data suggest that signalling through DAF-12 is required to maintain protein homeostasis.

Activation of the nuclear receptor NHR-1 enhances protein aggregation

It is unlikely that DAF-12 is the target of the steroid signal from ASJ because disrupting the pathway enhances aggregation and may cause a synergy with the pathway disrupted by the loss of *unc-1* function. Hence, we looked at NHR-1, a nuclear receptor that regulates insulin sensitivity in *C. elegans* and that is controlled by SSU-1 (Burton *et al*, 2018). To introduce mutant alleles of *nhr-1* on 40Q; *unc-1(vlt10)* worms, we used CRISPR to generate the mutation *vlt16*, which emulates *nhr-1(n6242)*. Additionally, we isolated and characterised a new allele of *nhr-1*, *vlt15*, which was produced by an abnormal homologous recombination and which is still likely a null allele because it causes a frameshift in the gene to produce a truncated protein (Appendix Fig S9C).

An analysis of both mutant alleles showed that disrupting *nhr-1* in *unc-1* worms substantially reduced the inclusion bodies observed in

© 2023 The Authors

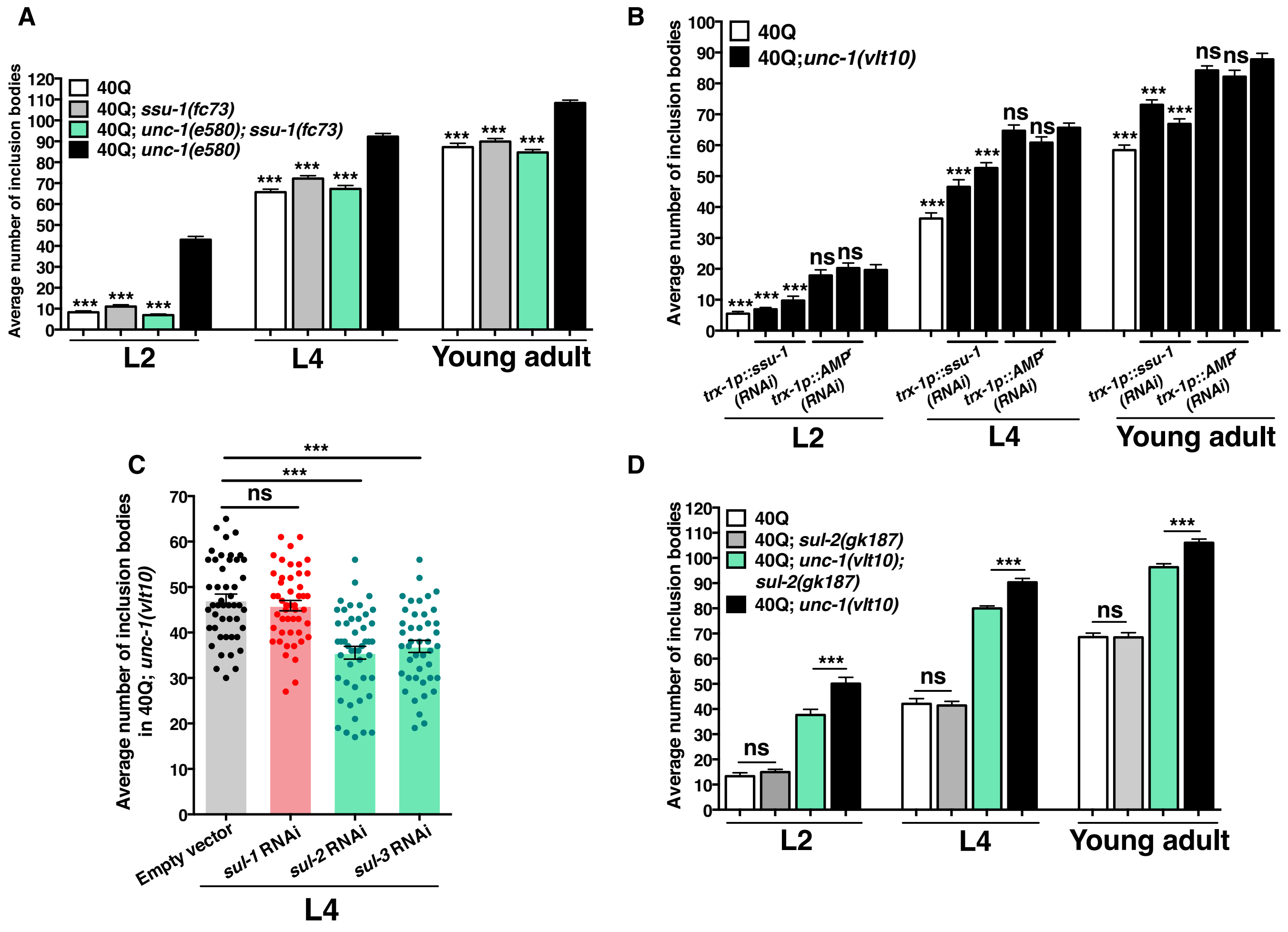

Figure 3. Neurohormonal signalling disruption produces an excess of signal that enhances polyQ aggregation.

A The average number of polyQ inclusion bodies in muscle cells after *ssu-1* and *unc-1* suppression in 40Q::YFP animals at different stages (L2, L4 and young adults).

B The average number of polyQ inclusion bodies in muscle cells after RNAi was used against ssu-1 in ASJ neurons in *unc-1(vlt10)* worms in two independent transgenic lines.

C The average number of polyQ inclusion bodies in muscle cells after ubiquitous silencing of the different sulfatases of *C. elegans* (*sul-1*, *sul-2* and *sul-3*) in the *unc-1 (vlt10)* background.

D The average number of polyQ inclusion bodies in muscle cells after disruption of *sul-2* by the loss-of-function allele, *gk187*, in the *unc-1(vlt10)* background at different stages (L2, L4 and young adult).

Data information: The plotted data show the mean ± SEM. For graphs (A, B and D), 30 animals were analysed per condition and/or strain and per experiment. For graph (C), 40 animals were analysed per condition and/or strain and per experiment. Each analysis has been reproduced at least three times. ***$P < 0.001$; ns: not significant, where values are in reference to the 40Q; *unc-1(vlt10)* strain (graph A and B) using the one-way ANOVA with *post-hoc* Tukey test for all cases.
Source data are available online for this figure.

unc-1 mutants (Fig 5A), suggesting that NHR-1 receives the signal generated by SSU-1 to regulate protein homeostasis. In addition, *nhr-1* ablation also partially restored the uncoordinated phenotype of *unc-1* worms (Fig EV1) in the same way as *ssu-1* ablation (Carroll *et al*, 2006). Carroll *et al* (2006) showed that disruption of *ssu-1* rescued the uncoordinated phenotype of *unc-1* mutants. In this regard, disruption of NHR-1 induced a similar rescue of motility in 40Q; *unc-1*; *nhr-1* mutants (Fig EV1A). Reintroduction of wild type *nhr-1* in muscle cells, in the 40Q; *unc-1*; *nhr-1* worms, substantially reduced motility, to levels similar to the *unc-1* mutants (Fig EV1A). Rescuing the polyQ-induced motor defect was also associated with a reduction in polyQ aggregation (Figs 5B and EV1A). To further confirm this correlation between motor restoration and reduced polyQ aggregation, we treated 40Q and 40Q; *unc-1(vlt10)* animals with 2 mM metformin, an anti-diabetic drug that activates AMPK to reduce polyQ aggregation (Sanchis *et al*, 2019; Gómez-Escribano *et al*, 2020). As expected, metformin treatment reduced polyQ aggregation and improved motor movement in 40Q and 40Q; *unc-1(vlt10)* animals, suggesting that the motility rescue is due to decreased polyQ aggregation (Fig EV1B and C).

To further investigate, whether NHR-1 is a modulator of proteostasis, or just a modulator of polyQ aggregation, we introduced both *vlt16* and *vlt10* alleles into an α-synuclein::YFP-expressing strain of worms. Analysis of protein aggregation, in 2-day-old adults, shows that ablation of *nhr-1* did not modify the number of α-synuclein aggregates in muscle cells (Fig EV2A and B). However, the *nhr-1*

 © 2023 The Authors

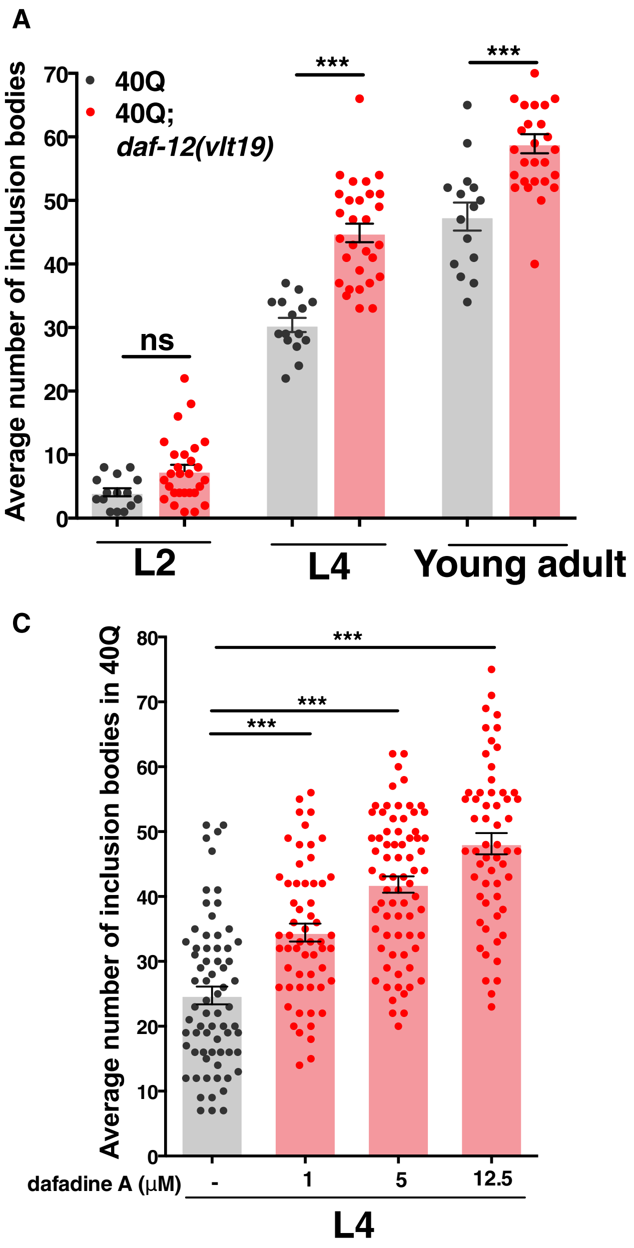

Figure 4. DAF-12 signalling is required to maintain protein homeostasis.

A The mean number of polyQ inclusion bodies in muscle cells in *daf-12(vlt19)* mutants at different stages (L2, L4 and young adult).

B The average number of polyQ inclusion bodies in muscle cells with *daf-36* suppression in 40Q::YFP compared with *daf-36* mutants rescued with an extrachromosomal array containing the whole genomic region of the gene.

C The average number of polyQ inclusion bodies in muscle cells after blocking DAF-9 via dafadine A in 40Q::YFP animals.

D The average number of polyQ inclusion bodies in muscle cells after treating 40Q::YFP young adult animals with 1 μM of Δ4-dafachronic acid.

Data information: We analysed the following number of worms per strain and/or condition: 30 animals for graph (A and B); more than 55 animals for graph (C); and more than 65 animals for graph (D). Each analysis has been reproduced at least three times. ****P* < 0.001; ns: not significant. Statistical test was done using the one-way ANOVA with multiple comparative test (Tukey's) (graphs A–C) or the Mann–Whitney *U*-test (graph D).
Source data are available online for this figure.

mutation was able to rescue protein aggregation of *unc-1* mutants (Fig EV2A and B), which suggests that *nhr-1* modulates proteostasis imbalance induced by aggregation-prone proteins.

One of the features of polyQ aggregation is that it induces some pathways of the UPR and the expression of genes encoding chaperones (Leitman *et al*, 2013; Shacham *et al*, 2019). Hence, we wanted to see whether these pathways were switched on in *unc-1* animals and whether disruption of *nhr-1* may restore them, which we did by examining the expression of key genes involved in such signalling events. If that was the case, disruption of *nhr-1* should

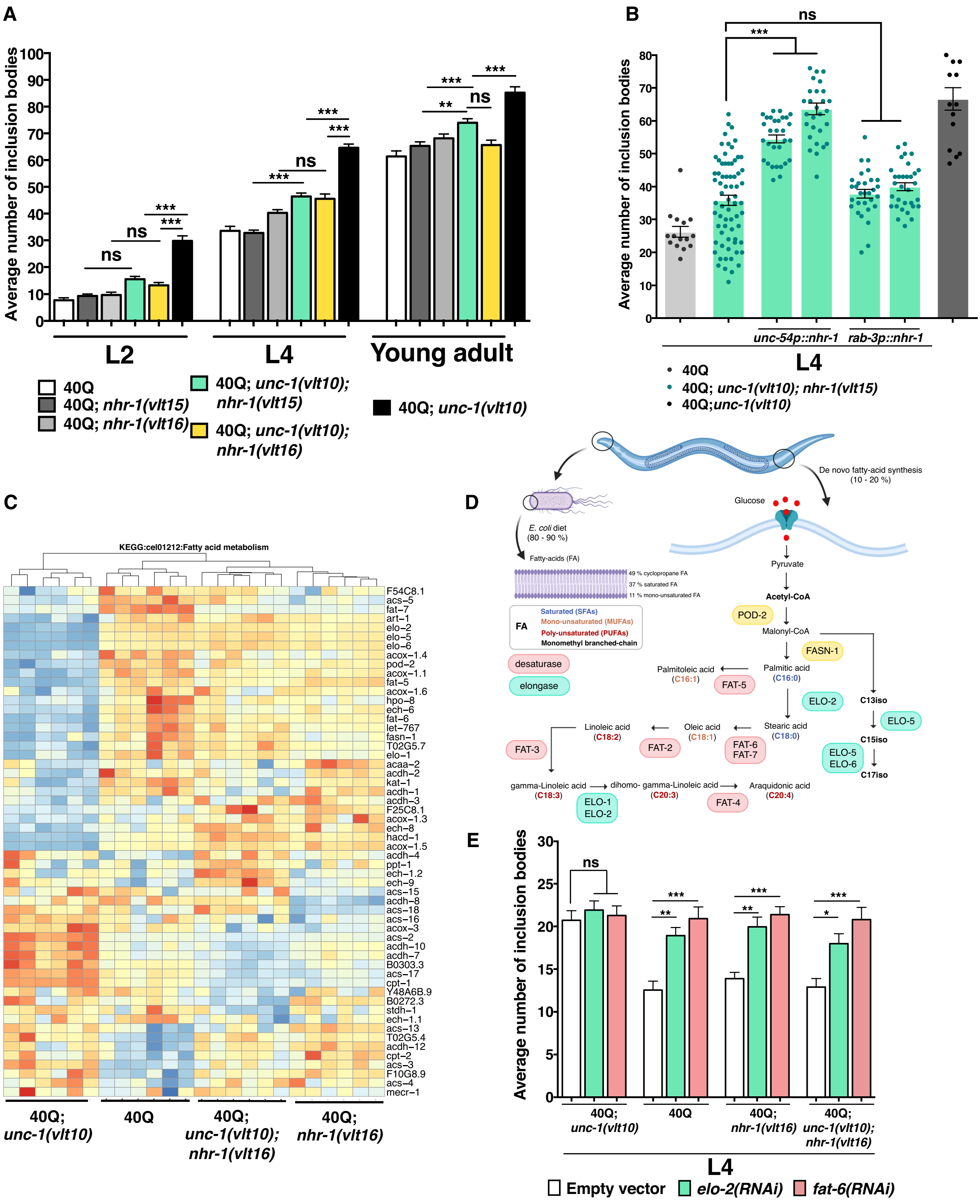

Figure 5.

© 2023 The Authors

Figure 5. NHR-1 modulates protein homeostasis by controlling genes of the fat metabolism.

A The average number of polyQ inclusion bodies in *unc-1* animals bearing *nhr-1* loss of function alleles *vlt15* and *vlt16* in 40Q::YFP animals.
B The average number of polyQ inclusion bodies after reintroducing the cDNA of *nhr-1* in muscle cells (*unc-54p::nhr-1*) and neurons (*rab-3p::nhr-1*) of the *unc-1; nhr-1* double mutant worms as compared to non-rescued animals.
C Heatmap showing the gene expression profile related to the metabolism of fatty acids (KEGG: "Fatty Acid Metabolism") of 40Q, 40Q; *unc-1(vlt10)* and 40Q; *nhr-1 (vlt16)* single mutants and 40Q; *unc-1(vlt10)*; *nhr-1(vlt16)* double mutant worms expressing polyQs. Blue indicates genes with reduced expression levels, while red indicate increased expression in each biological sample (N = 6). The highlighted genes have been selected according to a $P < 0.05$ and a fold change ≥ 2.
D Diagram of the *de novo* fatty acid synthesis pathway highlighting the role of several genes found to be downregulated (*pod-2*, *elo-2*, *elo-5*, *fat-5*, *fat-6*, *fat-7*, among others) in *unc-1* mutants.
E The average number of polyQ inclusion bodies in muscle cells in *unc-1* and *nhr-1* animals treated with RNAi against *elo-2* and *fat-6*.

Data information: Each analysis has been reproduced at least three times (more than 30 animals). $*P < 0.05$; $**P < 0.01$; $***P < 0.001$; ns: not significant, as calculated using the one-way ANOVA with *post-hoc* Tukey test.
Source data are available online for this figure.

reduce the associated stress in *unc-1(vlt10)* worms, and deactivate these pathways. Hence, we performed real-time PCR for selected genes related to the UPR of the mitochondria (UPR^{mt}), endoplasmic reticulum (UPR^{ER}) and cytosol (HSR) (Taylor *et al*, 2014), in *unc-1* and *nhr-1* mutants. As expected, *unc-1* mutants showed an increased expression of some of these genes, while double mutants *unc-1*; *nhr-1* showed reduced expression, suggesting that they have reduced stress (Fig EV1D). For example, the expression of *atfs-1*, *ire-1* and *atf-6* was upregulated in *unc-1* mutants, while their expression was normal in the *unc-1*; *nhr-1* double mutant (Fig EV1D); the same pattern occurs with the expression of the chaperone proteins *hsp-70* and *hsp-16.41* (Fig EV1D). To further demonstrate this, we sought to test the UPR^{ER} reporter *hsp-4::GFP in vivo* in wild type and *unc-1* mutants. To increase the ER-stress signal we treated *unc-1 (vlt10)* animals with mild amounts of tunicamycin (see the Material and Methods section) and compared them with wild-type worms. After treatment, *unc-1(vlt10)* animals showed higher *hsp-4::GFP* expression levels than wild type animals which further shows that *vlt10* animals have ER-stress (Fig EV1E and F). Altogether, these results show that *nhr-1* ablation restores a basal stress condition in *unc-1* mutants.

nhr-1 ablation rescues enhanced polyQ aggregation of *daf-12* mutants

We have shown that *nhr-1* and *daf-12* modulate polyQ aggregation in an opposing manner, and we wanted to next study whether they interact genetically. To do this, we first used CRISPR to produce a double mutant *nhr-1*; *daf-12* on a 40Q background because all three *loci* map very close to each other. The double mutants showed the same amount of polyQ aggregation as 40Q animals, indicating that *nhr-1* is epistatic over *daf-12* (Fig EV3A). We then introduced the *unc-1* mutation in these animals. As above, *daf-12* and *unc-1* are synthetically lethal, but the loss-of-function allele of *nhr-1* allowed us to isolate triple mutants *unc-1*; *daf-12*; *nhr-1* expressing polyQs (Fig EV3A). Because NHR-1 seems to inhibit hormone signalling through DAF-12, we studied the effect of ablating *nhr-1* in the expression of some of the genes related to the synthesis of dafachronic acids (*daf-36* and *daf-9*). To do this, we used the expression data from our transcriptomic analysis of the 40Q, 40Q; *unc-1*, 40Q; *nhr-1* and 40Q; *unc-1*; *nhr-1* worms (see below). This analysis showed that *daf-36* is clearly downregulated in 40Q; *unc-1* worms compared to 40Q animals, while *daf-9* tended to also be downregulated but less clearly (Appendix Fig S6A and B). Regardless, the worms carrying a lesion in *nhr-1* had increased expression of the two genes (Appendix Fig S6A and B). These data suggest that NHR-1 regulates the synthesis of dafachronic acids, and thus the signalling of DAF-12 (Appendix Fig S6C), and that both NHR-1 and DAF-12 produce opposing signals to modulate protein homeostasis (Fig EV3B).

NHR-1 regulates fat metabolism genes to modulate protein homeostasis

To elucidate the mechanism behind the effect of NHR-1 function, we performed transcriptomic analysis on the following strains: 40Q, 40Q; *unc-1(vlt10)*, 40Q; *nhr-1(vlt16)* and 40Q; *unc-1(vlt10)*; *nhr-1(vlt16)*. Here, *unc-1(vlt10)* mutants exhibited a distinct transcriptomic signature compared to 40Q, and introducing the *nhr-1* mutation in *40Q*; *unc-1* mutants restored the transcriptomic signature to the 40Q background (Appendix Fig S7). Among the many genes whose expression was altered in *unc-1(vlt10)* mutants, we have identified 532 genes whose expression is rescued by the *nhr-1(vlt16)* mutation (Appendix Fig S7). To identify cellular processes that were altered in *unc-1* mutants, we used the WormBase tool for Gene ontology (GO) analysis (Angeles-Albores *et al*, 2016, 2018) and KEGG pathway analysis (Kanehisa, 2000). We found that genes that were differentially expressed in *unc-1* mutants were significantly enriched in terms related to lipid metabolism, including fatty acid metabolism (KEGG:cel01212), biosynthesis (KEGG:cel00061), degradation (KEGG:cel00071) and fat content increase (WBPhenotype:0001184), UPR response and immune response, among others.

Among the altered genes, we identified expression differences in genes encoding key enzymes of lipid metabolism (Fig 5C and D). For example, we noticed that acetyl-CoA carboxylase (ACC) encoded by *pod-2* and fatty acid synthase *FAS* encoded by *fasn-1* show lower expression levels in *unc-1* mutants compared with double mutants (Fig 5C). Both enzymes are involved in the first steps of *de novo* fatty acid synthesis. Known for catalysing carbon chain extensions of fatty acids, elongases (*elo-1*, *elo-2*, *elo-5* and *elo-6*) also had altered expression in *unc-1* mutants compared to the 40Q worms (Fig 5C). Other types of enzymes were also downregulated in *unc-1* mutants, like desaturases (*fat-5*, *fat-6* and *fat-7*), which are involved in removing hydrogen atoms from carbon to produce double bonds (Fig 5C). In clear contrast, *nhr-1* ablation

© 2023 The Authors

reversed the expression patterns of these genes in *unc-1* mutants, and their expression was closer of that of 40Q worms (Fig 5C).

To validate the transcriptomic results, we analysed the role of the elongase *elo-2* and the desaturase *fat-6* in terms of polyQ aggregation, using RNAi by feeding to reduce their function in 40Q and mutant backgrounds. Both genes are essential for producing oleic acid, a lipid that is neuroprotective in rodents (Song *et al*, 2019), induces lifespan extension (Fang *et al*, 2016) and prevents polyQ aggregation in worms (Goudeau *et al*, 2011; Han *et al*, 2017; Imanikia *et al*, 2019). Silencing *fat-6* and *elo-2* expression increased the number of inclusion bodies in all strains except 40Q; *unc-1 (vlt10)* animals (Fig 5E), where there are already high levels of inclusion bodies. These functional data further suggest that NHR-1 controls the expression of some enzymes of the fat metabolism, which in turn are essential for maintaining protein homeostasis.

Finally, we wanted to confirm whether *unc-1* perturbs *ssu-1* signalling, which impacts polyQ aggregation. For this, we took advantage of our transcriptomic data, comparing it to genes shown by Burton *et al* (2018) to be regulated by *ssu-1*. The expression analysis showed that almost all genes have an opposite expression profile in *unc-1* mutants (Fig EV4A and B), suggesting that *unc-1* affects *ssu-1* signalling and that both are connected.

***unc-1* animals show altered fat metabolism and ablation of *nhr-1* restores their lipid profile**

To further investigate the altered lipid metabolism associated with *unc-1* mutants, we analysed their lipid content using Oil Red O. This die stains neutral lipids (triglycerides, mono- and di-glycerides, cholesterol esters, etc.) to provide a semi-quantitative measure of total neutral lipids. We observed a significant enhanced staining in 40Q; *unc-1* mutants compared to 40Q worms (Fig 6A and B). Conversely, *nhr-1* suppression completely restored the lipid content (Fig 6A and B).

Delving deeper into this apparent lipid dysregulation of *unc-1* mutants, we performed an untargeted lipidomic assay to determine the specific fatty acid content associated with the *vlt10* allele. Though we observed more staining of neutral lipids in *unc-1* mutants in this assay, we still observed a reduction in triglycerides (Fig EV5A). To learn which fatty acids may be remodelled, we investigated the absolute total levels of these compounds in our strains (Fig 6C–I). Stearic acid, the precursor of oleic acid, was significantly lower in mutants than in wild type worms (Fig 6F), while the levels of manolyl-CoA increased (Fig 6C). Moreover, the levels decreased for palmitic acid, palmitoleic acid, oleic acid, gamma-linoleic acid and dihomo-gamma-linoleic acid (Fig 6D, E and G–I). These results thus suggest that *vlt10* can induce the remodelling of lipid abundancy by decreasing fatty acids and, in turn, triglycerides, but may increase the amount of neutral lipids.

Among other fatty acids, *elo-2* and *fat-6* are involved in the *de novo* synthesis of oleic acid (Ntambi *et al*, 2002; Brock *et al*, 2007) and are protective of neurons stressed by polyQ expression (Imanikia *et al*, 2019). Hence, we tested whether this fatty acid can modulate polyQ aggregation. To do so, we treated 40Q worms with 2 mM of oleic acid and analysed the polyQ inclusion bodies. As expected, oleic acid reduced polyQ aggregation in 40Q worms (Fig 6J), but this treatment was more effective in *unc-1* mutants compared with 40Q, which had lower expression levels of oleic acid synthesis enzymes (Fig 6J). Oleic acid treatment was ineffective in *nhr-1(vlt16)* worms (Fig 6J), which suggests that *nhr-1* is required for the beneficial effect of oleic acid.

Discussion

To uncover hidden mechanisms of protein homeostasis, we isolated a gene from a forward genetic screen, *unc-1*, that alters protein aggregation and motor coordination in *C. elegans*. We show that UNC-1 functions non-cell autonomously, in neurons, and disruption of the gene led to secretion of an excess of a sulphated signal that activates NHR-1 in different tissues. Over-activation of NHR-1 results in downregulation of genes encoding enzymes for fat metabolism, which in turns disrupts proteostasis. In contrast, signalling through DAF-12 functions antagonistically to NHR-1 to control protein aggregation.

The identified gene, *unc-1*, encodes a *C. elegans* stomatin-like protein that regulates protein homeostasis in muscle cells and neurons, acting in IL2 neurons (Figs 1 and 2). Our data also shows that the effect on aggregation of polyQs is specific to certain stomatin-like proteins (Appendix Table S2). Aggregation can similarly be enhanced by the loss of function of several innexin genes, which suggests the involvement of gap junctions—the pore components of the electrical synapse—at least in invertebrates (see review Dahl & Muller, 2014). This suggests that electrical synapses are essential for maintaining appropriate proteostasis in *C. elegans*. If the ASJ-IL2 gap junctions served to simply shunt current, as we had expected, the loss of function of *unc-1* would result in a general increase in ASJ excitability (Appendix Fig S4), though this was not the case. Rather, the differential effect on different ASJ functions suggests that these innexins play more complex roles, which might be specific to different aspects of ASJ function. However, innexins can also operate as hemichannels (Bouhours *et al*, 2011; Hervé & Derangeon, 2013), which may explain this specificity. We believe that these data open many questions, regarding the influence of the electrical synapses, or their constituent subunits, over the control of metabolism, that are worth pursuing in the future. What is sensed by ASJ and/or IL2, and why these upstream signals regulate, is unknown to us, and it will be very interesting to study them in the future.

Our results also suggest that disrupting the communication between some neurons triggers the secretion of excess sulphated hormone, from ASJ, in a process dependent on SSU-1 (Fig 3A). An important remaining question is the nature of the sulphated signal released by ASJ. We have used multiple metabolomic approaches to try to identify this putative hormone, but this may require technological advances currently outside our reach. Whatever the nature of the signalling coming out of the neuron, our data shows that altering the function of *unc-1* induces changes in genes regulated by *ssu-1*, which shows that the flow of information from *unc-1* downstream, involves *ssu-1* (Fig EV4A and B).

For this hormonal signal to induce an enhanced polyQ aggregation on muscle cells and neurons, it requires functioning arylsulfatases SUL-2 and SUL-3 (Fig 3C). These proteins are arylsulfatases, evolutionarily closer to human steroid sulfatases, which suggests that the sulphur groups of this unknown hormone need to be removed for receptor binding.

 © 2023 The Authors

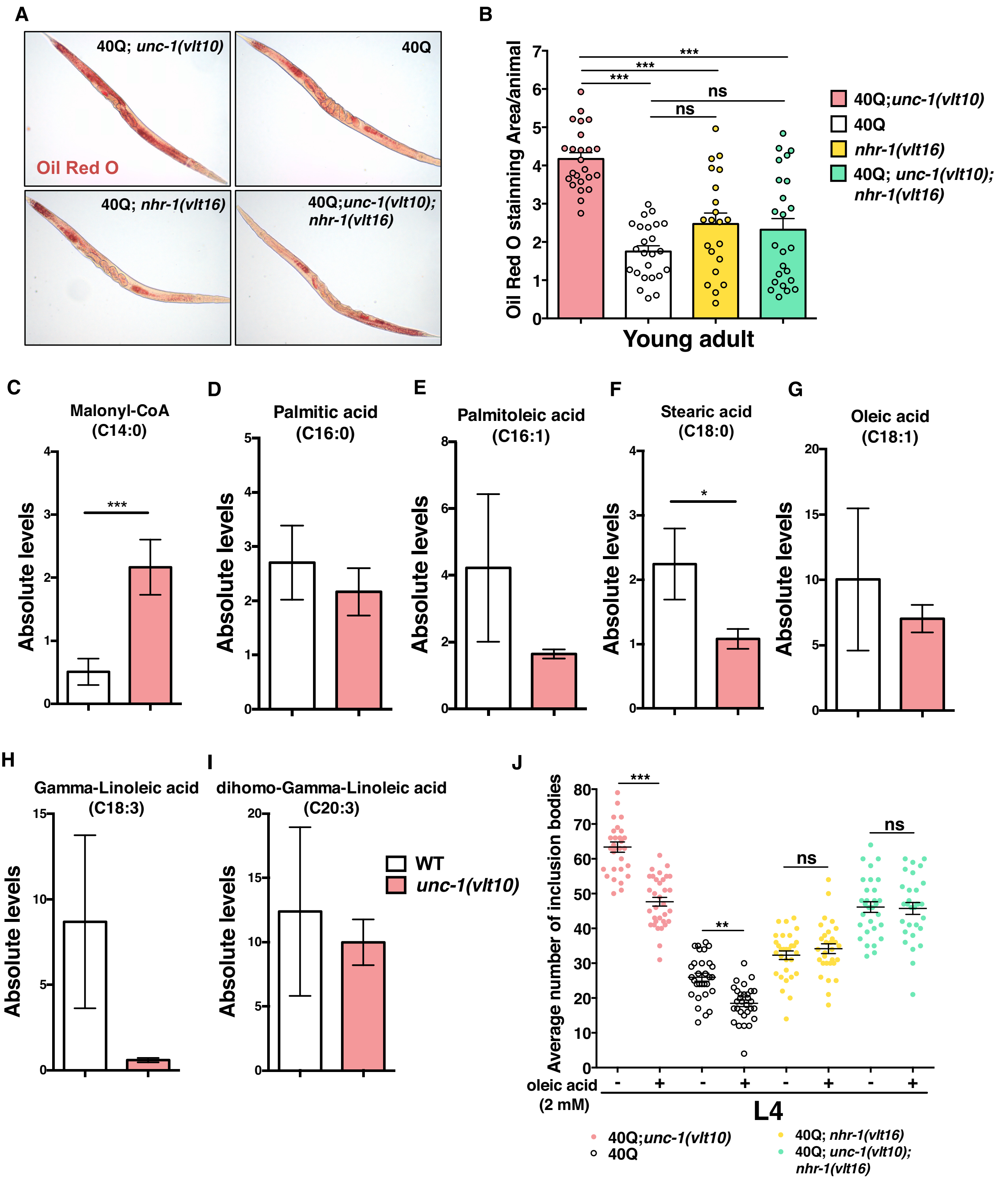
A
40Q; unc-1(vlt10)
40Q
Oil Red O
40Q; nhr-1(vlt16)
40Q;unc-1(vlt10); nhr-1(vlt16)
B
Oil Red O stainning Area/animal
***
***
***
ns
ns
Young adult
40Q;unc-1(vlt10)
40Q
nhr-1(vlt16)
40Q; unc-1(vlt10); nhr-1(vlt16)
C
Malonyl-CoA (C14:0)
***
Absolute levels
D
Palmitic acid (C16:0)
Absolute levels
E
Palmitoleic acid (C16:1)
Absolute levels
F
Stearic acid (C18:0)
*
Absolute levels
G
Oleic acid (C18:1)
Absolute levels
H
Gamma-Linoleic acid (C18:3)
Absolute levels
I
dihomo-Gamma-Linoleic acid (C20:3)
Absolute levels
WT
unc-1(vlt10)
J
Average number of inclusion bodies
***
**
ns
ns
oleic acid (2 mM)
- + - + - + - +
L4
40Q;unc-1(vlt10)
40Q
40Q; nhr-1(vlt16)
40Q; unc-1(vlt10); nhr-1(vlt16)

Figure 6.

Figure 6. Lipid metabolism modifies polyQ aggregation.

A Representative images of simple and double young adult mutants of unc-1 and nhr-1 stained with Oil Red O to examine neutral lipid accumulation.
B Semi-quantitative analysis of the OilRed O staining in (A) using a plugging from ImageJ.
C–I Absolute lipid levels of saturated (manoloyl-CoA, palmitic acid and stearic acid), monounsaturated (oleic acid) and polyunsaturated (gamma-linoleic and dihomo-gamma linoleic acids) fatty acids in wild type and *unc-1(υlt10)*mutants.
J The average number of polyQ inclusion bodies in muscle cells after treatment with 2 mM of oleic acid in 40Q, 40Q;*unc-1(υlt10)*, 40Q; *nhr-1(υlt16)* and double mutant of both genes.

Data information: The plotted data show the mean ± standard error of the mean (SEM). Each analysis has been reproduced at least three times (more than thirty animals) in A, B and J. At least twelve worm biological samples containing more than 2000 animals per sample were analysed in C-I. *$P < 0.05$; **$P < 0.01$; ***$P < 0.001$; ns: not significant. The statistical analysis was done using a Mann–Whitney *U*-test for C–I and one-way ANOVA with *post-hoc* Tukey test for B and J.
Source data are available online for this figure.

From our epistasis analysis, we saw that the steroid hormone produced by ASJ seems to be controlled by NHR-1 and that this nuclear receptor regulates genes related to lipid metabolism, which in turn affects proteostasis in *C. elegans*. Our transcriptomic analysis of single and double *unc-1* and *nhr-1* mutants indicated that many genes involved in fatty acid synthesis are altered in *unc-1* worms and that introducing *nhr-1* loss-of-function alleles restores their expression to close to that of 40Q worms (Fig 5C and E). We validated this role of fat metabolism by reducing the function of *elo-2* and *fat-6*, whose silencing increases polyQ aggregation (Fig 5E). Because FAT-6, together with FAT-7, converts stearic acid into oleic acid, a protective lipid that promotes longevity and improve neuronal proteostasis (Goudeau *et al*, 2011; Imanikia *et al*, 2019), we examined oleic acid synthesis in these worms. We showed that the enzymes involved in oleic acid synthesis are downregulated in *unc-1* mutants and that oleic acid is effective in reducing polyQ aggregation, suggesting reduced oleic acid synthesis in these animals. However, this lipid had no effect over *nhr-1* mutants (Fig 6J), suggesting that oleic acid may depend on functional NHR-1 to produce the beneficial effects on proteostasis. While oleic acid production is controlled by NHR-1, this result is still not surprising given that some nuclear receptors are controlled by feedback from the products of the enzymes they regulate (Bagamasbad & Denver, 2011). In support of both this and the transcriptomics analysis, an untargeted lipidomic assay showed that the *unc-1* mutants tended to produce lower amounts of certain free-fatty acids. Altogether, these results suggest that an altered neurohormonal signalling dysregulates fat metabolism, which in turns affects protein homeostasis.

Our data also strongly suggest that different steroid hormones and different signalling pathways exert opposing effects on protein homeostasis. In contrast with NHR-1 disruptions, disrupting the nuclear receptor DAF-12 (i.e., mutating *daf-12* or culturing worms on dafadine A, a well-known *daf-9* inhibitor) enhanced polyQ aggregation; adding dafachronic acids produced a protective effect (Fig 4) (Martin *et al*, 2008, 2009). In agreement with our findings, Farina *et al* (2017) showed that the neurosteroids that activate DAF-12 are neuroprotective in *C. elegans* models of polyQ-induced toxicity. Moreover, other authors showed that *daf-12* signalling promotes protein homeostasis by modulating the function of the ER stress response (Mark *et al*, 2016). In addition, *nhr-1* is epistatic over *daf-12*, suggesting that the two receptors compete to modulate protein homeostasis (Fig EV3, Appendix Fig S6).

To our knowledge, this is the first report showing that different steroid hormone signals have opposing modulator effects on protein homeostasis in animals, and it will be of interest to investigate if this is evolutionarily conserved between invertebrates and mammals. There is also evidence that neurosteroids may protect against neurodegenerative diseases that involve the dysregulation of protein homeostasis, such as for Alzheimer's, Parkinson's or HD (see review: Borowicz *et al*, 2011). These points are especially important for the development of drugs against inflammatory and metabolic and neurodegenerative diseases, as the wide range of metabolic processes regulated by nuclear receptors makes them outstanding druggable targets (see reviews: Schulman & Heyman, 2004; Saijo *et al*, 2010; Schulman, 2010; Skerrett *et al*, 2014; Moutinho *et al*, 2019). Inhibitors for many of the proteins in these signalling pathways have also been developed to treat different kinds of cancer and metabolic diseases, such as against sulfotransferases (Chapman *et al*, 2002; Rath *et al*, 2004), the cytochrome *daf-9*/CYP2J2 (Chen *et al*, 2009), sulfatases (Mostafa & Taylor, 2013; Yue *et al*, 2016; Pérez-Jiménez *et al*, 2021) and modulators of NHRs (Chen, 2008; Pinne & Raucy, 2014; Davis *et al*, 2018; Hong *et al*, 2018; Kang *et al*, 2018). Additionally, some attempts have been made to use fatty acid-enriched diets to treat neurodegeneration, although the results were not very promising (see reviews: Shamim *et al*, 2018; Bono-Yagüe *et al*, 2020). We expect that new approaches will focus on applying modulators of key enzymes of the lipid metabolism to modify the amount of protective lipids within the stressed tissue.

Overall, this work has provided the first evidence that nuclear receptors modulate proteostasis through the remodelling of lipid metabolism. Our findings transfer the attention to lipids to find potential pharmacological targets for treating pathological conditions produced by toxic mutant proteins that induce neurodegeneration.

Materials and Methods

Maintenance and culture of *C. elegans*

All strains were maintained under standard conditions (Brenner, 1974). Some strains used in this work were obtained from Caenorhabditis Genetics Center (CGC; Minneapolis, MN, USA). The CGC is funded by the National Institutes of Health's National Center for Research Resources (NGRRs). CW911 strain: *ssu-1(fc73) V*; *unc-1(e580) X* was kindly provided by Phillip G. Morgan (University of Washington, Seattle, USA). AX5828: dbEx804[*trx-1p::YC3.60*, *unc-122p::GFP*] was kindly provided by Mario de Bono. Appendix Table S4 provides a detailed list of strains used in this

 © 2023 The Authors

study. All strains were outcrossed at least three times and cultured at 20°C.

Genetic manipulation of *C. elegans*

To generate vectors for gene expression in *C. elegans*, we took advantage of Gateway® MultiSite Pro system (Invitrogen, Waltham, MA, USA). Primer sequences for cloning are listed at Appendix Table S5. Briefly, we amplified 1.7 kb from promoter region upstream of *rab-3* gene using specific primers wild type gDNA. We cloned the attB1-B2-flanked PCR product into pDONR™ 221-P1P5r to generate pDONR 221-P1P5r-*rab-3p* (pAPG1). We amplified the cDNA of *unc-1* to clone it into pDONR 221-P5P2-*unc-1* (pAPG2). We used pDONR 221-P1P5r-*myo-3p* kindly provided by Robyn Branicky. Plasmids containing each promoter region, were recombined with the cDNA of *unc-1*, within a destination vector that contains the transcription terminator from the gene *unc-54*, pHP2 (Walker *et al*, 2009), to generate the following destination constructs: pDEST HP2-*rab-3p::unc-1::unc-54t* (pAPG3) and pDEST HP2-*myo-3p::unc-1::unc-54t* (pAPG4). To induce *nhr-1* expression in muscle cells and neurons we used pVD105 and pVD106, respectively (Burton *et al*, 2018).

To produce a dominant negative form of *unc-1*, the *n494* allele, we introduced the mutation into a cDNA of *unc-1* containing in pAPG2 to obtain pDONR 221 P5P2- *unc-1(n494)* (pAPG5). Next, we used pAPG1 and pAPG5 as donor vectors and pHP2 as destination vector, to generate pAPG6 (*rab-3p::unc-1(n494)::unc-54t*). To rescue *unc-1* in IL2 neurons we obtained fusion PCR products using *osm-3p* and *oig-1p* (see more detailed Appendix Fig S8). DNA mixtures for injection consisted of 25–50 ng/μl of the interest DNA [except for *myo-3p* or *unc-54p* expression analysis (1–5 ng/μl)], a DNA marker (pCFJ90; *myo-2p*::mCherry) at 2.5–5 ng/μl, together with the empty yeast plasmid pYES (Thermofisher, Waltham, MA, USA) as carrier DNA, at a total final concentration of 120 ng/μl.

Cell-specific RNA interference

To induce tissue-specific RNAi we developed PCR fusion products using a promoter region to sense and antisense fragments of the interest gene (Appendix Fig S8). Therefore, simultaneous expression of these DNA constructs in a specific tissue, such as nervous system (which are refractory to RNAi), induce a double complementary strand RNA synthesis to activate RNAi mechanism in neurons. We amplified promoter regions (of *rab-3*, *trx-1*, *oig-1*, *osm-3*, *glr-1*, *gpa-9*) to allow us to express dsRNA in a tissue-specific manner (nervous system, ASJ, IL2, PVQ neurons, respectively) (Appendix Fig S8, Appendix Table S5). In contrast with ASJ, there are not promoters, to our knowledge, that drive the expression of genes exclusively in IL2L and/or PVQR neurons (Chelur & Chalfie, 2007). We therefore used pairs of promoters, whose expression overlapped only in our neurons of interest, to drive expression of the two RNA strands, such that expression of dsRNA was restricted to IL2 or PVQ. In parallel, we amplified sense/antisense fragment from our genes of interest (*unc-1*, *unc-7*, *inx-2*, *inx-6* and *ssu-1*), adding a sequence complementary to the specific promoter that we wanted to fuse with it (Appendix Fig S8, Appendix Table S5). Both PCR products were combined in a single PCR reaction to amplify fused PCR products, using appropriate external primers complementary to the promoter and gene (Appendix Fig S8, Appendix Table S5). We used a DNA fragment from the bacterial β-Lactamase gene (which we named AMP^r) as negative control in the tissue-specific RNAi experiments. We injected 2.5 ng/μl of each construct (sense/antisense) together with pCFJ90 (encodes a pharynx-expressed mCherry as a transgenic marker) as a reporter, and pYES (Thermofisher) as carrier DNA until 120 ng/μl.

RNA interference by feeding

To induce ubiquitous and constitutive RNAi, we feed the animals with modified HT115 *Escherichia coli* strain expressing dsRNA of each gene. We generated RNAi vectors for these genes cloning a coding fragment that covers all isoforms of each gene, into an EcoRV site of pL4440 vector. The PCR product for each gene was amplified using Phusion polymerase (Thermofisher) and specific primers (see detailed sequence in Appendix Table S5). HT115 strains containing empty pL4440, as a control, and RNAi vectors (pAPG11-*sul-1*, pAPG12-*sul-2*, pAPG13-*sul-3*, pAPG52-*elo-2* and pAPG53-*fat-6*) were grown in liquid Luria-Bertani medium containing 50 μg/ml carbenicillin over night at 37°C. Next, bacteria culture was induced by IPTG for 2 h at 37°C, before seeding RNAi plates. After drying plates, synchronised animals (L1 or L3; depending of the assay) were feed with HT115 bacteria containing empty vector or RNAi vectors until reaching L4 stage to be scored.

Construction of knock-in worms using CRISPR

We used CRISPR/Cas9 system to introduce point mutations into *nhr-1* and deletions into *inx-2* and *daf-12* sequences. We based on our design and strategy from Julián Ceron Lab (IDIBELL Institute, Barcelona, Spain) (Vicencio *et al*, 2019). To introduce deletions into *inx-2* and *daf-12* genes, we used two crRNAs for each one, which are complementary to distal exon regions. We generated a full deletion of *inx-2*, *vlt22*, using the following gRNAs: crRNA#1 5′ – ACCGGA GCTCTCCCACCAAA – 3′ and crRNA#2 5′ – GCACAATGAGAAACCA GTAT – 3′. We used the ssODN sequence to isolate mutant strains easier: 5′ – ACGTAGCGTTT GCGTGCGCACACCTCGCGTAGTGGTCC GCGTTCGCATTACTTGCGCTGG**GGAAA**CATACTGTACTGATCGATC AAGAGTTTTCACTGTCTTCTCGTCCATCACCAGCCATATTCATAATTT CTTTCAAT – 3′ (neutral nucleotides are marked in bold and it is useful to genotyping mutants) (Appendix Fig S9A). For *daf-12*, we generated *vlt19* allele using the following crRNAs: crRNA#1 5′ – ATATTATGGAT GTTACCATG – 3′ and gRNA#2 5′ – GGAATCGTTGTTCGGAGAGC – 3′. *vlt19* encodes a deletion of 500 pb inside of ligand binding domain (Appendix Fig S9B). We targeted *nhr-1* to emulate *n6242* allele by the following crRNA: 5′ – CACCACTCCACACCGTCTTC – 3′. The ssODN sequence to induce knock-in to generate the allele of interest was: 5′ – CCAACGAAGAAAATCAAGATGAGCAGCGGATCTGACGACGAGCAAG CCACCACTCCACAC**A**G**A**CT**C**T*A***A**GACCAGGTCAGTGGGCGAAACA-CATTTACCCCAATTTGGATGCATCTTGAATTTCAACAA – 3′ to generate *vlt16* in *nhr-1* (in bold are marked neutral changes and in italics are denoted the point-nonsense mutation (C/T) (Appendix Fig S9C)). We isolated a new allele, *vlt15*, by an abnormal random recombination that encodes a premature stop codon later than *vlt16* (Appendix Fig S9C). To produce the mixture for injection, the crRNAs were suspended in 20 μl of IDTE nuclease free buffer to obtain 100 μM stock. ssODNs were suspended in the same buffer at 1 μg/μl. CRISPR components were added at the following final concentrations: Cas9: 4.5 μM; ALT-R trackRNA: 32 μM; target gene crRNA: 35 μM; ssODN target gene: 175 ng/μl. For targeting deletions,

© 2023 The Authors

we used final concentrations 17.5 μM for each crRNAs. We used disruption of *dyp-10* gene as a selection marker.

Quantitative PCR

The relative expression of genes was measured by RT–qPCR using the ViiA7 thermal cycler from Applied Biosystems (Waltham, MA, USA) using Taqman™ probes. To verify the expression of genes included within extrachromosomal arrays, we selected by hand worms expressing reporter (*myo-2p::mCherry*) for RNA extraction (OMEGA Bio-Tek, Norcross, GA, USA) using the M165FC dissecting microscope (Leica, Wetzlar, Germany). To evaluate expression of genes in mutants, we collected a synchronised population of young adult worms in RNA lysis buffer. Extracts were frozen at −80°C before RNA extraction. RNA samples were treated by DNAase treatment (Qiagen, Hilden, Germany). cDNA synthesis was done using 0.1–1 μg approximately of RNA following the manufacturer protocol (Takara Bio, Kusatsu, Japan). We used the 2xPrimeTime® Gene Expression Master Mix (Takara Bio), and the PCR program was as follows: 1 cycle of 10 min at 50°C; 1 cycle of denaturalization at 95°C 15 s; 40 cycles of polymerisation at 60°C 1 min. To normalise relative expression, we used the housekeeping control, *pmp-3* (Taqman probe from Applied biosystems, Ce02485188_m1). To measure expression of interest genes or transgenes we designed customised probes from IDTDNA. We considered all isoforms for each gene in the customised design. We included at least three technical replicates for each measuring.

Western blot

We grew synchronised L1 worms until young adult stage to isolate total protein extract using RIPA buffer. The BCA kit was used to quantify total protein and after this, we added 4× SDS sample loading buffer and preserved the samples at −80°C. Total protein extracts were separated by 12% sodium dodecyl sulphated-polyacrylamide gel electrophoresis (SDS-PAGE) and transferred to polyvinylidene difluoride (PVDF) membranes (Bio-Rad Laboratories, Hercules, CA, USA) by semi-dry blotting (Trans-Blot Turbo, Bio-Rad). Membrane blocking was done with 5% non-fat milk, according to the specification of the following primary antibodies: mouse anti-polyQ (1:1,000, Sigma Ref. #P1874) and mouse anti-actin (1:500, Santa Cruz Biotechnologies ref. #sc-47778) to normalise the results. PVDF membranes were stripped after detecting actin. Primary antibody incubation was done at 4°C and next we incubated the second antibody anti-mouse conjugated to HRP at room temperature (1:10,000, Abcam, ref. #ab97023). PVDF membranes were stripped after detecting actin and next, polyQ was detected in the same membrane. Images were obtained using NZY Advanced ECL (Nzytech, ref. #MB40201). Quantification was done using the ImageJ software.

Analysis of the effect of Δ^4-dafachronic acid and dafadine A on worms

Briefly, we grown synchronised L1 animals on Dafachronic acid 1 μM (DA dissolved in ethanol) (Martin *et al*, 2008, 2009) in M9 buffer containing 5 μg/ml cholesterol, 12.5 μg/ml nystatin, 50 μg/ml streptomycin and OP50 *E. coli* at 0.5 Optic density. Dafadine A (Sigma-Aldrich-Merck, St. Louis, MO, USA) was added at three dose (1, 5, 12.5 μM) Animals were growth in liquid medium at 60°C and moderate shaking until young adults. We evaluated the effect of both compounds at least three times in independent experiments over polyQ aggregation pattern.

X-34 and Oil Red O staining

X-34 dye (Sigma-Aldrich-Merck) stains specifically amyloid deposits. One-day synchronised adults were incubated in a drop containing 1 mM X-34 and 10 mM TRIS pH 7.5 solution. Stained animals were washed with PBS-Tween and transferred to fresh NGM plates. We used the Oil Red O (Sigma-Aldrich-Merck) dye to stain triglycerides and lipoproteins for semi-quantitative purposes. Young adults were stained with diluted 3:2 Oil Red O stock solution for 2 h at room temperature in a rotator. After this, animals were washed two times with PBS-Tween (0.1%) for 30 min. Stained animals were mounted in 2% agarose pads with sodium azide (0.05 M) to count amyloid deposits using a DM2500 fluorescent microscope (Leica). To quantify the stained area of worms, with the Oild Red O stain, we used a DMD108 microscope (Leica). We analysed the pictures obtained from these animals using the Image J software.

Oleic acid treatment of worms

We generated NGM plates containing 2 mM oleic acid, which was added to molten NGM medium. Plates were conserved in the dark and seeded with OP50 before use it. Synchronised L1 animals were grown until they reached the L4 stage to score polyQ inclusion bodies.

Quantification of polyQ inclusion bodies in muscle cells and neurons

The expression of the *40Q::YFP* transgene produces body inclusion formation in muscle cells in an age-dependent manner. The average number of inclusion bodies were obtained by counting total number of polyQ::YFP inclusion bodies in muscle cells per animal *in vivo* using a dissecting microscope equipped with fluorescence (M165FC, Leica). PolyQ aggregation happens in an age-dependent manner, so we analysed aggregation in specific developmental stages (L2, L4 and young adult). Animals were analysed from a heterogeneous population and, after scoring, they were removed to avoid undesired duplicates. Scoring was from at least three independent experiments, in which we counted at least, 10 animals per genotype and stage. Thirty animals or more were analysed for each genotype and stage in total.

To score neuronal polyQ aggregates we used a model that expresses 40 glutamines under the control of the promoter of the *F25B3.3* gene. Neuronal aggregation is more complex to follow, in whole animals, so we selected the Ventral Nerve Cord only to count inclusion bodies. Sixty young adults were analysed for each genotype in total, from three independent experiments. The number of neuronal polyQ aggregates was scored using the DM2500 Leica vertical microscope, equipped with fluorescence.

Scoring of number of α-synuclein::YFP aggregates in muscle cells

The *unc-54p::α-synuclein::YFP* transgene induces late aggregation, in contrast with polyQ aggregation. Moreover, unlike 40Q::YFP inclusion bodies, which are visible using a dissecting microscope, α-

© 2023 The Authors

synuclein::YFP aggregates are only detectable using upright microscopes (like, the DM2500, Leica). Since it is not feasible to perform a complete count of whole muscle tissue, we selected the area located between the positions of the two pharyngeal bulbs, to evaluate the aggregation pattern of α-synuclein. Sixty 2-day-old adults were analysed for each genotype in total, from three independent experiments.

Scoring of number of beta-amyloid X-34-stained deposits in muscle cells

To evaluate β-amyloid protein aggregation, we used a *C. elegans* model that expresses the human Aβ peptide constitutively in muscle cells. To detect β-amyloid deposits, we stained 1-day-old adults with X-34 dye (Sigma-Aldrich-Merk). Stained 2-day-old animals were analysed using an upright microscope equipped with fluorescence (DM2500, Leica). We selected the area between the positions of the two pharyngeal bulbs to evaluate the number of β-amyloid deposits. Sixty 2-day-old adult animals were analysed for each genotype in total, from three independent experiments.

Motility thrashing assay

We evaluated motility capacity of young adults by thrashing. This assay consists of scoring the number of thrashes that show an animal when swimming in M9 buffer. We have considered a thrash when animal moves simultaneous head and tail. Animals were acclimated for 30 s before scoring number of thrashes for the following 30 s. Each animal was collected independently in a well. The average number of thrashes was extrapolated for 1 min to show the mean of the values. At least 15–20 animals were analysed for each genotype and transgenic line in three independent experiments at 20°C. Animals damaged during handling were discarded in this study.

Calcium imaging

Calcium imaging in ASJ was carried out as described previously (Kerr *et al*, 2000; Walker & Schafer, 2020). The ratiometric calcium indicator YC3.60 (Cameleon) was expressed in ASJ using the *trx-1* promoter (Fenk & de Bono, 2015). Briefly, animals were glued onto 2% agarose pads and temperature stimuli were administered using perfusion of CTX buffer (25 mM KPO_4 pH6, 1 mM $CaCl_2$, 1 mM $MgSO_4$). Images were recorded at 5 Hz using a Dualview beam splitter (Optical Insights) to simultaneously record two wavelengths and an iXon EM camera with IQ1.9 capture software (Andor Technology). Fluorescence was quantified and analysed in Matlab (Mathworks) using SpikeFinder and NeuronTracker, custom analysis scripts written by Rabinowitch *et al* (2013).

Transcriptomic analysis

Synchronised L1 (40Q, 40Q; *unc-1(vlt10)*, 40Q; *nhr-1(vlt16)* and 40Q; *unc-1(vlt10); nhr-1(vlt16)*) were grown in NGM plates at 20°C until they reached young adult stage. They were subsequently collected in M9 buffer, frozen, thawed and mechanically lysed with 200 mg of glass beads (Sigma-Aldrich-Merck) for 30 s using a Fastprep shaker apparatus (Thermofisher Scientific, FP120 model). RNA was extracted using NZY total RNA isolation kit, Nzytech. Supernatant was recovered from lysate samples by centrifuging extracts at maximum speed for 1 min. The purified RNA was treated with DNAse, quantified and sent for sequencing by Novogene (Cambridge, UK). Six biological replicates, consisting of pooled bulk nematode RNA were sequenced for each genotype. Briefly, after quality control, mRNA was enriched using oligo(dT) beads and randomly fragmented. cDNA was synthesised using random hexamers and reverse transcriptase. After first-strand synthesis, a custom second-strand synthesis buffer (Illumina) was added with dNTPs, RNase H and *E. coli* polymerase I in order to generate the second strand by nick-translation. To prepare the final cDNA library, a round of purification, terminal repair, A-tailing, ligation of sequencing adapters, size selection and PCR enrichment were performed. Library concentration was first quantified using a Qubit 2.0 fluorometer (Life Technologies). Insert size was checked on an Agilent 2100 and quantified using quantitative PCR (Q-PCR).

Reads were aligned to the *C. elegans* genome assembly WBCel235 using HISAT2 (Kim *et al*, 2015) for alignment, HTSeq (Anders *et al*, 2015) for gene expression quantification and DESeq2 (Love *et al*, 2014) for differential expression analysis. Genes with a fold change greater than 2 and a corrected *P*-value lower than 0.05 were retained. EnhancedVolcano (Blighe *et al*, 2022) was used to plot fold change versus *P*-values. Pathway enrichment analysis was performed using KEGG tool (www.genome.jp/kegg/) (Kanehisa, 2000). Heatmaps were performed in R (R Core Team, 2017) using the pheatmap package (Kolde, 2019). Read counts were centred and scaled for each gene to have mean zero and standard deviation one across the row.

Untargeted lipidomics assay

Young adults synchronised wild type and *unc-1(vlt10)* animals were collected in RIPA 1× buffer (Sigma-Aldrich-Merck) to obtain protein extract. We collected six biological samples per genotype in each independent experiment (n = 3 experiments and n = 10 cultures of worms per genotype). Total protein extract was quantified using the commercial kit (Pierce™ BCA Protein Assay kit #23225, Thermofisher) to normalise the lipid abundance of the samples. Samples were analysed using a liquid chromatography equipment coupled to a high-resolution mass spectrometer with an orbitrap detector (UPLC-Q-Exactive Plus) and an electrospray source (ESI) following the procedures optimised in the Analytical Unit (internal method not published). Samples and quality controls were randomly injected into the chromatographic system to avoid variability within the analytical sequence, as well as to improve the quality and reproducibility of the study. Data were acquired in in Full MS, DIA (data independent analysis) and DDA (data dependent analysis) scan modes and processed using an in-house script in the R software (v.3.6.1) with the XCMS and CAMERA packages for detection, filtering and alignment of spikes. The LipidMS library was used to identify candidate lipids (Alcoriza-Balaguer *et al*, 2019).

Imaging worms by microscopy

Fluorescence images were acquired using an SP5 confocal microscope and DM2500 vertical fluorescent microscope (Leica). Oil Red O images to analyse stained area per animal were taken with a digital dissecting microscope DMD108 (Leica). In all cases, animals

© 2023 The Authors

were mounted on agarose pads (2% on water) containing one drop of sodium acid (0.05 M) to anaesthetise them. Nematode selection and manipulation were performed using dissecting microscopes without or with fluorescence (MS5 and M165FC, Leica).

Statistics

Statistical analysis was performed using an analysis of variance test (one-way ANOVA) combined with a Tukey test to perform multiple comparisons of different strains and/or conditions. To perform comparisons between two conditions we used a Mann Whitney *t*-test to obtain the statistical significance of the data. In the graphs, we show the mean ± standard error (SEM). SEM is displayed as a bar, while asterisks show significant of the data (*P*-value) ($*P < 0.05$; $**P < 0.01$; $***P < 0.001$). The abbreviation "ns" means not significant.

Data availability

The transcriptomic data from this publication have been deposited to the Gene Expression Omnibus (https://www.ncbi.nlm.nih.gov/geo/) with the accession number GSE220662 (https://www.ncbi.nlm.nih.gov/geo/query/acc.cgi?acc=GSE220662).

Expanded View for this article is available online.

Acknowledgements

We wish to thank the CGC (funded by NIH Office of Research Infrastructure Programs; ref: P40 OD010440) and Phillip Morgan and Mario de Bono for worm strains. We are grateful to Antonio Miranda-Vizuete, Jorge Montesinos Selfa and Kaycie Butler for critical reading of the manuscript. We thank the Microscopy Unit and the Genomics Unit of IIS-La Fe, for their kind help. We also would like to thank Julián Cerón, for sharing protocols to induce CRISPR. We are grateful to Sara Torres and Howard Baylis for help and plasmids. We also thank AVAEH, the Valencian Association of the HD, for funding part of this work. RPVM held a "Miguel Servet" fellowship (Ref: CPII16/00004) funded by the Instituto de Salud Carlos III (ISCIII, Madrid, Spain) and by Social European Funds, and grants from the ISCIII (PI17/00011and PI20/00114). The funds from the ISCIII are partially supported by the European Regional Development Fund. RVM also received an Ayuda Miguel Gil grant to RPVM (VII Convocatoria Ayudas a la Investigación MHER, 2019, cofinanced by Colegio Oficial de Farmacéuticos de Sevilla and Fundación Cajasol) and a grant from Fundación Ramón Areces (CIVP19S8119). WRS is funded by the Medical Research Council, as part of United Kingdom Research and Innovation (MC-A023_5PB91).

Author contributions

Rafael P Vázquez-Manrique: Conceptualization; data curation; supervision; funding acquisition; investigation; visualization; methodology; writing – original draft; project administration; writing – review and editing. **Ana P Gómez-Escribano:** Conceptualization; data curation; formal analysis; validation; investigation; visualization; methodology; writing – original draft; writing – review and editing. **Carlos Mora-Martínez:** Conceptualization; resources; software; formal analysis; methodology; writing – review and editing. **Marta Roca:** Software; formal analysis; investigation; methodology; writing – review and editing. **Denise S Walker:** Formal analysis; investigation; methodology; writing – review and editing. **Joaquín Panadero:** Data curation; software; formal analysis; investigation. **Maria D Sequedo:** Investigation; methodology. **Ratni Saini:** Investigation. **Hans-Joachim Knölker:** Investigation. **Jose Blanca:** Investigation. **Juan Burguera:** Conceptualization; investigation. **Agustin Lahoz:** Conceptualization; validation; investigation; writing – review and editing. **Joaquin Cañizares:** Investigation. **José M Millán:** Conceptualization; investigation; writing – review and editing. **Nick O Burton:** Conceptualization; investigation; writing – original draft; writing – review and editing. **William R Schafer:** Investigation; writing – review and editing.

Disclosure and competing interests statement

The authors declare that they have no conflict of interest.

References

Alcoriza-Balaguer MI, García-Cañaveras JC, López A, Conde I, Juan O, Carretero J, Lahoz A (2019) LipidMS: an R package for lipid annotation in untargeted liquid chromatography-data independent acquisition-mass spectrometry lipidomics. *Anal Chem* 91: 836 – 845

Almanza A, Carlesso A, Chintha C, Creedican S, Doultsinos D, Leuzzi B, Luís A, McCarthy N, Montibeller L, More S *et al* (2019) Endoplasmic reticulum stress signalling—from basic mechanisms to clinical applications. *FEBS J* 286: 241 – 278

Altun ZF, Chen B, Wang Z-W, Hall DH (2009) High resolution map of *Caenorhabditis elegans* gap junction proteins. *Dev Dyn* 238: 1936 – 1950

Anders S, Pyl PT, Huber W (2015) HTSeq—a Python framework to work with high-throughput sequencing data. *Bioinformatics* 31: 166 – 169

Angeles-Albores D, Lee RYN, Chan J, Sternberg PW (2016) Tissue enrichment analysis for *C. elegans* genomics. *BMC Bioinformatics* 17: 366

Angeles-Albores D, Lee RYN, Chan J, Sternberg PW (2018) Two new functions in the WormBase Enrichment Suite. *MicroPubl Biol* 2018: 10.17912/W25Q2N

Antebi A (2015) Nuclear receptor signal transduction in *C. elegans*. *WormBook* 1 – 49

Antebi A, Yeh WH, Tait D, Hedgecock EM, Riddle DL (2000) daf-12 encodes a nuclear receptor that regulates the dauer diapause and developmental age in *C. elegans*. *Genes Dev* 14: 1512 – 1527

Bagamasbad P, Denver RJ (2011) Mechanisms and significance of nuclear receptor auto- and cross-regulation. *Gen Comp Endocrinol* 170: 3 – 17

Bi Y, Shi X, Zhu J, Guan X, Garbacz WG, Huang Y, Gao L, Yan J, Xu M, Ren S *et al* (2018) Regulation of cholesterol sulfotransferase SULT2B1b by hepatocyte nuclear factor 4α constitutes a negative feedback control of hepatic gluconeogenesis. *Mol Cell Biol* 38: e00654-17

Blighe K, Rana S, Lewis M (2022) *EnhancedVolcano: Publication-ready volcano plots with enhanced colouring and labeling*. R package version 1.16.0 https://github.com/kevinblighe/EnhancedVolcano

Bono-Yagüe J, Gómez-Escribano AP, Millán JM, Vázquez-Manrique RP (2020) Reactive species in Huntington disease: are they really the radicals you want to catch? *Antioxidants* 9: 577

Borowicz KK, Piskorska B, Banach M, Czuczwar SJ (2011) Neuroprotective actions of neurosteroids. *Front Endocrinol* 2: 50

Bouhours M, Po MD, Gao S, Hung W, Li H, Georgiou J, Roder JC, Zhen M (2011) A co-operative regulation of neuronal excitability by UNC-7 innexin and NCA/NALCN leak channel. *Mol Brain* 4: 16

Brenner S (1974) The genetics of *Caenorhabditis elegans*. *Genetics* 77: 71 – 94

Brock TJ, Browse J, Watts JL (2007) Fatty acid desaturation and the regulation of adiposity in *Caenorhabditis elegans*. *Genetics* 176: 865 – 875

Burton NO, Dwivedi VK, Burkhart KB, Kaplan REW, Baugh LR, Horvitz HR (2018) Neurohormonal signaling via a sulfotransferase antagonizes

 © 2023 The Authors

insulin-like signaling to regulate a *Caenorhabditis elegans* stress response. *Nat Commun* 9: 5152

Carroll BT, Dubyak GR, Sedensky MM, Morgan PG (2006) Sulfated signal from ASJ sensory neurons modulates stomatin-dependent coordination in *Caenorhabditis elegans*. *J Biol Chem* 281: 35989 – 35996

Chapman E, Ding S, Schultz PG, Wong C-H (2002) A potent and highly selective sulfotransferase inhibitor. *J Am Chem Soc* 124: 14524 – 14525

Chawla A (2001) Nuclear receptors and lipid physiology: opening the X-files. *Science* 294: 1866 – 1870

Chelur DS, Chalfie M (2007) Targeted cell killing by reconstituted caspases. *Proc Natl Acad Sci USA* 104: 2283 – 2288

Chen T (2008) Nuclear receptor drug discovery. *Curr Opin Chem Biol* 12: 418 – 426

Chen B, Liu Q, Ge Q, Xie J, Wang Z-W (2007) UNC-1 regulates gap junctions important to locomotion in *C. elegans*. *Curr Biol* 17: 1334 – 1339

Chen C, Li G, Liao W, Wu J, Liu L, Ma D, Zhou J, Elbekai RH, Edin ML, Zeldin DC *et al* (2009) Selective inhibitors of CYP2J2 related to terfenadine exhibit strong activity against human cancers *in vitro* and *in vivo*. *J Pharmacol Exp Ther* 329: 908 – 918

Cook SJ, Jarrell TA, Brittin CA, Wang Y, Bloniarz AE, Yakovlev MA, Nguyen KCQ, Tang LT-H, Bayer EA, Duerr JS *et al* (2019) Whole-animal connectomes of both *Caenorhabditis elegans* sexes. *Nature* 571: 63 – 71

Dahl G, Muller KJ (2014) Innexin and pannexin channels and their signaling. *FEBS Lett* 588: 1396 – 1402

Davis PJ, Tang H-Y, Hercbergs A, Lin H-Y, Keating KA, Mousa SA (2018) Bioactivity of thyroid hormone analogs at cancer cells. *Front Endocrinol* 9: 739

Fang B, Zhang M, Ren FZ, Zhou XD (2016) Lifelong diet including common unsaturated fatty acids extends the lifespan and affects oxidation in *Caenorhabditis elegans* consistently with hormesis model: life-long effects of dietary unsaturated fatty acids. *Eur J Lipid Sci Technol* 118: 1084 – 1092

Farina F, Lambert E, Commeau L, Lejeune F-X, Roudier N, Fonte C, Parker JA, Boddaert J, Verny M, Baulieu E-E *et al* (2017) The stress response factor daf-16/FOXO is required for multiple compound families to prolong the function of neurons with Huntington's disease. *Sci Rep* 7: 4014

Fenk LA, de Bono M (2015) Environmental CO2 inhibits Caenorhabditis elegans egg-laying by modulating olfactory neurons and evokes widespread changes in neural activity. *Proc Natl Acad Sci USA* 112: E3525 – E3534

Gamage N, Barnett A, Hempel N, Duggleby RG, Windmill KF, Martin JL, McManus ME (2006) Human sulfotransferases and their role in chemical metabolism. *Toxicol Sci* 90: 5 – 22

Garcia SM, Casanueva MO, Silva MC, Amaral MD, Morimoto RI (2007) Neuronal signaling modulates protein homeostasis in *Caenorhabditis elegans* post-synaptic muscle cells. *Genes Dev* 21: 3006 – 3016

Gilst MRV, Hadjivassiliou H, Jolly A, Yamamoto KR (2005) Nuclear hormone receptor NHR-49 controls fat consumption and fatty acid composition in *C. elegans*. *PLoS Biol* 3: e53

Goh GYS, Winter JJ, Bhanshali F, Doering KRS, Lai R, Lee K, Veal EA, Taubert S (2018) NHR-49/HNF4 integrates regulation of fatty acid metabolism with a protective transcriptional response to oxidative stress and fasting. *Aging Cell* 17: e12743

Gómez-Escribano A, Bono-Yagüe J, García-Gimeno M, Sequedo M, Hervás D, Fornés-Ferrer V, Torres-Sánchez S, Millán J, Sanz P, Vázquez-Manrique R (2020) Synergistic activation of AMPK prevents from polyglutamine-induced toxicity in *Caenorhabditis elegans*. *Pharmacol Res* 161: 105105

Goudeau J, Bellemin S, Toselli-Mollereau E, Shamalnasab M, Chen Y, Aguilaniu H (2011) Fatty acid desaturation links germ cell loss to longevity through NHR-80/HNF4 in *C. elegans*. *PLoS Biol* 9: e1000599

Han S, Schroeder EA, Silva-García CG, Hebestreit K, Mair WB, Brunet A (2017) Mono-unsaturated fatty acids link H3K4me3 modifiers to *C. elegans* lifespan. *Nature* 544: 185 – 190

Hattori K, Inoue M, Inoue T, Arai H, Tamura H (2006) A novel sulfotransferase abundantly expressed in the dauer larvae of *Caenorhabditis elegans*. *J Biochem* 139: 355 – 362

Hervé J-C, Derangeon M (2013) Gap-junction-mediated cell-to-cell communication. *Cell Tissue Res* 352: 21 – 31

Higuchi-Sanabria R, Durieux J, Kelet N, Homentcovschi S, de Los Rios Rogers M, Monshietehadi S, Garcia G, Dallarda S, Daniele JR, Ramachandran V *et al* (2020) Divergent nodes of non-autonomous UPRER signaling through serotonergic and dopaminergic neurons. *Cell Rep* 33: 108489

Hobert O (2013) The neuronal genome of *Caenorhabditis elegans*. *WormBook* 1 – 106

Hong F, Xu P, Zhai Y (2018) The opportunities and challenges of peroxisome proliferator-activated receptors ligands in clinical drug discovery and development. *Int J Mol Sci* 19: 2189

Imanikia S, Sheng M, Castro C, Griffin JL, Taylor RC (2019) XBP-1 remodels lipid metabolism to extend longevity. *Cell Rep* 28: 581 – 589

Joshi KK, Matlack TL, Pyonteck S, Vora M, Menzel R, Rongo C (2021) Biogenic amine neurotransmitters promote eicosanoid production and protein homeostasis. *EMBO Rep* 22: e51063

Kanehisa M (2000) KEGG: Kyoto encyclopedia of genes and genomes. *Nucleic Acids Res* 28: 27 – 30

Kang Z-S, Wang C, Han X-L, Wang B, Yuan H-L, Hou S-Y, Hao M-X, Du J-J, Li Y-Y, Zhou A-W *et al* (2018) Sulfonyl-containing phenyl-pyrrolyl pentane analogues: novel non-secosteroidal vitamin D receptor modulators with favorable physicochemical properties, pharmacokinetic properties and anti-tumor activity. *Eur J Med Chem* 157: 1174 – 1191

Kerr R, Lev-Ram V, Baird G, Vincent P, Tsien RY, Schafer WR (2000) Optical imaging of calcium transients in neurons and pharyngeal muscle of *C. elegans*. *Neuron* 26: 583 – 594

Kim D, Langmead B, Salzberg SL (2015) HISAT: a fast spliced aligner with low memory requirements. *Nat Methods* 12: 357 – 360

Kolde R (2019) pheatmap: pretty heatmaps. R package version 1.0.12

Lee D, An SWA, Jung Y, Yamaoka Y, Ryu Y, Goh GYS, Beigi A, Yang J-S, Jung GY, Ma DK *et al* (2019) MDT-15/MED15 permits longevity at low temperature via enhancing lipidostasis and proteostasis. *PLoS Biol* 17: e3000415

Leitman J, Ulrich Hartl F, Lederkremer GZ (2013) Soluble forms of polyQ-expanded huntingtin rather than large aggregates cause endoplasmic reticulum stress. *Nat Commun* 4: 2753

Love MI, Huber W, Anders S (2014) Moderated estimation of fold change and dispersion for RNA-seq data with DESeq2. *Genome Biol* 15: 550

Ludewig AH (2004) A novel nuclear receptor/coregulator complex controls *C. elegans* lipid metabolism, larval development, and aging. *Genes Dev* 18: 2120 – 2133

Maglich JM, Sluder A, Guan X, Shi Y, McKee DD, Carrick K, Kamdar K, Willson TM, Moore JT (2001) Comparison of complete nuclear receptor sets from the human, *Caenorhabditis elegans* and *Drosophila* genomes. *Genome Biol* 2: RESEARCH0029

Mark KA, Dumas KJ, Bhaumik D, Schilling B, Davis S, Oron TR, Sorensen DJ, Lucanic M, Brem RB, Melov S *et al* (2016) Vitamin D promotes protein homeostasis and longevity via the stress response pathway genes skn-1, ire-1, and xbp-1. *Cell Rep* 17: 1227 – 1237

Martin R, Däbritz F, Entchev EV, Kurzchalia TV, Knölker H-J (2008) Stereoselective synthesis of the hormonally active (25S)-Δ7-dafachronic acid, (25S)-Δ4-dafachronic acid, (25S)-dafachronic acid, and (25S)-cholestenoic acid. *Org Biomol Chem* 6: 4293 – 4295

Martin R, Entchev EV, Däbritz F, Kurzchalia TV, Knölker H-J (2009) Synthesis and hormonal activity of the (25*S*)-cholesten-26-oic acids—potent ligands for the DAF-12 receptor in *Caenorhabditis elegans*. *Eur J Org Chem* 2009: 3703 – 3714

© 2023 The Authors

Matyash V, Entchev EV, Mende F, Wilsch-Bräuninger M, Thiele C, Schmidt AW, Knölker H-J, Ward S, Kurzchalia TV (2004) Sterol-derived hormone(s) controls entry into diapause in *Caenorhabditis elegans* by consecutive activation of DAF-12 and DAF-16. *PLoS Biol* 2: e280

Mostafa YA, Taylor SD (2013) Steroid derivatives as inhibitors of steroid sulfatase. *J Steroid Biochem Mol Biol* 137: 183 – 198

Moutinho M, Codocedo JF, Puntambekar SS, Landreth GE (2019) Nuclear receptors as therapeutic targets for neurodegenerative diseases: lost in translation. *Annu Rev Pharmacol Toxicol* 59: 237 – 261

Ntambi JM, Miyazaki M, Stoehr JP, Lan H, Kendziorski CM, Yandell BS, Song Y, Cohen P, Friedman JM, Attie AD (2002) Loss of stearoyl-CoA desaturase-1 function protects mice against adiposity. *Proc Natl Acad Sci USA* 99: 11482 – 11486

Ohta A, Ujisawa T, Sonoda S, Kuhara A (2014) Light and pheromone-sensing neurons regulates cold habituation through insulin signalling in *Caenorhabditis elegans*. *Nat Commun* 5: 4412

Pathare PP, Lin A, Bornfeldt KE, Taubert S, Van Gilst MR (2012) Coordinate regulation of lipid metabolism by novel nuclear receptor partnerships. *PLoS Genet* 8: e1002645

Pérez-Jiménez MM, Monje-Moreno JM, Brokate-Llanos AM, Venegas-Calerón M, Sánchez-García A, Sansigre P, Valladares A, Esteban-García S, Suárez-Pereira I, Vitorica J *et al* (2021) Steroid hormones sulfatase inactivation extends lifespan and ameliorates age-related diseases. *Nat Commun* 12: 49

Pinne M, Raucy JL (2014) Advantages of cell-based high-volume screening assays to assess nuclear receptor activation during drug discovery. *Expert Opin Drug Discovery* 9: 669 – 686

R Core Team (2017) *R: a language and environment for statistical computing*. Vienna, Austria: R Foundation for Statistical Computing

Rabinowitch I, Chatzigeorgiou M, Schafer WR (2013) A gap junction circuit enhances processing of coincident mechanosensory inputs. *Curr Biol* 23: 963 – 967

Rajaram S, Sedensky MM, Morgan PG (1998) unc-1: a stomatin homologue controls sensitivity to volatile anesthetics in *Caenorhabditis elegans*. *Proc Natl Acad Sci USA* 95: 8761 – 8766

Rajaram S, Spangler TL, Sedensky MM, Morgan PG (1999) A stomatin and a degenerin interact to control anesthetic sensitivity in *Caenorhabditis elegans*. *Genetics* 153: 1673 – 1682

Rath VL, Verdugo D, Hemmerich S (2004) Sulfotransferase structural biology and inhibitor discovery. *Drug Discov Today* 9: 1003 – 1011

Saijo K, Crotti A, Glass CK (2010) Nuclear receptors, inflammation, and neurodegenerative diseases. In *Advances in Immunology*, Alt FW (ed), pp 21 – 59. Amsterdam: Elsevier

Sanchis A, García-Gimeno MA, Cañada-Martínez AJ, Sequedo MD, Millán JM, Sanz P, Vázquez-Manrique RP (2019) Metformin treatment reduces motor and neuropsychiatric phenotypes in the zQ175 mouse model of Huntington disease. *Exp Mol Med* 51: 1 – 16

Schackwitz WS, Inoue T, Thomas JH (1996) Chemosensory neurons function in parallel to mediate a pheromone response in *C. elegans*. *Neuron* 17: 719 – 728

Schulman IG (2010) Nuclear receptors as drug targets for metabolic disease. *Adv Drug Deliv Rev* 62: 1307 – 1315

Schulman IG, Heyman RA (2004) The flip side. *Chem Biol* 11: 639 – 646

Sedensky MM, Siefker JM, Morgan PG (2001) Model organisms: new insights into ion channel and transporter function. Stomatin homologues interact in *Caenorhabditis elegans*. *Am J Physiol Cell Physiol* 280: C1340 – C1348

Sever R, Glass CK (2013) Signaling by nuclear receptors. *Cold Spring Harb Perspect Biol* 5: a016709

Shacham T, Sharma N, Lederkremer GZ (2019) Protein misfolding and ER stress in Huntington's disease. *Front Mol Biosci* 6: 20

Shamim A, Mahmood T, Ahsan F, Kumar A, Bagga P (2018) Lipids: an insight into the neurodegenerative disorders. *Clin Nutr Exp* 20: 1 – 19

Silva MC, Amaral MD, Morimoto RI (2013) Neuronal reprograming of protein homeostasis by calcium-dependent regulation of the heat shock response. *PLoS Genet* 9: e1003711

Skerrett R, Malm T, Landreth G (2014) Nuclear receptors in neurodegenerative diseases. *Neurobiol Dis* 72: 104 – 116

Song J, Kim Y-S, Lee DH, Lee SH, Park HJ, Lee D, Kim H (2019) Neuroprotective effects of oleic acid in rodent models of cerebral ischaemia. *Sci Rep* 9: 10732

Steinbaugh MJ, Narasimhan SD, Robida-Stubbs S, Moronetti Mazzeo LE, Dreyfuss JM, Hourihan JM, Raghavan P, Operaña TN, Esmaillie R, Blackwell TK (2015) Lipid-mediated regulation of SKN-1/Nrf in response to germ cell absence. *Elife* 4: e07836

Taylor RC, Dillin A (2013) XBP-1 is a cell-nonautonomous regulator of stress resistance and longevity. *Cell* 153: 1435 – 1447

Taylor RC, Berendzen KM, Dillin A (2014) Systemic stress signalling: understanding the cell non-autonomous control of proteostasis. *Nat Rev Mol Cell Biol* 15: 211 – 217

Taylor SR, Santpere G, Weinreb A, Barrett A, Reilly MB, Xu C, Varol E, Oikonomou P, Glenwinkel L, McWhirter R *et al* (2021) Molecular topography of an entire nervous system. *Cell* 184: 4329 – 4347

Ujisawa T, Ohta A, Uda-Yagi M, Kuhara A (2016) Diverse regulation of temperature sensation by trimeric G-protein signaling in *Caenorhabditis elegans*. *PLoS One* 11: e0165518

Valastyan JS, Lindquist S (2014) Mechanisms of protein-folding diseases at a glance. *Dis Model Mech* 7: 9 – 14

Vicencio J, Martínez-Fernández C, Serrat X, Cerón J (2019) Efficient generation of endogenous fluorescent reporters by nested CRISPR in *Caenorhabditis elegans*. *Genetics* 211: 1143 – 1154

Walker DS, Schafer WR (2020) Distinct roles for innexin gap junctions and hemichannels in mechanosensation. *Elife* 9: e50597

Walker DS, Vázquez-Manrique RP, Gower NJD, Gregory E, Schafer WR, Baylis HA (2009) Inositol 1,4,5-trisphosphate signalling regulates the avoidance response to nose touch in *Caenorhabditis elegans*. *PLoS Genet* 5: e1000636

Wang Z, Stoltzfus J, You Y, Ranjit N, Tang H, Xie Y, Lok JB, Mangelsdorf DJ, Kliewer SA (2015) The nuclear receptor DAF-12 regulates nutrient metabolism and reproductive growth in nematodes. *PLoS Genet* 11: e1005027

Watts JL, Browse J (2002) Genetic dissection of polyunsaturated fatty acid synthesis in *Caenorhabditis elegans*. *Proc Natl Acad Sci USA* 99: 5854 – 5859

Webster CM, Pino EC, Carr CE, Wu L, Zhou B, Cedillo L, Kacergis MC, Curran SP, Soukas AA (2017) Genome-wide RNAi screen for fat regulatory genes in *C. elegans* identifies a Proteostasis-AMPK Axis critical for starvation survival. *Cell Rep* 20: 627 – 640

Yue X-H, Tong J-Q, Wang Z-J, Zhang J, Liu X, Liu X-J, Cai H-Y, Qi J-S (2016) Steroid sulfatase inhibitor DU-14 protects spatial memory and synaptic plasticity from disruption by amyloid β protein in male rats. *Horm Behav* 83: 83 – 92

License: This is an open access article under the terms of the Creative Commons Attribution License, which permits use, distribution and reproduction in any medium, provided the original work is properly cited.

© 2023 The Authors

Expanded View Figures

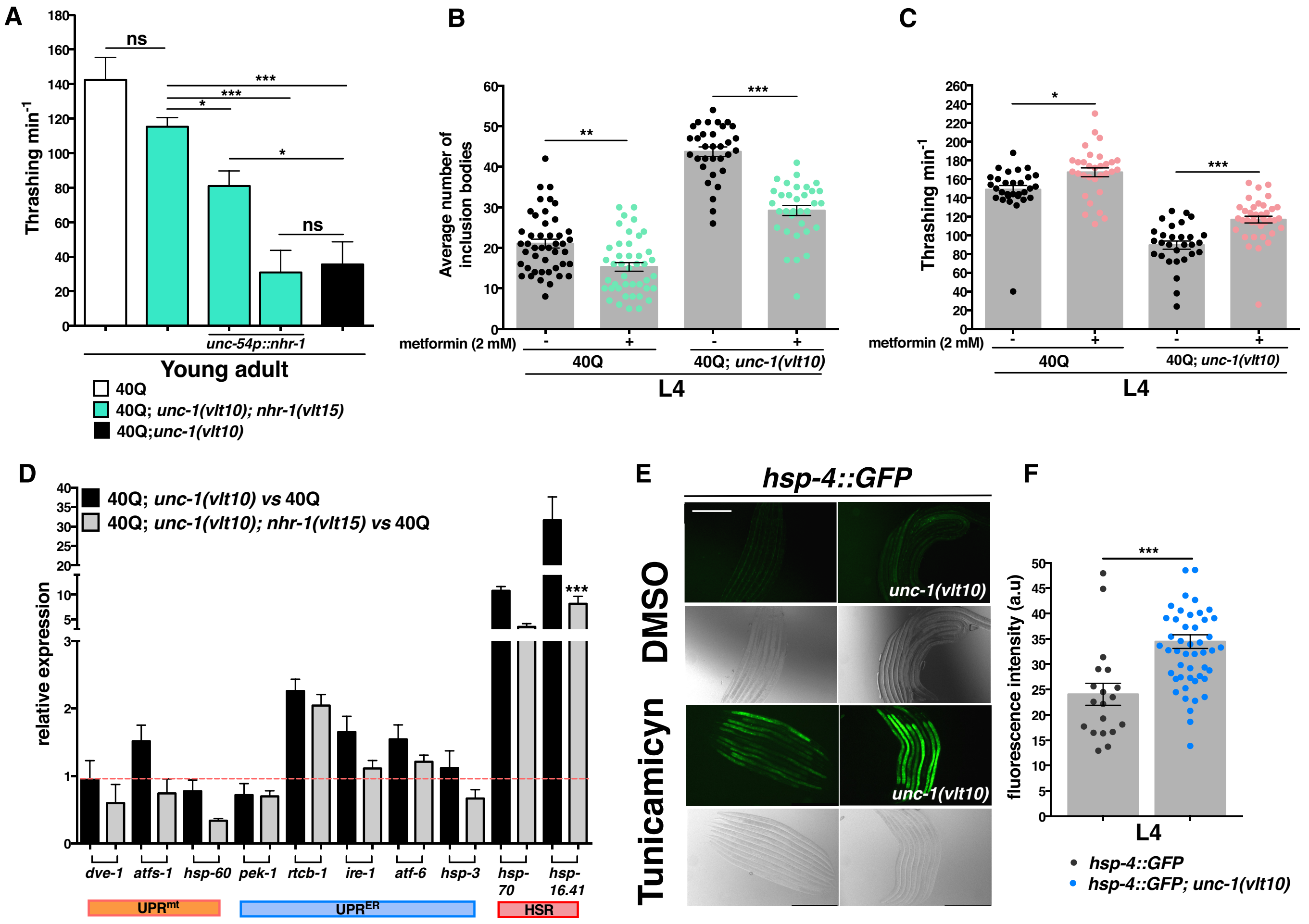

Figure EV1. Ablating *nhr-1* improves movement and health span and reduces *unc-1(vlt10)*-associated stress.

A Motility measurement after the muscle-specific restoration of NHR-1 in *unc-1(vlt10); nhr-1(vlt16)* young adults compared to non-rescued double mutants.

B, C The effects of metformin treatment in *unc-1* mutants as measured by the average number of polyQ inclusion bodies in muscle cells (B) and motility (C).

D The average expression levels of several genes related to the UPR in the endoplasmic reticulum, cytosol and mitochondria in 40Q; *unc-1(vlt10)* and 40Q; *unc-1 (vlt10); nhr-1(vlt15)* mutants as compared to 40Q (discontinuous red line).

E Representative images from wild type and *unc-1(vlt10)* animals expressing the *hsp-4::GFP* transgene, which induces stress in the endoplasmic reticulum. The expression of this transgene is activated by a mild treatment with tunicamycin (1 μg/ml). scale bar: 250 μm.

F Measured fluorescence intensity of the animals imaged in (E).

Data information: All plotted data show the mean ± standard error of the mean (SEM). At least 15 worms were analysed for thrashing assay and more than 30 animals were analysed for scoring inclusion bodies. UPR-stress gene expression was evaluated from three biological replicates and ER-stress reporter intensity was measured in more than 40 worms. $^*P < 0.05$; $^{**}P < 0.01$; $^{***}P < 0.001$; ns: not significant, as calculated using the one-way ANOVA with *post-hoc* Tukey test (graphs A–D) and the Mann–Whitney U-test (graph F).
Source data are available online for this figure.

© 2023 The Authors

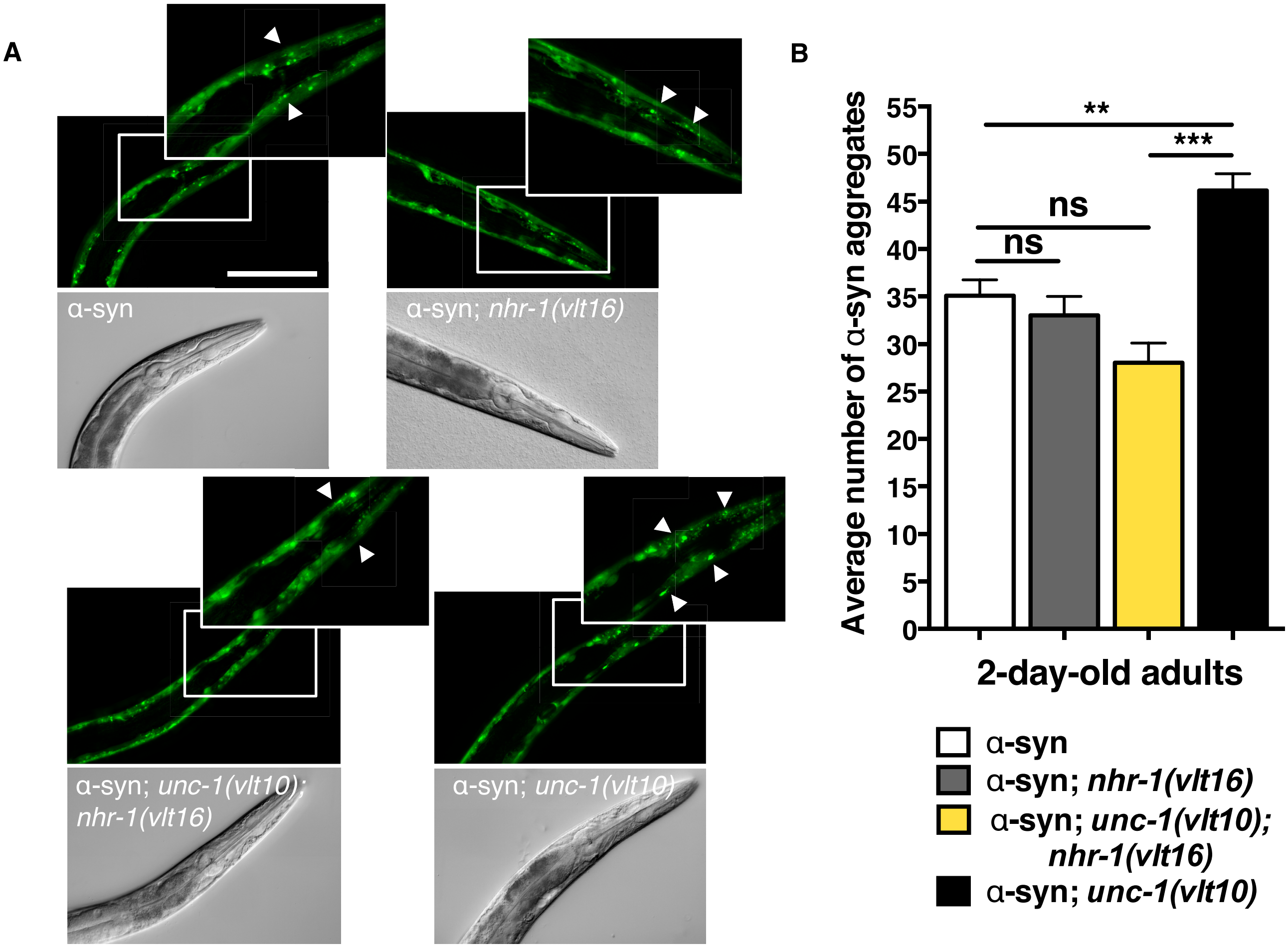

Figure EV2. NHR-1 modulates α-synuclein aggregation.

A Representative images of muscle pharyngeal cells of mutants containing *vlt10* and *vlt16* alleles in a α-synuclein background. Magnified insets show the two pharyngeal bulbs from which the α-synuclein aggregates (white arrows) were measured. Scale bar: 100 μm.

B The mean number of α-synuclein aggregates in *unc-1* animals bearing *nhr-1* loss of function alleles *vlt15* and *vlt16*.

Data information: The plotted data show the mean ± standard error of the mean (SEM). At least 30 animals were analysed in a three independent experiments. $^{**}P < 0.01$; $^{***}P < 0.001$; ns: not significant, as calculated using the one-way ANOVA with *post-hoc* Tukey test.
Source data are available online for this figure.

 © 2023 The Authors

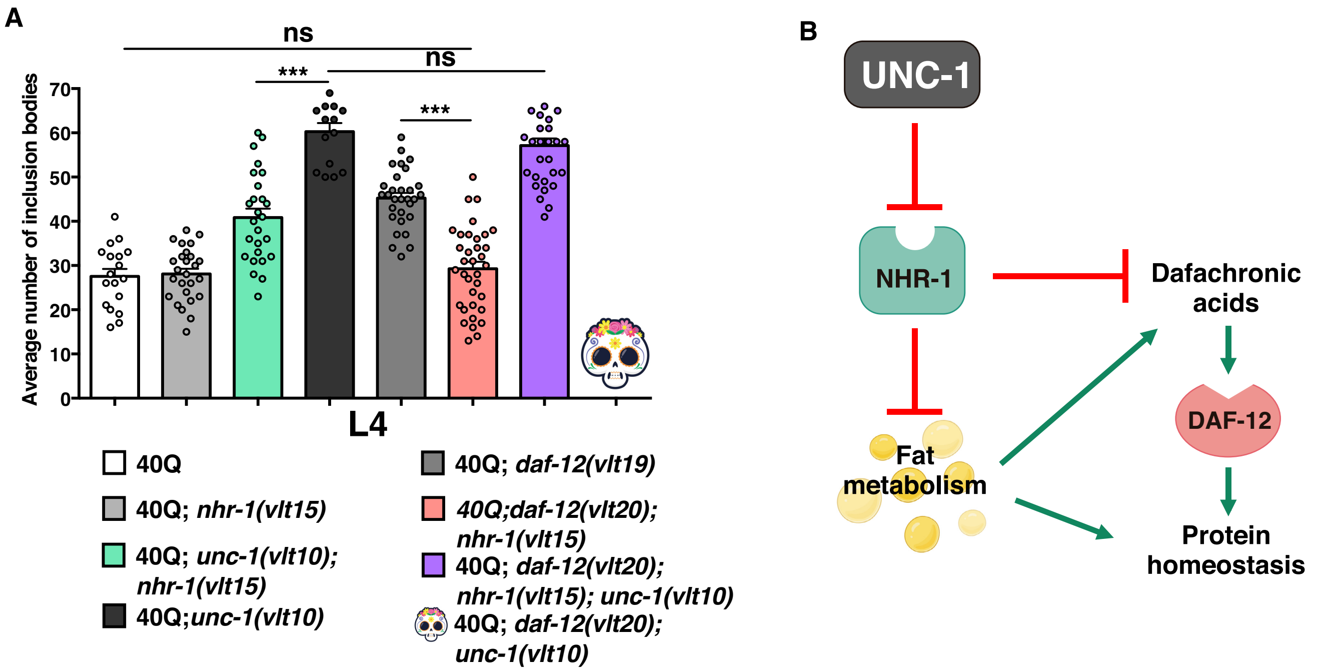

Figure EV3. NHR-1 and DAF-12 antagonization regulates protein homeostasis.

A The average number of polyQ inclusion bodies in muscle cells after *nhr-1* ablation in *daf-12* and *daf-12; unc-1* double mutants.

B A diagram showing the relationship between NHR-1 and DAF-12 in modulating protein homeostasis through fat metabolism changes and steroid hormonal signalling. The activation of NHR-1 represses the expression of genes involved in fat metabolism and dafachronic acid synthesis.

Data information: The plotted data show the mean ± standard error of the mean (SEM). At least 20 animals were analysed in a three independent experiments. ***$P < 0.001$; ns: not significant, as calculated using the one-way ANOVA with *post-hoc* Tukey test.
Source data are available online for this figure.

Figure EV4. Ablation of *unc-1* modifies downregulated and upregulated genes in *ssu-1*.

A Relative expression levels of genes that are not expressed in *ssu-1(fc73)* mutants or are downregulated but are upregulated in *unc-1(vlt10)* mutants.

B Genes that are upregulated in *ssu-1(fc73)* but are downregulated in *unc-1(vlt10)* mutants.

Data information: The plotted data show the mean ± standard error of the mean (SEM). FPKM values are from the transcriptomic analysis ($N = 6$ for each mutant strain) in Fig 5. *$P < 0.05$; **$P < 0.01$; ns: not significant. The statistical analysis was done using a Mann–Whitney *U*-test.
Source data are available online for this figure.

▶

© 2023 The Authors

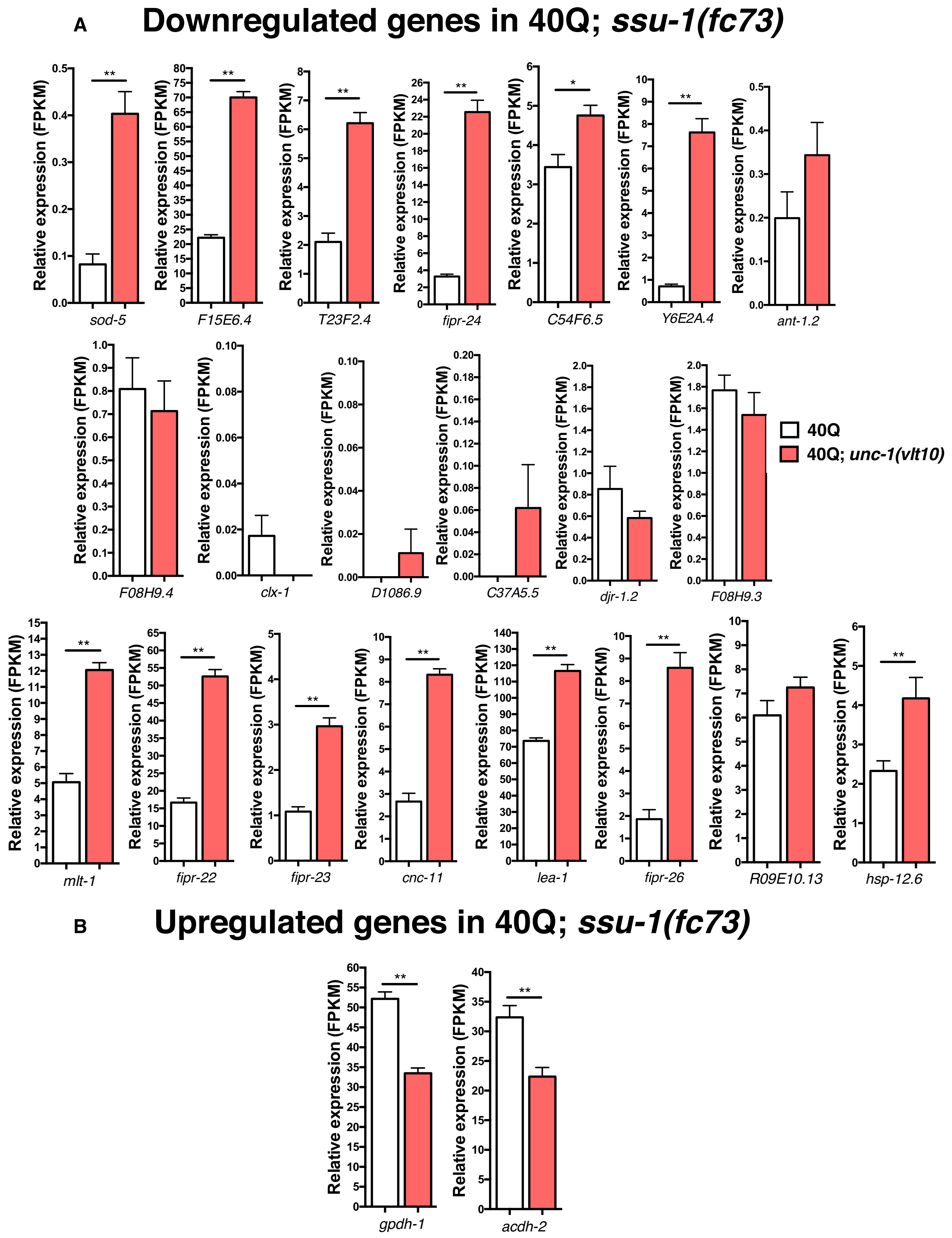

Figure EV4.

© 2023 The Authors

	unc-1(vlt10)	Wild type
Phospatidylcholines	55.8%	56.2%
Phosphatidyletanolamines	11.2%	12.5%
Monoacylglycerols*	8.4%	4.3%
Triglycerides*	6.6%	10.3%
Fatty acids	5.7%	5.5%
Lysophosphatidylcholines	3.8%	3.7%
Carnitines	2.2%	1.6%
Lysophosphatidylethanolamines	1.5%	1.3%
Sphingomyelins	0.9%	1.0%
Phosphoinositides	0.9%	0.6%
Phosphatidylglycerols	0.8%	0.4%
Ceramides	0.5%	1.5%
Sphingosine	0.4%	0.1%
Bile acids	0.4%	0.3%
Phosphatidylserines	0.4%	0.4%
Lysophosphatidylinositols	0.3%	0.4%
Diacylglycerols *	0.1%	0.1%

* Neutral Lipids

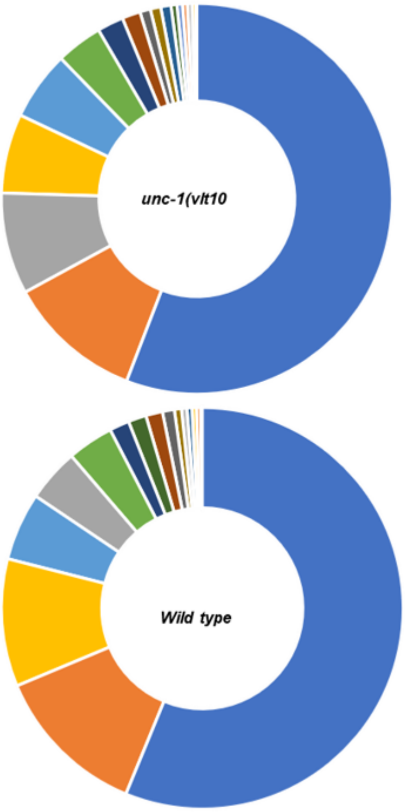

Figure EV5. Lipid abundance in *unc-1* mutants.

List of lipid classes detected in lipidomic assay in wild type and *unc-1(vlt10)* mutants. Asterisks show neutral lipids.
Data information: At least 12 worm biological samples containing more than 2000 animals per sample were analysed.

Source data are available online for this figure.

Appendix

Changes in lipid metabolism driven by steroid signalling modulate proteostasis in *C. elegans*

Gómez-Escribano AP, Mora-Martínez C, Roca M, Walker DS, Panadero J, Sequedo MD, Saini R, Knölker HJ, Blanca J, Burguera J, Lahoz A, Cañizares J, Millán JM, Burton NO, Schafer WR, Vázquez-Manrique RP*

*Corresponding author. Email: rafael_vazquez@iislafe.es

Appendix – Table of content

- Isolation of the *unc-1(vlt10)* mutation ……………………………………………2
- Appendix Figure S1……………………………………………………………4
- Appendix Figure S2……………………………………………………………5
- Appendix Figure S3……………………………………………………………6
- Appendix Figure S4……………………………………………………………7
- Appendix Figure S5……………………………………………………………8
- Appendix Figure S6……………………………………………………………9
- Appendix Figure S7……………………………………………………………10
- Appendix Figure S8……………………………………………………………11
- Appendix Figure S9……………………………………………………………12
- Appendix Table S1……………………………………………………………13
- Appendix Table S2……………………………………………………………13
- Appendix Table S3……………………………………………………………14
- Appendix Table S4……………………………………………………………15
- Appendix Table S5……………………………………………………………19
- Appendix References…………………………………………………………22

Isolation of the *unc-1(vlt10)* mutation

We induced random mutagenesis using 47 mM EMS (methanesulfonic acid ethyl ester, Sigma, St. Louis, Missouri, USA) in L4 animals of the AM141 strain. We incubated worms for 4 h at 20°C on this solution. After washing them they were pipetted onto NGM plates, seeded with OP50, and allowed them to lay the F1. F1 animals were bleached when they reached adulthood, and we searched among the F2 for animals uncoordinated and with abnormal aggregation patterns. Once isolated animals with the right phenotype (i.e. with abnormal motility and altered polyQ aggregation), they were outcrossed 5 times against the wild type background (N2, Bristol), before high-throughput sequencing of their genomic DNA. The reads provided by the sequencing service (Centre Nacional d'Anàlisi Genòmica – Centre de Regulacio Genomica, Barcelona, Spain) were mapped against the *C. elegans* WS245 reference using the mem algorithm implemented by the BWA software (Li, 2011, 2013). BAQ qualities were calculated and applied to the BAM alignments by using samtools calmd (Li, 2011). The SNP calling process was carried out by Freebayes (Garrison & Marth, 2012) with a minimum mapping quality of 57, a base quality threshold of 20, a minimum coverage of 6 and a minimum SNP quality of 20. A filtering process was stablished to look for the SNPs whose allelic frequencies were likely to have been affected by the selection process. SNPs were filtered out if they had more than one allele in all families, or if the reference allele was not present in any family, or if there were more than three mutant alleles when all families were considered. For the SNPs that passed all the filters a selection index was calculated. It consisted in the difference between the frequency of the most frequent allele in the back-crossed population and the frequency of that same allele in parental population, thus the SNPs with the highest differences between both populations would had the highest selection index. The predicted effect of each SNP was calculated by SnpEff (Cingolani *et al*, 2012).

To determine the molecular identity of this allele we sequenced the whole genome of this strain after six outcrossing steps and also the genome of the original strain (RVM10). EMS causes random lesions through the chromosomes and after outcrossing RVM10, most of these mutations would be lost during outcrossing, except the DNA changes that lie around the allele responsible for the aggregation phenotype. We took advantage of this, and when we compared both genomes

we observed a region with dense amount of mutations that "peaked" around the left arm of chromosome X (Appendix Figure S1A). Detailed analysis of this region showed that the *unc-1* gene, of RVM10, had a nonsense mutation in homozygosis that gives rise to premature stop codon, which would produce a putative null (Appendix Figure S1B). *unc-1* encodes a homologue of the Stomatin-like protein family from mammals (Lapatsina *et al*, 2012; Rajaram *et al*, 1998). The product of this gene has been shown to be involved in modulation of the electrical synapse (Chen *et al*, 2007) and sensitivity to anaesthetics (Rajaram *et al*, 1998). Null alleles of this gene are well-known to cause strong uncoordination (Chen *et al*, 2007), similar to *vlt10* mutant from our screen.

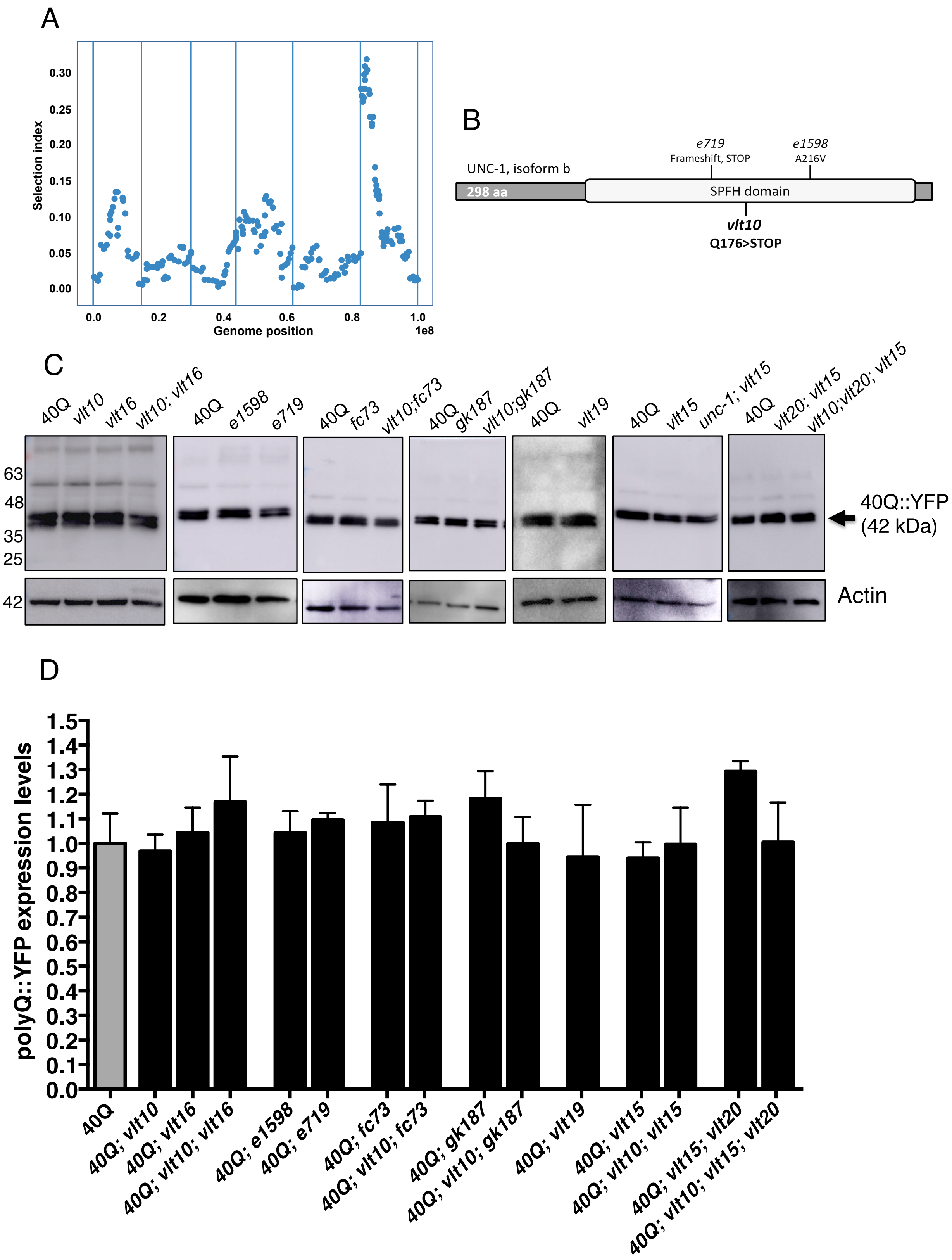
A
Selection index
Genome position
1e8
B
UNC-1, isoform b
298 aa
SPFH domain
e719
Frameshift, STOP
e1598
A216V
vlt10
Q176>STOP
C
40Q
vlt10
vlt16
vlt10; vlt16
e1598
e719
fc73
vlt10;fc73
gk187
vlt10;gk187
vlt19
vlt15
unc-1; vlt15
vlt20; vlt15
vlt10;vlt20; vlt15
40Q::YFP
(42 kDa)
Actin
D
polyQ::YFP expression levels
40Q
40Q; vlt10
40Q; vlt16
40Q; vlt10; vlt16
40Q; e1598
40Q; e719
40Q; fc73
40Q; vlt10; fc73
40Q; gk187
40Q; vlt10; gk187
40Q; vlt19
40Q; vlt15
40Q; vlt10; vlt15
40Q; vlt15; vlt20
40Q; vlt10; vlt15; vlt20

Appendix Figure S1. *vlt10* allele produces a premature stop codon in *unc-1/Stoml3,* which enhances polyQ aggregation without altering *40Q::YFP* transgene expression. (**A**) A map of SNPs used to determine the location of *vlt10* in the *C. elegans* genome. (**B**) Gene diagram showing the location in the SPFH domain of the premature stop codon (Q176 > STOP) produced by *vlt10* in *unc-1,* which likely produces a truncated UNC-1 and a putative loss of function protein. (**C**) Immunoblots showing the 40Q::YFP protein (42 kDa) in all genetic backgrounds. The double band identified by the anti-polyQ antibody most likely reflects proteolytic cleavage of 40Q::YFP. We used actin to normalize loading levels. (**D**) ImageJ quantification of 40Q::YFP protein levels in all genetic backgrounds, normalized to 40Q::YFP wild type (grey) to verify any changes according to transgene expression. All plotted data show the mean ± standard error of the mean (SEM). We performed ANOVA test, with post-hoc Tukey test, which shows that there were not significant differences between samples. Three biological samples were evaluated for each strain.

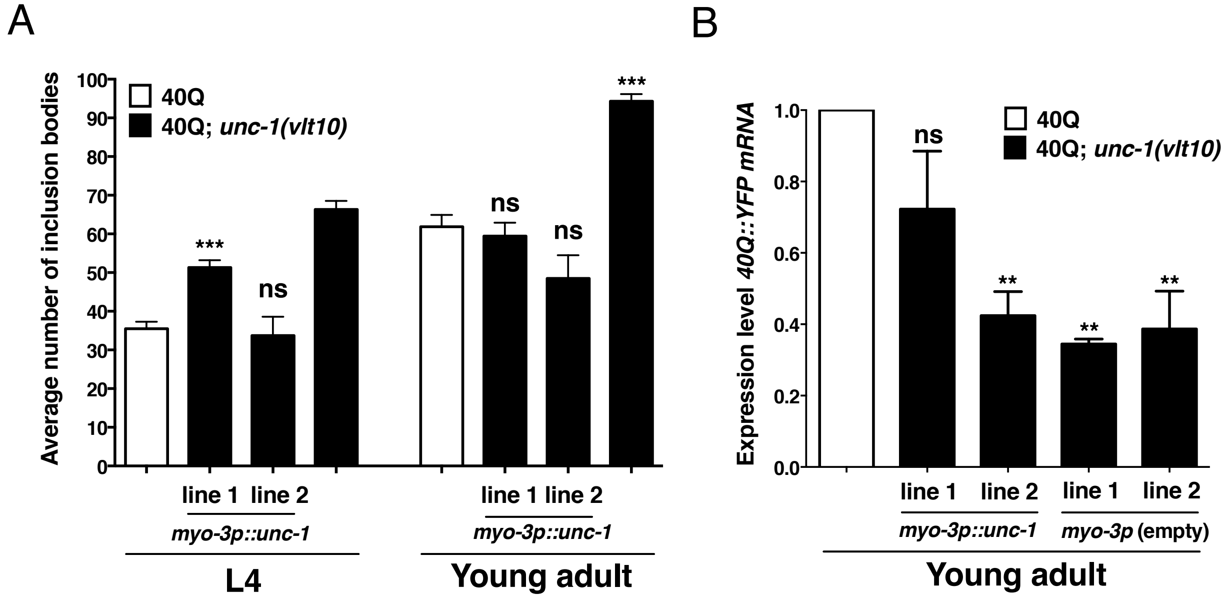

Appendix Figure S2. **Reintroduction of *unc-1* in muscle cells of *vlt10* mutants reduces *40Q::YFP* expression via a change in polyQ expression**. (**A**) The average number of polyQ inclusion bodies in muscle cells after introducing a construct containing the cDNA of *unc-1* under the control of the promoter of the *myo-3* gene in both transgenic stable lines (line 1 and line 2) expressing the *myo-3p::unc-1* (cDNA) transgene. (**B**) Relative expression levels of the *unc-54p::40Q::YFP* transgene in both transgenic stable lines (line 1 and line 2) expressing the *myo-3p::unc-1* (cDNA) or the *myo-3p* promoter-only control transgene.. Data information: The plotted data show mean ± standard error of the mean (SEM). At least thirty animals were used and at least three independent experiments were performed per experiment and condition. ****P*

< 0.001; ***P* < 0.01; ns: not significant, as calculated using the one-way ANOVA with post-hoc Tukey test.

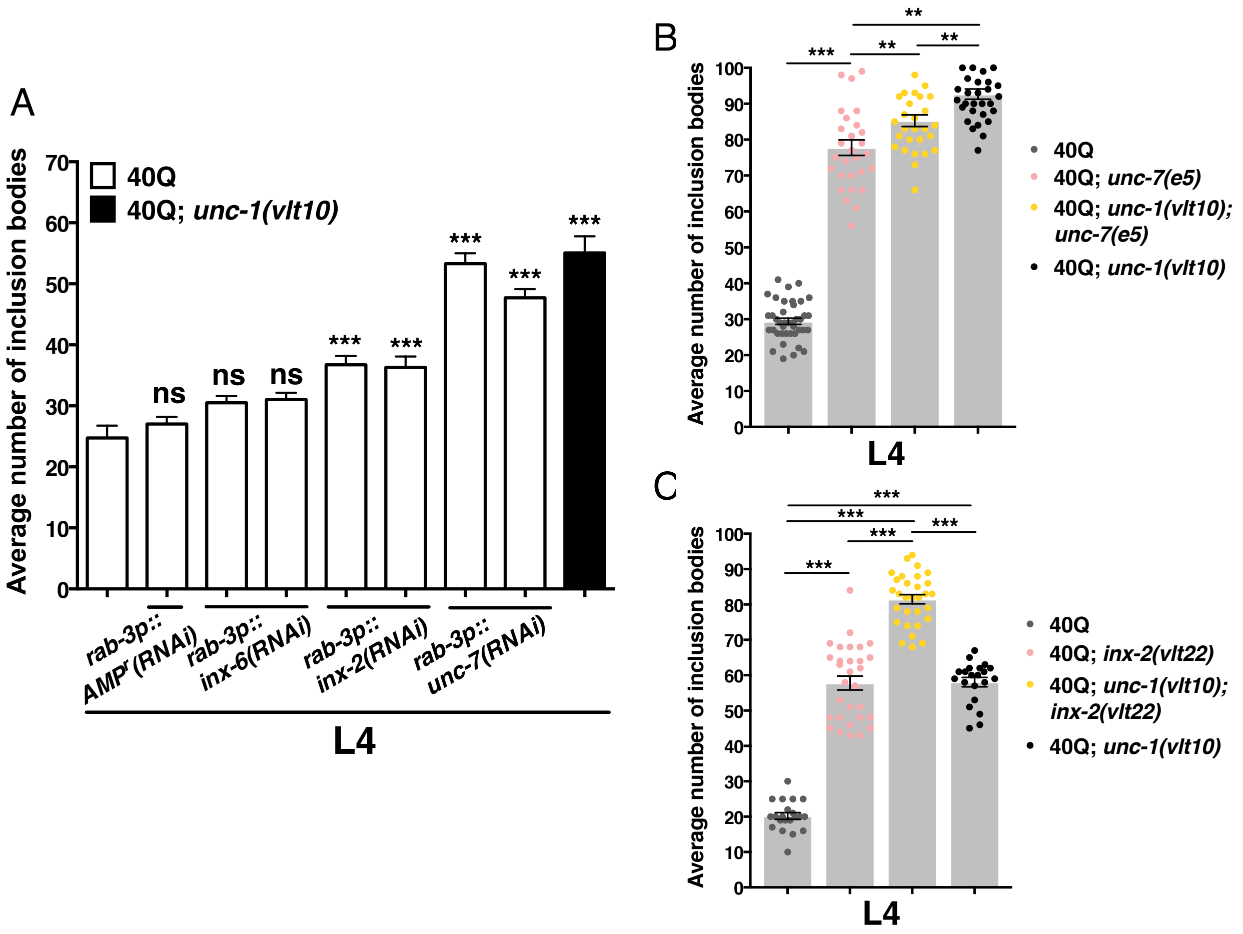

Appendix Figure S3. Neuronal disruption of innexins INX-2 and UNC-7 enhances aggregation of polyQs in muscle cells. (**A**) The average number of polyQ inclusion bodies after tissue-specific silencing of *inx-2*, *inx-6* and *unc-7* in neurons. (**B**) The average number of polyQ inclusion bodies in muscle cells for double mutant *unc-7(e5)*; *unc-1(vlt10)* compared with the single mutants and wild type animals . (**C**) The average number of polyQ inclusion bodies for double mutant *unc-1(vlt10); inx-2(vlt22)* compared with the single mutants and wild type animals. Data information: The plotted data show mean ± standard error of the mean (SEM). Thirty animals per condition and/or strain and per experiment were analysed. Each analysis has been reproduced at least three times. ***P* < 0.001; ***P* < 0.01; ns: not significant in reference to the wild type *40Q::YFP* strain (graph A), as calculated using the one-way ANOVA with post-hoc Tukey test.

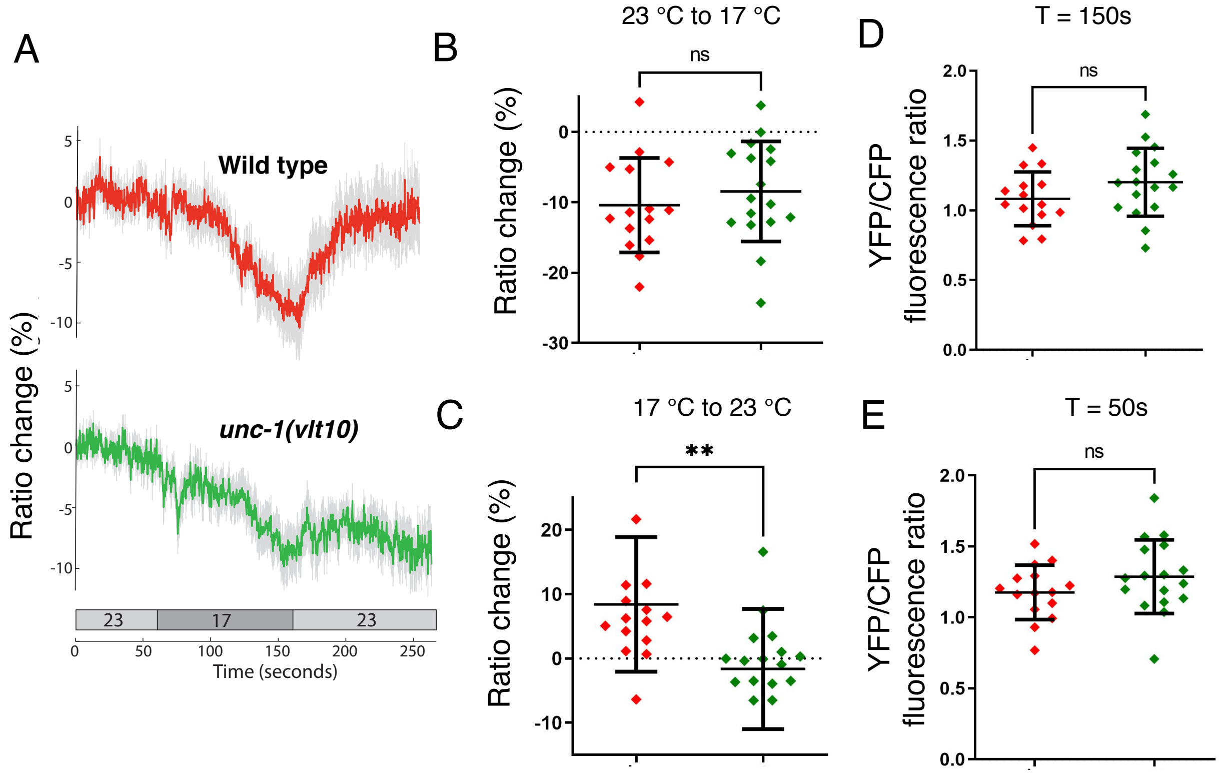

Appendix Figure S4. Calcium imaging in ASJ of *unc-1* mutants. (**A**–**E**) Calcium imaging in ASJ neurons with and without *unc-1(vlt10)* transgene in response to temperature shifts. (**A**) Mean traces of % YFP/CFP fluorescence ratio change. Grey trace indicates SEM, bar at the bottom indicates the perfusion temperature. (**B**) Scatter plot showing individual ratio changes in response to the temperature shift from 23°C to 17°C, made by comparing the mean YFP/CFP fluorescence ratio at T = 50s with T = 150s using a 20-frame window. (**C**) Scatter plot showing individual ratio changes in response to the temperature shift from 17°C to 23°C, made by comparing YFP/CFP fluorescence ratio at T = 150s with T = 50s. (**D**–**E**) Scatter plots showing the YFP/CFP fluorescence ratio at T = 150s (D) and T = 50s (E). Error bars show mean ± SEM. Data information: n = 15 (wild type), 17 (*unc-1(vlt10)* animals, recorded over at least three days. ** P < 0.01, ns: not significant, as calculated using unpaired t-test.

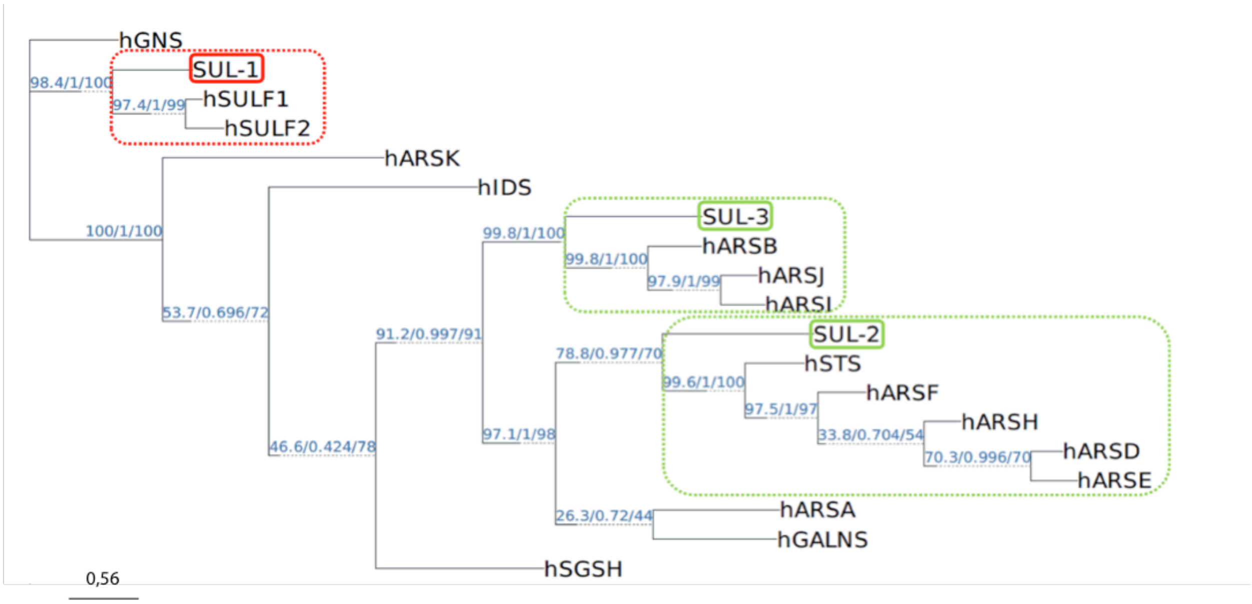

Appendix Figure S5. Phylogenetic analysis of *C. elegans* and human sulfatases. The diagram shows the phylogenetic tree of all *C. elegans* sulfatases (SUL-1, SUL-2 and SUL-3) obtained using the MUSCLE (Edgar, 2004) and IQ-TREE version 1.6.8 software (Chernomor *et al*, 2016; Li, 2013; Nguyen *et al*, 2015). The phylogenetic tree shows bootstrap values which provides confidence values for each node. This analysis suggests that SUL-2 and SUL-3 are closer to arylsulfatases, while SUL-1 is closer to hSULF1 and hSULF2.

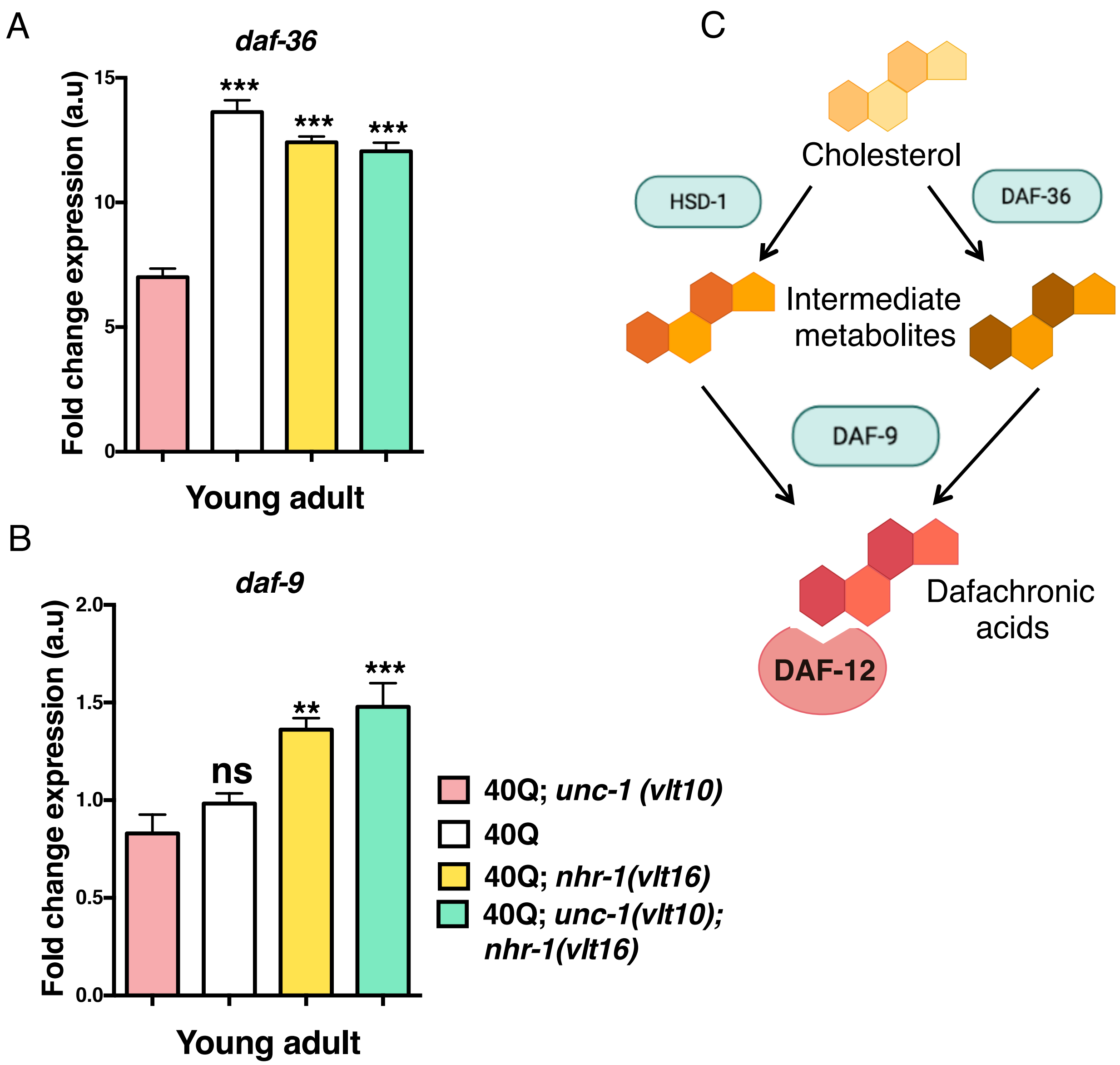

Appendix Figure S6. NHR-1 regulates the expression of some genes related to DAF-12 signalling. (**A**) Graph of the relative expression of *daf-36*, a gene that encodes an oxidoreductase enzyme involved in cholesterol processing, in *unc-1* and *nhr-1* single and double mutants compared to wild type worms. (**B**) Graph of the relative expression of *daf-9*, a cytochrome involved in dafachronic acids synthesis, in *unc-1* and *nhr-1* single and double mutants compared to wild type worms. (**C**) Diagram of parts of the synthesis pathway of dafachronic acids, placing the activity of the HSD-1, DAF-36 and DAF-9 enzymes. Data information: The plotted data show mean ± standard error of the mean (SEM). Six biological replicates were analysed for each strain. $^{**}P < 0.01$; $^{***}P < 0.005$, as calculated using the one-way ANOVA with post-hoc Tukey test and compared to *unc-1* mutants

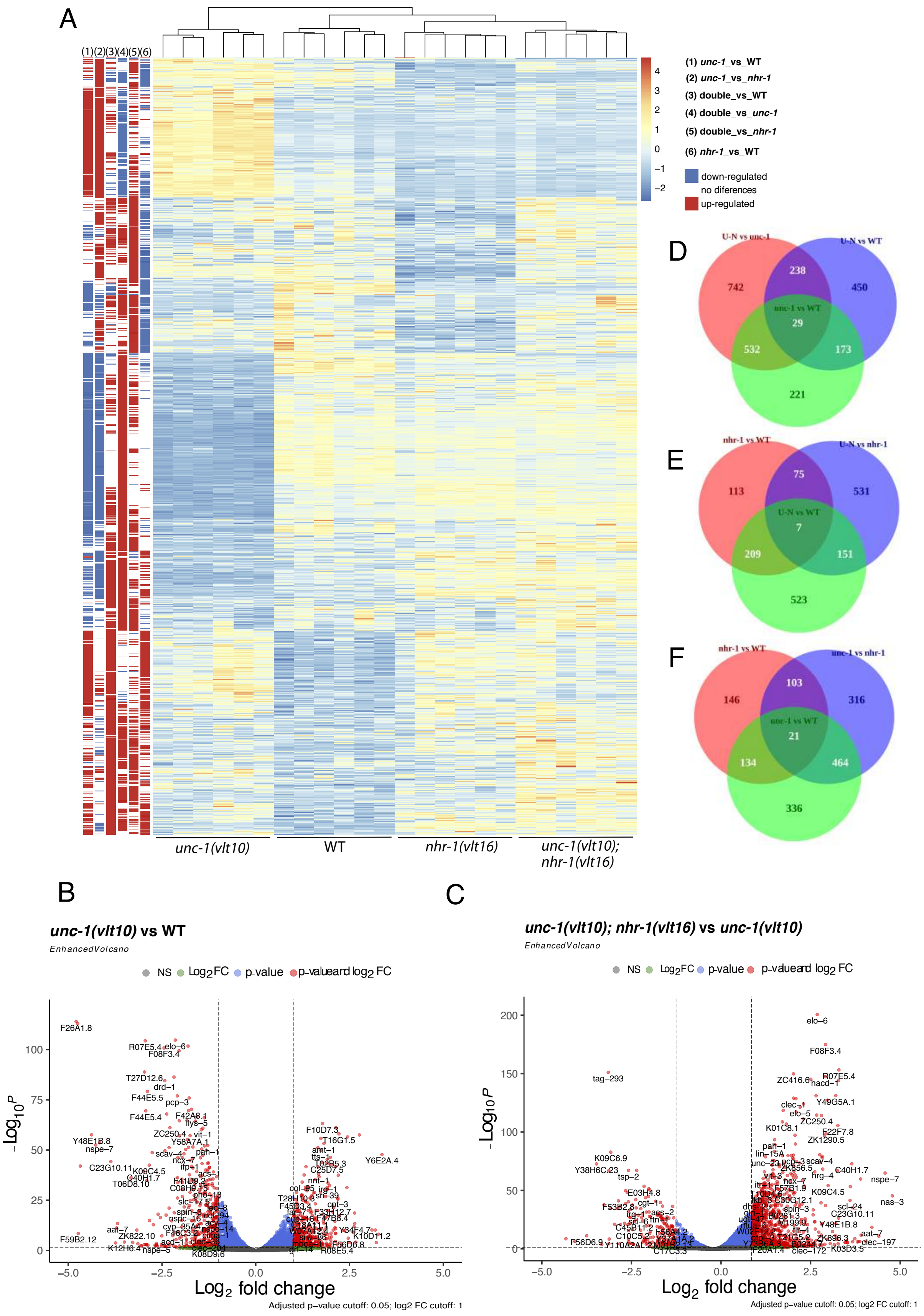
A
(1)(2)(3)(4)(5)(6)
(1) unc-1_vs_WT
(2) unc-1_vs_nhr-1
(3) double_vs_WT
(4) double_vs_unc-1
(5) double_vs_nhr-1
(6) nhr-1_vs_WT
down-regulated
no diferences
up-regulated
unc-1(vlt10)
WT
nhr-1(vlt16)
unc-1(vlt10); nhr-1(vlt16)
D
U-N vs unc-1
U-N vs WT
unc-1 vs WT
742
238
450
29
532
173
221
E
nhr-1 vs WT
U-N vs nhr-1
U-N vs WT
113
75
531
7
209
151
523
F
nhr-1 vs WT
unc-1 vs nhr-1
unc-1 vs WT
146
103
316
21
134
464
336
B
unc-1(vlt10) vs WT
EnhancedVolcano
NS Log2FC p-value p-value and log2 FC
Log2 fold change
−Log10 P
Adjusted p−value cutoff: 0.05; log2 FC cutoff: 1
C
unc-1(vlt10); nhr-1(vlt16) vs unc-1(vlt10)
EnhancedVolcano
NS Log2FC p-value p-value and log2 FC
Log2 fold change
−Log10 P
Adjusted p−value cutoff: 0.05; log2 FC cutoff: 1

Appendix Figure S7. Transcriptomic signature of *unc-1* and *nhr-1* mutants into a polyQ background. (A) Heatmap showing DESeq2 normalized read counts of differentially expressed genes (corrected $P < 0.05$ and fold change > 2, see Methods). Data were scaled and centred by gene. Heat map rows are based on Z-scores. The left side of the heatmap includes the statistically significant changes between genotypes [1: 40Q; *unc-1(vlt10)* vs 40Q; 2: 40Q; *unc-1(vlt10)* vs 40Q; *nhr-1(vlt16)*; 3: double mutant vs 40Q; 4: double mutant vs 40Q; *unc-1(vlt10)*; 5: double mutant vs 40Q; *nhr-1(vlt16);* and 6; *nhr-1(vlt16)* vs 40Q]. **(B–C)** Volcano plots showing the log2 fold change versus the -log10 of the DESeq2 corrected p-value for each pair of genotypes (40Q; *unc-1* vs 40Q and 40Q; *unc-1;nhr-1* vs 40Q; *unc-1*). **(D–F)** Venn diagrams showing the number of genes that are differentially expressed between genotypes. For example, graph D shows that there are 523 genes that are differentially expressed between double and 40Q; *unc-1* mutants and also between *unc-1* and 40Q, but not between double mutants and 40Q. This shows that these genes are rescued by the *vlt16* allele. In contrast, there are 173 differentially expressed genes that are specific to animals carrying the *vlt10* allele and that are not altered by *vlt16*.

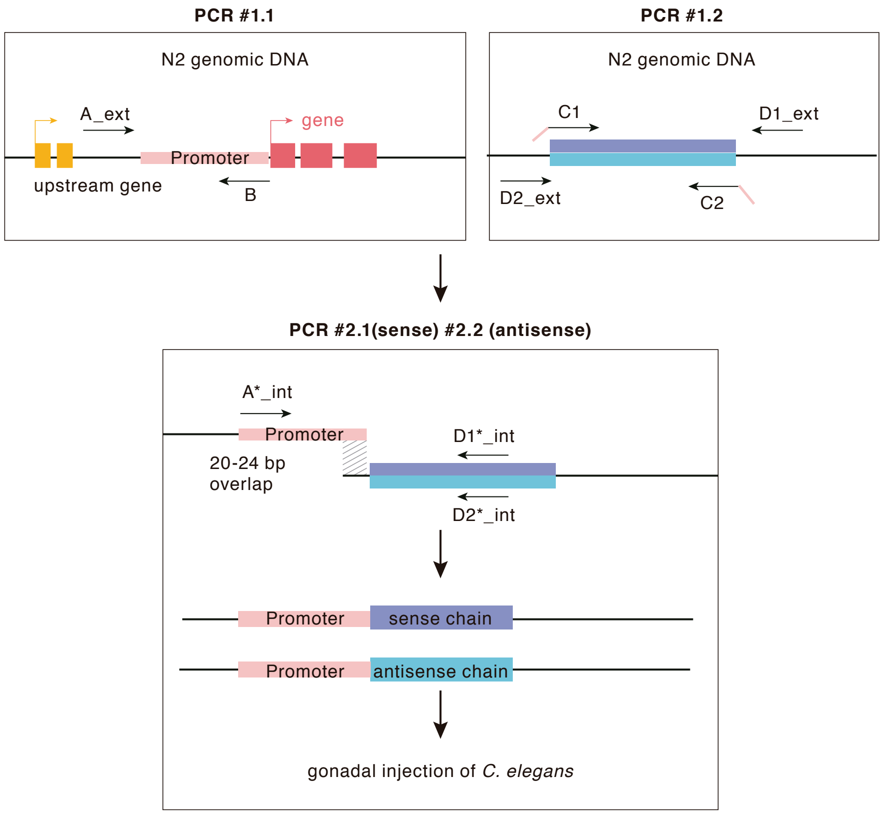

Appendix Figure S8. Diagram showing the production of PCR-generated constructs to induce RNAi. A promoter sequence for a gene fragment was assembled to promote tissue-specific RNAi via fusion PCR. External (ext) and internal (int) primers were used to amplify the promoter region (red), sense (purple) and antisense (blue) sequence from genomic DNA. The primers had 20 complementary nucleotides (red tail on primers) that allow fusion using nested primers.

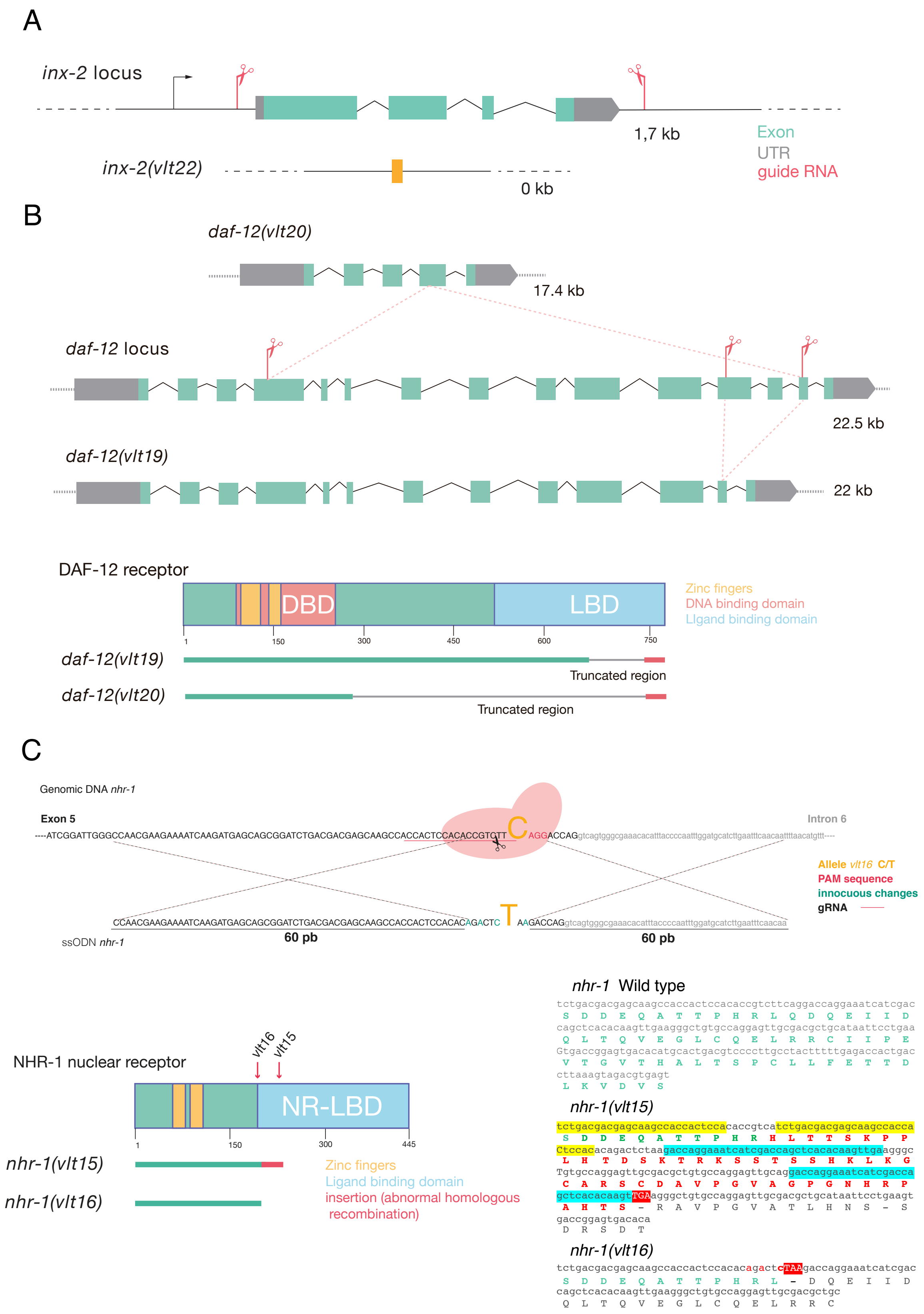
A
inx-2 locus
1,7 kb
Exon
UTR
guide RNA
inx-2(vlt22)
0 kb
B
daf-12(vlt20)
17.4 kb
daf-12 locus
22.5 kb
daf-12(vlt19)
22 kb
DAF-12 receptor
DBD
LBD
1
150
300
450
600
750
Zinc fingers
DNA binding domain
LIgand binding domain
daf-12(vlt19)
Truncated region
daf-12(vlt20)
Truncated region
C
Genomic DNA nhr-1
Exon 5
Intron 6
----ATCGGATTGGGCCAACGAAGAAAATCAAGATGAGCAGCGGATCTGACGACGAGCAAGCCACCACTCCACACCGTCTT C AGGACCAGgtcagtgggcgaaacacatttaccccaatttggatgcatcttgaatttcaacaattttaacatgttt----
CCAACGAAGAAAATCAAGATGAGCAGCGGATCTGACGACGAGCAAGCCACCACTCCACACAGACTC T AAGACCAGgtcagtgggcgaaacacatttaccccaatttggatgcatcttgaatttcaacaa
60 pb
60 pb
ssODN nhr-1
Allele vlt16 C/T
PAM sequence
innocuous changes
gRNA
nhr-1 Wild type
tctgacgacgagcaagccaccactccacaccgtcttcaggaccaggaaatcatcgac
S D D E Q A T T P H R L Q D Q E I I D
cagctcacacaagttgaagggctgtgccaggagttgcgacgctgcataattcctgaa
Q L T Q V E G L C Q E L R R C I I P E
Gtgaccggagtgacacatgcactgacgtccccttgcctacttttttgagaccactgac
V T G V T H A L T S P C L L F E T T D
cttaaagtagacgtgagt
L K V D V S
nhr-1(vlt15)
tctgacgacgagcaagccaccactccacaccgtcatctgacgacgagcaagccacca
S D D E Q A T T P H R H L T T S K P P
Ctccacacagactctaagaccaggaaatcatcgaccagctcacacaagttgaagggc
L H T D S K T R K S S T S S H K L K G
Tgtgccaggagttgcgacgctgtgccaggagttgcaggaccaggaaatcatcgacca
C A R S C D A V P G V A G P G N H R P
gctcacacaagtTGAagggctgtgccaggagttgcgacgctgcataattcctgaagt
A H T S - R A V P G V A T L H N S - S
gaccggagtgacaca
D R S D T
nhr-1(vlt16)
tctgacgacgagcaagccaccactccacacagactcTAAgaccaggaaatcatcgac
S D D E Q A T T P H R L - D Q E I I D
cagctcacacaagttgaagggctgtgccaggagttgcgacgctgc
Q L T Q V E G L C Q E L R R C
NHR-1 nuclear receptor
vlt16
vlt15
NR-LBD
1
150
300
445
nhr-1(vlt15)
nhr-1(vlt16)
Zinc fingers
Ligand binding domain
insertion (abnormal homologous recombination)

Appendix Figure S9. CRISPR strategies to modify gene expression. (**A**) The *inx-2* gene was disrupted using two sgRNAs that cut all coding sequences of the gene. The *vlt22* allele obtained from this experiment represents a complete loss of the *inx-2* gene. (**B**) The *daf-12* gene was disrupted using a combination of two gRNAs that target exons 12 and 14. *vlt19* is a 500-bp deletion that affects the ligand-binding domain (LBD) in the C-terminal region of the DAF-12 protein. (**C**) Diagram to introduce the *vlt16* mutation in *nhr-1,* which emulates the allele *n6242* and the novel *vlt15* allele as a result of an abnormal homologous recombination containing duplicate insertions (yellow and blue shading). *vlt15* induces a frameshift (red pattern) that produces a change (C/T) and a premature TGA stop codon upstream. Both alleles, *vlt16* and *vlt15*, are putative nulls.

Appendix Table S1. Modifier genes isolated in this work.

Gene	Orthologue in mammals	Mutation	Type of modifier of polyQ aggregation
unc-1	Stomatin like protein 3	Putative null (nonsense mutation)	Enhancer
tmem-24	C2CD2	Putative null (nonsense mutation)	Enhancer
pqn-62	Prion-like protein (no orthologue known in mammals)	Putative null (nonsense mutation)	Enhancer
sir-2.3	SIRT4	Putative null (nonsense mutation)	Suppressor

Appendix Table S2. Relative expression level of 40Q::YFP transgene.

	40Q	***40Q; unc-1(vlt10) +*** ***myo-3p::unc-1(cDNA)*** **line 1**	***40Q; unc-1(vlt10) +*** ***myo-3p::unc-1(cDNA)*** **line 2**	***40Q; unc-1(vlt10) +*** ***myo-3p*** **line 1**	***40Q; unc-1(vlt10)*** **+** ***myo-3p*** **line 2**
**EXP_1**	**1**	**0.4**	**0.521**	**0.348**	**0.553**

**EXP_2**	**1**	**0.849**	**0.294**	**0.319**	**0.418**
**EXP_3**	**1**	**0.919**	**0.457**	**0.367**	**0.189**

Table S3. Stomatin-like proteins and innexins are required to maintain protein homeostasis

Genotype	Focis (%)[1]	P value[2]	Protein	Function[3]	Expression[3]
unc-1(vlt10)	171.7 ± 3.1	***	Stomatin-like protein	Locomotion<br>Sensitivity to volatile anaesthetics	NS-WE[4]
unc-24(e138)	149.1 ± 3.8	***	Stomatin-like protein	Locomotion<br>Sensitivity to volatile anaesthetics	NS-WE
mec-2(e75)	114.5 ± 5.2	ns	Stomatin-like protein	Mechanosensation	Mechanosensory neurons
unc-7(e5)	178.7 ± 4.7	***	Innexin	Locomotion<br>Sensitivity to volatile anaesthetics<br>Sensitivity to ivermectin	NS[4]<br>Glial cells<br>Mesoderm
unc-9(ec27)	185.4 ± 2.7	***	Innexin	Locomotion<br>Sensitivity to volatile anaesthetics<br>Sensitivity to ivermectin<br>Electrical coupling of muscle cells	NS-WE<br>Enteric muscles<br>RS[4]<br>Hypodermis

					Glial cells<br>Embryos
inx-2(vlt22)	175.4 ± 4.3	***	Innexin	Role initial cellular proliferation stage	NS-AVK neuron<br>Pharynx<br>Intestine<br>Embryos
inx-7(ok2319)	98.7 ± 3.8	ns	Innexin	Unknown	NS-WE<br>Pharynx<br>RS<br>Mesoderm<br>Embryos
inx-6(rr5)	98.8 ± 4.1	ns	Innexin	Synchronized pharyngeal muscle contractions	Pharynx<br>Mesoderm<br>Embryos

[1]Average of inclusion bodies normalised with the background of wildtype siblings ±Standard Error of the Mean.
[2]ANOVA with post-hoc analysis of Tukey: ***p<0.001 referred to wild type; ns: no statistically significant.
[3]Function and expression according by the review of Altun et al., (Altun et al., 2009).
[4]NS-WE: nervous system-wide expression; NS: some neurons; RS: reproductive system.

Appendix Table S4. *C. elegans* strains used in this work.

Strain[1]	Genotype	Reference
Bristol N2	*Caenorhabditis elegans* wild type background	Brenner, 1974
AM141	*rmIs133[unc-54p::40Q::YFP] X*	Morley et al., 2002
RVM10	*rmIs133[unc-54p::40Q::YFP] X*; *unc-1(vlt10) X*	This work
CB719	*unc-1(e719)* X	Park and Horvitz, 1986
CB1598	*unc-1(e1598) X*	Park and Horvitz, 1986
RVM241	*unc-1(vlt10) X*	This work
RVM23	*rmIs133[unc-54p::40Q::YFP] X*; *unc-1(e719) X*	This work
RVM20	*rmIs133[unc-54p::40Q::YFP] X*; *unc-1(e1598) X*	This work
AM140	*rmIs132[unc-54p::35Q::YFP] X*	Morley et al., 2002
RVM21	*rmIs132[unc-54p::35Q::YFP]; unc-1(e1598) X*	This work
RVM24	*rmIs132[unc-54p::35Q::YFP] X; unc-1(e719) X*	This work
RVM26	*rmIs132[unc-54p::35Q::YFP] X; unc-1(vlt10) X*	This work
NL5901	*pkIs2386[unc-54p::α-synucleina::YFP + unc-119(+)] IV*	van Ham et al., 2008
CL2006	*dvIs2[pCL12(unc-54/Abeta human peptide 1-42 minigene)+pRF4]*	Link, 1995
RVM317	*pkIs2386[unc-54p::α-synuclein::YFP + unc-119(+)] IV; unc-1(vlt10) X*	This work
RVM328	*dvIs2[pCL12(unc-54/Abeta human peptide 1-42 minigene)+pRF4], unc-1(vlt10) X*	This work
RVM47	*rmIs133[unc-54p::40Q::YFP] X; unc-1(vlt10) X; vltEx47[myo-3p::unc-1(o/e); myo-2p::mCherry strain 1]*	This work
RVM155	*rmIs133[unc-54p::40Q::YFP] X; unc-1(vlt10) X; vltEx155[myo-3p::unc-1(o/e); myo-2p::mCherry strain 2]*	This work
RVM158	*rmIs133[unc-54p::40Q::YFP] X; unc-1(vlt10) X; vltEx158[myo-3p(o/e)); myo-2p::mCherry strain 1]*	This work
RVM159	*rmIs133[unc-54p::40Q::YFP] X; unc-1(vlt10) X; vltEx159[myo-3p(o/e)); myo-2p::mCherry strain 2]*	This work
RVM44	*rmIs133[unc-54p::40Q::YFP] X*; *unc-1(vlt10) X; vltEx44[rab-3p::unc-1(o/e)); myo-2p::mCherry strain 1]*	This work
RVM48	*rmIs133[unc-54p::40Q::YFP] X*; *unc-1(vlt10) X; vltEx48[rab-3p::unc-1(o/e)); myo-2p::mCherry strain 2]*	This work
RVM42	*rmIs133[unc-54p::40Q::YFP] X*; *vltEx42[rab-3p::AMP^r(RNAi)); myo-2p::mCherry strain 1]*	This work
RVM43	*rmIs133[unc-54p::40Q::YFP] X; vltEx43[rab-3p:: AMP^r(RNAi)); myo-2p::mCherry strain 2]*	This work
RVM71	*rmIs133[unc-54p::40Q::YFP] X*; *vltEx71[rab-3p::unc-1(RNAi)); myo-2p::mCherry strain 1]*	This work
RVM72	*rmIs133[unc-54p::40Q::YFP] X; vltEx72[rab-3p::unc-1(RNAi)); myo-2p::mCherry strain 2]*	This work
RVM205	*rmIs133[unc-54p::40Q::YFP] X; vltEx205[rab-3p(o/e)); myo-*	This work

	2p::mCherry strain 1]	
RVM206	*rmIs133[unc-54p::40Q::YFP] X; vltEx206[rab-3p(o/e)); myo-2p::mCherry strain 2]*	This work
RVM201	*rmIs133[unc-54p::40Q::YFP] X; vltEx201[rab-3p::unc-1(o/e)); myo-2p::mCherry strain 1]*	This work
RVM221	*rmIs133[unc-54p::40Q::YFP] X; vltEx221[rab-3p::unc-1(o/e)); myo-2p::mCherry strain 2]*	This work
RVM227	*rmIs133[unc-54p::40Q::YFP] X; vltEx227[rab-3p::unc-1(n494)(o/e)); myo-2p::mCherry strain 1]*	This work
RVM230	*rmIs133[unc-54p::40Q::YFP] X; vltEx230[rab-3p::unc-1(n494)(o/e)); myo-2p::mCherry strain 2]*	This work
RVM360	*rmIs133[unc-54p::40Q::YFP] X; vltEx360[trx-1p::AMP^{r}(RNAi)); myo-2p::mCherry strain 1]*	This work
RVM361	*rmIs133[unc-54p::40Q::YFP] X; vltEx361[trx-1p::AMP^{r}(RNAi)); myo-2p::mCherry strain 2]*	This work
RVM364	*rmIs133[unc-54p::40Q::YFP] X; vltEx364[trx-1p::unc-1(RNAi)); myo-2p::mCherry strain 1]*	This work
RVM365	*rmIs133[unc-54p::40Q::YFP] X; vltEx365[trx-1p::unc-1(RNAi)); myo-2p::mCherry strain 2]*	This work
RVM368	*rmIs133[unc-54p::40Q::YFP] X; vltEx368[oig-1p::AMP^{r}(RNAi); osm3p::AMP^{r}(RNAi); myo-2p::mCherry strain 1]*	This work
RVM369	*rmIs133[unc-54p::40Q::YFP] X; vltEx369[oig-1p::AMP^{r}(RNAi); osm-3p::AMP^{r}(RNAi); myo-2p::mCherry strain 2]*	This work
RVM355	*rmIs133[unc-54p::40Q::YFP] X; vltEx355[oig-1p::unc-1(RNAi); osm-3p::unc-1(RNAi); myo-2p::mCherry strain 1]*	This work
RVM356	*rmIs133[unc-54p::40Q::YFP] X; vltEx356[oig-1p::unc-1(RNAi); osm-3p::unc-1(RNAi); myo-2p::mCherry strain 2]*	This work
RVM362	*rmIs133[unc-54p::40Q::YFP] X; vltEx362[glr-1p::AMP^{r}(RNAi); gpa-9p::AMP^{r}(RNAi); myo-2p::mCherry strain 1]*	This work
RVM363	*rmIs133[unc-54p::40Q::YFP] X; vltEx363[glr-1p::AMP^{r}(RNAi); gpa-9p::AMP^{r}(RNAi); myo-2p::mCherry strain 2]*	This work
RVM366	*rmIs133[unc-54p::40Q::YFP] X; vltEx366[glr-1p::unc-1(RNAi); gpa-9p::unc-1(RNAi); myo-2p::mCherry strain 1]*	This work
RVM367	*rmIs133[unc-54p::40Q::YFP] X; vltEx367[glr-1p::unc-1(RNAi); gpa-9p::unc-1(RNAi); myo-2p::mCherry strain 2]*	This work
RVM386	*rmIs133[unc-54p::40Q::YFP] X; unc-1 (vlt10) X; vltEx386[oig-1p::unc-1(o/e); myo-2p::mCherry strain 1]*	This work
RVM387	*rmIs133[unc-54p::40Q::YFP] X; unc-1 (vlt10) X; vltEx387[oig-1p::unc-1(o/e); myo-2p::mCherry strain 2]*	This work
RVM384	*rmIs133[unc-54p::40Q::YFP] X; unc-1 (vlt10) X; vltEx384[osm-3p::unc-1(o/e); myo-2p::mCherry strain 1]*	This work
RVM385	*rmIs133[unc-54p::40Q::YFP] X; unc-1 (vlt10) X; vltEx385[osm-3p::unc-1(o/e); myo-2p::mCherry strain 2]*	This work
AM101	*rmIs110[F25B3.3-p::40Q::YFP]*	Brignull et al., 2006
RVM27	*rmIs110[F25B3.3-p::40Q::YFP]; unc-1(vlt10) X*	This work
RVM445	*rmIs110[F25B3.3-p::40Q::YFP]; unc-1(vlt10) X; vltEx445[osm-3p::unc-1(o/e); myo-2p::mCherry strain 1]*	This work
RVM446	*rmIs110[F25B3.3-p::40Q::YFP]; unc-1(vlt10) X; vltEx445[osm-3p::unc-1(o/e); myo-2p::mCherry strain 2]*	This work
CB75	*mec-2(e75) X*	Chalfie and Sulston, 1981

CB138	*unc-24(e138) IV*	Brenner, 1974
RVM208	*rmIs133[unc-54p::40Q::YFP] X*; *mec-2(e75) X*	This work
RVM192	*rmIs133[unc-54p::40Q::YFP] X*; *unc-24(e138) IV*	This work
CB5	*unc-7(e5) X*	Brenner, 1974
FH85	*unc-9(ec27) X*	Sedensky and Meneely, 1987
MR127	*inx-6(rr5) IV*	Li et al., 2003
RB1792	*inx-7(ok2319) IV*	The C. elegans deletion mutant consortium 2012
RVM209	*rmIs133[unc-54p::40Q::YFP] X*; *unc-7(e5) X*	This work
RVM175	*rmIs133[unc-54p::40Q::YFP] X*; *unc-9(ec27)* X	This work
RVM196	*rmIs133[unc-54p::40Q::YFP] X*; *inx-2(vlt22) X*	This work
RVM197	*rmIs133[unc-54p::40Q::YFP] X*; *inx-6(rr5) IV*	This work
RVM218	*rmIs133[unc-54p::40Q::YFP] X*; *inx-7(ok2319) IV*	This work
RVM340	*rmIs133[unc-54p::40Q::YFP] X; vltEx340[rab-3p::inx-6(RNAi)); myo-2p::mCherry strain 1]*	This work
RVM341	*rmIs133[unc-54p::40Q::YFP] X; vltEx341[rab-3p::inx-6(RNAi)); myo-2p::mCherry strain 2]*	This work
RVM333	*rmIs133[unc-54p::40Q::YFP] X; vltEx333[rab-3p::inx-2(RNAi)); myo-2p::mCherry strain 1]*	This work
RVM342	*rmIs133[unc-54p::40Q::YFP] X; vltEx342[rab-3p::inx-2(RNAi)); myo-2p::mCherry strain 2]*	This work
RVM335	*rmIs133[unc-54p::40Q::YFP] X; vltEx335[rab-3p::unc-7(RNAi)); myo-2p::mCherry strain 1]*	This work
RVM337	*rmIs133[unc-54p::40Q::YFP] X; vltEx337[rab-3p::unc-7(RNAi)); myo-2p::mCherry strain 2]*	This work
RVM346	*rmIs133[unc-54p::40Q::YFP] X; unc-7(e5) X*	This work
RVM337	*rmIs133[unc-54p::40Q::YFP] X; unc-1(vlt10) X; unc-7(e5) X*	This work
RVM349	*rmIs133[unc-54p::40Q::YFP] X; inx-2(vlt22) X*	This work
RVM348	*rmIs133[unc-54p::40Q::YFP] X; unc-1(vlt10) X; inx-2(vlt22) X*	This work
CW911	*ssu-1(fc73) V; unc-1(e580) X; rol-6(su1006)*	Carroll et al., 2006
RVM142	*rmIs133[unc-54p::40Q::YFP] X*; *ssu-1(fc73) V*	This work
RVM143	*rmIs133[unc-54p::40Q::YFP] X*; *unc-1(e580)* X	This work
RVM144	*rmIs133[unc-54p::40Q::YFP] X*; *ssu-1(fc73)* V; *unc-1(e580)* X	This work
RVM188	*rmIs133[unc-54p::40Q::YFP] X; unc-1 (vlt10) X; vltEx188[trx-1p::*AMP^{r}*(RNAi)); myo-2p::mCherry strain 1]*	This work
RVM189	*rmIs133[unc-54p::40Q::YFP] X; unc-1 (vlt10) X; vltEx189[trx-1p::*AMP^{r}*(RNAi)); myo-2p::mCherry strain 2]*	This work

RVM176	*rmIs133[unc-54p::40Q::YFP] X; unc-1(vlt10) X; vltEx176[trx-1p::ssu-1(RNAi)); myo-2p::mCherry strain 1]*	This work
RVM232	*rmIs133[unc-54p::40Q::YFP] X; unc-1(vlt10) X; vltEx232[trx-1p::ssu-1(RNAi)); myo-2p::mCherry strain 2]*	This work
VC382	*sul-2(gk187) V*	The C. elegans deletion mutant consortium 2012
RVM224	*rmIs133[unc-54p::40Q::YFP] X; sul-2(gk187) V*	This work
RVM225	*rmIs133[unc-54p::40Q::YFP] X; sul-2(gk187) V; unc-1(vlt10) X*	This work
RVM320	*rmIs133[unc-54p::40Q::YFP] X; daf-12(vlt19) X*	This work
AA292	*daf-36(k114) V*	Rottiers et al., 2006
RVM390	*rmIs133[unc-54p::40Q::YFP] X; daf-36(k114) V; vltEx390[daf-36p::daf-36; myo-2p::mCherry]*	This work
RVM288	*rmIs133[unc-54p::40Q::YFP] X; nhr-1(vlt15) X*	This work
RVM289	*rmIs133[unc-54p::40Q::YFP] X; unc-1(vlt10) X; nhr-1(vlt15) X*	This work
RVM293	*rmIs133[unc-54p::40Q::YFP] X; nhr-1(vlt16) X*	This work
RVM294	*rmIs133[unc-54p::40Q::YFP] X; unc-1(vlt10) X; nhr-1(vlt16) X*	This work
RVM290	*rmIs133[unc-54p::40Q::YFP] X; unc-1(vlt10) X; nhr-1(vlt15) X; vltEx290[unc-54p::nhr-1(o/e); myo-2p::mCherry strain 1]*	This work
RVM291	*rmIs133[unc-54p::40Q::YFP] X; unc-1(vlt10) X; nhr-1(vlt15) X; vltEx291[unc-54p::nhr-1(o/e); myo-2p::mCherry strain 2]*	This work
RVM449	*rmIs133[unc-54p::40Q::YFP] X; unc-1(vlt10) X; nhr-1(vlt15) X; vltEx290[rab-3p::nhr-1(o/e); myo-2p::mCherry strain 1]*	This work
RVM450	*rmIs133[unc-54p::40Q::YFP] X; unc-1(vlt10) X; nhr-1(vlt15) X; vltEx290[rab-3p::nhr-1(o/e); myo-2p::mCherry strain 2]*	This work
SJ4005	*zcIs4[hsp-4::GFP] V*	Calfon et al., 2002
RVM373	*zcIs4[hsp-4::GFP] V; unc-1(vlt10) X*	This work
RVM544	*pkIs2386[unc-54p::α-synuclein::YFP + unc-119(+)] IV; nhr-1(vlt16) X*	This work
RVM545	*pkIs2386[unc-54p::α-synuclein::YFP + unc-119(+)] IV; unc-1(vlt10) X; nhr-1(vlt16) X*	This work

[1]Strains are listed in order of appearance in the results.

Appendix Table S5. Primers used in this work.

PCR product	Primer sequence (5' – 3')	Strategy
attB1::rab-3p ::attB5r	Frw: **ggggacaagtttgtacaaaaaagcaggct**atcttcagatgggagcagtgg<br>Rev: **ggggacaacttttgtatacaaagttgt**catctgaaaatagggctactgtagat	Gateway MultiSite Pro
attB5::unc-1 ::attB2	Frw: gggacaactttgtatacaaaagttgaaatgtcaaacaaggaaagaac<br>Rev: ggggaccactttgtacaagaaagctgggtaaaggaaaatatgattattggtc	Gateway MultiSite Pro
unc-1(n494)	Frw: cttgatgaaagaactgaacat<br>Rev: atgttcagttctttcatcaag	Gateway MultiSite Pro
rab-3p	A: M13F<br>B: catctgaaaatagggctactgtagat	Tissue-specific RNAi PCR #1.1
unc-1 _sense/antisense overlapping: *rab-3p*	C1: **atctacagtagccctattttcagatg**atgtcaaacaaggaaagaacagag<br>D1: M13R<br>C2: **atctacagtagccctattttcagatg**ttattggtcttttttcataaatgctcc<br>D2: M13F<br>A*: atcttcagatgggagcagtgg<br>D1*: aaggaaaatatgattattggtc<br>D2*: aaatgtcaaacaaggaaagaac	Tissue-specific RNAi PCR #1.2 *PCR #2.1
AMP^r _sense/antisense overlapping: *rab-3p*	C1: **atctacagtagccctattttcagatg**gcatcttacggatggcatgacag<br>D1: acagagttcttgaagtggtggc<br>C2: **atctacagtagccctattttcagatg**cgtttggtatggcttcattcagc<br>D2: gcttacagacaagctgtgaccg<br>A*: atcttcagatgggagcagtgg<br>D1*: cgtttggtatggcttcattcagc<br>D2*: gcatcttacggatggcatgacag	Tissue-specific RNAi PCR #1.2 *PCR #2.1
inx-2 _sense/antisense overlapping: *rab-3p*	C1: **atctacagtagccctattttcagatg**gtaccacttgtccttcttttccaag<br>D1: gtcaaggaaatgtttcagaagaacc<br>C2: **atctacagtagccctattttcagatg**gtcaaggaaatgtttcagaagaacc<br>D2: gtaccacttgtccttcttttccaag<br>A*: atcttcagatgggagcagtgg<br>D1*: atttgcagaatgcaattgacaa<br>D2*: ctgccatgtttgtgctccctta	Tissue-specific RNAi PCR #1.2 *PCR #2.1
inx-6 _sense/antisense overlapping: *rab-3p*	C1: **atctacagtagccctattttcagatg**gtcaactttgtgaaccagtact<br>D1: aatgtatacagatggacgtcct<br>C2: **atctacagtagccctattttcagatg**aatgtatacagatggacgtcct<br>D2: gtcaactttgtgaaccagtact<br>A*: atcttcagatgggagcagtgg<br>D1*: tcgaaagctgctagccgagttttg<br>D2*: tgttccactcgaccagcaactagc	Tissue-specific RNAi PCR #1.2 *PCR #2.1
unc-7 _sense/antisense overlapping: *rab-3p*	C1: **atctacagtagccctattttcagatg**ctttgagcactcaaagaaaaactct<br>D1: tcagtctatcgtcccttgaccgtgt<br>C2: **atctacagtagccctattttcagatg**tcagtctatcgtcccttgaccgtgt<br>D2: ctttgagcactcaaagaaaaactct<br>A*: atcttcagatgggagcagtgg<br>D1*: tccgcatcccaaaattgcgtcgg<br>D2*: actataattcaaaagcaacctaatg	Tissue-specific RNAi PCR #1.2 *PCR #2.1
trx-1p	A: aaccaattgagttggcacttcg<br>B: aaccttggtgagagacatgatg	Tissue-specific RNAi PCR #1.1
ssu-1 _sense/antisense overlapping:	C1: **catcatgtctctcaccaaggtt**ccagagctctgtgtgcaatcgc<br>D1: catgtttcgcgtatttttctgc<br>C2: **catcatgtctctcaccaaggtt**ccttatacacagcattttcc<br>D2: cgtggcgggacccaaaatctc	Tissue-specific RNAi PCR #1.2 *PCR #2.1

trx-1p	A*: agaatggatacctgatcatt<br>D1*: ccttatacacagcattttcc<br>D2*: ccagagctctgtgtgcaatcgc	
AMP^{r} *_sense/antisense* overlapping: *trx-1p*	C1: **catcatgtctctcaccaaggtt**gcatcttacggatggcatgacag<br>D1: acagagttcttgaagtggtggc<br>C2: **catcatgtctctcaccaaggtt**cgtttggtatggcttcattcagc<br>D2: gcttacagacaagctgtgaccg<br>A*: agaatggatacctgatcatt<br>D1*: cgtttggtatggcttcattcagc<br>D2*: gcatcttacggatggcatgacag	Tissue-specific RNAi<br>PCR #1.2<br>*PCR #2.1
unc-1 _sense/antisense overlapping: *trx-1p*	C1: **catcatgtctctcaccaaggtt**atgtcaaacaaggaaagaacagag<br>D1: M13R<br>C2: **catcatgtctctcaccaaggtt**ttattggtctttttcataaatgctcc<br>D2: M13F<br>A*: agaatggatacctgatcatt<br>D1*: aaggaaaatatgattattggtc<br>D2*: aaatgtcaaacaaggaaagaac	Tissue-specific RNAi<br>PCR #1.2<br>*PCR #2.1
oig-1p	A: agagcaagcagtcagtgaaaatgt<br>B: agtcgaacgttttgcagaattatg	Tissue-specific RNAi PCR #1.1
osm-3p	A: gcttaaaatccggctaaaattca<br>B: tccgacgcatagctggaaattttg	Tissue-specific RNAi PCR #1.1
glr-1p	A: aacaagaaagtcgtagttgttac<br>B: tgtgaatgtgtcagattgggtgcc	Tissue-specific RNAi PCR #1.1
gpa-9p	A: gatggtcccggaaaacatcatcg<br>B: cccattgcatatttcattaaac	Tissue-specific RNAi PCR #1.1
unc-1 _sense overlapping: *oig-1p*	C1: **cataattctgcaaaacgttcgact**atgtcaaacaaggaaagaacagag<br>D1: M13R<br>A*: aacatgttttggagcatatttcgcg<br>D1*: aaggaaaatatgattattggtc	Tissue-specific RNAi<br>PCR #1.2<br>*PCR #2.1
unc-1 _antisense overlapping: *osm-3p*	C2: **caaaatttccagctatgcgtcgga**ttattggtctttttcataaatgctcc<br>D2: M13F<br>A*: aattaaatttgcctgaaaatccg<br>D2*: aaatgtcaaacaaggaaagaac	Tissue-specific RNAi<br>PCR #1.2<br>*PCR #2.1
unc-1 _sense overlapping: *glr-1p*	C1:**ggcacccaatctgacacattcaca**atgtcaaacaaggaaagaacagag<br>D1: M13R<br>A*: aataattataagagacgtgtag<br>D1*: aaggaaaatatgattattggtc	Tissue-specific RNAi<br>PCR #1.2<br>*PCR #2.1
unc-1 _antisense overlapping: *gpa-9p*	C2: **gtttaatgaaatatgcaatgggtt**attggtctttttcataaatgctcc<br>D2: M13F<br>A*: accgaatcaaaatatctgaat<br>D2*: aaatgtcaaacaaggaaagaac	Tissue-specific RNAi<br>PCR #1.2<br>*PCR #2.1
AMP^{r} *_sense* overlapping: *oig-1p*	C1: **cataattctgcaaaacgttcgact**gcatcttacggatggcatgacag<br>D1: acagagttcttgaagtggtggc<br>A*: aacatgttttggagcatatttcgcg<br>D1*: cgtttggtatggcttcattcagc	Tissue-specific RNAi<br>PCR #1.2<br>*PCR #2.1
AMP^{r} *_antisense* overlapping: *osm-3p*	C2: **caaaatttccagctatgcgtcgga**cgtttggtatggcttcattcagc<br>D2: gcttacagacaagctgtgaccg<br>A*: aattaaatttgcctgaaaatccg<br>D2*: gcatcttacggatggcatgacag	Tissue-specific RNAi<br>PCR #1.2<br>*PCR #2.1
AMP^{r} *_sense* overlapping:	C1: **ggcacccaatctgacacattcaca**gcatcttacggatggcatgacag<br>D1: acagagttcttgaagtggtggc<br>A*: aataattataagagacgtgtag	Tissue-specific RNAi<br>PCR #1.2

glr-1p	D1*: cgtttggtatggcttcattcagc	*PCR #2.1
AMP^r<br>*_antisense*<br>overlapping:<br>*gpa-9p*	C2: **gtttaatgaaatatgcaatggg**cgtttggtatggcttcattcagc<br>D2: gcttacagacaagctgtgaccg<br>A*: accgaatcaaaatatctgaat<br>D2*: gcatcttacggatggcatgacag	Tissue-specific<br>RNAi<br>PCR #1.2<br>*PCR #2.1
unc-1(cDNA)<br>*::unc-54t*<br>overlapping:<br>*oig-1p*	C1:**cataattctgcaaaacgttcgact**atgtcaaacaaggaaagaacagag<br>D1: aaacagttatgtttggtatattg<br>A*: aacatgttttggagcatatttcgcg<br>D1*: aatgtattctgtcatttaaggc	Tissue-specific<br>Rescue<br>PCR #1.2<br>*PCR #2.1
unc-1(cDNA)<br>*::unc-54t*<br>overlapping:<br>*osm-3p*	C1:**caaaatttccagctatgcgtcgga**atgtcaaacaaggaaagaacagag<br>D1: aaacagttatgtttggtatattg<br>A*: aattaaatttgcctgaaaatccg<br>D1*: aatgtattctgtcatttaaggc	Tissue-specific<br>Rescue<br>PCR #1.2<br>*PCR #2.1
sul-1	Frw: ttcagctctggaaatgctactac<br>Rev: tctttttgtacttcaagtagtgc	Feeding RNAi
sul-2	Frw: gctcacctagcagagctggattc<br>Rev: gtctctcgtgttgatcttgtgcc	Feeding RNAi
sul-3	Frw: ttacatacaccaaacctccggc<br>Rev: ttgaagtaacccgtttgtggtcc	Feeding RNAi
elo-2	Frw: tcatgcactggtatcatcatgcc<br>Rev: gcgcaaccaggaacagaatcag	Feeding RNAi
fat-6	Frw: cgtaagcatccacaagttaagg<br>Rev: ggtaattgaggaatcgtatggc	Feeding RNAi
skn-1	Frw: gatcgcgagagttgttccactgg<br>Rev: ggctttaataaggtttcgaccgag	Feeding RNAi

Appendix References

Altun ZF, Chen B, Wang Z-W & Hall DH (2009) High resolution map of *Caenorhabditis elegans* gap junction proteins. *Dev Dyn* 238: 1936–1950

Brenner S (1974) The genetics of Caenorhabditis elegans. *Genetics* 77: 71–94

Brignull HR, Moore FE, Tang SJ & Morimoto RI (2006) Polyglutamine Proteins at the Pathogenic Threshold Display Neuron-Specific Aggregation in a Pan-Neuronal Caenorhabditis elegans Model. *Journal of Neuroscience* 26: 7597–7606

Calfon M, Zeng H, Urano F, Till JH, Hubbard SR, Harding HP, Clark SG & Ron D (2002) IRE1 couples endoplasmic reticulum load to secretory capacity by processing the XBP-1 mRNA. *Nature* 415: 92–96

Carroll BT, Dubyak GR, Sedensky MM & Morgan PG (2006) Sulfated Signal from ASJ Sensory Neurons Modulates Stomatin-dependent Coordination in *Caenorhabditis elegans*. *J Biol Chem* 281: 35989–35996

Chalfie M & Sulston J (1981) Developmental genetics of the mechanosensory neurons of Caenorhabditis elegans. *Developmental Biology* 82: 358–370

Chernomor O, von Haeseler A & Minh BQ (2016) Terrace Aware Data Structure for Phylogenomic Inference from Supermatrices. *Syst Biol* 65: 997–1008

Cingolani P, Platts A, Wang LL, Coon M, Nguyen T, Wang L, Land SJ, Lu X & Ruden DM (2012) A program for annotating and predicting the effects of single nucleotide polymorphisms, SnpEff: SNPs in the genome of Drosophila melanogaster strain w1118; iso-2; iso-3. *Fly (Austin)* 6: 80–92

Garrison E & Marth G (2012) Haplotype-based variant detection from short-read sequencing. *arXiv:12073907 [q-bio]*

van Ham TJ, Thijssen KL, Breitling R, Hofstra RMW, Plasterk RHA & Nollen EAA (2008) C. elegans Model Identifies Genetic Modifiers of α-Synuclein Inclusion Formation During Aging. *PLoS Genet* 4: e1000027

Lapatsina L, Brand J, Poole K, Daumke O & Lewin GR (2012) Stomatin-domain proteins. *European Journal of Cell Biology* 91: 240–245

Li H (2011) Improving SNP discovery by base alignment quality. *Bioinformatics* 27: 1157–1158

Li H (2013) Aligning sequence reads, clone sequences and assembly contigs with BWA-MEM. *arXiv:13033997 [q-bio]*

Li S, Dent JA & Roy R (2003) Regulation of Intermuscular Electrical Coupling by the *Caenorhabditis elegans* Innexin *inx-6*. *MBoC* 14: 2630–2644

Link CD (1995) Expression of human beta-amyloid peptide in transgenic Caenorhabditis elegans. *Proceedings of the National Academy of Sciences* 92: 9368–9372

Morley JF, Brignull HR, Weyers JJ & Morimoto RI (2002) The threshold for polyglutamine-expansion protein aggregation and cellular toxicity is dynamic and influenced by aging in Caenorhabditis elegans. *Proceedings of the National Academy of Sciences* 99: 10417–10422

Nguyen L-T, Schmidt HA, von Haeseler A & Minh BQ (2015) IQ-TREE: A Fast and Effective Stochastic Algorithm for Estimating Maximum-Likelihood Phylogenies. *Molecular Biology and Evolution* 32: 268–274

Park EC & Horvitz HR (1986) Mutations with dominant effects on the behavior and morphology of the nematode Caenorhabditis elegans. *Genetics* 113: 821–852

Rajaram S, Sedensky MM & Morgan PG (1998) unc-1: A stomatin homologue controls sensitivity to volatile anesthetics in Caenorhabditis elegans. *Proceedings of the National Academy of Sciences* 95:

8761–8766
Rottiers V, Motola DL, Gerisch B, Cummins CL, Nishiwaki K, Mangelsdorf DJ & Antebi A (2006) Hormonal Control of C. elegans Dauer Formation and Life Span by a Rieske-like Oxygenase. *Developmental Cell* 10: 473–482
Sedensky M & Meneely P (1987) Genetic analysis of halothane sensitivity in Caenorhabditis elegans. *Science* 236: 952–954
The C. elegans Deletion Mutant Consortium (2012) Large-Scale Screening for Targeted Knockouts in the *Caenorhabditis elegans* Genome. *G3* 2: 1415–1425
