# Supplementary material for: Changes in lipid metabolism driven by steroid signalling modulate proteostasis in C. elegans
Source: EMBO Rep. 2023 Apr 27;24(6):e55556. doi: 10.15252/embr.202255556 (PMC10240203; doi:10.15252/embr.202255556)
Supplement: Supplementary file 11 [file EMBR-24-e55556-s001.pdf]

EMBO reports

Changes in lipid metabolism driven by steroid signalling modulate proteostasis in *C. elegans*

Ana Gómez-Escribano, Carlos Mora-Martinez, Marta Roca, Denise Walker, Joaquín Panadero, Maria Sequedo, Ratni Saini, Hans-Joachim Knölker, Jose Blanca, Juan Burguera, Agustin Lahoz, Joaquin Cañizares, José Millán, Nick Burton, William Schafer and Rafael Vazquez-Manrique
DOI: 10.15252/embr.202255556

Corresponding author(s): Rafael Vazquez-Manrique (rafael_vazquez@iislafe.es)

Review Timeline:

Submission Date:	8th Jun 22
Editorial Decision:	12th Jul 22
Revision Received:	9th Dec 22
Editorial Decision:	13th Jan 23
Revision Received:	2nd Mar 23
Accepted:	22nd Mar 23

Editor: Martina Rembold/Esther Schnapp

Transaction Report:

(Note: With the exception of the correction of typographical or spelling errors that could be a source of ambiguity, letters and reports are not edited. Depending on transfer agreements, referee reports obtained elsewhere may or may not be included in this compilation. Referee reports are anonymous unless the Referee chooses to sign their reports.)

1st Editorial Decision 12th Jul 2022

Dear Dr. Vazquez-Manrique,

Thank you for the submission of your manuscript to EMBO reports. I have taken over its handling as Martina is currently not in the office. We have now received the full set of referee reports that is pasted below.

As you will see, the referees acknowledge that the findings are potentially interesting. However, they also raise technical, experimental and conceptual concerns and note that the language and writing of the manuscript must be improved. I think all concerns are valid and should be addressed. Please let me know in case you disagree, and we can discuss the revisions further, also in a video chat if you like. This is a borderline case, and we can only move forward with your study here if at least 2 referees will support the publication of your revised manuscript.

Having said that, I would like to invite you to revise your manuscript with the understanding that the referee concerns must be fully addressed and their suggestions taken on board. Please address all referee concerns in a complete point-by-point response. Acceptance of the manuscript will depend on a positive outcome of a second round of review. It is EMBO reports policy to allow a single round of major revision only and acceptance or rejection of the manuscript will therefore depend on the completeness of your responses included in the next, final version of the manuscript.

We realize that it is difficult to revise to a specific deadline. In the interest of protecting the conceptual advance provided by the work, we recommend a revision within 3 months (12th Oct 2022). Please discuss the revision progress ahead of this time with the editor if you require more time to complete the revisions.

IMPORTANT NOTE: we perform an initial quality control of all revised manuscripts before re-review. Your manuscript will FAIL this control and the handling will be DELAYED if the following APPLIES:
1) A data availability section providing access to data deposited in public databases is missing. If you have not deposited any data, please add a sentence to the data availability section that explains that.
2) Your manuscript contains statistics and error bars based on n=2. Please use scatter blots in these cases. No statistics should be calculated if n=2.

When submitting your revised manuscript, please carefully review the instructions that follow below. Failure to include requested items will delay the evaluation of your revision.

1) a .docx formatted version of the manuscript text (including legends for main figures, EV figures and tables). Please make sure that the changes are highlighted to be clearly visible.

2) individual production quality figure files as .eps, .tif, .jpg (one file per figure). See https://wol-prod-cdn.literatumonline.com/pb-assets/embo-site/EMBOPress_Figure_Guidelines_061115-1561436025777.pdf for more info on how to prepare your figures.

3) We replaced Supplementary Information with Expanded View (EV) Figures and Tables that are collapsible/expandable online. A maximum of 5 EV Figures can be typeset. EV Figures should be cited as 'Figure EV1, Figure EV2" etc... in the text and their respective legends should be included in the main text after the legends of regular figures.

- For the figures that you do NOT wish to display as Expanded View figures, they should be bundled together with their legends in a single PDF file called *Appendix*, which should start with a short Table of Content. Appendix figures should be referred to in the main text as: "Appendix Figure S1, Appendix Figure S2" etc. See detailed instructions regarding expanded view here: <https://www.embopress.org/page/journal/14693178/authorguide#expandedview>

- Additional Tables/Datasets should be labeled and referred to as Table EV1, Dataset EV1, etc. Legends have to be provided in a separate tab in case of .xls files. Alternatively, the legend can be supplied as a separate text file (README) and zipped together with the Table/Dataset file.

4) a .docx formatted letter INCLUDING the reviewers' reports and your detailed point-by-point responses to their comments. As part of the EMBO Press transparent editorial process, the point-by-point response is part of the Review Process File (RPF), which will be published alongside your paper.

5) a complete author checklist, which you can download from our author guidelines <https://www.embopress.org/page/journal/14693178/authorguide>. Please insert information in the checklist that is also reflected in the manuscript. The completed author checklist will also be part of the RPF.

6) Please note that all corresponding authors are required to supply an ORCID ID for their name upon submission of a revised manuscript (<https://orcid.org/>). Please find instructions on how to link your ORCID ID to your account in our manuscript tracking system in our Author guidelines
<https://www.embopress.org/page/journal/14693178/authorguide#authorshipguidelines>

7) Before submitting your revision, primary datasets produced in this study need to be deposited in an appropriate public database (see https://www.embopress.org/page/journal/14693178/authorguide#datadeposition). Please remember to provide a reviewer password if the datasets are not yet public. The accession numbers and database should be listed in a formal "Data Availability" section placed after Materials & Method (see also https://www.embopress.org/page/journal/14693178/authorguide#datadeposition). Please note that the Data Availability Section is restricted to new primary data that are part of this study. * Note - All links should resolve to a page where the data can be accessed. *
If your study has not produced novel datasets, please mention this fact in the Data Availability Section.

8) We would also encourage you to include the source data for figure panels that show essential data. Numerical data should be provided as individual .xls or .csv files (including a tab describing the data). For blots or microscopy, uncropped images should be submitted (using a zip archive if multiple images need to be supplied for one panel). Additional information on source data and instruction on how to label the files are available at <https://www.embopress.org/page/journal/14693178/authorguide#sourcedata>.

9) Our journal also encourages inclusion of *data citations in the reference list* to directly cite datasets that were re-used and obtained from public databases. Data citations in the article text are distinct from normal bibliographical citations and should directly link to the database records from which the data can be accessed. In the main text, data citations are formatted as follows: "Data ref: Smith et al, 2001" or "Data ref: NCBI Sequence Read Archive PRJNA342805, 2017". In the Reference list, data citations must be labeled with "[DATASET]". A data reference must provide the database name, accession number/identifiers and a resolvable link to the landing page from which the data can be accessed at the end of the reference. Further instructions are available at https://www.embopress.org/page/journal/14693178/authorguide#referencesformat

10) Regarding data quantification (see Figure Legends: https://www.embopress.org/page/journal/14693178/authorguide#figureformat)

The following points must be specified in each figure legend:

- the name of the statistical test used to generate error bars and P values,

- the number (n) of independent experiments (please specify technical or biological replicates) underlying each data point,

- the nature of the bars and error bars (s.d., s.e.m.),

- If the data are obtained from n {less than or equal to} 2, use scatter blots showing the individual data points.

Discussion of statistical methodology can be reported in the materials and methods section, but figure legends should contain a basic description of n, P and the test applied.

- Please also include scale bars in all microscopy images.

11) The journal requires a statement specifying whether or not authors have competing interests (defined as all potential or actual interests that could be perceived to influence the presentation or interpretation of an article). In case of competing interests, this must be specified in your disclosure statement. Further information: https://www.embopress.org/competing-interests

We would also welcome the submission of cover suggestions, or motifs to be used by our Graphics Illustrator in designing a cover.

As part of the EMBO publication's Transparent Editorial Process, EMBO reports publishes online a Review Process File (RPF) to accompany accepted manuscripts. This File will be published in conjunction with your paper and will include the referee reports, your point-by-point response and all pertinent correspondence relating to the manuscript.

You are able to opt out of this by letting the editorial office know (emboreports@embo.org). If you do opt out, the Review Process File link will point to the following statement: "No Review Process File is available with this article, as the authors have chosen not to make the review process public in this case."

I look forward to seeing a revised form of your manuscript when it is ready. Please use this link to submit your revision: https://embor.msubmit.net/cgi-bin/main.plex

Yours sincerely,

Esther Schnapp, PhD

Senior Editor
EMBO reports

Referee #1:

Proteostasis and its regulation is an exciting topic, even more so when one considers extracellular signaling systems that allow one tissue to regulate proteostasis in another. The C. elegans genetic model organism has been unprecedented in its power to address this topic experimentally. Here, Gomez-Escribano et al use C. elegans genetics and genomics to examine how nervous system signaling and nuclear hormone receptors regulate protein aggregation in muscle through their modulation of lipid metabolism.

The authors conducted a screen in C. elegans for genes involved in polyQ-YFP aggregation. They identified a mutation in the stomatin unc-1 that resultsin increased polyQ aggregation in muscle, and they convincingly show through multiple approaches that UNC-1 normally acts in neurons (IL2 specifically) to inhibit this aggregation. A previously publication showed a genetic interaction between unc-1 and ssu-1, the lone C. elegans alcohol sulfotransferase. SSU-1 is only expressed in the neuron ASJ, which is connected by gap junctions to IL2; thus, the authors test and show that SSU-1 acts in ASJ to promote aggregation in an otherwise unc-1 mutant background. Hypothesizing that SSU-1 acts by sulfonating the dafachronic acid steroid hormones, the authors use CRISPR-Cas9 editing to make a ligand binding domain mutation in DAF-12, the nuclear hormone receptor through which dafachronic acid acts. These mutants show a slight elevation in polyQ aggregate number, suggesting that DAF-12 inhibits aggregation and is thus not the hormone substrate of SSU-1. Instead, the authors generate and analyze two new alleles of the nuclear hormone receptor NHR-1, showing that nhr-1 loss of function mutations suppress aggregation in unc-1 mutants. The authors perform a transcriptomics analysis to show that fatty acid synthesis is decreased in unc-1 mutants due to overactive NHR-1 activity. They conclude that NHR-1 and DAF-12 regulate proteostasis and protein aggregation by regulating lipid metabolism.

The topic is interesting. The paper is rich in experimental data. However, the manuscript at times reads more like a loosely connected set of experimental observations than a coherent whole. There are numerous inconsistencies between the experimental data and the conclusions drawn by the authors (as well as with their final model). The field at this stage is looking for more mechanistic insight, as well as a compelling explanation for how changes in lipid metabolism result in changes in proteostasis, and this current manuscript does not really do much to address these issues. It is not ready for publication, although it could be if multiple items were addressed:

The writing lacks clarity, particularly in the abstract. There are so many punctuation problems and grammatical error throughout the draft as to preclude listing all of them here, making it nearly impossible to review the manuscript. It should not be our job as reviewers to list all of the grammatical errors and areas where the writing lacks clarity. The authors should properly proof the manuscript and, if needed, they should obtain assistance from an English language editing service. The poor writing quality by itself would be grounds to merit rejection were it not dramatically improved upon future resubmission.

There is a large body of literature, most of which involves the use of C. elegans, describing non-autonomous mechanisms that regulate proteostasis, yet the authors only refer to a single example (the Morimoto HSF paper from 2013). There is no shortage of reviews on this subject, and the authors should cite some of the more recent ones. Moreover, there is extensive literature relating lipids to proteostasis. They should introduce the topic with a more thorough list of examples that reflects the state of the field. Papers they should consider mentioning (listed by PMID number): 28723566, 31408455, 33470040, 33296657, 26196144.

The figure 1A cartoon is not effective. The diagram lacks an F1 generation. The F2 phenotype are no displayed in any Mendelian fashion. It is unclear from the cartoon how the screen was conducted (e.g., clonal? Non-clonal?).

Figure 2F. I think the statistical comparison being made in this panel is the transgenic rescues (osm-3p::unc-1) to wild type. This really should be made to the non-transgenic unc-1 mutants if the authors are testing whether the transgene is rescuing in those two lines.

Figure S2. The labeling in this figure is unclear, as more than one black bar has the same label (e.g., the horizontal line indicating myo-3p::unc-1). How are the black bars different from one another? Different independent lines? If so, then this should be indicated.

On page 6, the authors state, "using the pan-neuronal promoter of the rab-3 gene, unc-1(vlt10) animals showed a substantial reduction of polyQ aggregation in all stages, with no effect in the expression of the transgene (Supplementary Fig. 2)," but there is no such data in Figure S2, which only describes the muscle-driven constructs.

On page 8, the authors describe results from calcium measurement experiments without providing some explanation of how they are doing this (i.e., without introducing their specific GCAMP reporter). Also, it is not clear what is meant by the concluding sentence, "It seems more likely that these reflect distinct, localized functions." It is not clear that changes in ASJ calcium activity in response to temperature fluctuation have any role with regard to muscle proteostasis. Is there any reason to think that the

temperature fluctuations sensed by ASJ are translated into differential neurotransmitter signaling and/or changes in muscle proteostasis? Perhaps this section (Figure 2G-K) should be removed from the manuscript, as it really does not add anything to the story or help prove their model.

If ASJ has a normal tonic role in promoting aggregation, why does a mutation in ssu-1, which is the sole alcohol sulfotransferase, not have an effect on aggregation by itself (i.e., it can only suppress the phenotype of an unc-1 mutant, but doesn't have a phenotype by itself)?

If SSU-1 sulfonates a hormone that promotes polyQ aggregation in unc-1 mutants, then why does loss of SUL-2 and SUL-3, enzymes which one would think normally desulfonate hormones, not enhance aggregation? Instead, the authors observed that sul-2 and sul-3 loss of function suppress aggregation in unc-1 mutants - the same phenotype as loss of ssu-1. This is a bit counterintuitive.

On page 10, the authors state, "Analysis of both mutant alleles showed that disruption of nhr-1, in unc-1 worms, produces a substantial reduction of the inclusion bodies observed in unc-1 mutants (Fig. 5A), indicating that NHR-1 acts downstream of SSU-1 to regulate protein homeostasis." This shows that nhr-1 is epistatic to unc-1, like how ssu-1 is epistatic to unc-1. It does not say anything about epistasis between nhr-1 and ssu-1, and thus doesn't tell us the order of these two genes with respect to one another.

Figure S6, panel A. Presumably the heat map is based on row Z-scores? That should be clearly indicated.

Figure 5F. It is great that oleic acid supplementation rescues unc-1 mutants with regard to their polyQ aggregation phenotype, but why does rescue not occur in nhr-1 mutants? The author's model is that UNC-1 inhibits NHR-1, which normally inhibits fatty acid synthesis, including the expression of FAT-6 and FAT-7, which synthesize oleic acid. If oleic acid acts downstream of NHR-1, then supplemental rescue with oleic acid should work regardless of whether nhr-1 is mutated.

Figure 5H. It is a bit puzzling why unc-1 mutants should show higher Oil Red O staining, indicative of higher levels of stored lipids (mostly triglycerides). Indeed, the author's own lipidomic analysis shows that triglycerides and fatty acids are down in unc-1 mutants relative to wild type, a result that better matches their transcriptomic analysis. So why do the authors observe more Oil Red O staining in unc-1 mutants if the triglyceride levels in these mutants are reduced?

Figure S7, panel A. There are too many slices in the pie charts to tell which lipid class correlates to which slice of the pie. Consider listing as a table instead, with percentages. Also, are the differences in panel B statistically significant? If so, this should be indicated. If not, this data should be removed.

Results from figure 5F (oleic acid supplementation rescue of unc-1) would suggest that oleic acid levels are reduced in unc-1 mutants. It was not clear from the figures whether this is the case. That is, the authors only show oleic acid (18:1) normalized to stearic acid (18:0), which might mask a reduction in absolute oleic acid levels in unc-1, as stearic acid levels are likely to also be reduced in these mutants.

Referee #2:

The manuscript starts off with a mutagenesis screen to identify factors that regulate protein aggregation. They identified one gene (unc-1), that upon mutation leads to an increase in protein aggregation (non-specifically, as polyQ proteins, a-synuclein and Abeta show an increased aggregation). The authors performed rescue experiments and cell-specific RNAi and identified that unc-1 in the IL2 neurons sets off the signalling cascade. IL2 neurons are communicating with ASJ neurons and this communication is disrupted in unc-1 mutants (via a sulfotransferase that modifies NHRs). This then in turn leads to an impaired NHR signalling and a dysregulation in the lipid metabolism. This signal cascade ultimately ends in DAF-12 that is a known regulator of protein homeostasis.

This manuscript is complex as a lot of very interesting findings are presented yet not in a coherent manner. The authors should carefully dissect correlation from causation. Nevertheless, the connection of the lipid metabolism and protein homeostasis are very interesting and the authors provide here a lot of molecular details on the regulation.
However, there are a number of major and minor comments that should be addressed to improve the manuscript.

Major comments:

1. The manuscripts starts off with a screen. Yet they only mention one hit. First, how many hits were identified in total and second, was unc-1 identified before in any previously published screens using the nematode model e.g. Silva et al., Plos Genetics 2011; van Ham et al., Plos Genetics 2008; Brehme et al., Cell Reports 2014

2. Please provide an overview of all alleles of unc-1 and provide experimental data which allele is expressed in specific

cells/tissues. If not, then please omit statements such as on page 7 n494 disrupts function in the nervous system.

3. As increased protein levels could lead to a higher aggregation propensity, please check the protein levels of the model substrate Q40-YFP in all genetic backgrounds by western blot. The western blot depicted in EV1A needs to be properly labelled. Which protein band is Q40-YFP? Why is there a double band?

4. What is actually sensed by the IL2 neurons that activates unc-1?
In other words: how does the wt animal sense and maintains the protein homeostasis via NHR and lipid metabolism?

5. Some experiments are not explained well. E.g. figure 1G: This Calcium sensor is not introduced in the text.

Minor comments:

1. There are numerous spelling mistakes throughout the manuscript. Please improve the writing e.g. statements such as on page 9: "Some authors..." should be replaced by "Some studies have shown" etc

2. Figure assignment is not always correct e.g. p6 bottom section there it is stated that EV2 shows expression of the transgene.

3. Please show original data of EV2B

4. Figure 2E: the labelling of the middle four columns is incomplete. I assume that two adjacent columns refer to neuronal and muscle Q40? But if so - state it and second, the controls need to be shown for both tissues.

5. The fonts of EV6B+C are far too small.

6. I can only assume that EV5D shows qRT-PCR data. There is no information on that. Why were these selected genes analysed? The authors performed transcriptomic analyses. Did those genes not show up? Please comment on that.

Referee #3:

The manuscript "Changes in lipid metabolism driven by steroid signaling modulate proteostasis in C. elegans" by Gómez-Escribano et al., explores the connection between protein homeostasis (proteostasis) in somatic tissues, measured by Q40 foci formation in muscle, and a novel mutation in neuronal stomatin-like gene unc-1, identified in an EMS mutagenesis screen. The authors first show that unc-1(vlt10) as well as other unc-1 alleles (and other stomatin-like and innexins) induced Q40 aggregation (as well as α-synuclein and β-amyloid) in muscle (and in neurons) when disrupted. They then use tissue-specific rescue of unc-1 expression to determine the affected cells - IL12, in which unc-1 is functional. Based on previous genetic interactions between unc-1 and the sulfotransferase ssu-1 and the fact the ssu-1 is expressed ASJ that has an electrical synapsis with IL12, the authors suggest that the unc-1 dependent disruption of IL12 function results in increased ssu-1 mediated signaling (an excess of a sulphated signal). Ssu-1 involvement was then examined based on reduced Q40 aggregation in muscle in unc-1;ssu-1 double mutant or in ssu-1 ASJ RNAi background. Furthermore, disrupting sulfatase enzymatic activity by KD of sul-2 and sul-3 partially reduced Q40 aggregation in unc-1 mutant background. Because sulfotransferases can modulate steroid hormones, the authors next examine the effect of disrupting daf-12 or daf-36, showing increased Q40 aggregation. Assuming that daf-12 is not the target of ASJ steroid signal (given the synthetic phenotype in the triple mutant), the authors then focused on nhr-1, which was previously connected to ssu-1. Analyzing Q40 aggregation they suggest that nhr-1 acts in body wall muscles, downstream of unc-1. The authors suggest that nhr-1 deletion restores proteostasis in unc-1 (and also in daf-12) mutant, reducing stress gene activation. Finally, they suggest that nhr-1 regulates fat metabolism and that this, in turn, regulates proteostasis. Specifically, they claim that nhr-1 deletion reverses the expression patterns of fat genes in unc-1 mutants back to a wild-type like pattern. Because nhr-1 reduced daf-12;Q40 aggregation and rescued daf-12;unc-1;Q40 lethality they suggest that nhr-1 and daf-12 act antagonistically on muscle proteostasis.

The casual links between unc-1 (disruption of ASJ), SSU-1, nhr-1 (nhr-1-daf-12), and muscle proteostasis are not really tangible. For example, ssu-1 involvement is assumed based on previous genetic interaction with unc-1 (which they showed is not specific as other stomatin-like and innexins also affected Q40), and the link is supported by the fact that the number of aggregates in ssu-1;unc-1 is similar to ssu-1 alone (rescue). ssu-1 has specific phenotypes and it was shown to modulate the expression of genes under osmotic and starvation stresses. If unc-1 increases ssu-1 signaling this can be tested directly. Likewise, the claim that nhr-1 impact on proteostasis is mediated by fatty acids is supported mainly by similarity (weak at best and not quantified) between WT and unc-1;nhr-1 double mutant on the gene expression profile of metabolism of fatty acids genes. Moreover, there is a single measure of proteostasis - Q40 aggregation, already shown to be modulated independently from Q40 toxicity. While the authors have produced a large body of results and were careful in most of the analyses they present, I find the conclusions overreaching. Finally, there are many studies examining cell non-autonomous proteostasis regulation including links to metabolism, the connection is not novel. I agree with the authors that the opposing function of daf-12

and nhr-1 on protein aggregation is novel and understanding this genetic interaction will be of interest.

We thank the referees and the editor for their positive and constructive comments. We believe that we have addressed all their concerns. Please see our responses in blue.

Referee #1:

Proteostasis and its regulation is an exciting topic, even more so when one considers extracellular signaling systems that allow one tissue to regulate proteostasis in another. The C. elegans genetic model organism has been unprecedented in its power to address this topic experimentally. Here, Gomez-Escribano et al use C. elegans genetics and genomics to examine how nervous system signaling and nuclear hormone receptors regulate protein aggregation in muscle through their modulation of lipid metabolism.

The authors conducted a screen in C. elegans for genes involved in polyQ-YFP aggregation. They identified a mutation in the stomatin unc-1 that results in increased polyQ aggregation in muscle, and they convincingly show through multiple approaches that UNC-1 normally acts in neurons (IL2 specifically) to inhibit this aggregation. A previously publication showed a genetic interaction between unc-1 and ssu-1, the lone C. elegans alcohol sulfotransferase. SSU-1 is only expressed in the neuron ASJ, which is connected by gap junctions to IL2; thus, the authors test and show that SSU-1 acts in ASJ to promote aggregation in an otherwise unc-1 mutant background. Hypothesizing that SSU-1 acts by sulfonating the dafachronic acid steroid hormones, the authors use CRISPR-Cas9 editing to make a ligand binding domain mutation in DAF-12, the nuclear hormone receptor through which dafachronic acid acts. These mutants show a slight elevation in polyQ aggregate number, suggesting that DAF-12 inhibits aggregation and is thus not the hormone substrate of SSU-1. Instead, the authors generate and analyze two new alleles of the nuclear hormone receptor NHR-1, showing that nhr-1 loss of function mutations suppress aggregation in unc-1 mutants. The authors perform a transcriptomics analysis to show that fatty acid synthesis is decreased in unc-1 mutants due to overactive NHR-1 activity. They conclude that NHR-1 and DAF-12 regulate proteostasis and protein aggregation by regulating lipid metabolism.

The topic is interesting. The paper is rich in experimental data. However, the manuscript at times reads more like a loosely connected set of experimental observations than a coherent whole. There are numerous inconsistencies between the experimental data and the conclusions drawn by the authors (as well as with their final model). The field at this stage is looking for more mechanistic insight, as well as a compelling explanation for how changes in lipid metabolism result in changes in proteostasis, and this current manuscript does not really do much to

address these issues. It is not ready for publication, although it could be if multiple items were addressed:

We thank the referee for his/her positive comments and have worked to address the highlighted issues.

1) The writing lacks clarity, particularly in the abstract. There are so many punctuation problems and grammatical error throughout the draft as to preclude listing all of them here, making it nearly impossible to review the manuscript. It should not be our job as reviewers to list all of the grammatical errors and areas where the writing lacks clarity. The authors should properly proof the manuscript and, if needed, they should obtain assistance from an English language editing service. The poor writing quality by itself would be grounds to merit rejection were it not dramatically improved upon future resubmission.

We have hired a scientific editor, Kaycie Butler (from ButlerSciComm) to assist in our storytelling and improve the language of the paper.

2) There is a large body of literature, most of which involves the use of C. elegans, describing non-autonomous mechanisms that regulate proteostasis, yet the authors only refer to a single example (the Morimoto HSF paper from 2013). There is no shortage of reviews on this subject, and the authors should cite some of the more recent ones. Moreover, there is extensive literature relating lipids to proteostasis. They should introduce the topic with a more thorough list of examples that reflects the state of the field. Papers they should consider mentioning (listed by PMID number): 28723566, 31408455, 33470040, 33296657, 26196144.

We agree with the referee that we should cite more reviews of lipid regulation and proteostasis in the introduction. We have also included more specific examples relevant to our paper in the text. As these reviews are already fairly comprehensive and to keep length in mind, though, we have refrained from re-reviewing the field, instead highlighting those examples most relevant to our work and that paint a better picture of the current state of the field. Therefore, we have included all references that referee point out in two paragraphs, which appear as follows:

Proteostasis can also be modulated by the metabolic status of the organism and has been shown to be connected to lipid metabolism (Webster et al, 2017; Steinbaugh et al, 2015; Joshi et al, 2021; Lee et al, 2019; Higuchi-Sanabria et al, 2020). For example, Webster C.M et al., showed that inhibiting the genes that encode amino-acyl tRNA synthetases increases fat production and activates the proteasomal AMPK network to extend survival under starvation conditions (Webster et al, 2017). In addition, the lipid homeostasis regulator MDT-15 is required for maintaining HSF–1-depedent proteostasis at low temperatures in 35Q::YFP worms

(Lee et al, 2019). Lipids can also modulate proteostasis via lipid depletion through serotonergic, but not dopaminergic, neuronal XBP-1s (Higuchi-Sanabria et al, 2020). Moreover, XBP-1s remodel lipid metabolism by non-cell autonomous signalling, which decreases triglyceride and increases oleic acid levels (Imanikia et al, 2019).

In terms of lipid metabolism, Joshi *et al.* claim that sensory neurons secrete biogenic amines to modulate lipid signalling, which in turn activates UPR pathways to maintain proteostasis (Joshi *et al*, 2021). This work is evidence that sensory neurons can release signalling molecules to regulate gene expression.

3) The figure 1A cartoon is not effective. The diagram lacks an F1 generation. The F2 phenotype are no displayed in any Mendelian fashion. It is unclear from the cartoon how the screen was conducted (e.g., clonal? Non-clonal?).

We agree with the referee that Figure 1A is not clear and does not contribute relevant information. Therefore, we have decided to remove it from Figure 1. Moreover, to address the reviewer's concerns about the clarity surrounding the screen, we have ensured that the Appendix Supplementary Methods section is updated to include all of the relevant information (please see the section on "isolation of *unc-1(vlt10)* mutation" of the Appendix).

4) Figure 2F. I think the statistical comparison being made in this panel is the transgenic rescues (osm-3p::unc-1) to wild type. This really should be made to the non-transgenic unc-1 mutants if the authors are testing whether the transgene is rescuing in those two lines.

We agree with the referee that we should show the comparison to the non-transgenic *unc-1* mutants, so we updated it. We have thus updated Figure 2F to do the same. Moreover, we applied the suggestion from the referee to Figure 2E, so we have modified everything accordingly.

5) Figure S2. The labeling in this figure is unclear, as more than one black bar has the same label (e.g., the horizontal line indicating myo-3p::unc-1). How are the black bars different from one another? Different independent lines? If so, then this should be indicated.

We apologise for not being clear here. The duplicated bars correspond to independent transgenic lines carrying the array; we usually do more than one stable line to avoid artefacts. We have rephrased this to make it clearer. We have modified Figure S2A and S2B belong to Appendix Figure S2. In the Figure, we have included the stable line termini as "Line 1 and Line 2" to differentiate between the independent, stable transgenic lines. In addition, we have included detailed information about it in the Figure legend.

6) On page 6, the authors state, "using the pan-neuronal promoter of the rab-3 gene, unc-1(vlt10) animals showed a substantial reduction of polyQ aggregation in all stages, with no effect in the expression of the transgene (Supplementary Fig. 2)," but there is no such data in Figure S2, which only describes the muscle-driven constructs.

We apologise for this mistake. We meant to refer to Figure 2A. We thank the referee for pointing it out, and we have corrected the mistake.

7) On page 8, the authors describe results from calcium measurement experiments without providing some explanation of how they are doing this (i.e., without introducing their specific GCAMP reporter). Also, it is not clear what is meant by the concluding sentence, "It seems more likely that these reflect distinct, localized functions." It is not clear that changes in ASJ calcium activity in response to temperature fluctuation have any role with regard to muscle proteostasis. Is there any reason to think that the temperature fluctuations sensed by ASJ are translated into differential neurotransmitter signaling and/or changes in muscle proteostasis? Perhaps this section (Figure 2G-K) should be removed from the manuscript, as it really does not add anything to the story or help prove their model.

Apologies for not adequately explaining our motivation for these experiments or the conclusions that we drew from them. We have edited the description in the Results to explain our hypothesis: if *unc-1* acts in IL2 and a mutation in *unc-1* increases *ssu-1*-mediated signalling from ASJ, we speculated that by disrupting gap junctions, the *unc-1* mutation prevents current shunting and thus increases ASJ excitability. Temperature sensing is a known function of ASJ, for which calcium imaging had previously been used to characterise the responses to changes in temperature. We therefore used it as a readout for overall ASJ activity. We agree that the results do not help to prove our model; the *unc-1* mutation did not appear to alter general excitability of ASJ; however, this was a pertinent question to ask. We have therefore retained the data but moved it to the Appendix (please revised Appendix Figure S4).

8) If ASJ has a normal tonic role in promoting aggregation, why does a mutation in ssu-1, which is the sole alcohol sulfotransferase, not have an effect on aggregation by itself (i.e., it can only suppress the phenotype of an unc-1 mutant, but doesn't have a phenotype by itself)?

A lack of function of a given gene does not necessarily need to show a phenotype, but it may induce epistasis over a mutation on another gene, which is the case for *ssu-1*. In Carrol et al., JBC 2006, the authors show data suggesting that *ssu-1* does not produce a phenotype by itself, yet modifies three phenotypes of *unc-1* mutants: volatile anaesthetic sensitivity, uncoordination, and dauer formation. We have

added a phrase to point this out in the manuscript: This is consistent with previous evidence that *ssu-1* mutations modified several *unc-1* phenotypes but not phenotypes in a wild type background (Carrol et al., JBC, 2006). We hope that this explanation, and the change in the manuscript, addresses the referee's issue.

9) If SSU-1 sulfonates a hormone that promotes polyQ aggregation in unc-1 mutants, then why does loss of SUL-2 and SUL-3, enzymes which one would think normally desulfonate hormones, not enhance aggregation? Instead, the authors observed that sul-2 and sul-3 loss of function suppress aggregation in unc-1 mutants - the same phenotype as loss of ssu-1. This is a bit counterintuitive.

The addition of a sulfate moiety in the hormone is required for it to exit the cell and/or travel and reach organs and tissues. Then, once it arrives, the hormone must be desulfonated to bind NRs. Thus, SSU-1 and SUL-2/SUL-3 are not acting antagonistically. Rather, they are acting at different stages of the signalling process. Mutants would thus be expected to have the same phenotype.

We have edited the text to explain this more carefully in the "Arylsulfatase activity modulates the aggregation dynamics of polyQs" section as the following: Sulfonated molecules are more water-soluble, and thus may more easily diffuse into organs and tissues than non-sulfonated signals. However, to bind nuclear receptors, the sulfonated molecules need to lose their sulphate moiety.

To support a clear visual explanation about the sulfation/desulfonation process by *ssu-1* (sulfotransferase) and *sul-2/sul-3* (aryl-sulfatases), we have included here the following diagram, for the editor and the reviewers to see:

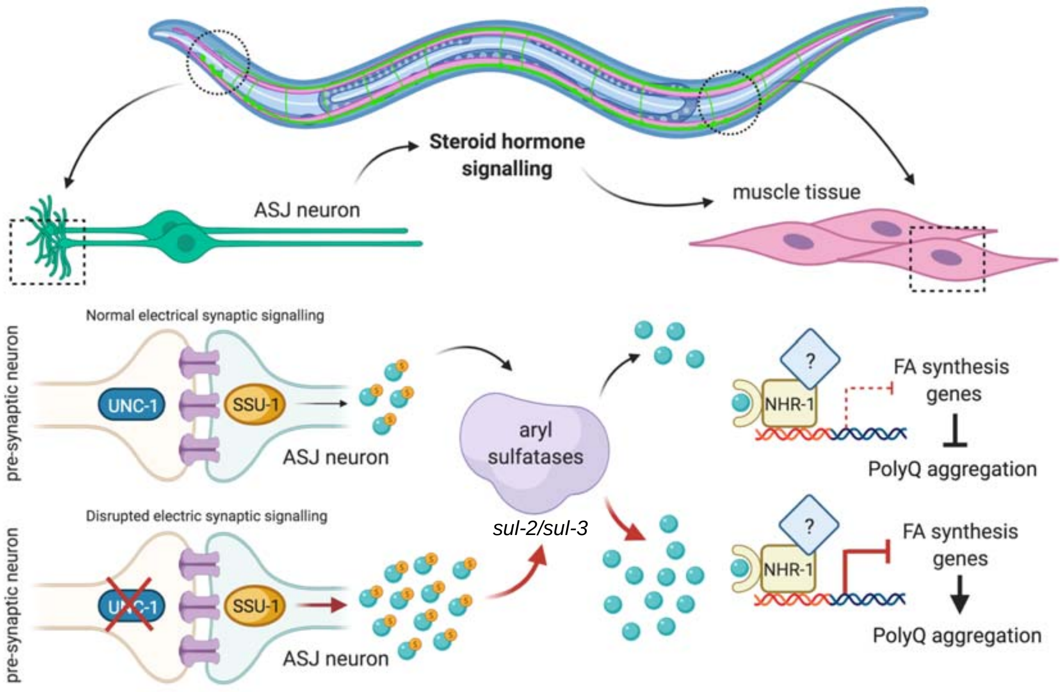

10) On page 10, the authors state, "Analysis of both mutant alleles showed that disruption of nhr-1, in unc-1 worms, produces a substantial reduction of the inclusion bodies observed in unc-1 mutants (Fig. 5A), indicating that NHR-1 acts downstream of SSU-1 to regulate protein homeostasis." This shows that nhr-1 is epistatic to unc-1, like how ssu-1 is epistatic to unc-1. It does not say anything about epistasis between nhr-1 and ssu-1, and thus doesn't tell us the order of these two genes with respect to one another.

We somewhat agree with the referee. We haven't shown a genetic interaction between *nhr-1* and *ssu-1*, but we never state that there is epistasis of *nhr-1* over *ssu-1*. In fact, as ablating both molecules reduces polyQs, it is hard to see how one mutant may be epistatic over the other. Hence, we believe that any experiments designed to clarify this point would not produce relevant information about the position of the molecules in the pathway.

In any case, SSU-1 is the enzyme producing the signal, and NHR-1 is the receptor of the signal. Of course, we cannot rule out that NHR-1 may induce a physiological response that affects SSU-1. However, putting the molecules in the following order: UNC-1 -> SSU-1 -> NHR-1 provides, in our opinion, the most parsimonious explanation for our data.

11) Figure S6, panel A. Presumably the heat map is based on row Z-scores? That should be clearly indicated.

Yes, the heat map is based on row z-scores in Figure S6 (from now Appendix Figure S7). We have included this information in the Appendix Figure S7 legend to make it clear. There, we point out that we used z-scores to identify changes between conditions independent of whether the gene expression mean was high or low.

12) Figure 5F. It is great that oleic acid supplementation rescues unc-1 mutants with regard to their polyQ aggregation phenotype, but why does rescue not occur in nhr-1 mutants? The author's model is that UNC-1 inhibits NHR-1, which normally inhibits fatty acid synthesis, including the expression of FAT-6 and FAT-7, which synthesize oleic acid. If oleic acid acts downstream of NHR-1, then supplemental rescue with oleic acid should work regardless of whether nhr-1 is mutated.

We agree with the referee that this may look like an unexpected result, though it is completely compatible with the nature of nuclear receptors. Although we do not have a complete explanation, the literature shows that nuclear receptors are extremely complex in their regulation. In this regard, it has been shown that some nuclear receptors may be retro-activated from products of the genes that they regulate. In this regard, Bagamasbad and Denver (2011 Gen Comp Endocrinol) wrote a nice review about the topic. Hence, we could speculate that NHR-1, or any potential nuclear receptor partner of NHR-1, requires oleic acid to work and also modulates oleic acid synthesis. We added two phrases about this issue in the discussion.

13) Figure 5H. It is a bit puzzling why unc-1 mutants should show higher Oil Red O staining, indicative of higher levels of stored lipids (mostly triglycerides). Indeed, the author's own lipidomic analysis shows that triglycerides and fatty acids are down in unc-1 mutants relative to wild type, a result that better matches their transcriptomic analysis. So why do the authors observe more Oil Red O staining in unc-1 mutants if the triglyceride levels in these mutants are reduced?

We agree that these two figures may look like contradictory results, but that is likely because we didn’t explain this well. As the reviewer commented, Oil Red Staining (ORO) does not exclusively stain triglycerides, but also stains other neutral lipids (mono-, di-glycerides, cholesterol esters, etc). Thus, this dye shows the sum of different classes of lipids, including but not limited to triglycerides. Conversely, lipidomics shows the semiquantitative measurement and specific determination of triglycerides. We have further clarified this issue in the text, and we have stated that the conclusion of the analysis is that *unc-1* mutants accumulate more neutral lipid (Figure 5G–H), whereas the proportion of triglycerides is lower compared to wild type (please see the Expanded View 5A).

14) Figure S7, panel A. There are too many slices in the pie charts to tell which lipid

class correlates to which slice of the pie. Consider listing as a table instead, with percentages. Also, are the differences in panel B statistically significant? If so, this should be indicated. If not, this data should be removed.

We agree with the referee about Figure S7A (now Expanded View 5A) not being clear. Now, we have included a list of all detected lipid classes, with percentages in each strain (*unc-1* and wild type animals), and we have improved the coloured pie chart to avoid confusion.

In addition, when doing this work, we detected a technical mistake from our side in the pie chart—we didn't show the correct total lipid distribution in *unc-1* mutants. We have corrected it, and now the major abundance of lipids corresponds to phosphatidylcholines (which usually is the most abundant lipid class).

We have included a modified legend for the Figure in Expanded View 5 to show more details.

15) Results from figure 5F (oleic acid supplementation rescue of unc-1) would suggest that oleic acid levels are reduced in unc-1 mutants. It was not clear from the figures whether this is the case. That is, the authors only show oleic acid (18:1) normalized to stearic acid (18:0), which might mask a reduction in absolute oleic acid levels in unc-1, as stearic acid levels are likely to also be reduced in these mutants.

As the referee points out, the results from Figure 5F suggest that *unc-1* has lower oleic acid levels. This agrees with the transcriptomic analysis, which shows that the enzymes that produce oleic acid are downregulated (Figure 5C; see *fat-6* and *fat-7*). In addition, we have validated the transcriptomic data *in vivo* using RNAi to silence one of these genes (Figure 5E), which had no effect on *unc-1* because, we reasoned, they are already at a low expression level. Next, we decided to quantify specific fatty acids in *unc-1* animals using lipidomics.

In our previous figure, Figure S7B–F (now removed) was designed to directly correlate gene expression and enzymatic activity using the products of each step of the synthetic pathway. But as it is confusing, we removed it. Hence, we followed the referee's suggestion regarding the manner of showing how fatty acids, like oleic acid, could mask differences, as our visual was not suitable for reflecting it.

Now, we have eliminated Figure S7B–F from the main text and have added the analysis of absolute fatty acid levels, including oleic acid, for *unc-1* and wild type animals (Expanded View 5B–H). We have included only the same fatty acids that were in the old figure. The new graphs contain information about the absolute levels of the following free fatty acids: (B) 14:0 (malonyl-CoA); (C) 16:0 (stearic); (D) 16:1 (palmitic); (E) 18:0 (stearic); (F) 18:1(oleic); (G) 18:3(gamma-linoleic); (H) 20:3 (dihomo-gamma-linoleic). We show statistically significant differences between

unc-1 vs wild type animals for malonyl-CoA (upper in *unc-1*) and stearic (lower in *unc-1*) free fatty acids, and we can see strong tendencies, though not statistically significant, for gamma-linoleic acid (lower in *unc-1*) (please see Figures EV5B, 5E and 5G). In addition, we observe a lower abundance with weak tendencies for palmitic, palmitoleic, oleic and dihomo-gamma-linoleic in *unc-1* mutants (please see Figures EV5C, 5D, 5F and 5H). We have extensively rephrased this section of the results.

We would like to clarify that the lipidomic data only shows free fatty acids, so we cannot see if there is fatty acid accumulation in complex lipids or organelles. In addition, this approach contains few samples, which explains (at least in part) our noted trends.

Referee #2:

The manuscript starts off with a mutagenesis screen to identify factors that regulate protein aggregation. They identified one gene (unc-1), that upon mutation leads to an increase in protein aggregation (non-specifically, as polyQ proteins, a-synuclein and Abeta show an increased aggregation). The authors performed rescue experiments and cell-specific RNAi and identified that unc-1 in the IL2 neurons sets off the signalling cascade. IL2 neurons are communicating with ASJ neurons and this communication is disrupted in unc-1 mutants (via a sulfotransferase that modifies NHRs). This then in turn leads to an impaired NHR signalling and a dysregulation in the lipid metabolism. This signal cascade ultimately ends in DAF-12 that is a known regulator of protein homeostasis.

This manuscript is complex as a lot of very interesting findings are presented yet not in a coherent manner. The authors should carefully dissect correlation from causation. Nevertheless, the connection of the lipid metabolism and protein homeostasis are very interesting and the authors provide here a lot of molecular details on the regulation.

We thank the referee very much for the positive comments and the helpful suggestions for improvement.

However, there are a number of major and minor comments that should be addressed to improve the manuscript.

Major comments:

1. The manuscripts starts off with a screen. Yet they only mention one hit. First, how many hits were identified in total and second, was unc-1 identified before in any previously published screens using the nematode model e.g. Silva et al., Plos Genetics 2011; van Ham et al., Plos Genetics 2008; Brehme et al., Cell Reports 2014

We obtained two mutants, but after NGS analysis we found that was the same mutation in *unc-1*, so they were probably not an independent mutation, though we cannot rule out this hypothesis. We have rephrased the text with the following text: “Though several mutants were identified from this screen, only one matched our criteria—that carrying the *vlt10* allele in *unc-1* (Appendix Figure S1A–B), which substantially enhanced aggregation without affecting 40Q transgene expression (Figure 1A–B and Appendix Figure S1C-D)”.

We have checked all the papers suggested by referee. We have verified that *unc-1* alleles haven’t been identified in these works, as explained in the following:

(1) Silva et al., Plos Genetics 2011: Screening is done to identify suppressors, and *unc-1(vlt10)* is an enhancer. In this work, they did not see *unc-1* alleles as a potential hits. They have identified only the unc gene, *unc-15*.

(2) Van Ham et al., Plos Genetics 2008: We have checked it to confirm that *unc-1* is not a hit.

(3) Brehme et al., Cell Reports 2014: We have confirmed that *unc-1* is not a hit from this work because it is not a chaperone gene. The authors only identified chaperone genes (we can cite textually from paper: “This functional screen identified 18 genes, corresponding to ten ATP-dependent chaperones, HSC70 (*hsp-1*), HSP90 (*daf-21*), and eight subunits of the CCT/TRiC chaperonin complex; the co-chaperones, HSP40 (*dnj-12*) and CDC37 (*cdc-37*); and the TPR-domain protein STI1 that upon knockdown significantly enhanced Ab42 proteotoxicity”).

2. Please provide an overview of all alleles of unc-1 and provide experimental data which allele is expressed in specific cells/tissues. If not, then please omit statements such as on page 7 n494 disrupts function in the nervous system.

We apologise because we may not have explained this experiment well. We are certainly not suggesting that this mutant allele changes the cells/tissues in which the gene is expressed. In this experiment, we exogenously introduced a cDNA containing the mutation found in the *n494* allele, which confers a dominant negative function on UNC-1. This is just a tool to disrupt UNC-1 function in certain cells and tissues. In this case, we used a neuronal promoter to disrupt UNC-1 function only in neurons, while its function is normal in the rest of tissues where the *unc-1* gene is expressed. We have rephrased this bit of the text to further explain the experiment, and we hope that now the reader will be able to better understand the technique. At the end of the page 7 we have included the following explanation: we disrupted *unc-1* function across the nervous system by overexpressing a cDNA containing the *n494* dominant allele of *unc-1*, again using the *rab-3* promoter to drive expression in all neurons.

3. As increased protein levels could lead to a higher aggregation propensity, please check the protein levels of the model substrate Q40-YFP in all genetic backgrounds by western blot. The western blot depicted in EV1A needs to be properly labelled. Which protein band is Q40-YFP? Why is there a double band?

Following the suggestion of the referee, we have analysed protein levels in all genetic backgrounds using western blots with an anti-polyQ antibody, and we were able to verify that the increase in body inclusions is not related to altered transgene expression levels (Appendix Figure S1C–D).

We have modified the labelling of Figure EV1A (now Appendix Figure S1C) to mark the 40Q::YFP bands. The double band identified by the anti-polyQ antibody most likely reflects proteolytic cleavage of 40Q::YFP. Many authors also show this pattern by western blot (Nollen et al., Proc Natl Acad Sci, 2004 and Muñoz-Lobato et al., Antioxidants and Redox Signaling, 2014). We have explained this topic in the appendix Figure S1 legend to avoid confusion.

In addition, we have checked polyQ protein levels in all genetic backgrounds related directly with UNC-1 > SSU-1 > NHR-1, by western blot. The New Appendix Figure S1C-D includes representative images and quantification data from western blot analysis.

4. What is actually sensed by the IL2 neurons that activates unc-1? In other words: how does the wt animal sense and maintains the protein homeostasis via NHR and lipid metabolism?

This is a very interesting question, and we certainly do not know the answer at the moment. It will be interesting future work to investigate the upstream signals that modulate this pathway, which is sufficient work to constitute its own separate paper. We added some comments regarding this question in the Discussion section: "What is sensing and signalling IL2 we don't know at this point, but we think that it would be interesting future work. Also, it will be interesting to investigate the role of the molecules that conduct the crosstalk between IL2 and ASJ, and the regulation of the electrical synapse over the control of metabolism".

5. Some experiments are not explained well. E.g. figure 1G: This Calcium sensor is not introduced in the text.

Apologies for our poor explanations. We have edited the description of the calcium imaging experiments in the Results section to better explain our motivation for performing them and our interpretation of the results. We also better introduce the calcium sensor. We have included the following text to explain calcium sensor:

“Since an increase in ASJ general excitability might be expected to increase overall cellular activity and the response to a temperature stimulus, we examined whether these temperature responses were intact in *unc-1(vlt-10)* animals by expressing the genetically encoded ratiometric calcium indicator Cameleon in ASJ”. We have included our interpretation of the results with the following text: “Thus, the effect of *unc-1(vlt10)* on signalling from ASJ does not stem from a general increase in ASJ excitability, so cannot simply be explained by a disruption in the gap junctions that eliminates current shunting to IL2”.

Regarding to Figure 1G (now Appendix Figure S4A) we have expanded details as the following way: (A) Temperature stimuli were delivered using perfusion and calcium responses in ASJ were measured as described previously (Walker and Schafer, eLife, 2020), using the ratiometric calcium indicator YC3.60 expressed under the ASJ-specific *trx-1* promoter (Fenk and de Bono, 2015). Moreover, we have included more information in the main Methods section (you can see in bold letter the new phrases):

Calcium imaging in ASJ was carried out as described previously (Kerr *et al*, 2000; Walker & Schafer, 2020). **The ratiometric calcium indicator YC3.60 (Cameleon) was expressed in ASJ using the *trx-1* promoter (Fenk and de Bono, 2015).** Briefly, animals were glued onto 2% agarose pads and temperature stimuli were administered using perfusion of CTX buffer (25 mM KPO4 pH6, 1 mM CaCl2, 1 mM MgSO4). Images were recorded at 5 Hz using **a Dualview beam splitter (Optical Insights) to simultaneously record two wavelengths, on** an iXon EM camera with IQ1.9 capture software (Andor Technology). **Fluorescence was quantified** and analysed in Matlab (Mathworks) using SpikeFinder and NeuronTracker, **custom** analysis scripts written by Ithai Rabinowitch (Rabinowitch *et al*, 2013).

Minor comments:

1. There are numerous spelling mistakes throughout the manuscript. Please improve the writing e.g. statements such as on page 9: "Some authors..." should be replaced by "Some studies have shown" etc

We sent the paper to a scientific editor (Kaycie Butler) to fix these issues. We hope that now the paper reads better and everything makes more sense.

2. Figure assignment is not always correct e.g. p6 bottom section there it is stated that EV2 shows expression of the transgene.

We are sorry for this mistake, and we have corrected it. We now reference Figure 2 and not figure EV2.

3. Please show original data of EV2B

We have added the qRT-PCR raw data from EV2B (from now Appendix Figure S2B) in the Appendix material denoted as Appendix Table S1.

4. Figure 2E: the labelling of the middle four columns is incomplete. I assume that two adjacent columns refer to neuronal and muscle Q40? But if so - state it and second, the controls need to be shown for both tissues.

Except wild type worms (WT, white bar) all the strains in Figure 2E are *unc-1* mutants [*unc-1(vlt10)*] (black bars). All the strains for WT and *unc-1* expressed the 40Q::YFP transgene. Then, we produced two independent transgenic stable lines carrying the *osm-3p::unc-1* transgene and two independent transgenic stable lines carrying the *oig-1p::unc-1* transgene. We have rephrased the figure legend to make it clearer in the paper.

Now, we have divided Figure 2E–F in the figure legend to clarify this statement and have indicated the following in the text: (**E**) The average number of polyQ inclusion bodies in muscle cells after *unc-1(cDNA)* was restored in *unc-1(vlt10)* mutants expressing *40Q::YFP* in muscle cells using either *osm-3* or *oig-1* promoters specific for IL2 neurons. We produced two independent transgenic stable lines (line 1 and line 2), carrying the *osm-3p::unc-1*(cDNA) transgene, and two independent transgenic stable lines (line 1 and line 2) carrying *oig-1p::unc-1*(cDNA) transgene. Comparison is done from all strains to *unc-1* mutants. (**F**) The average number of ventral nerve cord aggregates in *unc-1(vlt10)* mutants that express *40Q::YFP* in the whole nervous system and that had *unc-1(cDNA)* restored using the same extrachromosomal arrays as in (E) that drive *osm-3p::unc-1*(cDNA) expression in IL2 neurons. Comparison is done from all strains to *unc-1* mutants.

5. The fonts of EV6B+C are far too small.

We have made them bigger (EV6B+C; from now Appendix Figure S7B–C), as suggested by the referee.

6. I can only assume that EV5D shows qRT-PCR data. There is no information on that. Why were these selected genes analysed? The authors performed transcriptomic analyses. Did those genes not show up? Please comment on that.

Effectively, Figure EV5D (now Expanded View 1D) shows qRT-PCR data for several genes related to the unfolded protein response (UPR). We agree with the referee that we have not explained much in the Figure legend, but to remedy this, we have included more information about that in part of the Appendix material titled: *unc-1*

animals show ER stress that is rescued by the ablation of *nhr-1*.

We analysed these genes because UPR pathways regulate cellular proteostasis (Leitman, Nat Commun, 2013 and Shacham and Lederkremer, Fron. Mol. Biosci, 2019), and so we thought that the enhanced polyQ aggregation in *unc-1* animals might activate UPRs. Therefore, we selected genes of three main pathways (reticulum, mitochondria and cytosol) based on Taylor RC, Nature Rev Mol Cel Bio, 2014; to evaluate it. To be clearer, we have included a better and wide explanation into de Appendix Supplementary text ("*unc-1* animals show stress in the ER which is rescued by ablation of *nhr-1*").

Referee #3:

The manuscript "Changes in lipid metabolism driven by steroid signaling modulate proteostasis in C. elegans" by Gómez-Escribano et al., explores the connection between protein homeostasis (proteostasis) in somatic tissues, measured by Q40 foci formation in muscle, and a novel mutation in neuronal stomatin-like gene unc-1, identified in an EMS mutagenesis screen. The authors first show that unc-1(vlt10) as well as other unc-1 alleles (and other stomatin-like and innexins) induced Q40 aggregation (as well as α-synuclein and β-amyloid) in muscle (and in neurons) when disrupted. They then use tissue-specific rescue of unc-1 expression to determine the affected cells - IL12, in which unc-1 is functional. Based on previous genetic interactions between unc-1 and the sulfotransferase ssu-1 and the fact the ssu-1 is expressed ASJ that has an electrical synapsis with IL12, the authors suggest that the unc-1 dependent disruption of IL12 function results in increased ssu-1 mediated signaling (an excess of a sulphated signal). Ssu-1 involvement was then examined based on reduced Q40 aggregation in muscle in unc-1;ssu-1 double mutant or in ssu-1 ASJ RNAi background. Furthermore, disrupting sulfatase enzymatic activity by KD of sul-2 and sul-3 partially reduced Q40 aggregation in unc-1 mutant background. Because sulfotransferases can modulate steroid hormones, the authors next examine the effect of disrupting daf-12 or daf-36, showing increased Q40 aggregation. Assuming that daf-12 is not the target of ASJ steroid signal (given the synthetic phenotype in the triple mutant), the authors then focused on nhr-1, which was previously connected to ssu-1. Analyzing Q40 aggregation they suggest that nhr-1 acts in body wall muscles, downstream of unc-1. The authors suggest that nhr-1 deletion restores proteostasis in unc-1 (and also in daf-12) mutant, reducing stress gene activation. Finally, they suggest that nhr-1 regulates fat metabolism and that this, in turn, regulates proteostasis. Specifically, they claim that nhr-1 deletion reverses the expression patterns of fat genes in unc-1 mutants back to a wild-type like pattern. Because nhr-1 reduced daf-12;Q40

aggregation and rescued daf-12;unc-1;Q40 lethality they suggest that nhr-1 and daf-12 act antagonistically on muscle proteostasis.

1) The casual links between unc-1 (disruption of ASJ), SSU-1, nhr-1 (nhr-1-daf-12), and muscle proteostasis are not really tangible. For example, ssu-1 involvement is assumed based on previous genetic interaction with unc-1 (which they showed is not specific as other stomatin-like and innexins also affected Q40), and the link is supported by the fact that the number of aggregates in ssu-1; unc-1 is similar to ssu-1 alone (rescue).

Yes, the referee is right. We looked to the literature for potential interactors, which is how we got to *ssu-1*. It is true that other stomatins and innexins affect aggregation, but not all of them, so the interaction is restricted to some molecules. In any case, the point of the paper is not to show whether *unc-1* is essential for protein homeostasis, which it is, but to show that neurohormonal signalling controls proteostasis and that altered function of ASJ impacts in this process.

And yes, we show in our paper that *ssu-1* is epistatic over *unc-1* (i.e. the number of aggregates in *ssu-1; unc-1* is the same as the ones in *ssu-1*), which demonstrates a genetic interaction and shows how the information flows in this pathway. This is the same genetic principle behind the way that DAF-2/insulin-like receptor function was linked to DAF-16/FOXO (Reviewed by Murphy and Hu 2013 Wormbook). The rest of genetic dissection follows similar rules.

2) ssu-1 has specific phenotypes and it was shown to modulate the expression of genes under osmotic and starvation stresses. If unc-1 increases ssu-1 signaling this can be tested directly.

We thank the referee for this suggestion, which we believe is a great idea. We have taken advantage of our RNA-seq data to analyse the expression of potential genes. Considering the Burton N. et al. paper, we have identified several genes regulated by *ssu-1* in osmotic stress conditions (Burton et al., Nat Commun, 2018). We have analysed the relative expression levels of these genes in our RNA-seq analysis in 40Q and 40Q; *unc-1(vlt10)* worms. The RNA-seq data shows that upregulated genes in *ssu-1* mutants (Burton et al., Nat Commun, 2018) are downregulated in *unc-1* mutants (Expanded View 4). Inversely, genes without expression or that are downregulated in *ssu-1(fc73)* have upper expression levels in *unc-1* mutants (Expanded View 4). These results support that *unc-1* ablation induces gene expression changes in *ssu-1*-regulated genes, so it is evidence that *unc-1* could affect *ssu-1* signalling.

3) Likewise, the claim that nhr-1 impact on proteostasis is mediated by fatty acids is supported mainly by similarity (weak at best and not quantified) between WT and

unc-1; nhr-1 double mutant on the gene expression profile of metabolism of fatty acids genes. Moreover, there is a single measure of proteostasis - Q40 aggregation, already shown to be modulated independently from Q40 toxicity.

We thank the referee for the suggestion. We have investigated whether the NHR-1 nuclear receptor is able to modulate aggregation of other prone-to-aggregation molecules, like α-synuclein. This is now included in the paper as an Expanded View 2 (EV2). Therefore, we can conclude that NHR-1 modulates proteostasis due to modulate polyQ and α-synuclein protein aggregation.

4) While the authors have produced a large body of results and were careful in most of the analyses they present, I find the conclusions overreaching.

We agree that our initial wording was too ambitious and have extensively toned down many statements, so hopefully there is no more overreaching in our paper.

5) Finally, there are many studies examining cell non-autonomous proteostasis regulation including links to metabolism, the connection is not novel. I agree with the authors that the opposing function of daf-12 and nhr-1 on protein aggregation is novel and understanding this genetic interaction will be of interest.

We totally agree with the referee that understanding this genetic interaction is of great interest, but with the due respect, this story should be told in its own future paper. As per the novelty of this paper, it is the first evidence that nuclear receptors modulate polyQ aggregation by remodelling lipid metabolism.

Dear Dr. Vazquez-Manrique

Thank you for the submission of your revised manuscript to EMBO reports. We have now received the full set of referee reports that is copied below.

As you will see, all referees acknowledge that the revision has strengthened the data and conclusions. However, they also have a number of remaining concerns regarding text, figures and statistical analysis that need to be addressed. Referee 2 notes that the signal sensed by IL2 neurons has not been identified. Please acknowledge this limitation of the data in the manuscript and discuss potential scenarios. Please also provide a point-by-point response.

From the editorial side, there are also a few things that we need before we can proceed with the official acceptance of your study.

1) Your study will be published in our "Reports" section. In order to make this possible, please combine the Results and Discussion section and please keep an eye on our character limit of approx. 27,000 characters (including spaces but excluding materials & methods and references)

2) Please reduce the number of keywords to 5.

3) Please update the 'Conflict of interest' paragraph to our new 'Disclosure and competing interests statement'. For more information see
https://www.embopress.org/page/journal/14693178/authorguide#conflictsofinterest

4) Regarding the Author Contributions, we now use CRediT to specify the contributions of each author in the journal submission system. CRediT replaces the author contribution section, which therefore needs to be removed from the manuscript text. You can use the free text box in our system if you wish to provide more detailed descriptions. See also guide to authors
https://www.embopress.org/page/journal/14693178/authorguide#authorshipguidelines

- Please add the following funding information in our online submission system. Currently, it is only listed in the manuscript text: European Regional Development Fund, RPVM (VII Convocatoria Ayudas a la Investigación MHER, 2019, cofinanced by Colegio Oficial de Farmacéuticos de Sevilla and Fundación Cajasol) and CIVP19S8119

5) Please add callouts for Figure EV2A and EV2B, Figure EV4A and EV4B in the text.

6) The source data for main figures needs to be reorganized: It should be one file/folder per figure (zipped).

7) Author checklist: please complete the information in the third column, i.e., in which section the information has been provided, and the information on experimental animals.
- In the Author checklist you refer to a preprint on bioRxiv in the section 'Study design'. This section is meant to refer to the pre-registration of a study design or protocol. Can you please clarify?

8) Please note that all methods need to be part of the main manuscript. Therefore, please move the Appendix Supplementary Methods over to the main manuscript methods section.

9) Can the Appendix Supplementary Text be incorporated into the main manuscript text as well?

10) Appendix Fig. S1A and S7 have rather low resolution, which must be improved.

11) Information missing in the Appendix figure legends:
- General: Please use the prefix 'Data information:" if you describe something that refers to more than one panel in the legend, e.g., if you describe the plotted data, bars, error bars, number of samples shown in several panels in the figure
- S1D: define the bars and error bars.
- S2 has no panel C in contrast to what the legend says.
- S4: provide information on number of samples (technical, biological replicates), the statistical test used and define ** and ns
- S6A, B: number of samples, define bars and error bars.

12) I attach to this email a related manuscript file with comments by our data editors. Please address all comments and upload a revised file with tracked changes with your final manuscript submission. Please be more specific and clear in the abstract.

13) Finally, EMBO reports papers are accompanied online by A) a short (1-2 sentences) summary of the findings and their significance, B) 2-3 bullet points highlighting key results and C) a synopsis image that is 550x300-600 pixels large (width x height) in PNG for JPG format. You can either show a model or key data in the synopsis image. Please note that the size is

rather small and that text needs to be readable at the final size. Please send us this information along with the revised manuscript.

We look forward to seeing a final version of your manuscript as soon as possible.

With kind regards,

Martina Rembold, PhD
Senior Editor
EMBO reports

****

Referee #1:

Overall, the authors in Gomez-Escribano have addressed my concerns. There are still some loose ends to the story (e.g., it's still not clear what the exact role of unc-1 mutations is on ASJ excitability), but clearly they are "over the bar" regarding quantity of data and interesting observations and conclusions. That said, I do feel they need to fix a few things before publication:

1. The font sizes on their graphs vary from panel to panel in the same figures. For example, Figure 2 panel A has larger font labeling of the Y-axis compared to panel B-F. Figure 3 panel A has larger font than panel B. Figure 5 is the worst in this regard, as the Y-axis labeling is so small on panels E, F, and H as to make it nearly unreadable without magnifying the image. This is a simple fix.

2. Appendix Figure S7 appears to be a low-resolution raster image that has become pixelated in the conversion process to the PDF. As is, it is unreadable. The authors should instead use the original vector image generated by whatever graphing software they used, as such an image will not become pixelated in the final PDF.

3. The penultimate sentence of the abstract states that the author's findings are "suggesting for the first time that fat metabolism changes, controlled by neurohormonal signalling, contribute to protein homeostasis." As the authors acknowledge in their rebuttal letter and in the newest version of the manuscript, there have been previous published examples showing a connection between neurohormal signaling, lipid metabolism, and proteostasis. Two specific examples are Higuchi-Sanabria (2020) and Joshi (2021). This is a simple fix: remove the phrase "for the first time" from the abstract.

Referee #2:

The authors have experimentally addressed the protein expression levels of Q40-YFP in the different genetic backgrounds. The data are presented in the Appendix S1C-D. The western blots lack the molecular weight marker and that should be added - or the original blots should be provided. Depicted below is a graph of the quantification of three biological replicates. I see error bars, but no statistical analysis. These data need to be analysed by ANOVA etc to conclude whether or not the protein levels are unchanged.
Throughout their figures the authors use wildtype (WT) to refer to the parental Q40-YFP strain. This needs to be corrected. A WT is e.g. the N2 strain, but certainly not a transgenic line.

This reviewer is still puzzled that only one hit was identified in their screen and that this hit has not been identified before (van Ham et al for example). Moreover, why have the authors not identified any other hit from the previous screen by van Ham et al?

Where is the difference in the screen set up?

My previous question what is actually sensed by the IL2 neurons was a bit brushed away and given that the connection between lipid and protein homeostasis has been studied for a while, I would have expected at least an attempt to try to identify the signal and or signalling cascade by e.g. studying the role of endocrine signalling, neuropeptides etc.

Referee #3:

The authors addressed the reviewers' suggestions and questions. The additional data support their interpretation, and the text and Figures' presentation are improved considerably. Specifically, the revision strengthens the link between unc-1 and ssu-1,

and extends the impact of NHR-1 on proteostasis, supporting the authors' proposal that UNC-1 modulates SSU-1, which activates NHR-1-regulation of fat metabolism and proteostasis. While the data support the link between fat metabolism and proteostasis, how this is mediated remains an open question. However, the authors address this point in the discussion.

Minor comment: The text needs another round of editing. For example:
Introduction (last paragraph): "We also show showed that signalling through the well-known DAF-12 functions"

Discussion (page 16): Here, we showed that the enzymes involved in oleic acid synthesis are downregulated in unc-1 mutants and that oleic acid is effective reducing polyQ aggregation here"

Referee #2:

The authors have experimentally addressed the protein expression levels of Q40-YFP in the different genetic backgrounds. The data are presented in the Appendix S1C-D. The western blots lack the molecular weight marker and that should be added - or the original blots should be provided.
We thank the referee for this suggestion. Hence, we have included the molecular weight marker to the WB figure (please, see the revised Appendix Figure S1C).

Depicted below is a graph of the quantification of three biological replicates. I see error bars, but no statistical analysis. These data need to be analysed by ANOVA etc to conclude whether or not the protein levels are unchanged.
We have performed ANOVA test with posthoc Tukey analysis, and as expected there is not statistical differences between samples. We have stated that we performed statistics, and that they produced these results, within the figure legend.

Throughout their figures the authors use wildtype (WT) to refer to the parental Q40-YFP strain. This needs to be corrected. A WT is e.g. the N2 strain, but certainly not a transgenic line.
We agree that this could be confusing for some readers. We have edited to state the specific genetic background of each strain

This reviewer is still puzzled that only one hit was identified in their screen and that this hit has not been identified before (van Ham et al for example). Moreover, why have the authors not identified any other hit from the previous screen by van Ham et al?
Van Ham et al., were looking strictly for suppressors of polyQ aggregation in 40Q::YFP worms. The mutation in *unc-1* enhances polyQ aggregation in this strain. Hence, even if they produced a lesion in *unc-1*, they wouldn't pay attention to it.

Where is the difference in the screen set up?
Although we found a suppressor (see new Table S1 from Appendix), we were more interested in enhancers. And as stated above, Van Ham et al., were looking exclusively for suppressors of polyQ aggregation, so even if they produced such a mutation, they would not have isolated it.

My previous question what is actually sensed by the IL2 neurons was a bit brushed away and given that the connection between lipid and protein homeostasis has been studied for a while, I would have expected at least an attempt to try to identify the signal and or signalling cascade by e.g. studying the role of endocrine signalling, neuropeptides etc.
We agree with the referee that this is an important question, but we do not know and we do not have the technology to find out, right now. We have added a phrase to point out this limitation: *"What is sensed by ASJ and/or IL2, and why these upstream signals regulate, is unknown to us, and it will be very interesting to study them in the future."*

Regarding the hormone coming out of ASJ, we also do not know. In any case, two years ago we did experimental work to look for the hormone secreted by ASL, using all sort of metabolomics approaches. But, although we spent lots of resources and lots of months of work, we never managed to identify it. When we do more work in the future, to look for the hormone, we may need new approaches and different technologies that we have not within our reach at the moment. In any case, not knowing the signal does not change the message of the paper, in our opinion. We hope that the referee and editor agree with us. But for the sake of transparency, we have added the following phrases to the manuscript: *“An important remaining question is the nature of the sulphated signal released by ASJ. We have used multiple metabolomic approaches to try to identify this putative hormone, but this may require technological advances currently outside our reach“.*

Dr. Rafael Vazquez-Manrique
Health Research Institute-La Fe
GI Molecular, Cellular and Genomic Biomedicine
Avenida de Frenando Abril Martorell, 106
Torre A, Lab. 4.09
Valencia 46026
Spain

Dear Rafael,

I am very pleased to accept your manuscript for publication in the next available issue of EMBO reports. Thank you for your contribution to our journal.

At the end of this email I include important information about how to proceed. Please ensure that you take the time to read the information and complete and return the necessary forms to allow us to publish your manuscript as quickly as possible.

As part of the EMBO publication's Transparent Editorial Process, EMBO reports publishes online a Review Process File to accompany accepted manuscripts. As you are aware, this File will be published in conjunction with your paper and will include the referee reports, your point-by-point response and all pertinent correspondence relating to the manuscript.

If you do NOT want this File to be published, please inform the editorial office within 2 days, if you have not done so already, otherwise the File will be published by default [contact: emboreports@embo.org]. If you do opt out, the Review Process File link will point to the following statement: "No Review Process File is available with this article, as the authors have chosen not to make the review process public in this case."

Should you be planning a Press Release on your article, please get in contact with emboreports@wiley.com as early as possible, in order to coordinate publication and release dates.

Thank you again for your contribution to EMBO reports and congratulations on a successful publication. Please consider us again in the future for your most exciting work.

Kind regards,

Martina

Martina Rembold, PhD
Senior Editor
EMBO reports

****

THINGS TO DO NOW:

Please note that you will be contacted by Wiley Author Services to complete licensing and payment information. The required 'Page Charges Authorization Form' is available here: https://www.embopress.org/pb-assets/embo-site/er_apc.pdf - please download and complete the form and return to embopressproduction@wiley.com

EMBO Press participates in many Publish and Read agreements that allow authors to publish Open Access with reduced/no publication charges. Check your eligibility: https://authorservices.wiley.com/author-resources/Journal-Authors/open-access/affiliation-policies-payments/index.html

You will receive proofs by e-mail approximately 2-3 weeks after all relevant files have been sent to our Production Office; you should return your corrections within 2 days of receiving the proofs.

Please inform us if there is likely to be any difficulty in reaching you at the above address at that time. Failure to meet our deadlines may result in a delay of publication, or publication without your corrections.

All further communications concerning your paper should quote reference number EMBOR-2022-55556V3 and be addressed to emboreports@wiley.com.

Should you be planning a Press Release on your article, please get in contact with emboreports@wiley.com as early as possible, in order to coordinate publication and release dates.

EMBO Press Author Checklist

Corresponding Author Name: Rafael P Vázquez-Manrique	
Journal Submitted to: EMBO Reports	
Manuscript Number: EMBOR-2022-55556V1	

USEFUL LINKS FOR COMPLETING THIS FORM
The EMBO Journal - Author Guidelines
EMBO Reports - Author Guidelines
Molecular Systems Biology - Author Guidelines
EMBO Molecular Medicine - Author Guidelines

Reporting Checklist for Life Science Articles (updated January 2022)
This checklist is adapted from Materials Design Analysis Reporting (MDAR) Checklist for Authors. MDAR establishes a minimum set of requirements in transparent reporting in the life sciences (see Statement of Task: 10.31222/osf.io/9sm4x). Please follow the journal's guidelines in preparing your manuscript.
Please note that a copy of this checklist will be published alongside your article.

Abridged guidelines for figures
1. Data
The data shown in figures should satisfy the following conditions:
- the data were obtained and processed according to the field's best practice and are presented to reflect the results of the experiments in an accurate and unbiased manner.
- ideally, figure panels should include only measurements that are directly comparable to each other and obtained with the same assay.
- plots include clearly labeled error bars for independent experiments and sample sizes. Unless justified, error bars should not be shown for technical replicates.
- if n<5, the individual data points from each experiment should be plotted. Any statistical test employed should be justified.
- Source Data should be included to report the data underlying figures according to the guidelines set out in the authorship guidelines on Data Presentation.

2. Captions
Each figure caption should contain the following information, for each panel where they are relevant:
- a specification of the experimental system investigated (eg cell line, species name).
- the assay(s) and method(s) used to carry out the reported observations and measurements.
- an explicit mention of the biological and chemical entity(ies) that are being measured.
- an explicit mention of the biological and chemical entity(ies) that are altered/varied/perturbed in a controlled manner.
- the exact sample size (n) for each experimental group/condition, given as a number, not a range;
- a description of the sample collection allowing the reader to understand whether the samples represent technical or biological replicates (including how many animals, litters, cultures, etc.).
- a statement of how many times the experiment shown was independently replicated in the laboratory.
- definitions of statistical methods and measures:
  - common tests, such as t-test (please specify whether paired vs. unpaired), simple χ^2 tests, Wilcoxon and Mann-Whitney tests, can be unambiguously identified by name only, but more complex techniques should be described in the methods section;
  - are tests one-sided or two-sided?
  - are there adjustments for multiple comparisons?
  - exact statistical test results, e.g., P values = x but not P values < x;
  - definition of 'center values' as median or average;
  - definition of error bars as s.d. or s.e.m.

Please complete ALL of the questions below.
Select "Not Applicable" only when the requested information is not relevant for your study.

Materials

Newly Created Materials	Information included in the manuscript?	In which section is the information available? (Reagents and Tools Table, Materials and Methods, Figures, Data Availability Section)
New materials and reagents need to be available; do any restrictions apply?	Not Applicable	

Antibodies	Information included in the manuscript?	In which section is the information available? (Reagents and Tools Table, Materials and Methods, Figures, Data Availability Section)
For **antibodies** provide the following information:<br>- Commercial antibodies: RRID (if possible) or supplier name, catalogue number and or/clone number<br>- Non-commercial: RRID or citation	Yes	Material and Methods

DNA and RNA sequences	Information included in the manuscript?	In which section is the information available? (Reagents and Tools Table, Materials and Methods, Figures, Data Availability Section)
**Short novel DNA or RNA including primers, probes**: provide the sequences.	Yes	Appendix section

Cell materials	Information included in the manuscript?	In which section is the information available? (Reagents and Tools Table, Materials and Methods, Figures, Data Availability Section)
**Cell lines:** Provide species information, strain. Provide accession number in repository **OR** supplier name, catalog number, clone number, and/**OR** RRID.	Not Applicable	
**Primary cultures:** Provide species, strain, sex of origin, genetic modification status.	Not Applicable	
Report if the cell lines were recently **authenticated** (e.g., by STR profiling) and tested for mycoplasma contamination.	Not Applicable	

Experimental animals	Information included in the manuscript?	In which section is the information available? (Reagents and Tools Table, Materials and Methods, Figures, Data Availability Section)
**Laboratory animals or Model organisms:** Provide species, strain, sex, age, genetic modification status. Provide accession number in repository **OR** supplier name, catalog number, clone number, **OR** RRID.	Yes	Table S3_Appendix
**Animal observed in or captured from the field:** Provide species, sex, and age where possible.	Not Applicable	
Please detail **housing and husbandry conditions**.	Yes	Material and Methods

Plants and microbes	Information included in the manuscript?	In which section is the information available? (Reagents and Tools Table, Materials and Methods, Figures, Data Availability Section)
**Plants:** provide species and strain, ecotype and cultivar where relevant, unique accession number if available, and source (including location for collected wild specimens).	Not Applicable	
**Microbes:** provide species and strain, unique accession number if available, and source.	Not Applicable	

Human research participants	Information included in the manuscript?	In which section is the information available? (Reagents and Tools Table, Materials and Methods, Figures, Data Availability Section)
If collected and within the bounds of privacy constraints report on age, sex and gender or ethnicity for all study participants.	Not Applicable	

Core facilities	Information included in the manuscript?	In which section is the information available? (Reagents and Tools Table, Materials and Methods, Figures, Data Availability Section)
If your work benefited from core facilities, was their service mentioned in the acknowledgments section?	Yes	Acknowledgements

Design

Study protocol	Information included in the manuscript?	In which section is the information available? (Reagents and Tools Table, Materials and Methods, Figures, Data Availability Section)
If study protocol has been **pre-registered, provide DOI in the manuscript**. For clinical trials, provide the trial registration number **OR** cite DOI.	Not Applicable	
Report the **clinical trial registration number** (at ClinicalTrials.gov or equivalent), where applicable.	Not Applicable	

Laboratory protocol	Information included in the manuscript?	In which section is the information available? (Reagents and Tools Table, Materials and Methods, Figures, Data Availability Section)
Provide DOI OR other citation details if **external detailed step-by-step protocols** are available.	Not Applicable	

Experimental study design and statistics	Information included in the manuscript?	In which section is the information available? (Reagents and Tools Table, Materials and Methods, Figures, Data Availability Section)
Include a statement about **sample size** estimate even if no statistical methods were used.	Yes	Figure legends
Were any steps taken to minimize the effects of subjective bias when allocating animals/samples to treatment (e.g. **randomization procedure**)? If yes, have they been described?	Yes	Material and Methods
Include a statement about **blinding** even if no blinding was done.	Yes	Material and Methods
Describe **inclusion/exclusion criteria** if samples or animals were excluded from the analysis. Were the criteria pre-established?<br><br>If sample or data points were omitted from analysis, report if this was due to attrition or intentional exclusion and provide justification.	Not Applicable	
For every figure, are **statistical tests** justified as appropriate? Do the data meet the assumptions of the tests (e.g., normal distribution)? Describe any methods used to assess it. Is there an estimate of variation within each group of data? Is the variance similar between the groups that are being statistically compared?	Yes	Figure legends

Sample definition and in-laboratory replication	Information included in the manuscript?	In which section is the information available? (Reagents and Tools Table, Materials and Methods, Figures, Data Availability Section)
In the figure legends: state number of times the experiment was **replicated** in laboratory.	Yes	Figure legends
In the figure legends: define whether data describe **technical or biological replicates**.	Yes	Figure legends

Ethics

Ethics	Information included in the manuscript?	In which section is the information available? (Reagents and Tools Table, Materials and Methods, Figures, Data Availability Section)
Studies involving **human participants**: State details of **authority granting ethics approval** (IRB or equivalent committee(s), provide reference number for approval.	Not Applicable	
Studies involving **human participants**: Include a statement confirming that **informed consent** was obtained from all subjects and that the experiments conformed to the principles set out in the WMA Declaration of Helsinki and the Department of Health and Human Services Belmont Report.	Not Applicable	
Studies involving **human participants:** For publication of **patient photos**, include a statement confirming that consent to publish was obtained.	Not Applicable	
Studies involving experimental **animals**: State details of **authority granting ethics approva**l (IRB or equivalent committee(s), provide reference number for approval. Include a statement of compliance with ethical regulations.	Not Applicable	
Studies involving **specimen and field samples:** State if relevant **permits** obtained, provide details of authority approving study; if none were required, explain why.	Not Applicable	

Dual Use Research of Concern (DURC)	Information included in the manuscript?	In which section is the information available? (Reagents and Tools Table, Materials and Methods, Figures, Data Availability Section)
Could your study fall under dual use research restrictions? Please check biosecurity documents and list of **select agents and toxins** (CDC): https://www.selectagents.gov/sat/list.htm	Not Applicable	
If you used a select agent, is the security level of the lab appropriate and reported in the manuscript?	Not Applicable	
If a study is subject to dual use research of concern regulations, is the name of the **authority granting approval and reference number** for the regulatory approval provided in the manuscript?	Not Applicable	

Reporting

The MDAR framework recommends adoption of discipline-specific guidelines, established and endorsed through community initiatives. Journals have their own policy about requiring specific guidelines and recommendations to complement MDAR.

Adherence to community standards	Information included in the manuscript?	In which section is the information available? (Reagents and Tools Table, Materials and Methods, Figures, Data Availability Section)
State if relevant guidelines or checklists (e.g., **ICMJE, MIBBI, ARRIVE, PRISMA**) have been followed or provided.	Not Applicable	
For **tumor marker prognostic studies,** we recommend that you follow the **REMARK** reporting guidelines (see link list at top right). See author guidelines, under 'Reporting Guidelines'. Please confirm you have followed these guidelines.	Not Applicable	
For **phase II and III randomized controlled trials**, please refer to the **CONSORT** flow diagram (see link list at top right) and submit the CONSORT checklist (see link list at top right) with your submission. See author guidelines, under 'Reporting Guidelines'. Please confirm you have submitted this list.	Not Applicable	

Data Availability

Data availability	Information included in the manuscript?	In which section is the information available? (Reagents and Tools Table, Materials and Methods, Figures, Data Availability Section)
Have **primary datasets** been deposited according to the journal's guidelines (see 'Data Deposition' section) and the respective accession numbers provided in the Data Availability Section?	Yes	Data availability section
Were **human clinical and genomic datasets** deposited in a public access-controlled repository in accordance to ethical obligations to the patients and to the applicable consent agreement?	Not Applicable	
Are **computational models** that are central and integral to a study available without restrictions in a machine-readable form? Were the relevant accession numbers or links provided?	Not Applicable	
If publicly available data were reused, provide the respective **data citations in the reference list**.	Not Applicable	
